# Global Burden, Incidence and Disability-Adjusted Life-Years for Dermatitis: A Systematic Analysis Combined With Socioeconomic Development Status, 1990–2019

**DOI:** 10.3389/fcimb.2022.861053

**Published:** 2022-04-12

**Authors:** Yi Xue, Wu Bao, Jie Zhou, Qing-Liang Zhao, Su-Zhuang Hong, Jun Ren, Bai-Cheng Yang, Peng Wang, Bin Yin, Cheng-Chao Chu, Gang Liu, Chi-Yu Jia

**Affiliations:** ^1^ Department of Burns and Plastic and Wound Repair Surgery, Xiang’an Hospital of Xiamen University, School of Medicine, Xiamen University, Xiamen, China; ^2^ School of Medicine, Xiamen University, Xiamen, China; ^3^ State Key Laboratory of Molecular Vaccinology and Molecular Diagnostics and Center for Molecular Imaging and Translational Medicine, School of Public Health, Xiamen University, Xiamen, China; ^4^ Division of Plastic Surgery, Zhongshan Hospital Xiamen University, Xiamen, China; ^5^ Department of Dermatology, Zhongshan Hospital, Xiamen University, Xiamen, China; ^6^ Eye Institute of Xiamen University, Fujian Provincial Key Laboratory of Ophthalmology and Visual Science, Xiamen University, Xiamen, China

**Keywords:** dermatitis, socioeconomics, incidence rate, sociodemographic index, disability-adjusted life years, Global burden of disease study database

## Abstract

**Background:**

Dermatitis is an important global health problem that not only affects social interaction and physical and mental health but also causes economic burden. Health problems or distress caused by dermatitis may be easily overlooked, and relevant epidemiological data are limited. Therefore, a better understanding of the burden of dermatitis is necessary for developing global intervention strategies.

**Methods:**

All data on dermatitis, including atopic dermatitis (AD), contact dermatitis (CD) and seborrhoeic dermatitis (SD), were obtained from the Global Burden of Disease 2019 (GBD2019) database. The extracted age-standardized incidence rates (ASIR) and disability-adjusted life-years (DALYs) rates (ASDR) data were analysed by stratification, including by sex, country or region, and sociodemographic index (SDI) indicators. Finally, we analysed the correlation between the global burden of dermatitis and socioeconomic development status.

**Results:**

According to the GBD 2019 estimate, the ASIR and ASDR for the three major types of dermatitis in 2019 were 5244.3988 (95% CI 4551.7244–5979.3176) per 100,000 person-years and 131.6711 (95% CI 77.5876–206.8796) per 100,000 person-years. The ASIR and ASDR of atopic dermatitis, contact dermatitis and seborrhoeic dermatitis are: Incidence (95%CI,per 100,000 person-years), 327.91 (312.76-343.67), 3066.04 (2405.38-3755.38), 1850.44 (1706.25- 1993.74); DALYs (95%CI, per 100,000 person-years), 99.69 (53.09-167.43), 28.06 (17.62-41.78), 3.93 (2.24-6.25). In addition, among the three dermatitis types, the greatest burden was associated with AD. According to the ASDR from 1990 to 2019, the burden of dermatitis has exhibited a slow downward trend in recent years. In 2019, the ASIR showed that the USA had the greatest burden, while the ASDR showed that Asian countries (such as Japan, Mongolia, Kazakhstan, and Uzbekistan) and some European countries (France, Estonia) had the greatest burden. According to SDI stratification and the three major dermatitis types, high ASIR and ASDR corresponded to high SDI areas (especially for AD).

**Conclusion:**

The burden of dermatitis is related to socioeconomic development status, especially for AD, which is positively correlated with the SDI. The results based on GBD2019 data are valuable for formulating policy, preventing and treating dermatitis and reducing the global burden of dermatitis.

## Introduction

Dermatitis ranks first in global disease burden caused by skin diseases and mainly includes atopic dermatitis (AD), contact dermatitis (CD) and seborrhoeic dermatitis (SD). Of these types of dermatitis, AD has the greatest global burden, followed by SD and CD (James et al., 2018). In 2013, A recent study showed that the dermatitis (AD, CD, SD) burden accounted for 0.38% of the total disease burden (306 diseases and injuries)., far exceeding the disease burden caused by skin tumours, including 0.06% for malignant skin melanoma and 0.03% for keratinocyte carcinoma (Karimkhani et al., 2017). In 2017, the global incidence of dermatitis was approximately 274 million (James et al., 2018), but the mortality rate was very low. In a study in 1990–2017, the global incidence rate of dermatitis in 2007–2017 was 13.0%, which was far lower than the 24% in 1990–2007 (James et al., 2018). Among the 20major causes of disability worldwide (based on years lived with disability, YLD), the ranking of dermatitis has dropped from 18(1990) to 20(2017) (James et al., 2018). However, for dermatitis in the 0–9-year-old age group, the percentage of DALYs rose from 0.2 (0.1 to 0.3) in 1990 to 0.4 (0.2 to 0.7) in 2019, and its ranking among the major causes of disability worldwide rose from 45 to 20 (Vos et al., 2020).

The GBD 2019 includes existing evidence from 204 countries and regions on health levels and trends, various risk factors, and health system responses. Integrating data from 281,586 sources and providing 350 million estimates (global health outcomes or health system measures) provides a strong research foundation for detailed and extensive insights into global health trends and emerging challenges (Murray et al., 2020). Currently, research on the socioeconomic relationship between AD, CD and SD in the global burden of disease is lacking. In this study, we combined the latest data from GBD 2019 to systematically analyse the relationship between dermatitis and the SDI. Further measuring the changes in the burden of dermatitis from 1990 to 2019 and discussing the potential impact of such changes may have important implications for formulating global intervention strategies.

## Methods

### Overview and Data Sources

GBD 2019 includes 369 diseases and injuries in 204 countries or regions around the world as well as more than 80 behavioural, environmental and other risk factors (Vos et al., 2020). The latest data used to estimate the ASIR and ASDR of dermatitis were extracted (http://ghdx.healthdata.org/gbd-2019). According to the GBD world population, an age-standardized rate analysis was recorded per 100,000 person-years (Murray et al., 2018).

### DALYs

The loss of one year of healthy life is equivalent to a DALY, and the burden of disease is estimated based on DALYs (Almekhlafi et al., 2021). DALYs is the sum of years of life lost (YLL) to a disease, and YLD is estimated for each reason, location, age group, sex and year in GBD 2019.

### SDI

The SDI (http://ghdx.healthdata.org/gbd-2019) is a comprehensive indicator that reflects the development of society and the population. It is the geometric mean of the normalized value of the regional per capita income, the number of years of education of those 15 years old and above, and the total fertility rate (TFR) of women under 25. In GBD 2015, the original SDI was constructed using the Human Development Index methodology; the use of the SDI is also described in detail in GBD 2016 (Wang et al., 2017), and it can be used to estimate a summary measure of the location within the development range (James et al., 2018). The overall status of socioeconomic development can be stratified by the SDI. GBD 2019 divides countries into five categories based on SDI indicators: high SDI, high-middle SDI, middle SDI, low-middle SDI and low SDI.

### Uncertainty Analysis

In this study, it is assumed that the incidences in different years are independent of each other and that the incidence and DALYs rates of each year are log-normally distributed. The 2.5th and 97.5th percentiles of the draw level values represent the 95% uncertainty interval. Prism 8.0.1 software was used to draw related graphics. SPSS 23.0 for statistical analysis. *P*<0.05 (two tailed) was statistically significant.

### Role of the Funding Source

In this study, the Bill & Melinda Gates Foundation had no role in the research design and conduct, data collection, data sorting, data analysis, preparation, revision, submission, publication or interpretation of the manuscript. The authors had full access to the data in the study and final responsibility for the decision to submit for publication.

## Results

### Burden of Major Types of Dermatitis

**Figure 1:**
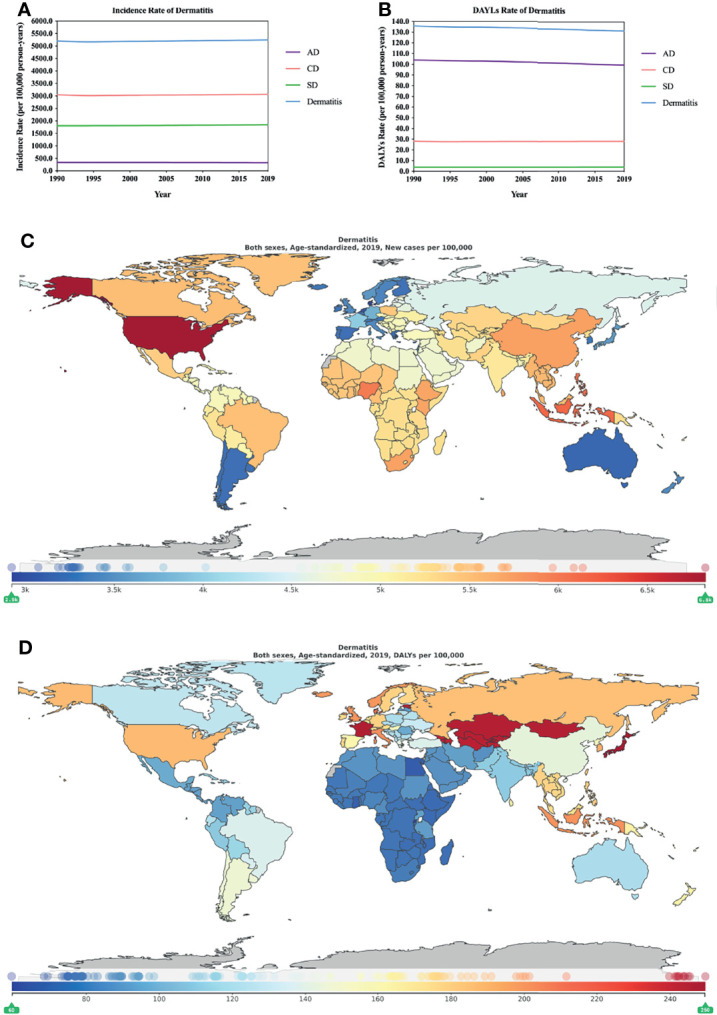
Burden of dermatitis for 204 countries and territories. ASIR **(A)** and ASDR **(B)** per 100,000 people (1990–2019) with dermatitis by country and region. The distribution of ASIR **(C)** and ASDR **(D)** per 100,000 population with dermatitis globally in 2019. **(C, D)** were generated by GDB2019.

From 1990 to 2019, according to the ASIR, the incidence of dermatitis rose (0.77% [0.45–1.11], **Supplementary Table 1**). CD ranked first out of the three major dermatitis types, followed by SD and AD (**Figure 1A**). In addition, the ASIR of the three major dermatitis types basically remained stable from 1990 to 2019 (**Figure 1A**; **Supplementary Table 2**), while AD (-4.20% [-3.58 to - 4.81]) showed a slight downward trend and CD (0.42% [-0.07 to 0.94]) and SD (2.30% [1.97–2.61]) showed an upward trend. The increase in the ASIR for SD was higher than that for CD (more than 5 times). The ASDR of the three major types of dermatitis had a similar trend to the age-standardized incidence rates, which basically maintained a stable state from 1990 to 2019. The difference is that AD ranked first among the three types of dermatitis, followed by CD and SD (**Figure 1B**). Furthermore, SD (2.70% [1.85–3.54]) showed a slight upward trend, AD (-4.14% [- 4.75 to -3.50]) and CD (-0.10% [-0.94 to 0.76]) both showed a downward trend, and the downward trend of AD was more than 4 times that of CD (**Figure 1B**; **Supplementary Table 3**). In 2019, the ASIR was 5244.3988 (4551.7244–5979.3176) (**Supplementary Table 4**), and the ASDR was 131.6711 (77.5876–206.8696) (**Supplementary Table 5**) per 100,000 person-years in terms of the three dermatitis types. The USA (6824.4648 [5904.7653–7797.1826] per 100,000 person-years) showed the greatest burden of the three major types of dermatitis in terms of the age-standardized incidence rate in 2019. Some Asian countries (including Indonesia, the Philippines, and China), some countries in Africa (Nigeria, South Africa, and Kenya) and Brazil in South America also showed a high burden (**Figure 1C**; **Supplementary Table 6**). The ASDR analysis in 2019 showed that several countries, such as Japan (249.5939 [135.8556–416.6162] per 100,000 person-years), Estonia, France, Mongolia, Kazakhstan, Tajikistan, Uzbekistan, Armenia, Turkmenistan and Azerbaijan, showed great global burdens associated with the three major dermatitis types (**Figure 1D**; **Supplementary Table 7**).

### Burden of Atopic Dermatitis

AD, also called atopic eczema and eczema (Langan et al., 2020), is a common inflammatory skin disease and has become the major factor in the global burden of skin diseases. The most common features of AD are pruritus, lichenification and xerosis (Yew et al., 2019), which greatly affect the social and mental health of patients and their families. Moreover, AD patients have a higher depressive ratio than people without AD (20.1% vs. 14.8%) (Patel et al., 2019; Sandhu et al., 2019). In 2017, the estimated number of new patients with AD was 27 million, ranking second among the three types of dermatitis (James et al., 2018). The prevalence of AD may vary by race: in the USA, the prevalence among whites (11%) is lower than that among African Americans (17%) (Kim et al., 2019; Wan et al., 2019); the prevalence of AD in infancy in China is as high as 30.48% (Guo et al., 2019); and the incidence and persistence of AD are higher in certain non-white racial/ethnic subgroups than in non-Hispanic whites (Kim et al., 2019). Although the mortality rate for AD is very low, some studies report that the long-term risk of atrial fibrillation in AD patients increases by 20% (Schmidt et al., 2020). To date, local treatment alone or in combination with phototherapy can control most AD, but moderate to severe AD requires systemic immune regulation to be fully controlled (Siegels et al., 2021). Systemic medication or treatment for AD may increase economic expenditure, leading to increased burden.

**Figure 2:**
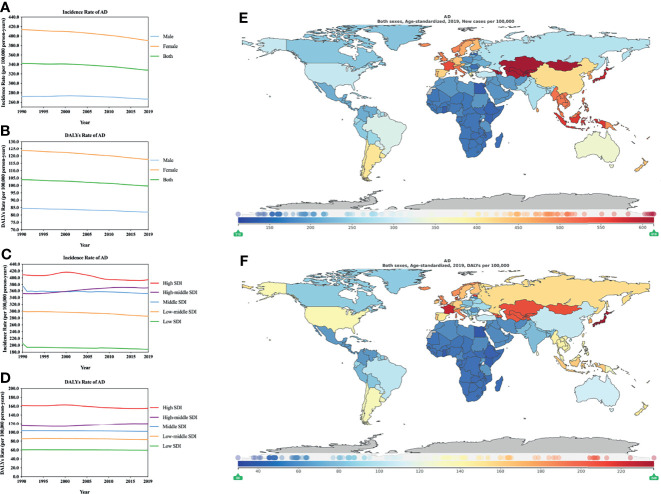
Burden of AD for 204 countries and territories. ASIR (**A**, by sex; **C**, by SDI) and ASDR (**B**, by sex; **D**, by SDI) per 100,000 population of AD (1990–2019) by country and region. The distribution of ASIR **(E)** and ASDR **(F)** per 100,000 population of AD globally in 2019. **(E, F)** were generated by GDB2019.

According to the estimates of GBD 2019, the ASIR for AD is the lowest among the three diseases, and the ASDR for AD is the highest among the three dermatitis types. In further analysis, females showed a higher ASIR and ASDR for AD than males (**Figures 2A, B**; **Supplementary Tables 8**, **9**), which is similar to the results of previous studies (Kim et al., 2016). A meta-analysis of data from 15 countries found that female AD patients also have higher durability than male patients (P ≤.0006) (Kim et al., 2016). However, the prevalence of AD in childhood (slightly higher among males than females) is different from that after puberty (females higher than males) (Kanda et al., 2019). This reversal may be due to the effect of sex hormones on the immune response and skin penetration barrier (Kanda et al., 2019). In addition, the increase in the burden of AD is on the same order as the increase in the SDI, in which high SDI areas record high AD ASIR and ASDR and low SDI areas record low AD ASIR and ASDR (**Figures 2C, D**; **Supplementary Tables 10**, **11**). We performed regression analysis on the ASIR and ASDR of AD and 5 SDI regions with different economic status in 2019, and the results were statistically significant (incidence, R^2 =^ 0.894, *P*=0.015; DALYs, R^2 =^ 0.983, *P*=0.001). Moreover, previous studies have suggested that adults (21%) and children (10%) are more likely to be affected by AD in high-income countries (Odhiambo et al., 2009; Silverberg and Hanifin, 2013).

The ASIR of AD increased in high-middle SDI (4.59%[3.67–5.56], **Supplementary Table 2**) locations from 1990 to 2019, while the remaining SDI areas showed a downward trend. It is worth mentioning that in the initial stage, the number of AD patients in the middle SDI areas gradually decreased over time, and the number of AD patients in the high-middle SDI areas gradually increased. The two formed a meeting point in approximately 2000, after which the high-middle SDI areas gradually surpassed the middle SDI areas (**Figure 2C**; **Supplementary Table 10**).

During the period from 1990 to 2019, among the ASDR of AD, the high SDI, low-middle SDI, middle SDI and Low SDI locations all showed a downward trend, of which the middle SDI regions declined the least (-1.57% [- 2.74 to -0.55], **Supplementary Table 3**). However, ASDR were increased in high-middle SDI regions (4.39% [3.26–5.56], **Supplementary Table 3**). The high SDI areas had the greatest decline (-3.84% [-4.74 to -2.95], **Supplementary Table 3**). In 2019, the ASIR for AD showed great differences among countries or regions (**Figure 2E**; **Supplementary Table 12**). The countries with the greatest burdens were mainly distributed in Asia. Mongolia (613.4804 [542.4961–694.0930] per 100,000 person-years) suffered the greatest burden, followed by Kazakhstan, Kyrgyzstan, Uzbekistan, and Tajikistan (**Supplementary Table 12**). The countries with lower burdens were mainly distributed in Africa; the country with the lowest burden was Rwanda (112.0677 [104.2422–120.4800] per 100,000) person-years). Similarly, the ASDR for AD varied greatly in different countries or regions. The highest ASIR were in Europe and Asia (**Figure 2F**; **Supplementary Table 13**). In 2019, the area with the highest ASDR (Japan, 236.1474 [126.3096–396.9329] per 100,000 person-years) was 7.8 times that of the lowest area (Rwanda, 30.0303 [16.0893–50.7356] per 100,000 person-years).

### Burden of Contact Dermatitis

CD is another common inflammatory skin disease that includes many types, such as allergic CD (ACD), photoallergic CD, irritant CD, and photoirritant CD. Irritant CD is the most common type of CD, accounting for approximately 80% of CD (Fonacier et al., 2015), while ACD accounts for approximately 20% of CD (Rashid and Shim, 2016). The estimated incidence of CD in 2017 was 221 million, and the prevalence of CD was 79 million (James et al., 2018). The percentage change in age-standardized rates of YLDs (thousands) dropped from -1.6% (-2.3 to -1.0) in 1990–2007 to -1.1% (-1.7 to -0.5) in 2007–2017 (James et al., 2018). In the past, it was thought that ACD was rare in children, but recently, it was estimated that 4.4 million children are affected in the USA alone, and this number is increasing (Borok et al., 2019). Patients with CD have eczema reactions such as erythema, blisters, and exudates. Severe or stubborn CD affects the quality of life of patients and requires systemic medication. In addition, CD may impose significant emotional, social, economic, and professional burdens on patients (Milam and Cohen, 2019). However, the primary immunological characteristics of different types of CD are different; for example, irritant CD causes direct cellular damage, while ACD is a type IV hypersensitivity reaction (Scheinman et al., 2021). The relevant clinical population is selected based on a high degree of visits for the patch test; however, the incidence and prevalence of ACD in the general population are difficult to estimate.

**Figure 3:**
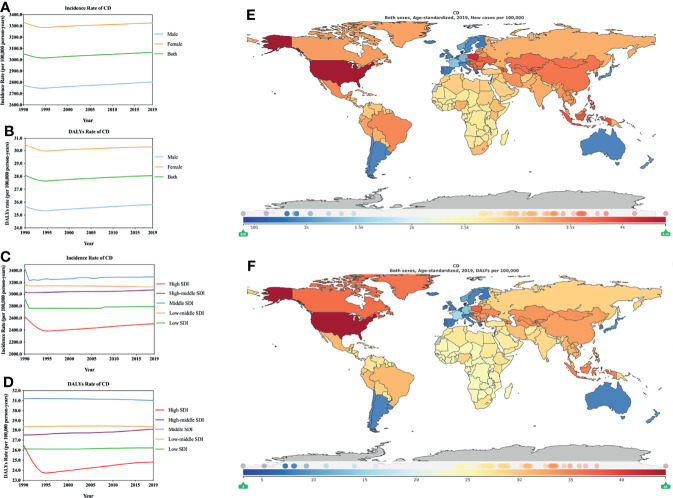
Burden of CD for 204 countries and territories. ASIR (**A**, by sex; **C**, by SDI) and ASDR (**B**, by sex; **D**, by SDI) per 100,000 population of CD (1990–2019) by country and region. The distribution of ASIR **(E)** and ASDR **(F)** per 100,000 population of CD globally in 2019. Figures **(E, F)** were generated by GDB2019.

In GBD 2019, the ASIR and ASDR for CD and AD showed fewer males than females (**Figure 3A, B**), similar to previous reports that men were less likely to be diagnosed with CD than women (Thyssen et al., 2010; de Waard-van der Spek et al., 2013; Malik and English, 2015). However, a recent study suggested that nearly one-third of patients (31.3%, n = 10,888) had a negative patch test (NPT) and that patients were more likely to be male (P <.0001) (Warshaw et al., 2019). Therefore, it is unclear whether the existence of NPT may indirectly cause statistical bias, leading to a statistically low incidence of male CD. In 1990–2019, CD’s ASIR change (95% CI, **Supplementary Table 2**) showed an increase in males (1.03% [0.48–1.51]) and a decrease in females (-0.20% [-0.84 to -0.47]). CD’s ASDR change (95% CI, **Supplementary Table 3**) also showed an increase in males (0.58% [-0.49 to 1.64]) and a decrease in females (-0.76% [-1.81 to -0.24]). In 2019, the ASIR was 3066.0421 (2405.3768–3755.3806) (**Figure 3A**; **Supplementary Table 14**), and the ASDR was 28.0575 (17.6212–41.7765) (**Figure 3B**; **Supplementary Table 15**) per 100,000 person-years in terms of the three types of dermatitis. According to SDI stratification and the ASIR and ASDR of CD, high SDI locations had the lowest rates, whereas the middle SDI locations recorded the highest rates (**Figures 3C, D**; **Supplementary Tables 16**, **17**). From 1990 to 2019, CD’s ASIR (**Figure 3C**; **Supplementary Table 2**) increased in the high-middle SDI regions (1.33% [0.68–1.98]), while those in the other SDI regions decreased. CD’s ASDR (**Figure 3D**; **Supplementary Table 3**) implied that there was basically no change in the low-middle SDI areas (0.00% [-1.24 to 1.28]) and an increase in the high-middle SDI areas (2.14% [0.66–3.58]) and low SDI areas (0.36% [-1.16 to 1.68]). In the high SDI areas (-6.45% [-8.02 to -4.79]) and the middle SDI areas (-0.60% [-1.64 to 0.40]), there was a decrease; the decrease in the high SDI areas was 10 times that of the middle SDI areas. Similar to AD, the ASIR and ASDR of CD differed in different countries or regions. According to the ASIR (per 100,000 person-years) in 2019 (**Figure 3E**; **Supplementary Table 18**), the greatest burden associated with CD was in the USA (4408.2936 [3514.1266–5389.1386]), and the lowest burden was in Denmark (395.7862 [308.7401–488.5793]). For CD’s ASDR (per 100,000 person-years) in 2019 (**Figure 3F**; **Supplementary Table 19**), the country with the greatest burden was also the USA (44.2739 [28.7410–64.7999]), and the country with the lowest burden was Denmark (3.1671 [1.9858–4.9095]). Furthermore, the ASIR and ASDR for the highest country was 10 times that of the lowest country.

### Burden of Seborrhoeic Dermatitis

SD is also a common chronic or recurrent inflammatory skin disease. In 2017, the estimated incidence of SD globally was approximately 25.6 million, and the prevalence of SD was approximately 10 million (James et al., 2018). The YLD of SD dropped from 20.8% (1990–2007) to 8.4% (2007–2017) (James et al., 2018). According to research reports, approximately 1–3% of the general population in the USA is affected by SD, of whom 3–5% of patients are young people, and the prevalence of SD is approximately 20–83% among HIV-positive individuals (Schechtman et al., 1995). The development of SD is related to many risk factors, such as sebum activity, host immunity (especially HIV infection), epidermal barrier integrity, skin microbiota, endocrine and nervous system factors, Malassezia spp. and environmental influences. In addition, the susceptibility of an individual to the development of SD is determined by the interaction of many factors (Wikramanayake et al., 2019). In a recent study, human T-cell lymphotropic virus type 1 infection was suggested to be associated with an increase in the prevalence of SD (OR 3.95, 95% CI [1.99–7.81]) (Schierhout et al., 2020). Red scaly rash is a characteristic of SD, although the pathophysiology of SD remains poorly understood. SD may affect the sebaceous glands in areas such as the face and scalp and is mainly treated by the use of antifungal agents. Since short term treatment anti-inflammatory drugs (such as topical corticosteroids) may have side effects and are only used in the short term (Clark et al., 2015), the improvement of modifiable lifestyle factors may help reduce the burden of disease (Sanders et al., 2019) Furthermore, approximately 50% of SD patients in China have serious emotional problems, which may substantially affect their quality of life (Xuan et al., 2020). This warrants global public health attention to SD.

**Figure 4:**
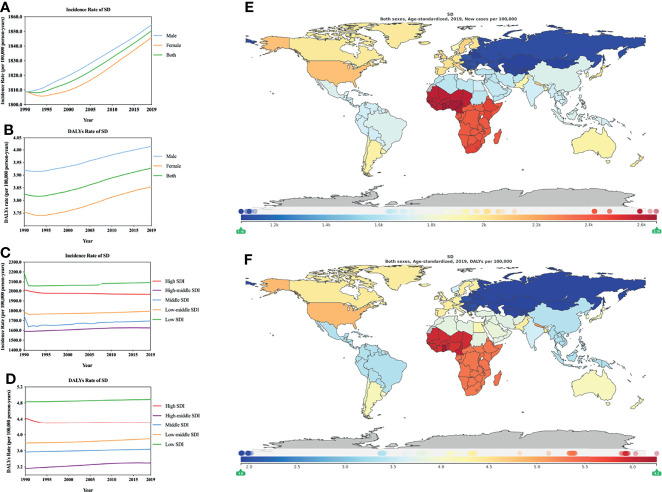
Burden of SD for 204 countries and territories. ASIR (**A**, by sex; **C**, by SDI) and ASDR (**B**, by sex; **D**, by SDI) per 100,000 population of SD (1990–2019) by country and region. The distribution of ASIR **(E)** and ASDR **(F)** per 100,000 population of SD globally in 2019. Figures **(E, F)** were generated by GDB2019.

According to the GBD 2019 ASIR and ASDR estimates, males had a higher SD burden than females, which differed from those of AD and CD (**Figures 4A, B**; **Supplementary Tables 20**, **21**). A study found that in Germany men (4.6%) are more susceptible to SD than women (1.4%) (Zander et al., 2019). A recent study also showed that men with light and dry skin are more likely to suffer from SD (Sanders et al., 2018). However, a study of data from outpatients in nine hospitals in China found that 67.3% of SD patients were female (Xuan et al., 2020). The ASIR and ASDR of SD showed an increasing trend for both males and females from 1990 to 2019. During 1990–2019, the changes in global ASIR (**Supplementary Table 2**) and ASDR rates (**Supplementary Table 3**) were estimated. The incidence rate of males was higher than that of females, but the opposite was true for DALYs: incidence, male (2.54% [2.16–2.90]) vs. female (2.02% [1.62–2.45]); DALYs, male (2.41% [1.33–3.47]) vs. female (2.66% [1.50–3.80]). From 1990 to 2019, we found that the highest ASIR and ASDR were in low SDI locations, followed by high SDI, low-middle SDI, middle SDI, and high-middle SDI locations (**Figures 4C, D**; **Supplementary Tables 22**, **23**). Except for low-middle SDI locations (0.48% [0.26–0.69], per 100,000 person-years) and high-middle SDI regions (2.17% [1.94–2.40], per 100,000 person-years), the ASIR of SD (**Figure 4C**; **Supplementary Table 2**) is rising. Other regions, including high SDI, middle SDI, and low SDI regions, all showed a downward trend, while ASDR, with the exception of high SDI regions (-2.67% [-4.05 to -1.35], per 100,000 person-years), declined, and the rest of the SDI regions exhibited increased ASDR from 1990–2019 (**Figure 4D**; **Supplementary Table 3**).

In 2019, the ASDR for SD and ASIR showed that SD varied greatly in different countries or regions (**Figures 4E, F**; **Supplementary Tables 24**, **25**).The countries with greater burdens are mainly distributed in Africa, especially in southern Africa, and the countries with lower burdens are mainly distributed in Asia. The countries with the greatest burden of incidence include Ghana (2657.7505 [2424.7568–2899.2722] per 100,000 person-years), followed by Nigeria, Cameroon, Sierra Leone, and Liberia; the countries with the greatest burden of DALYs include Ghana (6.2480 [3.5740–9.8433] per 100,000 person-years), followed by Cameroon and Cabo Verde, which may have a certain relationship with the local geographic location, climate, economic development level, and health status. The countries with low ASIR and ASDR burden of SD are Kazakhstan (1073.1836 [989.2014–1157.3785] per 100,000 person-years) and the Republic of Moldova (1.9119 [1.0725–3.0522] per 100,000 person-years). We extracted and summarized the key data from dermatitis, AD, CD and SD research to facilitate the readability of the data (**Supplementary Table 26**). Additionally, we marked the countries and regions (**Supplementary Table 27**) with the highest ASIR and ASDR (in 2019) for dermatitis, AD, CD and SD in the SDI (values) table.

## Discussion

According to GBD 2019 data, we explored the global burden of dermatitis (AD, CD and SD) and its relationship with socioeconomic status and conducted relevant analysis based on the differences in the SDI. The main findings were as follows: 1) The type of dermatitis with the heaviest global burden in 2019, according to ASIR, was CD followed by SD and AD; according to ASDR, it was AD followed by CD and SD. 2) There is a positive correlation between AD and socioeconomics. 3) From the ASIR and ASDR, these types of dermatitis showed a very stable trend from 1990 to 2019. 4) In 2019, the global burden of different types of dermatitis varied significantly in different regions. Several countries or regions located in Asia, Africa, and North America had the heaviest burden of major dermatitis.

Dermatitis has a heavy burden on global healthcare costs and morbidity (Karimkhani et al., 2017). The economic burden caused by AD in different countries and regions differs. It was reported that AD in the USA caused an economic burden of approximately US$3,300 (direct costs and indirect costs) per person per year in 2013 for children (Filanovsky et al., 2016)and adults (Drucker et al., 2018). Research reports in nine European countries showed that moderate to severe AD caused an economic burden of 927€ (2018) (Zink et al., 2019). In the past 30 years, the ASIR and ASDR for AD have been declining, which also reflects the continuous progress of global treatment and control measures for AD. However, further efforts to reduce the burden in high-SDI countries and minimize the global incidence of AD are urgently needed. The American Academy of Dermatology estimates that 4.17% of Americans are affected by CD, and the cost of CD in 2013 was as high as $1.5 billion (Lim et al., 2017), which makes the USA the country with the heaviest CD burden in the world. In addition, the high burden of CD in the identified countries may be related to many potential factors: 1) Although the EU implemented the Nickel Directive, the release of nickel in the USA was not regulated. In a study of 44,908 patients in the North American Contact Dermatitis Group (NACDG) screening series between 1994 and 2014 who were patch tested, nickel contact allergies increased from 14.3% (1994–1996) to 20.1% (2013) (Warshaw et al., 2019). 2) With regard to allergen exposure, according to a retrospective cross-sectional analysis of 50,507 patients with NACDG data for 22 years (Warshaw et al., 2020), 60.5% of NACDG allergens were sourced from personal care products. Other CD allergens mainly include adhesives, dyes, drugs, metals, and preservatives (Militello et al., 2020). 3) According to the estimates of the American Contact Dermatitis Association, the recent COVID-19 pandemic will increase the incidence of hand irritant contact and allergic CD (Rundle et al., 2020). At present, there are few reports on the economic burden caused by SD. However, the Academy of Dermatology reported that the cost of SD was as high as $339 million, and the calculation of this cost does not include prescription or OTC drugs or screening, vaccines and other related services in 2013 (Lim et al., 2017). In addition, the main types of dermatitis (both AD and CD) occur more often among females than males, but SD occurs more often among males than females. The predominance of women with AD and CD may be related to cosmetic or allergen exposure, while the predominance of men with SD may be related to sex hormones.

The aetiology and pathogenesis of each type of dermatitis are different, so different strategies need to be adopted in prevention or treatment. For AD, new prevention strategies and therapies that specifically target the disease are of great significance. For CD, special attention should be given to patient education, avoiding exposure to specific substances and local treatment, and systemic treatment for severe or intractable CD. For SD, in addition to local medication or placebo control, long-term management strategies can be adopted, including nondrug treatment and simple interventions to remove scales. In addition to treating dermatitis, patients with HIV-related dermatitis must be actively treated for HIV. The recent COVID-19 pandemic may increase the chance of dermatitis (Rundle et al., 2020), especially for medical staff. The global burden of dermatitis not only is an economic or medical burden but also affects psychosocial and social interactions and may seriously affect quality of life. Many people in low-income countries suffer from severe dermatitis. In addition to the living environment and geographical location, this may be related to insufficient sanitation facilities or limited medical conditions. The areas with the heaviest burden of AD are high SDI areas, and improving lifestyle or diet may relieve this burden to a certain extent. In short, minimizing the burden of dermatitis is an important component of the global health strategy.

There are some limitations of this study. 1) GBD data are derived from estimation and mathematical modelling. 2) The possibility of the underestimation of the dermatitis burden, especially in low-middle and low SDI locations, is due to inadequate screening. 3) The inability to adjust these confounding factors, such as patients, providers, and geographic levels, may limit the universality and accuracy of the research results. 4) It is impossible to conduct an analysis according to the severity of dermatitis, and there is a lack of relevant available data. Although there are certain limitations, the estimation of GBD 2019 data is very valuable for the formulation of global dermatitis prevention and control policies and the implementation of effective intervention measures to improve or reduce the burden of global dermatitis.

## Data Availability Statement

The original contributions presented in the study are included in the article/**Supplementary Material**. Further inquiries can be directed to the corresponding authors.

## Author Contributions

C-YJ, GL, C-CC, and YX, study conception and design. YX, C-CC, and Q-LZ, data acquisition. WB, JR and S-ZH, data analysis. JZ, BY, BC-Y and PW drew the picture. C-YJ, study supervision. GL, administrative support. YX, drafting the paper. C-YJ, GL, C-CC, critical revision of the manuscript for important intellectual content. All authors contributed to the article and approved the submitted version.

## Funding

This study was supported by the Starting Package of Xiang’an Hospital of Xiamen University(PM201809170010), Open project of Provincial Key Laboratory of Union Hospital Affiliated to Fujian Medical University in 2020 (No. XHZDSYS202004, No. XHZDSYS202005) and Xiamen municipal Bureau of Science and Technology Grant(3502Z20174079).

## Conflict of Interest

The authors declare that the research was conducted in the absence of any commercial or financial relationships that could be construed as a potential conflict of interest.

## Publisher’s Note

All claims expressed in this article are solely those of the authors and do not necessarily represent those of their affiliated organizations, or those of the publisher, the editors and the reviewers. Any product that may be evaluated in this article, or claim that may be made by its manufacturer, is not guaranteed or endorsed by the publisher.
