# Supplementary material for: Global Burden, Incidence and Disability-Adjusted Life-Years for Dermatitis: A Systematic Analysis Combined With Socioeconomic Development Status, 1990–2019
Source: Front Cell Infect Microbiol. 2022 Apr 12;12:861053. doi: 10.3389/fcimb.2022.861053 (PMC9039287; doi:10.3389/fcimb.2022.861053)
Supplement: Supplementary file 1 [file Table_1.docx]

# Global Burden, Incidence and Disability-Adjusted Life-Years for dermatitis：A systematic analysis combined with socioeconomic development status, 1990 -2019

**Table of Contents**

**S1 Table. Change in(incidence and DALYs) dermatitis, 1990-2019, Page 1**

**S2 Table. Change in Incidence rate, 1990-2019, Page 2-3
S3 Table. Change in DALYs rate, 1990-2019, Page 4-5
S4 Table. Incidence of dermatitis, 1990-2019, Page 6-8
S5 Table. DALYs of dermatitis, 1990-2019, Page 9-11
S6 Table. Incidence of dermatitis in 2019, Page 12-16**

**S7 Table. DALYs of dermatitis in 2019, Page 17-21**

**S8 Table. Incidence of AD stratified by sex in 1990~2019, Page 22-24**

**S9 Table. Incidence of AD stratified by SDI in 1990~2019,Global, Page 25-26**

**S10 Table. DALYs of AD stratified by sex in 1990~2019, Page 27-37**

**S11 Table. DALYs of AD stratified by SDI in 1990~2019, Page 38-48**

**S12 Table. Incidence of AD in 2019, Page 49-53
S13 Table. DALYs of AD in 2019, Page 54-58
S14 Table. Incidence of CD stratified by sex in 1990~2019, Page 59-61**

**S15 Table. Incidence of CD stratified by SDI in 1990~2019, Page 62-72**

**S16 Table. DALYs of CD stratified by sex in 1990~2019, Page 73-75**

**S17 Table. DALYs of CD stratified by SDI in 1990~2019, Page 76-86**

**S18 Table. Incidence of CD in 2019, Page 87-91
S19 Table. DALYs of CD in 2019, Page 92-96**

**S20 Table. Incidence of SD stratified by sex in 1990~2019, Page 97-99**

**S21 Table. Incidence of SD stratified by SDI in 1990~2019, Page 100-110**

**S22 Table. DALYs of SD stratified by sex in 1990~2019, Page 111-113**

**S23 Table. DALYs of SD by SDI in 1990~2019, Page 114-124**

**S24 Table. Incidence of SD in 2019, Page 125-129**

**S25 Table. DALYs of SD in 2019, Page 130-134**

**S26 Table. The age-standardized incidence rate of major dermatitis and DALYs combined with different factors (global, different SDI, gender) summarized results in 2019 (or 1990-2019), Page 135-138**

**S27 Table. SDI values for all estimated GBD locations in 2019(marked countries with the highest age-standardized incidence and DALYs , 1990-2019, Page 139-172**

**Table1 Change in(incidence and DALYs) dermatitis, 1990-2019, Page 1**

| **Measure** | **location** | **Sex** | **Change in Incidence/DALYs rate ( per 100 000 person-years)** | **95% CI (lower)** | **95% CI (upper)** |
| --- | --- | --- | --- | --- | --- |
| Incidence | Global | Male | 0.0142 | 0.0108 | 0.0175 |
| Incidence | Global | Female | 0.0012 | -0.0029 | 0.0056 |
| Incidence | Global | Both | 0.0077 | 0.0045 | 0.0111 |
| Incidence | High SDI | Both | -0.0356 | -0.0412 | -0.0300 |
| Incidence | High-middle SDI | Both | 0.0183 | 0.0143 | 0.0224 |
| Incidence | Middle SDI | Both | -0.0398 | -0.0426 | -0.0370 |
| Incidence | Low-middle SDI | Both | -0.0087 | -0.0105 | -0.0069 |
| Incidence | Low SDI | Both | -0.0464 | -0.0476 | -0.0451 |
| DALYs | Global | Male | -0.0205 | -0.0286 | -0.0124 |
| DALYs | Global | Female | -0.0393 | -0.0472 | -0.0314 |
| DALYs | Global | Both | -0.0311 | -0.0378 | -0.0249 |
| DALYs | High SDI | Both | -0.0417 | -0.0500 | -0.0344 |
| DALYs | High-middle SDI | Both | 0.0396 | 0.0301 | 0.0489 |
| DALYs | Middle SDI | Both | -0.0126 | -0.0209 | -0.0046 |
| DALYs | Low-middle SDI | Both | -0.0150 | -0.0249 | -0.0064 |
| DALYs | Low SDI | Both | -0.0093 | -0.0197 | 0.0011 |
|  |  |  |  |  |  |

**Table2 Change in Incidence rate, 1990-2019,Page 2-3**

| **Cause** | **Location** | **Sex** | **Change in Incidence Rate (per 100 000 person-years)** | **95% CI (lower)** | **95% CI (upper)** |
| --- | --- | --- | --- | --- | --- |
| AD | Global | Male | -0.0207 | -0.0283 | -0.0127 |
| AD | Global | Female | -0.0562 | -0.0643 | -0.0477 |
| AD | Global | Both | -0.0420 | -0.0481 | -0.0358 |
| CD | Global | Male | 0.0103 | 0.0048 | 0.0151 |
| CD | Global | Female | -0.0020 | -0.0084 | 0.0047 |
| CD | Global | Both | 0.0042 | -0.0007 | 0.0094 |
| SD | Global | Male | 0.0254 | 0.0216 | 0.0290 |
| SD | Global | Female | 0.0202 | 0.0162 | 0.0245 |
| SD | Global | Both | 0.0230 | 0.0197 | 0.0261 |
| AD | High SDI | Male | -0.0376 | -0.0508 | -0.0237 |
| AD | High SDI | Female | -0.0313 | -0.0411 | -0.0207 |
| AD | High SDI | Both | -0.0359 | -0.0442 | -0.0277 |
| CD | High SDI | Male | -0.0415 | -0.0522 | -0.0306 |
| CD | High SDI | Female | -0.0460 | -0.0569 | -0.0348 |
| CD | High SDI | Both | -0.0457 | -0.0549 | -0.0361 |
| SD | High SDI | Male | -0.0157 | -0.0206 | -0.0112 |
| SD | High SDI | Female | -0.0260 | -0.0305 | -0.0216 |
| SD | High SDI | Both | -0.0223 | -0.0261 | -0.0187 |
| AD | High-middle SDI | Male | 0.0464 | 0.0338 | 0.0604 |
| AD | High-middle SDI | Female | 0.0517 | 0.0391 | 0.0640 |
| AD | High-middle SDI | Both | 0.0459 | 0.0367 | 0.0556 |
| CD | High-middle SDI | Male | 0.0359 | 0.0281 | 0.0432 |
| CD | High-middle SDI | Female | -0.0005 | -0.0082 | 0.0080 |
| CD | High-middle SDI | Both | 0.0133 | 0.0068 | 0.0198 |
| SD | High-middle SDI | Male | 0.0181 | 0.0151 | 0.0209 |
| SD | High-middle SDI | Female | 0.0246 | 0.0216 | 0.0277 |
| SD | High-middle SDI | Both | 0.0217 | 0.0194 | 0.0240 |
| AD | Middle SDI | Male | -0.0847 | -0.0974 | -0.0713 |
| AD | Middle SDI | Female | -0.0502 | -0.0637 | -0.0371 |
| AD | Middle SDI | Both | -0.0622 | -0.0717 | -0.0517 |
| CD | Middle SDI | Male | -0.0874 | -0.0935 | -0.0794 |
| CD | Middle SDI | Female | -0.0084 | -0.0130 | -0.0043 |
| CD | Middle SDI | Both | -0.0433 | -0.0469 | -0.0395 |
| SD | Middle SDI | Male | -0.0589 | -0.0634 | -0.0542 |
| SD | Middle SDI | Female | 0.0046 | 0.0009 | 0.0087 |
| SD | Middle SDI | Both | -0.0282 | -0.0315 | -0.0249 |
| AD | Low-middle SDI | Male | -0.0473 | -0.0517 | -0.0430 |
| AD | Low-middle SDI | Female | -0.0494 | -0.0546 | -0.0441 |
| AD | Low-middle SDI | Both | -0.0471 | -0.0506 | -0.0434 |
| CD | Low-middle SDI | Male | -0.0249 | -0.0274 | -0.0221 |
| CD | Low-middle SDI | Female | -0.0061 | -0.0086 | -0.0033 |
| CD | Low-middle SDI | Both | -0.0127 | -0.0149 | -0.0104 |
| SD | Low-middle SDI | Male | -0.0146 | -0.0169 | -0.0121 |
| SD | Low-middle SDI | Female | 0.0242 | 0.0211 | 0.0272 |
| SD | Low-middle SDI | Both | 0.0048 | 0.0026 | 0.0069 |
| SD | Low SDI | Male | -0.0934 | -0.0953 | -0.0915 |
| SD | Low SDI | Female | 0.0044 | 0.0030 | 0.0057 |
| SD | Low SDI | Both | -0.0442 | -0.0456 | -0.0428 |
| AD | Low SDI | Male | -0.1558 | -0.1614 | -0.1499 |
| AD | Low SDI | Female | -0.0246 | -0.0302 | -0.0185 |
| AD | Low SDI | Both | -0.0853 | -0.0900 | -0.0804 |
| CD | Low SDI | Male | -0.0988 | -0.1013 | -0.0962 |
| CD | Low SDI | Female | -0.0014 | -0.0034 | 0.0007 |
| CD | Low SDI | Both | -0.0453 | -0.0471 | -0.0432 |
|  |  |  |  |  |  |

**Table3 Change in DALYs rate, 1990-2019, Page 4-5**

| **Cause** | **Location** | **Sex** | **Change in DALYs Rate (per 100 000 person-years)** | **95% CI (lower)** | **95% CI (upper)** |
| --- | --- | --- | --- | --- | --- |
| AD | Global | Male | -0.0305 | -0.0395 | -0.0215 |
| AD | Global | Female | -0.0491 | -0.0578 | -0.0404 |
| AD | Global | Both | -0.0414 | -0.0475 | -0.0350 |
| CD | Global | Male | 0.0058 | -0.0049 | 0.0164 |
| CD | Global | Female | -0.0076 | -0.0181 | 0.0024 |
| CD | Global | Both | -0.0010 | -0.0094 | 0.0076 |
| SD | Global | Male | 0.0241 | 0.0133 | 0.0347 |
| SD | Global | Female | 0.0266 | 0.0150 | 0.0380 |
| SD | Global | Both | 0.0270 | 0.0185 | 0.0354 |
| AD | High SDI | Male | -0.0390 | -0.0531 | -0.0255 |
| AD | High SDI | Female | -0.0349 | -0.0460 | -0.0229 |
| AD | High SDI | Both | -0.0384 | -0.0474 | -0.0295 |
| CD | High SDI | Male | -0.0603 | -0.0800 | -0.0397 |
| CD | High SDI | Female | -0.0652 | -0.0861 | -0.0445 |
| CD | High SDI | Both | -0.0645 | -0.0802 | -0.0479 |
| SD | High SDI | Male | -0.0227 | -0.0419 | -0.0033 |
| SD | High SDI | Female | -0.0361 | -0.0544 | -0.0172 |
| SD | High SDI | Both | -0.0267 | -0.0405 | -0.0135 |
| AD | High-middle SDI | Male | 0.0392 | 0.0230 | 0.0555 |
| AD | High-middle SDI | Female | 0.0526 | 0.0378 | 0.0675 |
| AD | High-middle SDI | Both | 0.0439 | 0.0326 | 0.0556 |
| CD | High-middle SDI | Male | 0.0500 | 0.0280 | 0.0718 |
| CD | High-middle SDI | Female | 0.0027 | -0.0155 | 0.0199 |
| CD | High-middle SDI | Both | 0.0214 | 0.0066 | 0.0358 |
| SD | High-middle SDI | Male | 0.0380 | 0.0105 | 0.0652 |
| SD | High-middle SDI | Female | 0.0407 | 0.0142 | 0.0673 |
| SD | High-middle SDI | Both | 0.0421 | 0.0245 | 0.0621 |
| AD | Middle SDI | Male | -0.0008 | -0.0177 | 0.0159 |
| AD | Middle SDI | Female | -0.0282 | -0.0427 | -0.0144 |
| AD | Middle SDI | Both | -0.0157 | -0.0274 | -0.0055 |
| CD | Middle SDI | Male | -0.0081 | -0.0220 | 0.0063 |
| CD | Middle SDI | Female | -0.0057 | -0.0193 | 0.0085 |
| CD | Middle SDI | Both | -0.0060 | -0.0164 | 0.0040 |
| SD | Middle SDI | Male | 0.0225 | 0.0025 | 0.0428 |
| SD | Middle SDI | Female | 0.0130 | -0.0063 | 0.0317 |
| SD | Middle SDI | Both | 0.0190 | 0.0048 | 0.0322 |
| AD | Low-middle SDI | Male | -0.0159 | -0.0323 | 0.0012 |
| AD | Low-middle SDI | Female | -0.0299 | -0.0449 | -0.0153 |
| AD | Low-middle SDI | Both | -0.0219 | -0.0338 | -0.0103 |
| CD | Low-middle SDI | Male | -0.0025 | -0.0201 | 0.0159 |
| CD | Low-middle SDI | Female | -0.0013 | -0.0175 | 0.0158 |
| CD | Low-middle SDI | Both | 0.0000 | -0.0124 | 0.0128 |
| SD | Low-middle SDI | Male | 0.0270 | 0.0021 | 0.0510 |
| SD | Low-middle SDI | Female | 0.0308 | 0.0064 | 0.0578 |
| SD | Low-middle SDI | Both | 0.0283 | 0.0113 | 0.0456 |
| AD | Low SDI | Male | -0.0242 | -0.0440 | -0.0063 |
| AD | Low SDI | Female | -0.0109 | -0.0272 | 0.0072 |
| AD | Low SDI | Both | -0.0166 | -0.0302 | -0.0029 |
| CD | Low SDI | Male | 0.0015 | -0.0182 | 0.0216 |
| CD | Low SDI | Female | 0.0037 | -0.0159 | 0.0232 |
| CD | Low SDI | Both | 0.0036 | -0.0116 | 0.0168 |
| SD | Low SDI | Male | 0.0181 | -0.0042 | 0.0390 |
| SD | Low SDI | Female | 0.0072 | -0.0146 | 0.0306 |
| SD | Low SDI | Both | 0.0124 | -0.0035 | 0.0280 |
|  |  |  |  |  |  |

**Table4 Incidence of dermatitis, 1990-2019, Page 6-8**

| **cause_name** | **sex_name** | **year** | **Age-standardised incidence rate（per 100 000 person-years）** | **95%CI(lower)** | **95%CI(upper)** |
| --- | --- | --- | --- | --- | --- |
| AD | Both | 1990 | 342.3050 | 327.0355 | 358.4175 |
| CD | Both | 1990 | 3053.2513 | 2401.4428 | 3741.1057 |
| SD | Both | 1990 | 1808.8728 | 1668.9329 | 1945.8972 |
| Dermatitis | Both | 1990 | 5204.4291 | 4517.0914 | 5949.2963 |
| AD | Both | 1992 | 341.8515 | 326.5309 | 357.9311 |
| CD | Both | 1992 | 3029.6938 | 3029.6938 | 3029.6938 |
| SD | Both | 1992 | 1808.2822 | 1668.7724 | 1945.0516 |
| Dermatitis | Both | 1993 | 5171.1421 | 4490.9478 | 5907.2631 |
| Dermatitis | Both | 1992 | 5179.8275 | 4496.8242 | 5917.6715 |
| Dermatitis | Both | 1991 | 5191.4079 | 4506.3134 | 5931.9825 |
| AD | Both | 1994 | 341.3385 | 325.9302 | 357.3515 |
| CD | Both | 1994 | 3016.6312 | 2375.0448 | 3688.9839 |
| SD | Both | 1994 | 1808.6077 | 1669.2657 | 1945.4492 |
| AD | Both | 1991 | 342.0828 | 326.7936 | 358.2657 |
| CD | Both | 1991 | 3040.7683 | 2392.4908 | 3724.1324 |
| SD | Both | 1991 | 1808.5568 | 1668.8618 | 1945.4266 |
| Dermatitis | Both | 1995 | 5167.2371 | 4489.2483 | 5902.4397 |
| AD | Both | 1993 | 341.6102 | 326.2494 | 357.4832 |
| CD | Both | 1993 | 3021.3173 | 2378.3815 | 3696.4403 |
| SD | Both | 1993 | 1808.2147 | 1668.8382 | 1944.9841 |
| Dermatitis | Both | 1994 | 5166.5773 | 4488.0703 | 5901.7273 |
| AD | Both | 1996 | 340.8303 | 325.5656 | 356.8282 |
| CD | Both | 1996 | 3019.4934 | 2377.6037 | 3691.5227 |
| SD | Both | 1996 | 1810.7219 | 1671.0994 | 1948.0154 |
| Dermatitis | Both | 1996 | 5171.0456 | 4492.6456 | 5906.3429 |
| AD | Both | 1995 | 341.0297 | 325.7256 | 357.0027 |
| CD | Both | 1995 | 3016.6461 | 2375.4466 | 3687.8294 |
| SD | Both | 1995 | 1809.5613 | 1670.1317 | 1946.5765 |
| Dermatitis | Both | 1997 | 5174.7858 | 4495.8827 | 5910.5159 |
| AD | Both | 1997 | 340.8256 | 325.5982 | 356.8368 |
| CD | Both | 1997 | 3022.1483 | 2379.6155 | 3695.3822 |
| SD | Both | 1997 | 1811.8119 | 1672.0203 | 1949.3812 |
| AD | Both | 1998 | 340.9495 | 325.7181 | 356.94 |
| CD | Both | 1998 | 3024.789 | 2381.7751 | 3699.5477 |
| SD | Both | 1998 | 1812.8099 | 1672.7567 | 1950.6278 |
| Dermatitis | Both | 1998 | 5178.5484 | 4499.0581 | 5914.5957 |
| Dermatitis | Both | 1999 | 5182.3486 | 4502.1731 | 5918.4694 |
| AD | Both | 1999 | 341.0569 | 325.8156 | 357.0257 |
| CD | Both | 1999 | 3027.4407 | 2383.9948 | 3703.6518 |
| SD | Both | 1999 | 1813.8509 | 1673.49 | 1951.8996 |
| AD | Both | 2000 | 340.9791 | 325.7384 | 356.9292 |
| CD | Both | 2000 | 3029.7717 | 2385.6847 | 3707.1497 |
| SD | Both | 2000 | 1815.0524 | 1674.4238 | 1953.3341 |
| Dermatitis | Both | 2000 | 5185.8031 | 4505.0336 | 5921.988 |
| AD | Both | 2001 | 340.7385 | 325.4575 | 356.6937 |
| CD | Both | 2001 | 3032.1113 | 2387.1889 | 3710.263 |
| SD | Both | 2001 | 1816.4112 | 1675.6118 | 1954.9506 |
| Dermatitis | Both | 2001 | 5189.261 | 4507.9314 | 5925.475 |
| AD | Both | 2002 | 340.3747 | 325.0463 | 356.3574 |
| CD | Both | 2002 | 3034.1943 | 2388.323 | 3713.1585 |
| SD | Both | 2002 | 1817.8748 | 1677.0003 | 1956.6687 |
| Dermatitis | Both | 2002 | 5192.4438 | 4510.728 | 5928.9529 |
| AD | Both | 2004 | 339.4394 | 324.2848 | 355.4934 |
| CD | Both | 2004 | 3038.1394 | 2390.7594 | 3717.9992 |
| SD | Both | 2004 | 1821.0417 | 1680.1142 | 1960.3272 |
| Dermatitis | Both | 2003 | 5195.5679 | 4513.5375 | 5932.3954 |
| Dermatitis | Both | 2007 | 5207.5909 | 4523.0036 | 5944.0756 |
| AD | Both | 2003 | 339.9279 | 324.6264 | 356.0212 |
| CD | Both | 2003 | 3036.2404 | 2389.4643 | 3715.7878 |
| SD | Both | 2003 | 1819.3996 | 1678.5144 | 1958.4371 |
| Dermatitis | Both | 2004 | 5198.6206 | 4516.3321 | 5935.7329 |
| Dermatitis | Both | 2005 | 5201.5009 | 4518.3811 | 5938.88 |
| AD | Both | 2006 | 338.3032 | 323.1693 | 354.4342 |
| CD | Both | 2006 | 3041.7112 | 2392.5883 | 3721.8244 |
| SD | Both | 2006 | 1824.6184 | 1683.0604 | 1964.2785 |
| Dermatitis | Both | 2006 | 5204.6328 | 4520.7348 | 5940.9678 |
| AD | Both | 2008 | 337.0254 | 321.816 | 352.938 |
| CD | Both | 2008 | 3045.1923 | 2394.2071 | 3727.5129 |
| SD | Both | 2008 | 1828.2693 | 1685.9541 | 1968.2269 |
| AD | Both | 2005 | 338.8859 | 323.7432 | 354.8851 |
| CD | Both | 2005 | 3039.8141 | 2391.7712 | 3719.8787 |
| SD | Both | 2005 | 1822.8009 | 1681.5714 | 1962.288 |
| AD | Both | 2007 | 337.6854 | 322.4273 | 353.6775 |
| CD | Both | 2007 | 3043.4927 | 2393.5569 | 3724.7296 |
| SD | Both | 2007 | 1826.4128 | 1684.5064 | 1966.2292 |
| Dermatitis | Both | 2008 | 5210.487 | 4525.1849 | 5947.778 |
| SD | Both | 2009 | 1830.2198 | 1687.4584 | 1970.3109 |
| AD | Both | 2009 | 336.3642 | 321.102 | 352.5601 |
| CD | Both | 2009 | 3046.8926 | 2394.5223 | 3730.0091 |
| Dermatitis | Both | 2009 | 5213.4766 | 4527.3973 | 5950.5227 |
| AD | Both | 2010 | 335.7984 | 320.3534 | 352.0703 |
| CD | Both | 2010 | 3048.6551 | 2395.0663 | 3732.8318 |
| SD | Both | 2010 | 1832.1898 | 1688.9756 | 1972.4573 |
| Dermatitis | Both | 2010 | 5216.6433 | 4529.8006 | 5953.5511 |
| AD | Both | 2011 | 335.1685 | 319.8666 | 351.4238 |
| CD | Both | 2011 | 3050.7401 | 2395.7477 | 3735.8672 |
| SD | Both | 2011 | 1834.1463 | 1690.7794 | 1974.6079 |
| Dermatitis | Both | 2011 | 5220.0548 | 4532.225 | 5956.9267 |
| Dermatitis | Both | 2012 | 5223.0962 | 4534.3061 | 5959.4096 |
| AD | Both | 2012 | 334.3158 | 319.1697 | 350.4963 |
| CD | Both | 2012 | 3052.7088 | 2396.4095 | 3738.5064 |
| SD | Both | 2012 | 1836.0716 | 1692.6354 | 1976.7609 |
| AD | Both | 2013 | 333.3268 | 318.3105 | 349.3894 |
| CD | Both | 2013 | 3054.7446 | 2397.2306 | 3741.2038 |
| SD | Both | 2013 | 1837.9967 | 1694.4654 | 1978.9385 |
| Dermatitis | Both | 2013 | 5226.0682 | 4536.337 | 5961.6528 |
| SD | Both | 2014 | 1839.9654 | 1696.3236 | 1981.1958 |
| AD | Both | 2014 | 332.3125 | 317.1769 | 348.2579 |
| CD | Both | 2014 | 3056.7238 | 2398.1493 | 3743.8446 |
| Dermatitis | Both | 2015 | 5231.8768 | 4540.2344 | 5967.0122 |
| AD | Both | 2016 | 330.4757 | 315.2305 | 346.256 |
| CD | Both | 2016 | 3060.5074 | 2400.9523 | 3748.668 |
| SD | Both | 2016 | 1844.0262 | 1700.1466 | 1985.9738 |
| Dermatitis | Both | 2014 | 5229.0016 | 4538.3303 | 5964.0652 |
| Dermatitis | Both | 2016 | 5235.0093 | 4542.6022 | 5970.1557 |
| AD | Both | 2015 | 331.3748 | 316.1018 | 347.199 |
| CD | Both | 2015 | 3058.5307 | 2399.2855 | 3746.0797 |
| SD | Both | 2015 | 1841.9713 | 1698.2059 | 1983.5342 |
| Dermatitis | Both | 2017 | 5237.9762 | 4545.301 | 5972.9506 |
| AD | Both | 2017 | 329.4468 | 314.2388 | 345.1537 |
| CD | Both | 2017 | 3062.4013 | 2402.3791 | 3750.8906 |
| SD | Both | 2017 | 1846.128 | 1702.1388 | 1988.5011 |
| Dermatitis | Both | 2018 | 5241.094 | 4548.3928 | 5976.0405 |
| AD | Both | 2018 | 328.5466 | 313.2764 | 344.2054 |
| CD | Both | 2018 | 3064.279 | 2403.8359 | 3753.1442 |
| SD | Both | 2018 | 1848.2684 | 1704.1809 | 1991.0989 |
| Dermatitis | Both | 2019 | 5244.3988 | 4551.7244 | 5979.3176 |
| AD | Both | 2019 | 327.9136 | 312.7599 | 343.6677 |
| CD | Both | 2019 | 3066.0421 | 2405.3768 | 3755.3806 |
| SD | Both | 2019 | 1850.4431 | 1706.2477 | 1993.7403 |

**Table5 DALYs of dermatitis, 1990-2019, Page 9-11**

| **cause_name** | **sex_name** | **year** | **Age-standardised DAYLs rate（per 100 000 person-years）** | **95%CI(lower)** | **95%CI(upper)** |
| --- | --- | --- | --- | --- | --- |
| Dermatitis | Both | 1990 | 135.9009 | 79.9488 | 214.0275 |
| AD | Both | 1990 | 103.9911 | 55.3169 | 174.5661 |
| CD | Both | 1990 | 28.0846 | 17.6198 | 41.6829 |
| SD | Both | 1990 | 3.8252 | 2.1781 | 6.0547 |
| AD | Both | 1991 | 103.8902 | 55.3838 | 174.6280 |
| CD | Both | 1991 | 27.9388 | 17.5429 | 41.5677 |
| SD | Both | 1991 | 3.8210 | 2.1790 | 6.0662 |
| Dermatitis | Both | 1992 | 135.4310 | 79.7096 | 213.9196 |
| AD | Both | 1992 | 103.8033 | 55.2332 | 174.6960 |
| CD | Both | 1992 | 27.8096 | 17.4737 | 41.3084 |
| SD | Both | 1992 | 3.8181 | 2.1762 | 6.0501 |
| Dermatitis | Both | 1991 | 135.6499 | 79.7119 | 213.7519 |
| AD | Both | 1993 | 103.6928 | 55.4024 | 174.3778 |
| CD | Both | 1993 | 27.7081 | 17.3996 | 41.1430 |
| SD | Both | 1993 | 3.8163 | 2.1774 | 6.0658 |
| Dermatitis | Both | 1993 | 135.2173 | 79.5412 | 213.6991 |
| Dermatitis | Both | 1994 | 135.0295 | 79.4420 | 213.7596 |
| AD | Both | 1994 | 103.5646 | 55.2742 | 174.2763 |
| CD | Both | 1994 | 27.6482 | 17.3438 | 41.0534 |
| SD | Both | 1994 | 3.8168 | 2.1778 | 6.0447 |
| Dermatitis | Both | 1995 | 134.8693 | 79.3031 | 213.2935 |
| AD | Both | 1995 | 103.4079 | 55.2827 | 174.2878 |
| CD | Both | 1995 | 27.6426 | 17.3748 | 41.1272 |
| SD | Both | 1995 | 3.8189 | 2.1757 | 6.0672 |
| AD | Both | 1996 | 103.2676 | 55.2193 | 173.8933 |
| CD | Both | 1996 | 27.6674 | 17.3884 | 41.1150 |
| SD | Both | 1996 | 3.8226 | 2.1801 | 6.0509 |
| Dermatitis | Both | 1996 | 134.7576 | 79.3586 | 213.1300 |
| Dermatitis | Both | 1997 | 134.7065 | 79.2609 | 213.1434 |
| AD | Both | 1997 | 103.1895 | 55.1626 | 173.2932 |
| CD | Both | 1997 | 27.6911 | 17.3915 | 41.1055 |
| SD | Both | 1997 | 3.8259 | 2.1731 | 6.0663 |
| Dermatitis | Both | 1998 | 134.6783 | 79.2245 | 213.1161 |
| Dermatitis | Both | 1999 | 134.6543 | 79.2620 | 213.2958 |
| AD | Both | 1998 | 103.1334 | 55.1354 | 173.7863 |
| CD | Both | 1998 | 27.7153 | 17.4204 | 41.2779 |
| SD | Both | 1998 | 3.8297 | 2.1816 | 6.0710 |
| Dermatitis | Both | 2000 | 134.5794 | 79.2329 | 213.0597 |
| AD | Both | 1999 | 103.0832 | 55.1311 | 173.5122 |
| CD | Both | 1999 | 27.7383 | 17.4162 | 41.2555 |
| SD | Both | 1999 | 3.8328 | 2.1840 | 6.0773 |
| AD | Both | 2000 | 102.9841 | 55.0087 | 173.4829 |
| CD | Both | 2000 | 27.7584 | 17.4740 | 41.2582 |
| SD | Both | 2000 | 3.8370 | 2.1858 | 6.0902 |
| AD | Both | 2001 | 102.8470 | 54.9619 | 173.2033 |
| CD | Both | 2001 | 27.7776 | 17.4895 | 41.3028 |
| Dermatitis | Both | 2001 | 134.4664 | 79.2812 | 212.9288 |
| SD | Both | 2002 | 3.8454 | 2.1891 | 6.0937 |
| AD | Both | 2002 | 102.6828 | 54.9837 | 173.0625 |
| CD | Both | 2002 | 27.7953 | 17.5067 | 41.3179 |
| Dermatitis | Both | 2002 | 134.3235 | 79.0972 | 212.7725 |
| SD | Both | 2001 | 3.8418 | 2.1975 | 6.0856 |
| Dermatitis | Both | 2003 | 134.1769 | 79.1130 | 212.1925 |
| AD | Both | 2004 | 102.3491 | 54.6588 | 172.0290 |
| CD | Both | 2004 | 27.8302 | 17.4795 | 41.3119 |
| SD | Both | 2004 | 3.8562 | 2.1959 | 6.1205 |
| AD | Both | 2003 | 102.5111 | 54.8391 | 172.6206 |
| CD | Both | 2003 | 27.8152 | 17.4947 | 41.3427 |
| SD | Both | 2003 | 3.8505 | 2.1949 | 6.0890 |
| Dermatitis | Both | 2004 | 134.0355 | 78.8884 | 211.9232 |
| Dermatitis | Both | 2006 | 133.7401 | 78.8022 | 211.2211 |
| Dermatitis | Both | 2005 | 133.8785 | 78.7876 | 211.7347 |
| AD | Both | 2007 | 101.8576 | 54.4607 | 171.3572 |
| CD | Both | 2007 | 27.8820 | 17.4972 | 41.3758 |
| SD | Both | 2007 | 3.8723 | 2.2150 | 6.1321 |
| AD | Both | 2006 | 102.0096 | 54.5755 | 171.5930 |
| CD | Both | 2006 | 27.8633 | 17.5238 | 41.4692 |
| SD | Both | 2006 | 3.8672 | 2.2131 | 6.0903 |
| AD | Both | 2005 | 102.1726 | 54.5912 | 171.6622 |
| CD | Both | 2005 | 27.8436 | 17.4548 | 41.4132 |
| SD | Both | 2005 | 3.8623 | 2.2064 | 6.1375 |
| Dermatitis | Both | 2007 | 133.6119 | 78.7427 | 211.2324 |
| AD | Both | 2008 | 101.7114 | 54.4610 | 171.5495 |
| CD | Both | 2008 | 27.9016 | 17.5650 | 41.4047 |
| SD | Both | 2008 | 3.8785 | 2.2121 | 6.1635 |
| Dermatitis | Both | 2008 | 133.4915 | 78.6897 | 210.8369 |
| Dermatitis | Both | 2009 | 133.3939 | 78.7682 | 210.4800 |
| AD | Both | 2009 | 101.5894 | 54.4494 | 171.0920 |
| CD | Both | 2009 | 27.9205 | 17.5627 | 41.5213 |
| SD | Both | 2009 | 3.8841 | 2.2149 | 6.1614 |
| Dermatitis | Both | 2012 | 132.9531 | 78.2359 | 209.5792 |
| Dermatitis | Both | 2010 | 133.2987 | 78.5643 | 209.9017 |
| AD | Both | 2012 | 101.0868 | 53.9647 | 170.2401 |
| CD | Both | 2012 | 27.9678 | 17.5764 | 41.6363 |
| SD | Both | 2012 | 3.8985 | 2.2264 | 6.1995 |
| AD | Both | 2010 | 101.4755 | 54.3997 | 171.1972 |
| CD | Both | 2010 | 27.9341 | 17.5944 | 41.4840 |
| SD | Both | 2010 | 3.8892 | 2.2208 | 6.1566 |
| Dermatitis | Both | 2011 | 133.1614 | 78.6365 | 209.8144 |
| AD | Both | 2014 | 100.5751 | 53.6629 | 169.2160 |
| CD | Both | 2014 | 27.9993 | 17.5858 | 41.6784 |
| SD | Both | 2014 | 3.9076 | 2.2263 | 6.2138 |
| AD | Both | 2013 | 100.8296 | 53.7727 | 169.4363 |
| CD | Both | 2013 | 27.9830 | 17.6161 | 41.6532 |
| SD | Both | 2013 | 3.9032 | 2.2254 | 6.2058 |
| AD | Both | 2011 | 101.3191 | 54.0525 | 170.8260 |
| CD | Both | 2011 | 27.9484 | 17.5530 | 41.5110 |
| SD | Both | 2011 | 3.8939 | 2.2202 | 6.1744 |
| Dermatitis | Both | 2013 | 132.7157 | 78.2398 | 209.1059 |
| Dermatitis | Both | 2014 | 132.4819 | 78.2019 | 208.0788 |
| Dermatitis | Both | 2015 | 132.3020 | 77.8961 | 207.7889 |
| AD | Both | 2017 | 99.9874 | 53.2943 | 167.6512 |
| CD | Both | 2017 | 28.0366 | 17.5983 | 41.6581 |
| SD | Both | 2017 | 3.9203 | 2.2317 | 6.2205 |
| Dermatitis | Both | 2016 | 132.1417 | 77.9551 | 207.1304 |
| AD | Both | 2016 | 100.2028 | 53.4566 | 168.4888 |
| CD | Both | 2016 | 28.0225 | 17.5868 | 41.7422 |
| SD | Both | 2016 | 3.9164 | 2.2325 | 6.2160 |
| AD | Both | 2015 | 100.3793 | 53.6008 | 168.7494 |
| CD | Both | 2015 | 28.0108 | 17.6032 | 41.5691 |
| SD | Both | 2015 | 3.9118 | 2.2245 | 6.2341 |
| AD | Both | 2018 | 99.8022 | 53.0738 | 167.6737 |
| CD | Both | 2018 | 28.0444 | 17.5843 | 41.7567 |
| SD | Both | 2018 | 3.9237 | 2.2296 | 6.2285 |
| AD | Both | 2019 | 99.6852 | 53.0912 | 167.4329 |
| CD | Both | 2019 | 28.0575 | 17.6212 | 41.7765 |
| SD | Both | 2019 | 3.9284 | 2.2410 | 6.2459 |
| Dermatitis | Both | 2017 | 131.9443 | 77.8904 | 206.8349 |
| Dermatitis | Both | 2018 | 131.7702 | 77.7905 | 206.6640 |
| Dermatitis | Both | 2019 | 131.6711 | 77.5876 | 206.8696 |
|  |  |  |  |  |  |

**Table6 Incidence of dermatitis in 2019, Page 12-16**

| **location_name** | **sex_name** | **year** | **Age-standardised incidence rate (per 100 000 person-years)** | **95% CI(lower)** | **95% CI(upper)** |
| --- | --- | --- | --- | --- | --- |
| Sri Lanka | Both | 2019 | 5693.8383 | 4901.4604 | 6561.8100 |
| Tonga | Both | 2019 | 5310.3095 | 4593.3576 | 6044.9228 |
| Uzbekistan | Both | 2019 | 5305.3345 | 4515.4417 | 6124.5705 |
| Thailand | Both | 2019 | 5548.8668 | 4795.9092 | 6360.0574 |
| China | Both | 2019 | 5714.8606 | 4919.2091 | 6571.8882 |
| Brunei Darussalam | Both | 2019 | 3291.6975 | 3023.7000 | 3566.1366 |
| Republic of Korea | Both | 2019 | 3300.2689 | 3020.5588 | 3572.3631 |
| France | Both | 2019 | 4014.0049 | 3625.1520 | 4444.8683 |
| Singapore | Both | 2019 | 3271.6479 | 2997.8065 | 3544.8386 |
| Germany | Both | 2019 | 3776.3597 | 3428.0752 | 4141.8511 |
| Vanuatu | Both | 2019 | 5296.9393 | 4579.9501 | 6031.1196 |
| Spain | Both | 2019 | 3245.1174 | 2993.0821 | 3501.2389 |
| Tajikistan | Both | 2019 | 5290.3867 | 4505.4499 | 6107.7159 |
| Burundi | Both | 2019 | 5234.0643 | 4605.8382 | 5881.6085 |
| Latvia | Both | 2019 | 4567.6010 | 3842.8145 | 5290.2780 |
| Japan | Both | 2019 | 3586.4386 | 3298.8998 | 3895.2985 |
| Taiwan (Province of China) | Both | 2019 | 5345.0712 | 4630.0278 | 6080.6680 |
| Democratic People's Republic of Korea | Both | 2019 | 5278.5015 | 4561.0547 | 6006.2609 |
| Turkmenistan | Both | 2019 | 5265.4307 | 4484.0084 | 6077.1955 |
| Montenegro | Both | 2019 | 4909.6192 | 4125.7409 | 5726.4561 |
| Greece | Both | 2019 | 3210.3469 | 2946.5439 | 3466.8966 |
| Lithuania | Both | 2019 | 4631.1167 | 3911.0519 | 5354.3189 |
| New Zealand | Both | 2019 | 3458.9595 | 3175.9749 | 3767.9630 |
| Somalia | Both | 2019 | 5245.8564 | 4611.1935 | 5896.8036 |
| Comoros | Both | 2019 | 5241.6098 | 4609.3164 | 5893.4059 |
| Armenia | Both | 2019 | 5315.2664 | 4521.2947 | 6140.6881 |
| Benin | Both | 2019 | 5444.5642 | 4799.6373 | 6124.5064 |
| Kyrgyzstan | Both | 2019 | 5311.4672 | 4520.2460 | 6132.2712 |
| Switzerland | Both | 2019 | 3265.8951 | 3022.4180 | 3524.6873 |
| Myanmar | Both | 2019 | 5561.6342 | 4806.4246 | 6371.4561 |
| Cambodia | Both | 2019 | 5558.0046 | 4803.2807 | 6361.8933 |
| Republic of Moldova | Both | 2019 | 4631.4241 | 3910.5203 | 5350.4563 |
| Slovakia | Both | 2019 | 4904.5229 | 4123.4769 | 5719.9288 |
| Malaysia | Both | 2019 | 5529.0198 | 4776.5242 | 6332.6774 |
| Panama | Both | 2019 | 4883.4138 | 4196.6741 | 5579.2316 |
| India | Both | 2019 | 5147.6170 | 4408.3662 | 5915.9053 |
| Portugal | Both | 2019 | 3277.3094 | 3033.0627 | 3535.4460 |
| USA | Both | 2019 | 6824.4648 | 5904.7653 | 7797.1826 |
| Mongolia | Both | 2019 | 5313.4338 | 4522.0366 | 6134.1332 |
| Maldives | Both | 2019 | 5472.3549 | 4722.4755 | 6263.9024 |
| Cyprus | Both | 2019 | 3257.9379 | 2998.5704 | 3518.5084 |
| Denmark | Both | 2019 | 2924.0784 | 2721.0332 | 3124.3000 |
| Slovenia | Both | 2019 | 4885.4923 | 4109.5272 | 5698.1286 |
| Lao People's Democratic Republic | Both | 2019 | 5542.2692 | 4788.5736 | 6351.4586 |
| Indonesia | Both | 2019 | 6138.1585 | 5243.5160 | 7041.3116 |
| Hungary | Both | 2019 | 4960.6919 | 4176.3110 | 5777.0605 |
| Micronesia (Federated States of) | Both | 2019 | 5300.9837 | 4583.6121 | 6035.8950 |
| Kiribati | Both | 2019 | 5320.6658 | 4603.5431 | 6055.6738 |
| Burkina Faso | Both | 2019 | 5449.4635 | 4802.7300 | 6126.4148 |
| Papua New Guinea | Both | 2019 | 5289.6619 | 4571.9962 | 6024.8298 |
| Sweden | Both | 2019 | 3419.4733 | 3148.0156 | 3702.0875 |
| North Macedonia | Both | 2019 | 4888.2437 | 4112.3137 | 5699.1116 |
| Georgia | Both | 2019 | 5291.4145 | 4500.7661 | 6109.5642 |
| Fiji | Both | 2019 | 5301.3422 | 4583.1323 | 6037.0844 |
| Cuba | Both | 2019 | 4943.4644 | 4255.7451 | 5639.9396 |
| Venezuela (Bolivarian Republic of) | Both | 2019 | 4889.3820 | 4202.0850 | 5587.9904 |
| Sudan | Both | 2019 | 4757.9246 | 4091.5863 | 5442.2357 |
| Cameroon | Both | 2019 | 5495.3810 | 4862.4500 | 6173.7197 |
| United Republic of Tanzania | Both | 2019 | 5283.0267 | 4650.2726 | 5934.3737 |
| Romania | Both | 2019 | 4861.4519 | 4087.5501 | 5668.7890 |
| Iceland | Both | 2019 | 3307.4897 | 3052.0810 | 3560.8168 |
| Cook Islands | Both | 2019 | 5312.9464 | 4597.4030 | 6046.2822 |
| Honduras | Both | 2019 | 4891.8360 | 4204.4589 | 5592.7814 |
| Samoa | Both | 2019 | 5294.8758 | 4575.7080 | 6032.4155 |
| Suriname | Both | 2019 | 4947.7249 | 4260.5386 | 5646.0136 |
| Dominican Republic | Both | 2019 | 4942.6259 | 4255.3752 | 5638.7638 |
| Paraguay | Both | 2019 | 4988.0839 | 4307.7907 | 5686.6948 |
| Greenland | Both | 2019 | 5466.0183 | 4732.1258 | 6248.7828 |
| Dominica | Both | 2019 | 4937.8339 | 4253.9268 | 5632.8731 |
| Albania | Both | 2019 | 4910.1029 | 4122.7318 | 5729.0677 |
| Oman | Both | 2019 | 4714.2799 | 4053.8952 | 5380.5275 |
| Turkey | Both | 2019 | 4867.3186 | 4203.0922 | 5558.0612 |
| Estonia | Both | 2019 | 4716.7421 | 4002.0749 | 5429.2470 |
| Bahamas | Both | 2019 | 4951.2739 | 4262.7452 | 5650.9378 |
| Afghanistan | Both | 2019 | 4763.0883 | 4097.8791 | 5444.0083 |
| American Samoa | Both | 2019 | 5303.4895 | 4585.4744 | 6038.2699 |
| Philippines | Both | 2019 | 6087.1973 | 5190.9992 | 6989.4754 |
| Viet Nam | Both | 2019 | 5550.3233 | 4798.1951 | 6356.5878 |
| Timor-Leste | Both | 2019 | 5539.5047 | 4786.2677 | 6351.2563 |
| Guam | Both | 2019 | 5291.5952 | 4572.9521 | 6028.5351 |
| El Salvador | Both | 2019 | 4903.2027 | 4212.2862 | 5604.6181 |
| Saint Vincent and the Grenadines | Both | 2019 | 4936.3249 | 4253.1659 | 5629.8556 |
| Bermuda | Both | 2019 | 4948.1595 | 4260.4224 | 5645.2193 |
| Bulgaria | Both | 2019 | 4899.8458 | 4120.4523 | 5713.8790 |
| Serbia | Both | 2019 | 4905.2874 | 4124.0434 | 5720.7873 |
| Ireland | Both | 2019 | 3275.3447 | 3031.1084 | 3532.4205 |
| Algeria | Both | 2019 | 4757.8009 | 4091.8999 | 5440.8210 |
| Antigua and Barbuda | Both | 2019 | 4947.5115 | 4261.3821 | 5646.5435 |
| Equatorial Guinea | Both | 2019 | 5247.7776 | 4611.4995 | 5901.2128 |
| Italy | Both | 2019 | 3443.4691 | 3181.9917 | 3730.0392 |
| Democratic Republic of the Congo | Both | 2019 | 5249.4952 | 4616.6549 | 5901.5785 |
| Trinidad and Tobago | Both | 2019 | 4942.2579 | 4255.8669 | 5637.8376 |
| Belarus | Both | 2019 | 4633.9150 | 3913.7811 | 5358.6447 |
| Bolivia (Plurinational State of) | Both | 2019 | 5107.5024 | 4393.7912 | 5865.8184 |
| Australia | Both | 2019 | 3184.7093 | 2909.6596 | 3461.1932 |
| Saint Kitts and Nevis | Both | 2019 | 4942.7181 | 4257.0639 | 5638.3692 |
| Eritrea | Both | 2019 | 5247.1007 | 4612.7285 | 5898.0178 |
| Norway | Both | 2019 | 3567.0273 | 3243.4966 | 3903.8532 |
| Brazil | Both | 2019 | 5479.3051 | 4726.1074 | 6305.9739 |
| Kuwait | Both | 2019 | 4739.9039 | 4078.1065 | 5420.8356 |
| Marshall Islands | Both | 2019 | 5292.1905 | 4573.2840 | 6026.9443 |
| Iran (Islamic Republic of) | Both | 2019 | 5237.2652 | 4459.5942 | 6025.4664 |
| Chad | Both | 2019 | 5433.8790 | 4791.2716 | 6103.9099 |
| Poland | Both | 2019 | 5428.5255 | 4521.2061 | 6358.9018 |
| Finland | Both | 2019 | 3265.8206 | 3022.0829 | 3525.2341 |
| Uganda | Both | 2019 | 5289.5912 | 4652.2597 | 5945.2809 |
| Barbados | Both | 2019 | 4950.0219 | 4261.7572 | 5649.5916 |
| Czechia | Both | 2019 | 4897.2379 | 4118.7311 | 5710.8704 |
| Qatar | Both | 2019 | 4684.1441 | 4034.6879 | 5337.1648 |
| Peru | Both | 2019 | 5210.0955 | 4492.9162 | 5988.9898 |
| Ethiopia | Both | 2019 | 5678.4528 | 4950.5349 | 6403.0221 |
| Nicaragua | Both | 2019 | 4892.9039 | 4203.0758 | 5589.6512 |
| Chile | Both | 2019 | 3240.9560 | 2971.5822 | 3514.7119 |
| Puerto Rico | Both | 2019 | 4951.4959 | 4263.0691 | 5651.3634 |
| Eswatini | Both | 2019 | 5260.5845 | 4625.0537 | 5914.5908 |
| Argentina | Both | 2019 | 3244.3922 | 2974.0231 | 3518.5921 |
| Togo | Both | 2019 | 5450.9166 | 4803.7485 | 6125.5557 |
| Ecuador | Both | 2019 | 5123.5626 | 4406.6137 | 5888.5521 |
| Guyana | Both | 2019 | 4947.3726 | 4260.2530 | 5645.0063 |
| Kazakhstan | Both | 2019 | 5319.3782 | 4525.3116 | 6143.3203 |
| Colombia | Both | 2019 | 4891.5737 | 4202.2434 | 5589.2485 |
| Malta | Both | 2019 | 3263.2678 | 3019.5489 | 3522.6124 |
| Kenya | Both | 2019 | 5683.1855 | 4957.9827 | 6406.4581 |
| Cabo Verde | Both | 2019 | 5441.8136 | 4801.6299 | 6127.4373 |
| Azerbaijan | Both | 2019 | 5293.5871 | 4505.6259 | 6110.7799 |
| Libya | Both | 2019 | 4755.8191 | 4091.5388 | 5437.8397 |
| Haiti | Both | 2019 | 4950.2277 | 4263.3314 | 5649.8411 |
| Syrian Arab Republic | Both | 2019 | 4763.6936 | 4100.0248 | 5449.7745 |
| Yemen | Both | 2019 | 4762.2017 | 4096.4992 | 5446.4118 |
| Bahrain | Both | 2019 | 4717.5613 | 4057.9347 | 5391.6850 |
| Mozambique | Both | 2019 | 5251.1087 | 4619.3785 | 5904.8317 |
| Zimbabwe | Both | 2019 | 5260.3744 | 4624.0177 | 5917.4086 |
| Pakistan | Both | 2019 | 5348.4210 | 4612.2184 | 6081.5302 |
| Nepal | Both | 2019 | 5107.1718 | 4479.2052 | 5759.1204 |
| Nigeria | Both | 2019 | 5969.4910 | 5206.9948 | 6738.1713 |
| United Arab Emirates | Both | 2019 | 4679.8741 | 4021.9423 | 5342.3245 |
| Rwanda | Both | 2019 | 5209.6457 | 4573.1358 | 5865.6151 |
| Mexico | Both | 2019 | 5376.7101 | 4622.9699 | 6203.0766 |
| Croatia | Both | 2019 | 4906.6982 | 4124.4043 | 5722.4081 |
| Israel | Both | 2019 | 3270.8493 | 3026.9014 | 3529.4110 |
| Uruguay | Both | 2019 | 3245.7007 | 2974.9328 | 3520.4480 |
| Bangladesh | Both | 2019 | 4909.2418 | 4301.0249 | 5571.0648 |
| Andorra | Both | 2019 | 3263.8762 | 3021.5408 | 3522.0773 |
| United Kingdom | Both | 2019 | 3449.9049 | 3183.2977 | 3729.5041 |
| Tunisia | Both | 2019 | 4768.1242 | 4101.2648 | 5453.3310 |
| Bosnia and Herzegovina | Both | 2019 | 4915.3156 | 4131.8035 | 5732.0783 |
| Grenada | Both | 2019 | 4940.8830 | 4255.6271 | 5636.2440 |
| Guatemala | Both | 2019 | 4896.2273 | 4207.4984 | 5596.7022 |
| Solomon Islands | Both | 2019 | 5297.3321 | 4578.8009 | 6032.1740 |
| Luxembourg | Both | 2019 | 3266.1821 | 3023.2065 | 3524.3746 |
| Costa Rica | Both | 2019 | 4891.6793 | 4203.3531 | 5590.4105 |
| Jamaica | Both | 2019 | 4943.7759 | 4258.4703 | 5640.0365 |
| Djibouti | Both | 2019 | 5224.5690 | 4593.1734 | 5870.4865 |
| Lebanon | Both | 2019 | 4779.8787 | 4110.4748 | 5462.9977 |
| C么te d'Ivoire | Both | 2019 | 5428.8768 | 4791.0539 | 6102.8213 |
| Niger | Both | 2019 | 5441.1029 | 4795.7341 | 6120.1882 |
| Saudi Arabia | Both | 2019 | 4737.1497 | 4074.5884 | 5408.1408 |
| Belize | Both | 2019 | 4940.5600 | 4256.4158 | 5636.8185 |
| Russian Federation | Both | 2019 | 4548.6962 | 3969.9889 | 5127.8387 |
| Ukraine | Both | 2019 | 5071.7484 | 4238.5819 | 5885.6443 |
| Sao Tome and Principe | Both | 2019 | 5438.2196 | 4797.2379 | 6117.7756 |
| Bhutan | Both | 2019 | 4893.7239 | 4285.0505 | 5546.4899 |
| San Marino | Both | 2019 | 3286.4146 | 3041.9803 | 3542.6820 |
| Morocco | Both | 2019 | 4761.2558 | 4095.4476 | 5445.0945 |
| Egypt | Both | 2019 | 4740.9832 | 4080.0870 | 5423.9579 |
| Madagascar | Both | 2019 | 5239.5011 | 4608.3693 | 5891.4314 |
| Zambia | Both | 2019 | 5240.9365 | 4609.6849 | 5891.0652 |
| Saint Lucia | Both | 2019 | 4944.2380 | 4257.9154 | 5640.7820 |
| Tokelau | Both | 2019 | 5293.0990 | 4574.0231 | 6029.8481 |
| Northern Mariana Islands | Both | 2019 | 5289.2197 | 4569.9019 | 6025.5889 |
| Malawi | Both | 2019 | 5248.2346 | 4615.5443 | 5899.6831 |
| Gambia | Both | 2019 | 5441.1023 | 4797.4682 | 6118.4723 |
| Seychelles | Both | 2019 | 5521.4670 | 4770.0611 | 6320.2461 |
| Angola | Both | 2019 | 5255.1739 | 4620.7294 | 5911.5771 |
| Palestine | Both | 2019 | 4762.2452 | 4097.2520 | 5445.8981 |
| Palau | Both | 2019 | 5270.4489 | 4549.0774 | 6003.3235 |
| Mauritius | Both | 2019 | 5545.4416 | 4792.6494 | 6355.2315 |
| Guinea | Both | 2019 | 5443.0037 | 4799.0663 | 6116.5408 |
| Liberia | Both | 2019 | 5433.9072 | 4794.3292 | 6108.2587 |
| Guinea-Bissau | Both | 2019 | 5448.6643 | 4802.1956 | 6126.4628 |
| Tuvalu | Both | 2019 | 5293.4137 | 4572.7350 | 6029.4426 |
| Ghana | Both | 2019 | 5489.8182 | 4856.1397 | 6176.9975 |
| Central African Republic | Both | 2019 | 5254.5329 | 4620.3418 | 5907.0163 |
| Senegal | Both | 2019 | 5439.8383 | 4795.1738 | 6117.8658 |
| Austria | Both | 2019 | 3266.3209 | 3022.8527 | 3525.3368 |
| Botswana | Both | 2019 | 5254.2091 | 4620.6910 | 5907.5652 |
| South Sudan | Both | 2019 | 5236.6923 | 4606.7462 | 5889.4884 |
| Netherlands | Both | 2019 | 3073.6063 | 2855.6828 | 3292.8616 |
| Gabon | Both | 2019 | 5251.4995 | 4619.9802 | 5904.3849 |
| Iraq | Both | 2019 | 4760.5712 | 4092.2553 | 5441.8808 |
| Mali | Both | 2019 | 5444.5627 | 4799.8462 | 6121.1608 |
| Sierra Leone | Both | 2019 | 5437.6115 | 4795.8789 | 6113.4927 |
| Congo | Both | 2019 | 5246.7924 | 4614.3941 | 5898.3746 |
| Belgium | Both | 2019 | 3270.5632 | 3027.1626 | 3528.6798 |
| United States Virgin Islands | Both | 2019 | 4953.4944 | 4266.1587 | 5654.1650 |
| Jordan | Both | 2019 | 4750.0726 | 4087.2614 | 5430.0330 |
| Lesotho | Both | 2019 | 5255.9189 | 4621.6336 | 5909.2454 |
| Canada | Both | 2019 | 5504.5819 | 4760.2227 | 6296.3265 |
| South Africa | Both | 2019 | 5709.6840 | 4981.4064 | 6435.9013 |
| Mauritania | Both | 2019 | 5440.0928 | 4795.8695 | 6115.8224 |
| Namibia | Both | 2019 | 5256.5640 | 4622.0728 | 5912.6557 |
| Monaco | Both | 2019 | 3276.9002 | 3033.2326 | 3534.7934 |
| Nauru | Both | 2019 | 5308.6499 | 4585.7453 | 6041.6447 |
| Niue | Both | 2019 | 5302.0086 | 4583.5668 | 6039.6636 |
|  |  |  |  |  |  |

**Table7 DALYs of dermatitis in 2019, Page 17-21**

| **location_name** | **sex_name** | **cause_name** | **year** | **Age-standardised DALYs rate (per 100 000 person-years)** | **95% CI(lower)** | **95% CI(upper)** |
| --- | --- | --- | --- | --- | --- | --- |
| Luxembourg | Both | Dermatitis | 2019 | 175.2142 | 95.4721 | 288.8818 |
| China | Both | Dermatitis | 2019 | 143.4617 | 84.4568 | 224.8485 |
| Belize | Both | Dermatitis | 2019 | 115.5029 | 68.3422 | 182.0130 |
| Kiribati | Both | Dermatitis | 2019 | 165.9170 | 96.3005 | 263.4811 |
| Malaysia | Both | Dermatitis | 2019 | 173.8057 | 100.7918 | 275.7581 |
| Maldives | Both | Dermatitis | 2019 | 165.0638 | 96.2939 | 260.5333 |
| Argentina | Both | Dermatitis | 2019 | 146.8887 | 81.7760 | 240.3382 |
| Myanmar | Both | Dermatitis | 2019 | 177.4077 | 102.7871 | 282.5804 |
| Lao People's Democratic Republic | Both | Dermatitis | 2019 | 175.5277 | 103.1442 | 278.4918 |
| Cuba | Both | Dermatitis | 2019 | 115.2619 | 67.7211 | 180.8663 |
| Malta | Both | Dermatitis | 2019 | 174.9942 | 96.3877 | 287.9471 |
| Trinidad and Tobago | Both | Dermatitis | 2019 | 115.2789 | 68.3005 | 183.1782 |
| Belgium | Both | Dermatitis | 2019 | 175.5722 | 96.3179 | 289.9380 |
| Micronesia (Federated States of) | Both | Dermatitis | 2019 | 164.2228 | 95.3387 | 259.0285 |
| Marshall Islands | Both | Dermatitis | 2019 | 163.4227 | 95.4409 | 259.6053 |
| Mexico | Both | Dermatitis | 2019 | 98.5747 | 59.4418 | 153.8884 |
| Cyprus | Both | Dermatitis | 2019 | 165.4998 | 91.1260 | 277.1402 |
| Chile | Both | Dermatitis | 2019 | 146.6330 | 81.5510 | 242.9028 |
| Solomon Islands | Both | Dermatitis | 2019 | 164.6804 | 96.1740 | 260.7369 |
| Azerbaijan | Both | Dermatitis | 2019 | 241.2404 | 136.9586 | 390.8953 |
| Nicaragua | Both | Dermatitis | 2019 | 94.3766 | 56.2210 | 146.2067 |
| Papua New Guinea | Both | Dermatitis | 2019 | 163.2514 | 95.1563 | 260.3717 |
| Dominica | Both | Dermatitis | 2019 | 115.2637 | 67.8630 | 180.5123 |
| Uruguay | Both | Dermatitis | 2019 | 146.8860 | 81.3944 | 243.6459 |
| Kazakhstan | Both | Dermatitis | 2019 | 243.7333 | 137.4822 | 400.1568 |
| Tonga | Both | Dermatitis | 2019 | 166.5557 | 96.9075 | 265.0779 |
| Bulgaria | Both | Dermatitis | 2019 | 122.7307 | 73.6919 | 193.2016 |
| Hungary | Both | Dermatitis | 2019 | 155.2551 | 91.2529 | 246.1341 |
| Georgia | Both | Dermatitis | 2019 | 239.7196 | 136.1015 | 389.7758 |
| North Macedonia | Both | Dermatitis | 2019 | 122.4807 | 72.5058 | 191.2993 |
| Latvia | Both | Dermatitis | 2019 | 92.5997 | 56.7029 | 142.9486 |
| Romania | Both | Dermatitis | 2019 | 97.2245 | 58.6100 | 151.0367 |
| Syrian Arab Republic | Both | Dermatitis | 2019 | 89.6683 | 54.4222 | 139.9950 |
| Lithuania | Both | Dermatitis | 2019 | 125.2958 | 74.2152 | 198.2850 |
| Singapore | Both | Dermatitis | 2019 | 172.0594 | 94.9235 | 283.7327 |
| Dominican Republic | Both | Dermatitis | 2019 | 115.5892 | 67.6292 | 182.5589 |
| Puerto Rico | Both | Dermatitis | 2019 | 115.7062 | 68.3315 | 182.0633 |
| Indonesia | Both | Dermatitis | 2019 | 200.3172 | 115.4761 | 320.0415 |
| Mongolia | Both | Dermatitis | 2019 | 244.0416 | 139.1489 | 397.7763 |
| Samoa | Both | Dermatitis | 2019 | 164.3167 | 95.1056 | 262.2787 |
| Czechia | Both | Dermatitis | 2019 | 122.5178 | 72.4938 | 192.0462 |
| Croatia | Both | Dermatitis | 2019 | 122.7926 | 73.1571 | 191.9392 |
| Belarus | Both | Dermatitis | 2019 | 125.2646 | 74.3279 | 197.7276 |
| Panama | Both | Dermatitis | 2019 | 94.3414 | 56.8712 | 147.6094 |
| Republic of Moldova | Both | Dermatitis | 2019 | 125.1105 | 74.1735 | 197.2328 |
| Turkmenistan | Both | Dermatitis | 2019 | 241.7089 | 137.4128 | 396.2237 |
| Ecuador | Both | Dermatitis | 2019 | 125.0273 | 73.9903 | 197.5246 |
| Tunisia | Both | Dermatitis | 2019 | 89.8501 | 53.9494 | 137.5022 |
| Tajikistan | Both | Dermatitis | 2019 | 242.4172 | 136.5976 | 397.5173 |
| Australia | Both | Dermatitis | 2019 | 122.6240 | 68.2519 | 201.8104 |
| Armenia | Both | Dermatitis | 2019 | 241.9185 | 135.6114 | 398.3650 |
| Estonia | Both | Dermatitis | 2019 | 245.3806 | 138.4801 | 400.3282 |
| Spain | Both | Dermatitis | 2019 | 160.7559 | 88.1907 | 267.7844 |
| Kyrgyzstan | Both | Dermatitis | 2019 | 243.4831 | 138.0166 | 394.1162 |
| Libya | Both | Dermatitis | 2019 | 89.2037 | 53.5956 | 139.0218 |
| Poland | Both | Dermatitis | 2019 | 121.5147 | 72.1541 | 189.3839 |
| Thailand | Both | Dermatitis | 2019 | 176.9693 | 103.3361 | 280.1353 |
| Philippines | Both | Dermatitis | 2019 | 179.6873 | 104.5488 | 283.3061 |
| Saint Kitts and Nevis | Both | Dermatitis | 2019 | 115.5232 | 68.2654 | 183.3465 |
| United Kingdom | Both | Dermatitis | 2019 | 199.6712 | 110.1850 | 330.7798 |
| Qatar | Both | Dermatitis | 2019 | 86.2406 | 52.1436 | 132.9717 |
| Senegal | Both | Dermatitis | 2019 | 78.6531 | 46.8878 | 122.5528 |
| Turkey | Both | Dermatitis | 2019 | 138.7189 | 80.9086 | 222.6255 |
| Guam | Both | Dermatitis | 2019 | 164.0798 | 94.6729 | 261.1138 |
| Tokelau | Both | Dermatitis | 2019 | 164.4435 | 95.8355 | 261.0233 |
| Switzerland | Both | Dermatitis | 2019 | 175.3480 | 96.1785 | 291.4001 |
| Kuwait | Both | Dermatitis | 2019 | 89.1953 | 53.4091 | 138.4261 |
| Italy | Both | Dermatitis | 2019 | 201.3663 | 110.1225 | 334.9727 |
| France | Both | Dermatitis | 2019 | 244.9062 | 134.2995 | 406.4230 |
| Timor-Leste | Both | Dermatitis | 2019 | 175.1901 | 102.2973 | 277.0282 |
| Andorra | Both | Dermatitis | 2019 | 175.2643 | 95.9074 | 292.3938 |
| American Samoa | Both | Dermatitis | 2019 | 164.8152 | 95.7987 | 263.7601 |
| Bermuda | Both | Dermatitis | 2019 | 116.0187 | 68.1881 | 182.3301 |
| Iran (Islamic Republic of) | Both | Dermatitis | 2019 | 93.9813 | 57.2029 | 145.2255 |
| Russian Federation | Both | Dermatitis | 2019 | 184.4547 | 105.4721 | 297.2389 |
| Congo | Both | Dermatitis | 2019 | 76.9501 | 46.5846 | 118.0952 |
| Grenada | Both | Dermatitis | 2019 | 115.1267 | 68.6646 | 179.9548 |
| San Marino | Both | Dermatitis | 2019 | 176.4722 | 96.6218 | 290.7675 |
| Ethiopia | Both | Dermatitis | 2019 | 74.0383 | 45.2767 | 112.0334 |
| Fiji | Both | Dermatitis | 2019 | 163.8815 | 94.9103 | 260.3245 |
| Slovenia | Both | Dermatitis | 2019 | 122.4293 | 73.1492 | 192.5647 |
| Viet Nam | Both | Dermatitis | 2019 | 176.6183 | 102.2870 | 280.1843 |
| Gambia | Both | Dermatitis | 2019 | 78.7189 | 47.3450 | 122.0687 |
| Brunei Darussalam | Both | Dermatitis | 2019 | 189.1946 | 103.0737 | 314.2394 |
| Germany | Both | Dermatitis | 2019 | 178.3181 | 98.4868 | 291.3697 |
| Suriname | Both | Dermatitis | 2019 | 115.1441 | 68.5470 | 182.1181 |
| Montenegro | Both | Dermatitis | 2019 | 122.8948 | 72.6921 | 193.6425 |
| United Arab Emirates | Both | Dermatitis | 2019 | 86.9821 | 52.1011 | 135.9223 |
| Austria | Both | Dermatitis | 2019 | 175.1001 | 96.1658 | 289.1909 |
| Albania | Both | Dermatitis | 2019 | 123.0723 | 72.9022 | 193.6607 |
| Bosnia and Herzegovina | Both | Dermatitis | 2019 | 122.7983 | 72.7273 | 191.1304 |
| Greenland | Both | Dermatitis | 2019 | 127.0504 | 74.3841 | 199.5131 |
| Uzbekistan | Both | Dermatitis | 2019 | 242.3436 | 136.8326 | 393.1561 |
| Angola | Both | Dermatitis | 2019 | 77.4289 | 46.6010 | 120.0795 |
| Eritrea | Both | Dermatitis | 2019 | 75.0757 | 45.5792 | 116.1479 |
| Saudi Arabia | Both | Dermatitis | 2019 | 88.2822 | 52.9982 | 137.9130 |
| Greece | Both | Dermatitis | 2019 | 142.2042 | 78.9740 | 240.4573 |
| Venezuela (Bolivarian Republic of) | Both | Dermatitis | 2019 | 94.3652 | 56.6954 | 147.5016 |
| Lesotho | Both | Dermatitis | 2019 | 76.8673 | 46.3050 | 119.0606 |
| Pakistan | Both | Dermatitis | 2019 | 112.6613 | 66.7150 | 175.6676 |
| Lebanon | Both | Dermatitis | 2019 | 89.7641 | 53.7416 | 137.5318 |
| Kenya | Both | Dermatitis | 2019 | 73.3042 | 44.8220 | 112.0602 |
| Tuvalu | Both | Dermatitis | 2019 | 163.3826 | 94.9504 | 260.9125 |
| Iraq | Both | Dermatitis | 2019 | 89.1229 | 53.9898 | 137.3105 |
| Bolivia (Plurinational State of) | Both | Dermatitis | 2019 | 116.6507 | 69.8236 | 181.9254 |
| Ireland | Both | Dermatitis | 2019 | 175.7287 | 97.9372 | 290.6830 |
| Burkina Faso | Both | Dermatitis | 2019 | 78.8439 | 47.5221 | 121.5021 |
| New Zealand | Both | Dermatitis | 2019 | 162.5271 | 89.5205 | 266.8282 |
| Jordan | Both | Dermatitis | 2019 | 89.1415 | 53.5923 | 138.6758 |
| Sierra Leone | Both | Dermatitis | 2019 | 78.6618 | 47.5431 | 120.3570 |
| Djibouti | Both | Dermatitis | 2019 | 74.9330 | 45.7847 | 116.2396 |
| Barbados | Both | Dermatitis | 2019 | 115.7425 | 68.3570 | 183.3690 |
| Vanuatu | Both | Dermatitis | 2019 | 164.8932 | 95.6834 | 262.9326 |
| Yemen | Both | Dermatitis | 2019 | 88.9940 | 53.7706 | 138.0777 |
| Central African Republic | Both | Dermatitis | 2019 | 76.8631 | 46.9416 | 118.4362 |
| Botswana | Both | Dermatitis | 2019 | 76.9883 | 45.9703 | 118.2969 |
| Iceland | Both | Dermatitis | 2019 | 197.7339 | 107.6950 | 329.9356 |
| Peru | Both | Dermatitis | 2019 | 111.0661 | 66.4362 | 173.5611 |
| Cameroon | Both | Dermatitis | 2019 | 87.0013 | 52.0525 | 132.9342 |
| Sweden | Both | Dermatitis | 2019 | 177.9017 | 97.7366 | 293.8071 |
| Honduras | Both | Dermatitis | 2019 | 94.2532 | 56.8641 | 145.7238 |
| Cambodia | Both | Dermatitis | 2019 | 176.3486 | 102.3682 | 278.4563 |
| Israel | Both | Dermatitis | 2019 | 175.7949 | 96.9568 | 289.2353 |
| Democratic People's Republic of Korea | Both | Dermatitis | 2019 | 155.1847 | 90.8107 | 246.6115 |
| Cook Islands | Both | Dermatitis | 2019 | 167.1190 | 96.8959 | 262.3281 |
| Madagascar | Both | Dermatitis | 2019 | 75.2369 | 44.9593 | 115.5704 |
| Uganda | Both | Dermatitis | 2019 | 93.4504 | 55.4253 | 145.8938 |
| Finland | Both | Dermatitis | 2019 | 175.4054 | 96.0097 | 291.9713 |
| Chad | Both | Dermatitis | 2019 | 78.5128 | 47.4784 | 120.5880 |
| Denmark | Both | Dermatitis | 2019 | 211.6843 | 112.4577 | 353.1747 |
| Bahrain | Both | Dermatitis | 2019 | 88.0707 | 52.9156 | 137.2554 |
| Bhutan | Both | Dermatitis | 2019 | 109.0640 | 63.9066 | 171.8318 |
| Mauritania | Both | Dermatitis | 2019 | 78.9629 | 47.5147 | 120.8687 |
| Sri Lanka | Both | Dermatitis | 2019 | 163.5972 | 95.7806 | 260.2389 |
| Brazil | Both | Dermatitis | 2019 | 137.7985 | 81.0081 | 215.9230 |
| C么te d'Ivoire | Both | Dermatitis | 2019 | 78.3424 | 47.4245 | 121.4994 |
| Zambia | Both | Dermatitis | 2019 | 74.9139 | 45.2088 | 114.4689 |
| Cabo Verde | Both | Dermatitis | 2019 | 79.1903 | 47.6624 | 122.2854 |
| Taiwan (Province of China) | Both | Dermatitis | 2019 | 182.7161 | 105.4951 | 295.0938 |
| India | Both | Dermatitis | 2019 | 112.0028 | 66.3938 | 173.8211 |
| Malawi | Both | Dermatitis | 2019 | 75.2699 | 45.7625 | 114.9816 |
| Burundi | Both | Dermatitis | 2019 | 74.9592 | 45.2264 | 114.6999 |
| Japan | Both | Dermatitis | 2019 | 249.5939 | 135.8556 | 416.6162 |
| Ukraine | Both | Dermatitis | 2019 | 131.5109 | 77.4713 | 205.9828 |
| Republic of Korea | Both | Dermatitis | 2019 | 190.9873 | 105.7173 | 317.6920 |
| Egypt | Both | Dermatitis | 2019 | 68.4362 | 41.7299 | 105.7235 |
| Palau | Both | Dermatitis | 2019 | 159.6139 | 92.2781 | 254.2234 |
| Niger | Both | Dermatitis | 2019 | 78.7390 | 47.7299 | 122.3960 |
| Canada | Both | Dermatitis | 2019 | 128.3511 | 76.6448 | 199.3156 |
| Nepal | Both | Dermatitis | 2019 | 110.7715 | 64.7172 | 174.8945 |
| Nigeria | Both | Dermatitis | 2019 | 83.1826 | 50.6463 | 127.6039 |
| Comoros | Both | Dermatitis | 2019 | 75.3907 | 45.5022 | 115.8962 |
| USA | Both | Dermatitis | 2019 | 186.2657 | 109.8738 | 292.8049 |
| Netherlands | Both | Dermatitis | 2019 | 173.7575 | 95.3662 | 288.3426 |
| Bangladesh | Both | Dermatitis | 2019 | 110.0123 | 64.6495 | 173.6883 |
| Serbia | Both | Dermatitis | 2019 | 122.8084 | 72.3482 | 192.9124 |
| Norway | Both | Dermatitis | 2019 | 198.8253 | 108.4091 | 326.9992 |
| Colombia | Both | Dermatitis | 2019 | 94.4178 | 56.2530 | 146.5773 |
| Zimbabwe | Both | Dermatitis | 2019 | 77.5118 | 46.7455 | 119.8341 |
| Antigua and Barbuda | Both | Dermatitis | 2019 | 115.5823 | 68.0597 | 179.3601 |
| Sao Tome and Principe | Both | Dermatitis | 2019 | 79.0832 | 47.5811 | 122.8290 |
| Portugal | Both | Dermatitis | 2019 | 175.8970 | 97.1242 | 290.1092 |
| Costa Rica | Both | Dermatitis | 2019 | 94.5563 | 56.5062 | 148.0380 |
| Slovakia | Both | Dermatitis | 2019 | 122.8442 | 72.7886 | 191.7845 |
| Mauritius | Both | Dermatitis | 2019 | 174.7436 | 102.3275 | 276.4057 |
| Seychelles | Both | Dermatitis | 2019 | 171.7840 | 99.9647 | 273.7576 |
| Benin | Both | Dermatitis | 2019 | 78.9139 | 47.5445 | 121.0728 |
| Saint Lucia | Both | Dermatitis | 2019 | 115.2847 | 68.2061 | 183.5173 |
| Bahamas | Both | Dermatitis | 2019 | 115.8076 | 68.2834 | 182.2134 |
| Mozambique | Both | Dermatitis | 2019 | 74.9893 | 45.2607 | 114.8418 |
| Equatorial Guinea | Both | Dermatitis | 2019 | 76.6603 | 46.1753 | 118.2774 |
| Somalia | Both | Dermatitis | 2019 | 74.9528 | 45.3921 | 116.7848 |
| Rwanda | Both | Dermatitis | 2019 | 59.6079 | 36.6174 | 89.3525 |
| Saint Vincent and the Grenadines | Both | Dermatitis | 2019 | 115.1339 | 68.1482 | 181.5376 |
| Morocco | Both | Dermatitis | 2019 | 89.5291 | 54.0159 | 138.9560 |
| Gabon | Both | Dermatitis | 2019 | 77.0284 | 46.6467 | 118.9687 |
| Algeria | Both | Dermatitis | 2019 | 89.6212 | 53.1330 | 138.2067 |
| United Republic of Tanzania | Both | Dermatitis | 2019 | 93.0147 | 55.1357 | 142.4130 |
| El Salvador | Both | Dermatitis | 2019 | 94.4237 | 56.4830 | 147.1989 |
| Palestine | Both | Dermatitis | 2019 | 89.1723 | 53.4109 | 139.0261 |
| South Sudan | Both | Dermatitis | 2019 | 74.5625 | 44.7264 | 114.6610 |
| Liberia | Both | Dermatitis | 2019 | 78.1827 | 47.1571 | 119.9491 |
| Guyana | Both | Dermatitis | 2019 | 114.8015 | 68.1228 | 180.9741 |
| Guatemala | Both | Dermatitis | 2019 | 94.2069 | 56.9098 | 145.7825 |
| Oman | Both | Dermatitis | 2019 | 87.7125 | 52.9386 | 136.9779 |
| Haiti | Both | Dermatitis | 2019 | 114.8209 | 67.9656 | 179.9166 |
| Nauru | Both | Dermatitis | 2019 | 165.5031 | 94.6565 | 261.7975 |
| Namibia | Both | Dermatitis | 2019 | 77.2792 | 46.0930 | 119.1019 |
| Mali | Both | Dermatitis | 2019 | 82.1353 | 49.7094 | 127.7827 |
| Jamaica | Both | Dermatitis | 2019 | 115.4108 | 68.2294 | 181.5113 |
| South Africa | Both | Dermatitis | 2019 | 80.4877 | 48.4739 | 123.0535 |
| Niue | Both | Dermatitis | 2019 | 164.4733 | 95.2203 | 261.5005 |
| Paraguay | Both | Dermatitis | 2019 | 136.4629 | 79.4440 | 214.4040 |
| Democratic Republic of the Congo | Both | Dermatitis | 2019 | 76.8653 | 46.8111 | 118.6991 |
| Eswatini | Both | Dermatitis | 2019 | 76.9629 | 46.8102 | 119.3491 |
| Togo | Both | Dermatitis | 2019 | 78.8961 | 47.9330 | 120.8122 |
| Ghana | Both | Dermatitis | 2019 | 69.4493 | 42.2650 | 107.3541 |
| Northern Mariana Islands | Both | Dermatitis | 2019 | 163.8060 | 94.4704 | 260.9626 |
| Guinea | Both | Dermatitis | 2019 | 78.8301 | 47.8191 | 121.1912 |
| United States Virgin Islands | Both | Dermatitis | 2019 | 115.8922 | 67.5417 | 183.7528 |
| Guinea-Bissau | Both | Dermatitis | 2019 | 78.8657 | 47.5962 | 122.7044 |
| Monaco | Both | Dermatitis | 2019 | 176.2287 | 96.4648 | 292.6942 |
| Afghanistan | Both | Dermatitis | 2019 | 88.4678 | 52.6043 | 139.1401 |
| Sudan | Both | Dermatitis | 2019 | 89.3188 | 53.3797 | 139.4139 |
|  |  |  |  |  |  |  |

**Table8 Incidence of AD stratified by sex in 1990~2019, Page 22-24**

| **cause_name** | **location_name** | **sex_name** | **year** | **Age-standardised incidence rate (per 100 000 person-years)** | **95%CI(lower)** | **95%CI(upper)** |
| --- | --- | --- | --- | --- | --- | --- |
| AD | Global | Male | 1990 | 272.3400 | 259.0031 | 286.1006 |
| AD | Global | Female | 1990 | 413.8120 | 394.9024 | 433.1748 |
| AD | Global | Both | 1990 | 342.3050 | 327.0355 | 358.4175 |
| AD | Global | Male | 1992 | 272.4704 | 259.6197 | 286.2580 |
| AD | Global | Female | 1992 | 412.8804 | 394.0120 | 431.8136 |
| AD | Global | Both | 1992 | 341.8515 | 326.5309 | 357.9311 |
| AD | Global | Male | 1991 | 272.4133 | 259.2592 | 286.4110 |
| AD | Global | Female | 1991 | 413.3531 | 394.6373 | 432.4538 |
| AD | Global | Both | 1991 | 342.0828 | 326.7936 | 358.2657 |
| AD | Global | Male | 1994 | 272.5063 | 259.8702 | 286.4735 |
| AD | Global | Female | 1994 | 411.8590 | 393.0592 | 430.5864 |
| AD | Global | Both | 1994 | 341.3385 | 325.9302 | 357.3515 |
| AD | Global | Male | 1993 | 272.5002 | 259.9002 | 286.6199 |
| AD | Global | Female | 1993 | 412.3998 | 393.4797 | 431.4509 |
| AD | Global | Both | 1993 | 341.6102 | 326.2494 | 357.4832 |
| AD | Global | Male | 1995 | 272.4654 | 259.7318 | 286.0089 |
| AD | Global | Female | 1995 | 411.2727 | 392.5530 | 429.9634 |
| AD | Global | Both | 1995 | 341.0297 | 325.7256 | 357.0027 |
| AD | Global | Male | 1996 | 272.5384 | 259.8472 | 286.1725 |
| AD | Global | Female | 1996 | 410.7816 | 392.0865 | 429.2853 |
| AD | Global | Both | 1996 | 340.8303 | 325.5656 | 356.8282 |
| AD | Global | Male | 1998 | 273.1578 | 260.5393 | 286.9599 |
| AD | Global | Female | 1998 | 410.3277 | 391.8301 | 428.7601 |
| AD | Global | Both | 1998 | 340.9495 | 325.7181 | 356.9400 |
| AD | Global | Male | 1997 | 272.7958 | 260.1029 | 286.5154 |
| AD | Global | Female | 1997 | 410.4831 | 391.8339 | 428.9647 |
| AD | Global | Both | 1997 | 340.8256 | 325.5982 | 356.8368 |
| AD | Global | Male | 1999 | 273.5014 | 260.9069 | 287.2601 |
| AD | Global | Female | 1999 | 410.1511 | 391.6534 | 428.5236 |
| AD | Global | Both | 1999 | 341.0569 | 325.8156 | 357.0257 |
| AD | Global | Male | 2000 | 273.6719 | 261.1017 | 287.3590 |
| AD | Global | Female | 2000 | 409.7779 | 391.2106 | 427.9706 |
| AD | Global | Both | 2000 | 340.9791 | 325.7384 | 356.9292 |
| AD | Global | Male | 2001 | 273.6903 | 261.0906 | 287.3520 |
| AD | Global | Female | 2001 | 409.2423 | 390.9076 | 427.5109 |
| AD | Global | Both | 2001 | 340.7385 | 325.4575 | 356.6937 |
| AD | Global | Male | 2002 | 273.5898 | 260.9505 | 287.2543 |
| AD | Global | Female | 2002 | 408.5891 | 390.5099 | 426.7289 |
| AD | Global | Both | 2002 | 340.3747 | 325.0463 | 356.3574 |
| AD | Global | Male | 2004 | 273.2033 | 260.4945 | 286.8556 |
| AD | Global | Female | 2004 | 407.0697 | 389.2170 | 425.2624 |
| AD | Global | Both | 2004 | 339.4394 | 324.2848 | 355.4934 |
| AD | Global | Male | 2003 | 273.4129 | 260.8437 | 287.0160 |
| AD | Global | Female | 2003 | 407.8521 | 389.8417 | 425.9772 |
| AD | Global | Both | 2003 | 339.9279 | 324.6264 | 356.0212 |
| AD | Global | Male | 2006 | 272.6301 | 260.1864 | 286.3815 |
| AD | Global | Female | 2006 | 405.3544 | 387.4614 | 423.9499 |
| AD | Global | Both | 2006 | 338.3032 | 323.1693 | 354.4342 |
| AD | Global | Male | 2005 | 272.9347 | 260.1984 | 286.7762 |
| AD | Global | Female | 2005 | 406.2171 | 388.3369 | 424.5031 |
| AD | Global | Both | 2005 | 338.8859 | 323.7432 | 354.8851 |
| AD | Global | Male | 2007 | 272.2773 | 259.8607 | 285.8295 |
| AD | Global | Female | 2007 | 404.4842 | 386.6557 | 423.0892 |
| AD | Global | Both | 2007 | 337.6854 | 322.4273 | 353.6775 |
| AD | Global | Male | 2008 | 271.9030 | 259.3598 | 285.5027 |
| AD | Global | Female | 2008 | 403.5512 | 385.5707 | 421.9955 |
| AD | Global | Both | 2008 | 337.0254 | 321.8160 | 352.9380 |
| AD | Global | Male | 2010 | 271.2841 | 258.5124 | 284.8002 |
| AD | Global | Female | 2010 | 401.7030 | 383.2178 | 420.3631 |
| AD | Global | Both | 2010 | 335.7984 | 320.3534 | 352.0703 |
| AD | Global | Male | 2009 | 271.5519 | 258.8457 | 285.1099 |
| AD | Global | Female | 2009 | 402.5791 | 384.1254 | 420.9607 |
| AD | Global | Both | 2009 | 336.3642 | 321.1020 | 352.5601 |
| AD | Global | Male | 2012 | 270.3498 | 257.6831 | 284.3995 |
| AD | Global | Female | 2012 | 399.6620 | 381.2881 | 417.9818 |
| AD | Global | Both | 2012 | 334.3158 | 319.1697 | 350.4963 |
| AD | Global | Male | 2014 | 268.9868 | 256.3471 | 282.2769 |
| AD | Global | Female | 2014 | 397.0159 | 378.8127 | 415.7429 |
| AD | Global | Both | 2014 | 332.3125 | 317.1769 | 348.2579 |
| AD | Global | Male | 2013 | 269.6587 | 256.9880 | 283.3957 |
| AD | Global | Female | 2013 | 398.3727 | 379.9629 | 416.8844 |
| AD | Global | Both | 2013 | 333.3268 | 318.3105 | 349.3894 |
| AD | Global | Male | 2011 | 270.9316 | 258.2755 | 284.8298 |
| AD | Global | Female | 2011 | 400.7879 | 382.2513 | 419.2801 |
| AD | Global | Both | 2011 | 335.1685 | 319.8666 | 351.4238 |
| AD | Global | Male | 2015 | 268.4274 | 255.5082 | 281.9346 |
| AD | Global | Female | 2015 | 395.7023 | 377.4427 | 414.5003 |
| AD | Global | Both | 2015 | 331.3748 | 316.1018 | 347.1990 |
| AD | Global | Male | 2016 | 267.9319 | 255.1133 | 281.4065 |
| AD | Global | Female | 2016 | 394.4059 | 376.1296 | 413.1103 |
| AD | Global | Both | 2016 | 330.4757 | 315.2305 | 346.2560 |
| AD | Global | Male | 2018 | 266.8928 | 254.2281 | 280.2019 |
| AD | Global | Female | 2018 | 391.5977 | 373.5117 | 410.0360 |
| AD | Global | Both | 2018 | 328.5466 | 313.2764 | 344.2054 |
| AD | Global | Male | 2017 | 267.3433 | 254.6100 | 280.7562 |
| AD | Global | Female | 2017 | 392.9426 | 374.6274 | 411.7744 |
| AD | Global | Both | 2017 | 329.4468 | 314.2388 | 345.1537 |
| AD | Global | Male | 2019 | 266.6894 | 253.8420 | 280.2492 |
| AD | Global | Female | 2019 | 390.5420 | 372.4707 | 408.3218 |
| AD | Global | Both | 2019 | 327.9136 | 312.7599 | 343.6677 |
|  |  |  |  |  |  |  |

**Table9 Incidence of AD stratified by SDI in 1990~2019,Global, Page 25-26**

| **location_name** | **cause_name** | **sex_name** | **year** | **Age-standardised DALYs rate (per 100 000 person-years)** | **95%CI(lower)** | **95%CI(upper)** |
| --- | --- | --- | --- | --- | --- | --- |
| Global | AD | Male | 1990 | 84.5891 | 45.1163 | 142.1066 |
| Global | AD | Female | 1990 | 123.7267 | 65.7921 | 207.2789 |
| Global | AD | Both | 1990 | 103.9911 | 55.3169 | 174.5661 |
| Global | AD | Male | 1991 | 84.5209 | 45.1142 | 142.1701 |
| Global | AD | Female | 1991 | 123.6112 | 65.8257 | 207.7248 |
| Global | AD | Both | 1991 | 103.8902 | 55.3838 | 174.6280 |
| Global | AD | Male | 1992 | 84.4560 | 45.0324 | 142.0333 |
| Global | AD | Female | 1992 | 123.5173 | 65.7630 | 207.7435 |
| Global | AD | Both | 1992 | 103.8033 | 55.2332 | 174.6960 |
| Global | AD | Male | 1994 | 84.2973 | 45.1470 | 141.6295 |
| Global | AD | Female | 1994 | 123.2135 | 65.7140 | 207.6138 |
| Global | AD | Both | 1994 | 103.5646 | 55.2742 | 174.2763 |
| Global | AD | Male | 1993 | 84.3866 | 45.2339 | 141.8378 |
| Global | AD | Female | 1993 | 123.3763 | 65.7449 | 207.7418 |
| Global | AD | Both | 1993 | 103.6928 | 55.4024 | 174.3778 |
| Global | AD | Male | 1995 | 84.1840 | 45.1677 | 141.5869 |
| Global | AD | Female | 1995 | 123.0118 | 65.6827 | 207.6106 |
| Global | AD | Both | 1995 | 103.4079 | 55.2827 | 174.2878 |
| Global | AD | Male | 1996 | 84.0943 | 45.2321 | 141.2048 |
| Global | AD | Female | 1996 | 122.8155 | 65.8024 | 206.9243 |
| Global | AD | Both | 1996 | 103.2676 | 55.2193 | 173.8933 |
| Global | AD | Male | 1997 | 84.0378 | 45.2045 | 141.1772 |
| Global | AD | Female | 1997 | 122.7085 | 65.6161 | 206.4862 |
| Global | AD | Both | 1997 | 103.1895 | 55.1626 | 173.2932 |
| Global | AD | Male | 1998 | 83.9983 | 45.1244 | 141.3450 |
| Global | AD | Female | 1998 | 122.6251 | 65.5272 | 206.9256 |
| Global | AD | Both | 1998 | 103.1334 | 55.1354 | 173.7863 |
| Global | AD | Male | 1999 | 83.9784 | 45.0418 | 141.4678 |
| Global | AD | Female | 1999 | 122.5309 | 65.3930 | 206.1819 |
| Global | AD | Both | 1999 | 103.0832 | 55.1311 | 173.5122 |
| Global | AD | Male | 2000 | 83.9176 | 44.9782 | 141.4151 |
| Global | AD | Female | 2000 | 122.3798 | 65.4498 | 206.4104 |
| Global | AD | Both | 2000 | 102.9841 | 55.0087 | 173.4829 |
| Global | AD | Male | 2002 | 83.7057 | 45.2101 | 140.5294 |
| Global | AD | Female | 2002 | 121.9677 | 65.2187 | 205.4229 |
| Global | AD | Both | 2002 | 102.6828 | 54.9837 | 173.0625 |
| Global | AD | Male | 2003 | 83.5994 | 45.0385 | 140.5943 |
| Global | AD | Female | 2003 | 121.7209 | 64.9532 | 204.3344 |
| Global | AD | Both | 2003 | 102.5111 | 54.8391 | 172.6206 |
| Global | AD | Male | 2001 | 83.8272 | 45.0350 | 140.7691 |
| Global | AD | Female | 2001 | 122.1835 | 65.2235 | 205.8179 |
| Global | AD | Both | 2001 | 102.8470 | 54.9619 | 173.2033 |
| Global | AD | Male | 2004 | 83.4976 | 44.9498 | 140.0656 |
| Global | AD | Female | 2004 | 121.4919 | 64.9758 | 203.8868 |
| Global | AD | Both | 2004 | 102.3491 | 54.6588 | 172.0290 |
| Global | AD | Male | 2007 | 83.2074 | 44.7437 | 139.9678 |
| Global | AD | Female | 2007 | 120.7912 | 64.4826 | 202.6388 |
| Global | AD | Both | 2007 | 101.8576 | 54.4607 | 171.3572 |
| Global | AD | Male | 2006 | 83.2907 | 44.8987 | 140.1349 |
| Global | AD | Female | 2006 | 121.0107 | 64.4666 | 203.4371 |
| Global | AD | Both | 2006 | 102.0096 | 54.5755 | 171.5930 |
| Global | AD | Male | 2005 | 83.3862 | 44.9238 | 140.1117 |
| Global | AD | Female | 2005 | 121.2433 | 64.8597 | 203.4053 |
| Global | AD | Both | 2005 | 102.1726 | 54.5912 | 171.6622 |
| Global | AD | Male | 2008 | 83.1398 | 44.7793 | 139.8482 |
| Global | AD | Female | 2008 | 120.5680 | 64.2979 | 203.3018 |
| Global | AD | Both | 2008 | 101.7114 | 54.4610 | 171.5495 |
| Global | AD | Male | 2009 | 83.0866 | 44.7596 | 139.6614 |
| Global | AD | Female | 2009 | 120.3764 | 64.2754 | 202.3641 |
| Global | AD | Both | 2009 | 101.5894 | 54.4494 | 171.0920 |
| Global | AD | Male | 2010 | 83.0437 | 44.8151 | 139.6253 |
| Global | AD | Female | 2010 | 120.1884 | 64.1174 | 202.3279 |
| Global | AD | Both | 2010 | 101.4755 | 54.3997 | 171.1972 |
| Global | AD | Male | 2011 | 82.9622 | 44.4897 | 139.6367 |
| Global | AD | Female | 2011 | 119.9546 | 63.8381 | 202.5101 |
| Global | AD | Both | 2011 | 101.3191 | 54.0525 | 170.8260 |
| Global | AD | Male | 2012 | 82.8019 | 44.4180 | 139.4727 |
| Global | AD | Female | 2012 | 119.6505 | 63.6476 | 200.8180 |
| Global | AD | Both | 2012 | 101.0868 | 53.9647 | 170.2401 |
| Global | AD | Male | 2013 | 82.6296 | 44.1944 | 138.9860 |
| Global | AD | Female | 2013 | 119.3074 | 63.4848 | 200.3708 |
| Global | AD | Both | 2013 | 100.8296 | 53.7727 | 169.4363 |
| Global | AD | Male | 2014 | 82.4678 | 44.1651 | 139.2033 |
| Global | AD | Female | 2014 | 118.9595 | 63.2917 | 199.5920 |
| Global | AD | Both | 2014 | 100.5751 | 53.6629 | 169.2160 |
| Global | AD | Male | 2015 | 82.3549 | 44.0299 | 138.5427 |
| Global | AD | Female | 2015 | 118.6832 | 63.3096 | 199.4709 |
| Global | AD | Both | 2015 | 100.3793 | 53.6008 | 168.7494 |
| Global | AD | Male | 2016 | 82.2650 | 43.9407 | 138.6281 |
| Global | AD | Female | 2016 | 118.4229 | 63.1164 | 199.1665 |
| Global | AD | Both | 2016 | 100.2028 | 53.4566 | 168.4888 |
| Global | AD | Male | 2017 | 82.1480 | 43.9286 | 138.2817 |
| Global | AD | Female | 2017 | 118.1112 | 62.7985 | 197.9309 |
| Global | AD | Both | 2017 | 99.9874 | 53.2943 | 167.6512 |
| Global | AD | Male | 2018 | 82.0546 | 43.7471 | 137.8845 |
| Global | AD | Female | 2018 | 117.8370 | 62.5479 | 197.9583 |
| Global | AD | Both | 2018 | 99.8022 | 53.0738 | 167.6737 |
| Global | AD | Male | 2019 | 82.0109 | 43.7200 | 137.7186 |
| Global | AD | Female | 2019 | 117.6519 | 62.6067 | 197.4150 |
| Global | AD | Both | 2019 | 99.6852 | 53.0912 | 167.4329 |
|  |  |  |  |  |  |  |

**Table10 DALYs of AD stratified by sex in 1990~2019, Page 27-37**

| **location_name** | **sex_name** | **cause_name** | **year** | **Age-standardised incidence rate (per 100 000 person-years)** | **95%CI(lower)** | **95%CI(upper)** |
| --- | --- | --- | --- | --- | --- | --- |
| High-middle SDI | Male | AD | 1990 | 266.6719 | 252.6510 | 281.0475 |
| High-middle SDI | Female | AD | 1990 | 440.6767 | 419.3842 | 461.9207 |
| High-middle SDI | Both | AD | 1990 | 352.2975 | 335.5211 | 369.9588 |
| Low SDI | Male | AD | 1990 | 189.4868 | 178.5749 | 201.9551 |
| Low SDI | Female | AD | 1990 | 222.2747 | 210.1642 | 235.8632 |
| Low SDI | Both | AD | 1990 | 205.7307 | 194.4640 | 218.1661 |
| Low-middle SDI | Male | AD | 1991 | 258.6765 | 244.1069 | 274.8460 |
| Low-middle SDI | Female | AD | 1991 | 339.8418 | 322.1264 | 358.4471 |
| Low-middle SDI | Both | AD | 1991 | 298.6373 | 283.2305 | 315.6808 |
| Low-middle SDI | Male | AD | 1990 | 260.6269 | 245.4221 | 276.7242 |
| Low-middle SDI | Female | AD | 1990 | 339.6482 | 321.5116 | 358.6253 |
| Low-middle SDI | Both | AD | 1990 | 299.5397 | 283.7929 | 316.4791 |
| High SDI | Male | AD | 1990 | 321.2784 | 301.3824 | 341.3855 |
| High SDI | Female | AD | 1990 | 499.8202 | 472.8721 | 527.5276 |
| High SDI | Both | AD | 1990 | 409.1284 | 385.7456 | 432.8233 |
| High SDI | Male | AD | 1991 | 320.5063 | 300.4159 | 340.6152 |
| High SDI | Female | AD | 1991 | 498.4436 | 471.5059 | 526.1249 |
| High SDI | Both | AD | 1991 | 408.0183 | 384.8423 | 431.4153 |
| High-middle SDI | Male | AD | 1991 | 265.2675 | 251.5190 | 279.7308 |
| High-middle SDI | Female | AD | 1991 | 440.6123 | 419.5393 | 461.7411 |
| High-middle SDI | Both | AD | 1991 | 351.5092 | 334.9853 | 368.9691 |
| Middle SDI | Male | AD | 1990 | 284.0483 | 271.2118 | 297.5573 |
| Middle SDI | Female | AD | 1990 | 469.6452 | 448.7131 | 490.1584 |
| Middle SDI | Both | AD | 1990 | 375.5650 | 359.5706 | 390.9805 |
| Low SDI | Male | AD | 1991 | 166.6112 | 156.9954 | 177.4351 |
| Low SDI | Female | AD | 1991 | 222.2016 | 210.2441 | 235.6199 |
| Low SDI | Both | AD | 1991 | 194.0943 | 183.5141 | 205.8481 |
| Middle SDI | Male | AD | 1991 | 250.5934 | 239.2549 | 262.3790 |
| Middle SDI | Female | AD | 1991 | 468.9132 | 448.1847 | 489.5503 |
| Middle SDI | Both | AD | 1991 | 358.2533 | 342.9077 | 373.5074 |
| High-middle SDI | Male | AD | 1992 | 265.3158 | 251.6044 | 279.4391 |
| High-middle SDI | Female | AD | 1992 | 440.6830 | 419.7225 | 461.8629 |
| High-middle SDI | Both | AD | 1992 | 351.5357 | 334.7136 | 368.9744 |
| High SDI | Male | AD | 1992 | 319.9896 | 300.0247 | 340.6421 |
| High SDI | Female | AD | 1992 | 497.3496 | 470.3915 | 524.3520 |
| High SDI | Both | AD | 1992 | 407.1814 | 383.9368 | 430.4104 |
| Low-middle SDI | Male | AD | 1992 | 259.1756 | 244.9655 | 275.4227 |
| Low-middle SDI | Female | AD | 1992 | 339.9336 | 322.5504 | 358.1711 |
| Low-middle SDI | Both | AD | 1992 | 298.9440 | 283.7034 | 315.3793 |
| Low SDI | Male | AD | 1992 | 166.7644 | 157.3766 | 177.5688 |
| Low SDI | Female | AD | 1992 | 222.2848 | 210.1509 | 235.5945 |
| Low SDI | Both | AD | 1992 | 194.1988 | 183.6701 | 205.9662 |
| Middle SDI | Male | AD | 1992 | 253.4700 | 242.1923 | 265.4907 |
| Middle SDI | Female | AD | 1992 | 468.1962 | 447.8177 | 488.6054 |
| Middle SDI | Both | AD | 1992 | 359.3199 | 343.9289 | 374.6965 |
| High-middle SDI | Male | AD | 1993 | 265.4943 | 251.7421 | 279.8348 |
| High-middle SDI | Female | AD | 1993 | 440.9700 | 420.0238 | 462.2717 |
| High-middle SDI | Both | AD | 1993 | 351.7512 | 335.1024 | 369.1397 |
| High SDI | Male | AD | 1993 | 319.7603 | 300.5526 | 340.7845 |
| High SDI | Female | AD | 1993 | 496.6333 | 469.6525 | 524.0396 |
| High SDI | Both | AD | 1993 | 406.6825 | 383.8267 | 430.1923 |
| Low SDI | Male | AD | 1993 | 166.8775 | 157.3634 | 177.6739 |
| Low SDI | Female | AD | 1993 | 222.3702 | 210.4514 | 235.5330 |
| Low SDI | Both | AD | 1993 | 194.2843 | 183.7232 | 206.0524 |
| Middle SDI | Male | AD | 1993 | 251.3066 | 239.9658 | 262.8384 |
| Middle SDI | Female | AD | 1993 | 467.5042 | 447.1282 | 487.4141 |
| Middle SDI | Both | AD | 1993 | 357.8382 | 342.2980 | 372.9984 |
| Low-middle SDI | Male | AD | 1993 | 259.4946 | 244.9402 | 275.5627 |
| Low-middle SDI | Female | AD | 1993 | 339.8777 | 322.6637 | 357.7325 |
| Low-middle SDI | Both | AD | 1993 | 299.0898 | 283.5901 | 315.6159 |
| High-middle SDI | Male | AD | 1994 | 265.8447 | 252.1147 | 279.8505 |
| High-middle SDI | Female | AD | 1994 | 441.4775 | 420.5059 | 462.8484 |
| High-middle SDI | Both | AD | 1994 | 352.1728 | 335.3448 | 369.6075 |
| High SDI | Male | AD | 1994 | 319.8513 | 300.6490 | 340.8175 |
| High SDI | Female | AD | 1994 | 496.3041 | 469.0902 | 523.5543 |
| High SDI | Both | AD | 1994 | 406.5401 | 383.4956 | 430.3116 |
| Low-middle SDI | Male | AD | 1994 | 259.6746 | 244.9270 | 275.5837 |
| Low-middle SDI | Female | AD | 1994 | 339.6651 | 322.4986 | 357.2949 |
| Low-middle SDI | Both | AD | 1994 | 299.0930 | 283.3458 | 315.2335 |
| Low SDI | Male | AD | 1994 | 166.7587 | 157.0505 | 177.2725 |
| Low SDI | Female | AD | 1994 | 222.2102 | 210.5155 | 235.3557 |
| Low SDI | Both | AD | 1994 | 194.1403 | 183.6484 | 205.9326 |
| Middle SDI | Male | AD | 1994 | 254.3605 | 242.9284 | 266.1197 |
| Middle SDI | Female | AD | 1994 | 466.8264 | 446.0688 | 486.4099 |
| Middle SDI | Both | AD | 1994 | 359.0397 | 343.5442 | 374.2927 |
| Middle SDI | Male | AD | 1995 | 254.9606 | 243.6522 | 266.5497 |
| Middle SDI | Female | AD | 1995 | 466.1599 | 445.5603 | 485.9413 |
| Middle SDI | Both | AD | 1995 | 359.0161 | 343.4562 | 374.1954 |
| High-middle SDI | Male | AD | 1995 | 266.3536 | 252.5931 | 280.1835 |
| High-middle SDI | Female | AD | 1995 | 442.2275 | 420.9424 | 463.7278 |
| High-middle SDI | Both | AD | 1995 | 352.8034 | 335.8307 | 370.1876 |
| High SDI | Male | AD | 1995 | 320.2945 | 301.3081 | 341.6221 |
| High SDI | Female | AD | 1995 | 496.4269 | 468.8680 | 524.1768 |
| High SDI | Both | AD | 1995 | 406.8018 | 383.5611 | 430.8234 |
| Low-middle SDI | Male | AD | 1995 | 259.6022 | 244.7206 | 275.2414 |
| Low-middle SDI | Female | AD | 1995 | 339.2669 | 321.7410 | 356.8662 |
| Low-middle SDI | Both | AD | 1995 | 298.8780 | 283.1324 | 315.0109 |
| Low SDI | Male | AD | 1995 | 166.4668 | 156.7614 | 176.9037 |
| Low SDI | Female | AD | 1995 | 221.9094 | 210.1955 | 235.1105 |
| Low SDI | Both | AD | 1995 | 193.8433 | 183.4254 | 205.5879 |
| High-middle SDI | Male | AD | 1996 | 267.0331 | 253.4346 | 280.8591 |
| High-middle SDI | Female | AD | 1996 | 443.2587 | 421.8450 | 464.9646 |
| High-middle SDI | Both | AD | 1996 | 353.6537 | 336.6454 | 371.1705 |
| High SDI | Male | AD | 1996 | 321.7616 | 302.6344 | 343.0533 |
| High SDI | Female | AD | 1996 | 497.4303 | 470.3836 | 524.9399 |
| High SDI | Both | AD | 1996 | 408.0186 | 384.9727 | 431.9235 |
| Low SDI | Male | AD | 1996 | 166.1120 | 156.5050 | 176.6017 |
| Low SDI | Female | AD | 1996 | 221.5803 | 209.8770 | 234.6517 |
| Low SDI | Both | AD | 1996 | 193.5012 | 183.1387 | 205.2133 |
| Low-middle SDI | Male | AD | 1996 | 259.3812 | 244.4920 | 275.0024 |
| Low-middle SDI | Female | AD | 1996 | 338.7524 | 321.2881 | 356.3098 |
| Low-middle SDI | Both | AD | 1996 | 298.5305 | 282.8067 | 314.7039 |
| Middle SDI | Male | AD | 1996 | 253.0687 | 241.7720 | 264.6271 |
| Middle SDI | Female | AD | 1996 | 465.4423 | 444.7669 | 485.1260 |
| Middle SDI | Both | AD | 1996 | 357.7142 | 342.1793 | 372.7926 |
| High-middle SDI | Male | AD | 1997 | 267.8379 | 254.3894 | 281.7366 |
| High-middle SDI | Female | AD | 1997 | 444.6032 | 423.0678 | 466.4232 |
| High-middle SDI | Both | AD | 1997 | 354.7244 | 337.6617 | 372.1821 |
| High SDI | Male | AD | 1997 | 324.4912 | 305.2681 | 345.7106 |
| High SDI | Female | AD | 1997 | 499.4563 | 472.3401 | 526.9800 |
| High SDI | Both | AD | 1997 | 410.3888 | 387.5590 | 434.0317 |
| Low-middle SDI | Male | AD | 1997 | 259.1813 | 244.2939 | 274.7811 |
| Low-middle SDI | Female | AD | 1997 | 338.2283 | 320.8311 | 355.7348 |
| Low-middle SDI | Both | AD | 1997 | 298.1900 | 282.4467 | 314.4097 |
| Low SDI | Male | AD | 1997 | 165.8126 | 156.2642 | 176.2502 |
| Low SDI | Female | AD | 1997 | 221.3471 | 209.6523 | 234.2777 |
| Low SDI | Both | AD | 1997 | 193.2356 | 182.9242 | 204.9097 |
| Middle SDI | Male | AD | 1997 | 253.6889 | 242.3484 | 265.1995 |
| Middle SDI | Female | AD | 1997 | 464.6383 | 443.8717 | 484.2217 |
| Middle SDI | Both | AD | 1997 | 357.6678 | 342.1263 | 372.7209 |
| High-middle SDI | Male | AD | 1998 | 268.7496 | 255.4584 | 282.6307 |
| High-middle SDI | Female | AD | 1998 | 446.1776 | 424.7633 | 467.9875 |
| High-middle SDI | Both | AD | 1998 | 355.9673 | 338.9987 | 373.4654 |
| High SDI | Male | AD | 1998 | 327.6461 | 308.1551 | 348.9944 |
| High SDI | Female | AD | 1998 | 501.9179 | 474.7089 | 530.0766 |
| High SDI | Both | AD | 1998 | 413.1963 | 390.2341 | 436.7538 |
| Low-middle SDI | Male | AD | 1998 | 258.9806 | 244.0725 | 274.5674 |
| Low-middle SDI | Female | AD | 1998 | 337.7057 | 320.3790 | 355.1451 |
| Low-middle SDI | Both | AD | 1998 | 297.8510 | 282.0864 | 314.1197 |
| Low SDI | Male | AD | 1998 | 165.5621 | 156.0901 | 175.9542 |
| Low SDI | Female | AD | 1998 | 221.2143 | 209.5238 | 234.0696 |
| Low SDI | Both | AD | 1998 | 193.0458 | 182.7598 | 204.7099 |
| Middle SDI | Male | AD | 1998 | 254.2849 | 242.8764 | 265.7975 |
| Middle SDI | Female | AD | 1998 | 463.8105 | 442.9376 | 483.3804 |
| Middle SDI | Both | AD | 1998 | 357.6123 | 342.0869 | 372.6640 |
| Low SDI | Male | AD | 1999 | 165.3157 | 155.9149 | 175.6443 |
| Low SDI | Female | AD | 1999 | 221.1137 | 209.4232 | 234.0065 |
| Low SDI | Both | AD | 1999 | 192.8751 | 182.6137 | 204.4914 |
| Middle SDI | Male | AD | 1999 | 254.8794 | 243.4682 | 266.5267 |
| Middle SDI | Female | AD | 1999 | 462.9782 | 442.0557 | 482.5372 |
| Middle SDI | Both | AD | 1999 | 357.5626 | 342.0341 | 372.6035 |
| High-middle SDI | Male | AD | 1999 | 269.6937 | 256.3919 | 283.7966 |
| High-middle SDI | Female | AD | 1999 | 447.7827 | 426.4714 | 469.5727 |
| High-middle SDI | Both | AD | 1999 | 357.2397 | 340.2402 | 374.7568 |
| High SDI | Male | AD | 1999 | 330.2960 | 310.3189 | 351.6051 |
| High SDI | Female | AD | 1999 | 504.0490 | 476.7872 | 531.7775 |
| High SDI | Both | AD | 1999 | 415.5942 | 392.4329 | 439.5458 |
| Low-middle SDI | Male | AD | 1999 | 258.7875 | 243.8354 | 274.3674 |
| Low-middle SDI | Female | AD | 1999 | 337.1871 | 319.9293 | 354.6205 |
| Low-middle SDI | Both | AD | 1999 | 297.5169 | 281.7366 | 313.8148 |
| High-middle SDI | Male | AD | 2000 | 270.5706 | 257.2694 | 284.6505 |
| High-middle SDI | Female | AD | 2000 | 449.2553 | 428.0980 | 471.1960 |
| High-middle SDI | Both | AD | 2000 | 358.4097 | 341.3599 | 375.9592 |
| High SDI | Male | AD | 2000 | 331.5127 | 311.3584 | 352.5191 |
| High SDI | Female | AD | 2000 | 505.0709 | 478.0490 | 532.5516 |
| High SDI | Both | AD | 2000 | 416.7119 | 393.0194 | 440.8253 |
| Low-middle SDI | Male | AD | 2000 | 258.5176 | 243.5122 | 274.1058 |
| Low-middle SDI | Female | AD | 2000 | 336.6204 | 319.4382 | 354.0494 |
| Low-middle SDI | Both | AD | 2000 | 297.1189 | 281.3598 | 313.4193 |
| Low SDI | Male | AD | 2000 | 165.0542 | 155.7232 | 175.3343 |
| Low SDI | Female | AD | 2000 | 221.0148 | 209.2817 | 233.9384 |
| Low SDI | Both | AD | 2000 | 192.6977 | 182.4228 | 204.2652 |
| Middle SDI | Male | AD | 2000 | 255.4355 | 244.0479 | 267.1681 |
| Middle SDI | Female | AD | 2000 | 462.0885 | 440.9757 | 481.5799 |
| Middle SDI | Both | AD | 2000 | 357.4692 | 341.9432 | 372.5251 |
| Low SDI | Male | AD | 2001 | 164.7848 | 155.4827 | 175.0415 |
| Low SDI | Female | AD | 2001 | 220.9237 | 209.1286 | 233.8239 |
| Low SDI | Both | AD | 2001 | 192.5183 | 182.2318 | 204.0326 |
| High-middle SDI | Male | AD | 2001 | 271.5099 | 258.3436 | 285.5721 |
| High-middle SDI | Female | AD | 2001 | 450.7591 | 429.5938 | 472.8509 |
| High-middle SDI | Both | AD | 2001 | 359.6070 | 342.4739 | 377.2908 |
| High SDI | Male | AD | 2001 | 330.9452 | 311.0138 | 351.8740 |
| High SDI | Female | AD | 2001 | 504.7435 | 478.0969 | 532.6514 |
| High SDI | Both | AD | 2001 | 416.2534 | 392.8965 | 440.3215 |
| Middle SDI | Male | AD | 2001 | 255.9858 | 244.5811 | 267.5133 |
| Middle SDI | Female | AD | 2001 | 461.2240 | 440.4285 | 480.4305 |
| Middle SDI | Both | AD | 2001 | 357.3826 | 341.8520 | 372.4690 |
| Low-middle SDI | Male | AD | 2001 | 258.2646 | 243.3246 | 273.8151 |
| Low-middle SDI | Female | AD | 2001 | 336.0431 | 318.9526 | 353.3426 |
| Low-middle SDI | Both | AD | 2001 | 296.7243 | 280.9881 | 313.0608 |
| High-middle SDI | Male | AD | 2002 | 272.4653 | 259.2691 | 286.6800 |
| High-middle SDI | Female | AD | 2002 | 452.2674 | 430.9305 | 473.9365 |
| High-middle SDI | Both | AD | 2002 | 360.7955 | 343.7704 | 378.6491 |
| High SDI | Male | AD | 2002 | 329.1867 | 309.0903 | 350.1586 |
| High SDI | Female | AD | 2002 | 503.5046 | 476.6708 | 531.6995 |
| High SDI | Both | AD | 2002 | 414.7379 | 391.5973 | 438.7036 |
| Low-middle SDI | Male | AD | 2002 | 258.0336 | 243.1640 | 273.5696 |
| Low-middle SDI | Female | AD | 2002 | 335.4864 | 318.4431 | 352.7569 |
| Low-middle SDI | Both | AD | 2002 | 296.3502 | 280.6061 | 312.6599 |
| Low SDI | Male | AD | 2002 | 164.5233 | 155.2529 | 174.7586 |
| Low SDI | Female | AD | 2002 | 220.8596 | 209.0214 | 233.8419 |
| Low SDI | Both | AD | 2002 | 192.3550 | 182.0579 | 203.8238 |
| Middle SDI | Male | AD | 2002 | 259.0842 | 247.6334 | 270.8023 |
| Middle SDI | Female | AD | 2002 | 460.4031 | 440.0223 | 479.4903 |
| Middle SDI | Both | AD | 2002 | 358.6114 | 343.1751 | 373.7661 |
| Low SDI | Male | AD | 2003 | 164.2578 | 155.0181 | 174.4702 |
| Low SDI | Female | AD | 2003 | 220.8040 | 208.9452 | 233.8696 |
| Low SDI | Both | AD | 2003 | 192.1971 | 181.9362 | 203.6339 |
| Middle SDI | Male | AD | 2003 | 259.5675 | 248.0869 | 271.6236 |
| Middle SDI | Female | AD | 2003 | 459.5913 | 439.0726 | 478.8613 |
| Middle SDI | Both | AD | 2003 | 358.4999 | 343.1189 | 373.6554 |
| High-middle SDI | Male | AD | 2003 | 273.4359 | 260.1996 | 287.5087 |
| High-middle SDI | Female | AD | 2003 | 453.7871 | 432.4542 | 475.4032 |
| High-middle SDI | Both | AD | 2003 | 361.9890 | 345.1308 | 379.7613 |
| High SDI | Male | AD | 2003 | 326.6719 | 306.5463 | 347.6098 |
| High SDI | Female | AD | 2003 | 501.6244 | 474.5605 | 529.7795 |
| High SDI | Both | AD | 2003 | 412.5225 | 389.3040 | 436.3613 |
| Low-middle SDI | Male | AD | 2003 | 257.7763 | 242.9467 | 273.3136 |
| Low-middle SDI | Female | AD | 2003 | 334.9264 | 317.8398 | 352.1054 |
| Low-middle SDI | Both | AD | 2003 | 295.9612 | 280.2218 | 312.2142 |
| High-middle SDI | Male | AD | 2004 | 274.4362 | 260.9682 | 288.8014 |
| High-middle SDI | Female | AD | 2004 | 455.4068 | 434.2003 | 477.0684 |
| High-middle SDI | Both | AD | 2004 | 363.2404 | 346.2969 | 380.7357 |
| High SDI | Male | AD | 2004 | 323.9544 | 303.6362 | 344.5162 |
| High SDI | Female | AD | 2004 | 499.5193 | 472.7465 | 527.5665 |
| High SDI | Both | AD | 2004 | 410.0900 | 386.6460 | 433.8731 |
| Low-middle SDI | Male | AD | 2004 | 257.4883 | 242.6639 | 273.0235 |
| Low-middle SDI | Female | AD | 2004 | 334.3341 | 317.1110 | 351.6112 |
| Low-middle SDI | Both | AD | 2004 | 295.5408 | 279.8135 | 311.7240 |
| Low SDI | Male | AD | 2004 | 163.9937 | 154.7736 | 174.1819 |
| Low SDI | Female | AD | 2004 | 220.7457 | 208.8681 | 233.8238 |
| Low SDI | Both | AD | 2004 | 192.0408 | 181.7795 | 203.4458 |
| Middle SDI | Male | AD | 2004 | 259.9886 | 248.4905 | 272.2218 |
| Middle SDI | Female | AD | 2004 | 458.7631 | 438.2515 | 478.1876 |
| Middle SDI | Both | AD | 2004 | 358.3422 | 342.8031 | 373.7028 |
| Low-middle SDI | Male | AD | 2005 | 257.1378 | 242.4484 | 272.6714 |
| Low-middle SDI | Female | AD | 2005 | 333.6931 | 316.4983 | 350.9232 |
| Low-middle SDI | Both | AD | 2005 | 295.0647 | 279.3532 | 311.1521 |
| High-middle SDI | Male | AD | 2005 | 275.3293 | 261.7299 | 289.6145 |
| High-middle SDI | Female | AD | 2005 | 456.9781 | 435.7462 | 478.8724 |
| High-middle SDI | Both | AD | 2005 | 364.4091 | 347.3629 | 382.2174 |
| Low SDI | Male | AD | 2005 | 163.7230 | 154.5246 | 173.8810 |
| Low SDI | Female | AD | 2005 | 220.6641 | 208.8274 | 233.7241 |
| Low SDI | Both | AD | 2005 | 191.8712 | 181.5875 | 203.2700 |
| Middle SDI | Male | AD | 2005 | 257.7549 | 246.2814 | 270.1533 |
| Middle SDI | Female | AD | 2005 | 457.8943 | 437.3391 | 477.5939 |
| Middle SDI | Both | AD | 2005 | 356.8075 | 341.2840 | 372.1376 |
| High SDI | Male | AD | 2005 | 321.5301 | 301.0629 | 341.8534 |
| High SDI | Female | AD | 2005 | 497.4908 | 471.0319 | 526.2282 |
| High SDI | Both | AD | 2005 | 407.8424 | 384.2380 | 431.6896 |
| High-middle SDI | Male | AD | 2006 | 276.0503 | 262.3739 | 290.2572 |
| High-middle SDI | Female | AD | 2006 | 458.4223 | 437.0963 | 480.4687 |
| High-middle SDI | Both | AD | 2006 | 365.4178 | 348.1839 | 383.3066 |
| High SDI | Male | AD | 2006 | 319.0005 | 298.8727 | 339.4503 |
| High SDI | Female | AD | 2006 | 494.8709 | 468.2715 | 523.2929 |
| High SDI | Both | AD | 2006 | 405.2295 | 381.8618 | 428.8663 |
| Low-middle SDI | Male | AD | 2006 | 256.7796 | 241.9756 | 272.3242 |
| Low-middle SDI | Female | AD | 2006 | 333.0763 | 316.0328 | 350.0531 |
| Low-middle SDI | Both | AD | 2006 | 294.5963 | 278.9505 | 310.4817 |
| Low SDI | Male | AD | 2006 | 163.4367 | 154.2606 | 173.5673 |
| Low SDI | Female | AD | 2006 | 220.5294 | 208.7268 | 233.5120 |
| Low SDI | Both | AD | 2006 | 191.6694 | 181.4195 | 203.1107 |
| Middle SDI | Male | AD | 2006 | 258.0359 | 246.7065 | 270.2596 |
| Middle SDI | Female | AD | 2006 | 457.1726 | 436.9004 | 476.6482 |
| Middle SDI | Both | AD | 2006 | 356.6165 | 341.0648 | 372.0257 |
| Low-middle SDI | Male | AD | 2007 | 256.4158 | 241.5273 | 271.9127 |
| Low-middle SDI | Female | AD | 2007 | 332.4936 | 315.4412 | 349.4344 |
| Low-middle SDI | Both | AD | 2007 | 294.1423 | 278.5689 | 310.0847 |
| Low SDI | Male | AD | 2007 | 163.1384 | 153.9788 | 173.2467 |
| Low SDI | Female | AD | 2007 | 220.3447 | 208.6351 | 233.2445 |
| Low SDI | Both | AD | 2007 | 191.4376 | 181.2852 | 202.9136 |
| High-middle SDI | Male | AD | 2007 | 276.5771 | 262.8583 | 290.7585 |
| High-middle SDI | Female | AD | 2007 | 459.7268 | 438.1436 | 481.7631 |
| High-middle SDI | Both | AD | 2007 | 366.2530 | 348.8481 | 384.2303 |
| High SDI | Male | AD | 2007 | 316.0567 | 296.2658 | 336.4624 |
| High SDI | Female | AD | 2007 | 491.3916 | 464.7930 | 519.4350 |
| High SDI | Both | AD | 2007 | 401.9501 | 378.7518 | 425.3869 |
| Middle SDI | Male | AD | 2007 | 260.8014 | 249.2501 | 272.7939 |
| Middle SDI | Female | AD | 2007 | 456.6299 | 436.1505 | 476.2594 |
| Middle SDI | Both | AD | 2007 | 357.7715 | 342.1609 | 373.3174 |
| High-middle SDI | Male | AD | 2008 | 277.0582 | 263.3480 | 291.4863 |
| High-middle SDI | Female | AD | 2008 | 461.0186 | 439.5293 | 483.3795 |
| High-middle SDI | Both | AD | 2008 | 367.0622 | 349.5471 | 384.9113 |
| High SDI | Male | AD | 2008 | 313.2385 | 293.7578 | 333.6991 |
| High SDI | Female | AD | 2008 | 487.8637 | 461.3824 | 515.5278 |
| High SDI | Both | AD | 2008 | 398.6815 | 375.7766 | 421.9938 |
| Low-middle SDI | Male | AD | 2008 | 256.0247 | 241.2520 | 271.3468 |
| Low-middle SDI | Female | AD | 2008 | 331.9100 | 314.9223 | 348.9329 |
| Low-middle SDI | Both | AD | 2008 | 293.6761 | 278.1473 | 309.5914 |
| Low SDI | Male | AD | 2008 | 165.4941 | 156.1914 | 175.8479 |
| Low SDI | Female | AD | 2008 | 220.1263 | 208.5675 | 232.9413 |
| Low SDI | Both | AD | 2008 | 192.5339 | 182.3397 | 204.1109 |
| Middle SDI | Male | AD | 2008 | 260.9289 | 249.4675 | 273.1954 |
| Middle SDI | Female | AD | 2008 | 455.9789 | 435.4070 | 475.6704 |
| Middle SDI | Both | AD | 2008 | 357.5376 | 341.8499 | 372.8221 |
| Low-middle SDI | Male | AD | 2009 | 255.6075 | 240.7630 | 270.7595 |
| Low-middle SDI | Female | AD | 2009 | 331.3029 | 314.4348 | 348.2764 |
| Low-middle SDI | Both | AD | 2009 | 293.1862 | 277.6926 | 308.8325 |
| Low SDI | Male | AD | 2009 | 165.1847 | 155.8856 | 175.5112 |
| Low SDI | Female | AD | 2009 | 219.8957 | 208.3794 | 232.6672 |
| Low SDI | Both | AD | 2009 | 192.2745 | 182.0884 | 203.8748 |
| Middle SDI | Male | AD | 2009 | 261.2519 | 249.6095 | 273.8343 |
| Middle SDI | Female | AD | 2009 | 455.1371 | 434.5848 | 474.5202 |
| Middle SDI | Both | AD | 2009 | 357.3226 | 341.5104 | 372.7755 |
| High-middle SDI | Male | AD | 2009 | 277.5695 | 263.8046 | 292.1540 |
| High-middle SDI | Female | AD | 2009 | 462.3236 | 440.7249 | 484.9872 |
| High-middle SDI | Both | AD | 2009 | 367.8962 | 350.2974 | 385.9888 |
| High SDI | Male | AD | 2009 | 311.0782 | 291.7281 | 331.2064 |
| High SDI | Female | AD | 2009 | 485.0997 | 459.1200 | 512.4911 |
| High SDI | Both | AD | 2009 | 396.1432 | 373.3360 | 419.1963 |
| Low SDI | Male | AD | 2011 | 164.3859 | 155.0687 | 174.9324 |
| Low SDI | Female | AD | 2011 | 219.3933 | 207.8643 | 232.2140 |
| Low SDI | Both | AD | 2011 | 191.6446 | 181.4341 | 203.1145 |
| Middle SDI | Male | AD | 2011 | 261.2165 | 249.5664 | 274.0091 |
| Middle SDI | Female | AD | 2011 | 453.4067 | 432.8542 | 472.5078 |
| Middle SDI | Both | AD | 2011 | 356.5024 | 340.5818 | 372.3034 |
| Low-middle SDI | Male | AD | 2011 | 254.4620 | 239.7171 | 269.9739 |
| Low-middle SDI | Female | AD | 2011 | 329.8611 | 313.1354 | 346.9929 |
| Low-middle SDI | Both | AD | 2011 | 291.9311 | 276.4762 | 307.9182 |
| High-middle SDI | Male | AD | 2011 | 278.7521 | 264.7437 | 293.5632 |
| High-middle SDI | Female | AD | 2011 | 464.8863 | 442.9857 | 487.7283 |
| High-middle SDI | Both | AD | 2011 | 369.6026 | 352.1397 | 387.6355 |
| High SDI | Male | AD | 2011 | 309.8737 | 290.9488 | 329.8390 |
| High SDI | Female | AD | 2011 | 483.7137 | 457.9045 | 510.2643 |
| High SDI | Both | AD | 2011 | 394.8038 | 372.3522 | 417.8163 |
| High-middle SDI | Male | AD | 2010 | 278.1661 | 264.0997 | 293.0368 |
| High-middle SDI | Female | AD | 2010 | 463.6582 | 441.8030 | 486.5675 |
| High-middle SDI | Both | AD | 2010 | 368.7845 | 351.2347 | 386.8791 |
| High SDI | Male | AD | 2010 | 310.1026 | 290.7143 | 330.3890 |
| High SDI | Female | AD | 2010 | 483.9418 | 457.9399 | 511.0938 |
| High SDI | Both | AD | 2010 | 395.0536 | 372.2201 | 417.9399 |
| Low-middle SDI | Male | AD | 2010 | 255.1544 | 240.2388 | 270.1346 |
| Low-middle SDI | Female | AD | 2010 | 330.6503 | 313.8946 | 347.6791 |
| Low-middle SDI | Both | AD | 2010 | 292.6547 | 277.1932 | 308.2247 |
| Low SDI | Male | AD | 2010 | 164.8446 | 155.5528 | 175.1417 |
| Low SDI | Female | AD | 2010 | 219.6710 | 208.1687 | 232.3877 |
| Low SDI | Both | AD | 2010 | 192.0029 | 181.8282 | 203.6221 |
| Middle SDI | Male | AD | 2010 | 260.9705 | 249.3055 | 273.8911 |
| Middle SDI | Female | AD | 2010 | 454.2578 | 433.7100 | 473.6084 |
| Middle SDI | Both | AD | 2010 | 356.7730 | 340.8808 | 372.2741 |
| High-middle SDI | Male | AD | 2012 | 279.0600 | 265.0448 | 293.8733 |
| High-middle SDI | Female | AD | 2012 | 465.6545 | 443.6223 | 488.4075 |
| High-middle SDI | Both | AD | 2012 | 370.0435 | 352.6050 | 388.0049 |
| High SDI | Male | AD | 2012 | 309.5407 | 290.5745 | 329.2746 |
| High SDI | Female | AD | 2012 | 483.1710 | 457.2654 | 509.7715 |
| High SDI | Both | AD | 2012 | 394.3406 | 372.3320 | 417.3665 |
| Low-middle SDI | Male | AD | 2012 | 253.4540 | 239.2005 | 269.0960 |
| Low-middle SDI | Female | AD | 2012 | 328.9127 | 312.2929 | 346.3014 |
| Low-middle SDI | Both | AD | 2012 | 290.9661 | 275.6814 | 306.9064 |
| Low SDI | Male | AD | 2012 | 163.7853 | 154.5065 | 174.2461 |
| Low SDI | Female | AD | 2012 | 219.0366 | 207.4149 | 231.7782 |
| Low SDI | Both | AD | 2012 | 191.1743 | 180.9683 | 202.7852 |
| Middle SDI | Male | AD | 2012 | 260.7568 | 249.0973 | 273.4778 |
| Middle SDI | Female | AD | 2012 | 452.5645 | 432.0188 | 471.5952 |
| Middle SDI | Both | AD | 2012 | 355.8747 | 339.9784 | 371.6106 |
| High-middle SDI | Male | AD | 2013 | 279.1379 | 265.0978 | 294.2592 |
| High-middle SDI | Female | AD | 2013 | 465.9013 | 443.9448 | 488.5380 |
| High-middle SDI | Both | AD | 2013 | 370.1248 | 352.6692 | 387.9919 |
| High SDI | Male | AD | 2013 | 309.1589 | 290.7454 | 328.6709 |
| High SDI | Female | AD | 2013 | 482.4312 | 456.6369 | 509.4760 |
| High SDI | Both | AD | 2013 | 393.7521 | 372.0363 | 416.6609 |
| Low-middle SDI | Male | AD | 2013 | 252.3382 | 238.4661 | 267.6623 |
| Low-middle SDI | Female | AD | 2013 | 327.8933 | 311.0520 | 345.4846 |
| Low-middle SDI | Both | AD | 2013 | 289.9104 | 274.8339 | 305.8954 |
| Low SDI | Male | AD | 2013 | 163.1195 | 154.0852 | 173.4130 |
| Low SDI | Female | AD | 2013 | 218.6464 | 206.9532 | 231.3149 |
| Low SDI | Both | AD | 2013 | 190.6539 | 180.4497 | 202.1957 |
| Middle SDI | Male | AD | 2013 | 260.4813 | 248.6045 | 273.0742 |
| Middle SDI | Female | AD | 2013 | 451.6515 | 431.0772 | 470.6953 |
| Middle SDI | Both | AD | 2013 | 355.2998 | 339.4235 | 370.8390 |
| High SDI | Male | AD | 2015 | 308.4756 | 289.9453 | 327.3361 |
| High SDI | Female | AD | 2015 | 481.0709 | 455.8669 | 507.6196 |
| High SDI | Both | AD | 2015 | 392.6761 | 371.2835 | 415.2930 |
| Low-middle SDI | Male | AD | 2015 | 250.5475 | 236.0485 | 265.6736 |
| Low-middle SDI | Female | AD | 2015 | 326.0130 | 308.4867 | 343.7884 |
| Low-middle SDI | Both | AD | 2015 | 288.0965 | 272.9599 | 304.1918 |
| Low SDI | Male | AD | 2015 | 161.9094 | 152.7364 | 172.2226 |
| Low SDI | Female | AD | 2015 | 217.9424 | 206.3544 | 230.7933 |
| Low SDI | Both | AD | 2015 | 189.7104 | 179.4757 | 200.9909 |
| Middle SDI | Male | AD | 2015 | 259.9594 | 248.1008 | 272.3764 |
| Middle SDI | Female | AD | 2015 | 449.6733 | 428.7342 | 469.0693 |
| Middle SDI | Both | AD | 2015 | 354.0591 | 338.4042 | 369.6233 |
| High-middle SDI | Male | AD | 2015 | 279.1267 | 264.9380 | 294.2185 |
| High-middle SDI | Female | AD | 2015 | 465.7940 | 443.6958 | 488.6480 |
| High-middle SDI | Both | AD | 2015 | 369.9289 | 352.0108 | 387.8896 |
| High-middle SDI | Male | AD | 2014 | 279.1181 | 264.9990 | 294.3199 |
| High-middle SDI | Female | AD | 2014 | 465.8483 | 443.9131 | 488.4468 |
| High-middle SDI | Both | AD | 2014 | 370.0199 | 352.3822 | 387.8769 |
| High SDI | Male | AD | 2014 | 308.8082 | 290.3638 | 327.8682 |
| High SDI | Female | AD | 2014 | 481.7042 | 455.8441 | 508.7551 |
| High SDI | Both | AD | 2014 | 393.1863 | 371.5274 | 416.0948 |
| Low-middle SDI | Male | AD | 2014 | 251.3163 | 237.2382 | 266.6351 |
| Low-middle SDI | Female | AD | 2014 | 326.8977 | 309.7277 | 344.6538 |
| Low-middle SDI | Both | AD | 2014 | 288.9129 | 273.7227 | 304.9426 |
| Low SDI | Male | AD | 2014 | 162.4697 | 153.5673 | 172.6552 |
| Low SDI | Female | AD | 2014 | 218.2693 | 206.7526 | 231.0345 |
| Low SDI | Both | AD | 2014 | 190.1475 | 179.9431 | 201.3718 |
| Middle SDI | Male | AD | 2014 | 260.1884 | 248.2728 | 272.6475 |
| Middle SDI | Female | AD | 2014 | 450.6672 | 429.9271 | 469.8076 |
| Middle SDI | Both | AD | 2014 | 354.6697 | 338.9447 | 370.1715 |
| High-middle SDI | Male | AD | 2016 | 278.8642 | 264.6396 | 293.8254 |
| High-middle SDI | Female | AD | 2016 | 465.2136 | 443.0666 | 487.7769 |
| High-middle SDI | Both | AD | 2016 | 369.4346 | 351.6231 | 387.3271 |
| High SDI | Male | AD | 2016 | 308.2459 | 289.6741 | 327.0922 |
| High SDI | Female | AD | 2016 | 480.7848 | 455.7505 | 507.1969 |
| High SDI | Both | AD | 2016 | 392.3891 | 370.7359 | 414.8600 |
| Low-middle SDI | Male | AD | 2016 | 249.9642 | 235.5010 | 265.0080 |
| Low-middle SDI | Female | AD | 2016 | 325.2074 | 307.7042 | 342.9299 |
| Low-middle SDI | Both | AD | 2016 | 287.4129 | 272.3348 | 303.4476 |
| Low SDI | Male | AD | 2016 | 161.5142 | 152.4131 | 171.7233 |
| Low SDI | Female | AD | 2016 | 217.7654 | 206.1689 | 230.5852 |
| Low SDI | Both | AD | 2016 | 189.4288 | 179.2926 | 200.7515 |
| Middle SDI | Male | AD | 2016 | 259.7749 | 247.9373 | 272.2116 |
| Middle SDI | Female | AD | 2016 | 448.7037 | 427.7135 | 468.2090 |
| Middle SDI | Both | AD | 2016 | 353.4717 | 337.8997 | 369.0466 |
| High SDI | Male | AD | 2017 | 308.0585 | 289.4126 | 327.7096 |
| High SDI | Female | AD | 2017 | 480.6381 | 454.9738 | 507.0726 |
| High SDI | Both | AD | 2017 | 392.1946 | 370.3730 | 414.7575 |
| Low-middle SDI | Male | AD | 2017 | 249.3675 | 234.9340 | 264.3498 |
| Low-middle SDI | Female | AD | 2017 | 324.3682 | 306.9197 | 342.0373 |
| Low-middle SDI | Both | AD | 2017 | 286.7059 | 271.6516 | 302.7008 |
| High-middle SDI | Male | AD | 2017 | 278.3121 | 264.0840 | 293.0913 |
| High-middle SDI | Female | AD | 2017 | 464.0293 | 441.9447 | 486.4275 |
| High-middle SDI | Both | AD | 2017 | 368.5030 | 350.8628 | 386.3803 |
| Low SDI | Male | AD | 2017 | 161.0857 | 151.8573 | 171.2990 |
| Low SDI | Female | AD | 2017 | 217.5478 | 205.9874 | 230.3719 |
| Low SDI | Both | AD | 2017 | 189.1079 | 178.9835 | 200.2858 |
| Middle SDI | Male | AD | 2017 | 259.7412 | 247.8054 | 272.1880 |
| Middle SDI | Female | AD | 2017 | 447.5412 | 426.6662 | 467.1351 |
| Middle SDI | Both | AD | 2017 | 352.8575 | 337.2798 | 368.5060 |
| Low SDI | Male | AD | 2018 | 160.5266 | 151.3704 | 170.6530 |
| Low SDI | Female | AD | 2018 | 217.1789 | 205.6734 | 229.9599 |
| Low SDI | Both | AD | 2018 | 188.6452 | 178.5390 | 199.7575 |
| High SDI | Male | AD | 2018 | 308.3197 | 289.9341 | 328.3205 |
| High SDI | Female | AD | 2018 | 481.6448 | 455.6244 | 508.2051 |
| High SDI | Both | AD | 2018 | 392.7945 | 370.8672 | 415.3535 |
| Low-middle SDI | Male | AD | 2018 | 248.8004 | 234.3455 | 263.8870 |
| Low-middle SDI | Female | AD | 2018 | 323.5698 | 306.1547 | 341.2562 |
| Low-middle SDI | Both | AD | 2018 | 286.0304 | 270.9472 | 302.0252 |
| Middle SDI | Male | AD | 2018 | 259.6395 | 247.7067 | 272.1576 |
| Middle SDI | Female | AD | 2018 | 446.5698 | 426.4031 | 465.3219 |
| Middle SDI | Both | AD | 2018 | 352.2998 | 336.7901 | 367.7133 |
| High-middle SDI | Male | AD | 2018 | 278.3213 | 264.4293 | 293.1252 |
| High-middle SDI | Female | AD | 2018 | 463.3597 | 441.7269 | 485.4131 |
| High-middle SDI | Both | AD | 2018 | 368.1235 | 350.8453 | 385.8887 |
| High SDI | Male | AD | 2019 | 309.1893 | 289.6693 | 329.9791 |
| High SDI | Female | AD | 2019 | 484.1783 | 457.7784 | 512.1446 |
| High SDI | Both | AD | 2019 | 394.4542 | 371.5985 | 417.3112 |
| Low-middle SDI | Male | AD | 2019 | 248.2918 | 233.8096 | 263.5387 |
| Low-middle SDI | Female | AD | 2019 | 322.8606 | 305.4998 | 340.9221 |
| Low-middle SDI | Both | AD | 2019 | 285.4254 | 270.3178 | 301.3075 |
| Low SDI | Male | AD | 2019 | 159.9655 | 150.8988 | 170.0096 |
| Low SDI | Female | AD | 2019 | 216.8113 | 205.3695 | 229.5318 |
| Low SDI | Both | AD | 2019 | 188.1815 | 178.0950 | 199.2461 |
| High-middle SDI | Male | AD | 2019 | 279.0329 | 264.8316 | 293.5968 |
| High-middle SDI | Female | AD | 2019 | 463.4600 | 441.5803 | 485.2813 |
| High-middle SDI | Both | AD | 2019 | 368.4836 | 351.0207 | 386.7380 |
| Middle SDI | Male | AD | 2019 | 259.9803 | 248.1490 | 272.5343 |
| Middle SDI | Female | AD | 2019 | 446.0551 | 425.7193 | 464.7858 |
| Middle SDI | Both | AD | 2019 | 352.1898 | 336.4264 | 367.4982 |
|  |  |  |  |  |  |  |

**Table11 DALYs of AD stratified by SDI in 1990~2019, Page 38-48**

| **location_name** | **sex_name** | **cause_name** | **year** | **Age-standardised DALYs rate (per 100 000 person-years)** | **95%(lower)** | **95%(upper)** |
| --- | --- | --- | --- | --- | --- | --- |
| High-middle SDI | Male | AD | 1990 | 91.8325 | 49.1399 | 154.7714 |
| High-middle SDI | Female | AD | 1990 | 139.4014 | 73.7746 | 235.2552 |
| High-middle SDI | Both | AD | 1990 | 115.3627 | 61.2870 | 194.0445 |
| High SDI | Male | AD | 1990 | 135.2475 | 72.3893 | 228.6431 |
| High SDI | Female | AD | 1990 | 188.9878 | 101.3340 | 316.1332 |
| High SDI | Both | AD | 1990 | 161.7520 | 86.6560 | 272.4654 |
| Low-middle SDI | Male | AD | 1990 | 75.0453 | 40.0443 | 125.5224 |
| Low-middle SDI | Female | AD | 1990 | 96.1913 | 51.4049 | 160.4714 |
| Low-middle SDI | Both | AD | 1990 | 85.4521 | 45.6966 | 142.5232 |
| Middle SDI | Male | AD | 1990 | 79.1386 | 42.4901 | 132.3130 |
| Middle SDI | Female | AD | 1990 | 129.3402 | 68.8542 | 216.4033 |
| Middle SDI | Both | AD | 1990 | 103.8815 | 55.4667 | 173.5997 |
| Low SDI | Male | AD | 1990 | 56.2466 | 30.5120 | 94.8664 |
| Low SDI | Female | AD | 1990 | 65.2079 | 35.1038 | 108.7454 |
| Low SDI | Both | AD | 1990 | 60.6871 | 32.6904 | 101.7106 |
| Low SDI | Male | AD | 1991 | 56.2707 | 30.2818 | 94.5548 |
| Low SDI | Female | AD | 1991 | 65.2186 | 35.1039 | 108.8116 |
| Low SDI | Both | AD | 1991 | 60.7032 | 32.6030 | 101.4776 |
| Low-middle SDI | Male | AD | 1991 | 75.2396 | 40.5063 | 126.3157 |
| Low-middle SDI | Female | AD | 1991 | 96.3740 | 51.5072 | 160.9463 |
| Low-middle SDI | Both | AD | 1991 | 85.6408 | 45.9504 | 143.1758 |
| High-middle SDI | Male | AD | 1991 | 91.6694 | 48.8648 | 154.6351 |
| High-middle SDI | Female | AD | 1991 | 139.2827 | 74.3399 | 234.1902 |
| High-middle SDI | Both | AD | 1991 | 115.2081 | 61.4687 | 193.9157 |
| High SDI | Male | AD | 1991 | 135.0774 | 72.0111 | 226.8348 |
| High SDI | Female | AD | 1991 | 188.7421 | 100.6904 | 317.2505 |
| High SDI | Both | AD | 1991 | 161.5308 | 86.1458 | 271.6827 |
| Middle SDI | Male | AD | 1991 | 79.1016 | 42.1577 | 132.2279 |
| Middle SDI | Female | AD | 1991 | 129.2465 | 68.8004 | 216.2255 |
| Middle SDI | Both | AD | 1991 | 103.8111 | 55.2806 | 173.6032 |
| High-middle SDI | Male | AD | 1992 | 91.4870 | 48.7956 | 154.1410 |
| High-middle SDI | Female | AD | 1992 | 139.1489 | 74.1326 | 235.0134 |
| High-middle SDI | Both | AD | 1992 | 115.0412 | 61.2392 | 193.8002 |
| High SDI | Male | AD | 1992 | 134.9603 | 71.8599 | 227.0686 |
| High SDI | Female | AD | 1992 | 188.5470 | 100.8403 | 317.0059 |
| High SDI | Both | AD | 1992 | 161.3640 | 86.1396 | 271.9079 |
| Low-middle SDI | Male | AD | 1992 | 75.4263 | 40.5726 | 126.0879 |
| Low-middle SDI | Female | AD | 1992 | 96.5155 | 51.6028 | 160.9157 |
| Low-middle SDI | Both | AD | 1992 | 85.8061 | 45.9649 | 143.2072 |
| Low SDI | Male | AD | 1992 | 56.2947 | 30.2096 | 94.1918 |
| Low SDI | Female | AD | 1992 | 65.3007 | 34.9664 | 108.7640 |
| Low SDI | Both | AD | 1992 | 60.7537 | 32.5075 | 101.0592 |
| High-middle SDI | Male | AD | 1993 | 91.2379 | 48.6792 | 152.9681 |
| High-middle SDI | Female | AD | 1993 | 138.9747 | 74.3776 | 234.1092 |
| High-middle SDI | Both | AD | 1993 | 114.8238 | 61.0417 | 193.0789 |
| High SDI | Male | AD | 1993 | 134.9480 | 72.2446 | 226.8294 |
| High SDI | Female | AD | 1993 | 188.3310 | 100.7245 | 317.3419 |
| High SDI | Both | AD | 1993 | 161.2418 | 86.2693 | 271.8677 |
| Low-middle SDI | Male | AD | 1993 | 75.6025 | 40.9653 | 125.9687 |
| Low-middle SDI | Female | AD | 1993 | 96.6474 | 51.5075 | 161.1210 |
| Low-middle SDI | Both | AD | 1993 | 85.9631 | 46.1340 | 143.7682 |
| Low SDI | Male | AD | 1993 | 56.3644 | 30.2620 | 94.4313 |
| Low SDI | Female | AD | 1993 | 65.3617 | 34.9076 | 109.1952 |
| Low SDI | Both | AD | 1993 | 60.8171 | 32.6580 | 101.8835 |
| Middle SDI | Male | AD | 1992 | 79.0865 | 42.2575 | 132.3050 |
| Middle SDI | Female | AD | 1992 | 129.2188 | 68.7419 | 217.6889 |
| Middle SDI | Both | AD | 1992 | 103.7846 | 55.3107 | 174.5899 |
| Middle SDI | Male | AD | 1993 | 79.0759 | 42.2964 | 132.7263 |
| Middle SDI | Female | AD | 1993 | 129.1445 | 68.8748 | 217.1436 |
| Middle SDI | Both | AD | 1993 | 103.7393 | 55.3950 | 174.2361 |
| Middle SDI | Male | AD | 1994 | 79.1262 | 42.2076 | 132.4164 |
| Middle SDI | Female | AD | 1994 | 129.0622 | 69.0439 | 216.3461 |
| Middle SDI | Both | AD | 1994 | 103.7248 | 55.4060 | 174.1575 |
| High-middle SDI | Male | AD | 1994 | 90.9660 | 48.4372 | 154.2143 |
| High-middle SDI | Female | AD | 1994 | 138.8156 | 73.6710 | 233.7153 |
| High-middle SDI | Both | AD | 1994 | 114.6052 | 60.9015 | 192.9630 |
| High SDI | Male | AD | 1994 | 134.9104 | 71.8275 | 228.6189 |
| High SDI | Female | AD | 1994 | 188.2334 | 100.8543 | 316.4978 |
| High SDI | Both | AD | 1994 | 161.1661 | 86.0603 | 271.7312 |
| Low-middle SDI | Male | AD | 1994 | 75.7251 | 40.8048 | 125.5790 |
| Low-middle SDI | Female | AD | 1994 | 96.7287 | 52.0309 | 162.3388 |
| Low-middle SDI | Both | AD | 1994 | 86.0695 | 46.3298 | 144.3093 |
| Low SDI | Male | AD | 1994 | 56.3531 | 30.3168 | 94.7508 |
| Low SDI | Female | AD | 1994 | 65.3663 | 35.0403 | 109.7334 |
| Low SDI | Both | AD | 1994 | 60.8129 | 32.7867 | 102.0220 |
| Middle SDI | Male | AD | 1995 | 79.1283 | 42.1245 | 132.7267 |
| Middle SDI | Female | AD | 1995 | 128.9844 | 69.2672 | 216.4698 |
| Middle SDI | Both | AD | 1995 | 103.6918 | 55.4036 | 174.1137 |
| High-middle SDI | Male | AD | 1995 | 90.7732 | 48.4438 | 153.7868 |
| High-middle SDI | Female | AD | 1995 | 138.6912 | 73.6752 | 233.8890 |
| High-middle SDI | Both | AD | 1995 | 114.4464 | 60.8549 | 192.7124 |
| High SDI | Male | AD | 1995 | 135.0027 | 71.8388 | 228.6428 |
| High SDI | Female | AD | 1995 | 188.1323 | 100.6805 | 316.8060 |
| High SDI | Both | AD | 1995 | 161.1568 | 86.0191 | 271.7549 |
| Low-middle SDI | Male | AD | 1995 | 75.7770 | 40.8691 | 125.8491 |
| Low-middle SDI | Female | AD | 1995 | 96.7341 | 51.9701 | 161.6014 |
| Low-middle SDI | Both | AD | 1995 | 86.1042 | 46.2515 | 144.7056 |
| Low SDI | Male | AD | 1995 | 56.2671 | 30.1286 | 95.2966 |
| Low SDI | Female | AD | 1995 | 65.3173 | 35.0549 | 109.2942 |
| Low SDI | Both | AD | 1995 | 60.7455 | 32.5667 | 101.8372 |
| Low SDI | Male | AD | 1996 | 56.2163 | 30.2148 | 94.8506 |
| Low SDI | Female | AD | 1996 | 65.2463 | 35.0680 | 109.1615 |
| Low SDI | Both | AD | 1996 | 60.6853 | 32.7835 | 102.2952 |
| Middle SDI | Male | AD | 1996 | 79.1502 | 42.3824 | 132.5777 |
| Middle SDI | Female | AD | 1996 | 128.8719 | 68.9959 | 216.7093 |
| Middle SDI | Both | AD | 1996 | 103.6572 | 55.3419 | 174.5178 |
| Low-middle SDI | Male | AD | 1996 | 75.7820 | 40.8152 | 126.2274 |
| Low-middle SDI | Female | AD | 1996 | 96.6553 | 52.0318 | 162.1087 |
| Low-middle SDI | Both | AD | 1996 | 86.0742 | 46.3195 | 144.5398 |
| High-middle SDI | Male | AD | 1996 | 90.5516 | 48.3556 | 152.6590 |
| High-middle SDI | Female | AD | 1996 | 138.6123 | 74.7529 | 233.5934 |
| High-middle SDI | Both | AD | 1996 | 114.2952 | 61.0508 | 192.5119 |
| High SDI | Male | AD | 1996 | 135.3069 | 71.9915 | 228.5930 |
| High SDI | Female | AD | 1996 | 188.2853 | 100.9072 | 317.4627 |
| High SDI | Both | AD | 1996 | 161.3793 | 86.1150 | 272.0894 |
| High-middle SDI | Male | AD | 1997 | 90.3115 | 48.2688 | 151.4344 |
| High-middle SDI | Female | AD | 1997 | 138.5875 | 74.2343 | 233.4758 |
| High-middle SDI | Both | AD | 1997 | 114.1618 | 60.9409 | 192.0112 |
| High SDI | Male | AD | 1997 | 135.9139 | 72.6477 | 229.9841 |
| High SDI | Female | AD | 1997 | 188.7351 | 100.8156 | 317.2759 |
| High SDI | Both | AD | 1997 | 161.9019 | 86.4722 | 273.8142 |
| Low-middle SDI | Male | AD | 1997 | 75.7658 | 40.6818 | 126.2958 |
| Low-middle SDI | Female | AD | 1997 | 96.6289 | 51.9718 | 161.6405 |
| Low-middle SDI | Both | AD | 1997 | 86.0601 | 46.1765 | 144.2046 |
| Low SDI | Male | AD | 1997 | 56.1447 | 30.2117 | 94.4310 |
| Low SDI | Female | AD | 1997 | 65.2386 | 34.9482 | 108.8236 |
| Low SDI | Both | AD | 1997 | 60.6459 | 32.5542 | 101.6554 |
| Middle SDI | Male | AD | 1997 | 79.1572 | 42.5573 | 132.3146 |
| Middle SDI | Female | AD | 1997 | 128.7780 | 69.0989 | 216.7183 |
| Middle SDI | Both | AD | 1997 | 103.6282 | 55.4489 | 173.8615 |
| High-middle SDI | Male | AD | 1998 | 90.0890 | 48.0594 | 151.1368 |
| High-middle SDI | Female | AD | 1998 | 138.6657 | 74.1170 | 233.6690 |
| High-middle SDI | Both | AD | 1998 | 114.0889 | 60.7905 | 192.2535 |
| High SDI | Male | AD | 1998 | 136.6034 | 72.5112 | 230.7643 |
| High SDI | Female | AD | 1998 | 189.2510 | 100.8633 | 319.2500 |
| High SDI | Both | AD | 1998 | 162.5005 | 86.5018 | 275.0955 |
| Low-middle SDI | Male | AD | 1998 | 75.7477 | 40.6984 | 127.0054 |
| Low-middle SDI | Female | AD | 1998 | 96.5543 | 51.9300 | 162.0536 |
| Low-middle SDI | Both | AD | 1998 | 86.0213 | 46.2950 | 144.9643 |
| Low SDI | Male | AD | 1998 | 56.0809 | 30.2546 | 94.1083 |
| Low SDI | Female | AD | 1998 | 65.2220 | 35.1715 | 108.8137 |
| Low SDI | Both | AD | 1998 | 60.6063 | 32.6798 | 101.3897 |
| Middle SDI | Male | AD | 1998 | 79.1587 | 42.4050 | 132.6789 |
| Middle SDI | Female | AD | 1998 | 128.6858 | 68.6694 | 216.7818 |
| Middle SDI | Both | AD | 1998 | 103.6010 | 55.3738 | 174.2053 |
| Middle SDI | Male | AD | 1999 | 79.1543 | 42.2564 | 132.8270 |
| Middle SDI | Female | AD | 1999 | 128.5962 | 68.6794 | 215.8626 |
| Middle SDI | Both | AD | 1999 | 103.5743 | 55.1810 | 173.9587 |
| High-middle SDI | Male | AD | 1999 | 89.9749 | 48.3502 | 151.3524 |
| High-middle SDI | Female | AD | 1999 | 138.7630 | 74.1550 | 233.3310 |
| High-middle SDI | Both | AD | 1999 | 114.0802 | 60.7497 | 191.9419 |
| High SDI | Male | AD | 1999 | 137.2322 | 72.9396 | 231.7927 |
| High SDI | Female | AD | 1999 | 189.7065 | 101.0165 | 318.6671 |
| High SDI | Both | AD | 1999 | 163.0413 | 86.6614 | 274.8136 |
| Low-middle SDI | Male | AD | 1999 | 75.7303 | 40.6450 | 127.4048 |
| Low-middle SDI | Female | AD | 1999 | 96.4744 | 51.9040 | 160.4297 |
| Low-middle SDI | Both | AD | 1999 | 85.9811 | 46.2081 | 144.0091 |
| Low SDI | Male | AD | 1999 | 56.0867 | 29.9822 | 93.9900 |
| Low SDI | Female | AD | 1999 | 65.2335 | 35.3434 | 109.0246 |
| Low SDI | Both | AD | 1999 | 60.6164 | 32.8464 | 101.9393 |
| High-middle SDI | Male | AD | 2000 | 89.9686 | 47.7707 | 150.9246 |
| High-middle SDI | Female | AD | 2000 | 138.9495 | 74.2675 | 234.8327 |
| High-middle SDI | Both | AD | 2000 | 114.1684 | 60.8128 | 192.9077 |
| Middle SDI | Male | AD | 2000 | 79.1686 | 42.3978 | 132.9453 |
| Middle SDI | Female | AD | 2000 | 128.4746 | 68.6943 | 215.2734 |
| Middle SDI | Both | AD | 2000 | 103.5421 | 55.3044 | 173.8687 |
| Low SDI | Male | AD | 2001 | 56.0281 | 29.9646 | 94.1626 |
| Low SDI | Female | AD | 2001 | 65.2019 | 35.1200 | 108.9280 |
| Low SDI | Both | AD | 2001 | 60.5733 | 32.5147 | 101.6482 |
| Low-middle SDI | Male | AD | 2000 | 75.6955 | 40.8176 | 127.1149 |
| Low-middle SDI | Female | AD | 2000 | 96.3866 | 51.9001 | 160.0715 |
| Low-middle SDI | Both | AD | 2000 | 85.9278 | 46.3006 | 144.2053 |
| High SDI | Male | AD | 2000 | 137.4161 | 73.1012 | 232.3972 |
| High SDI | Female | AD | 2000 | 189.8628 | 101.3780 | 319.9063 |
| High SDI | Both | AD | 2000 | 163.2079 | 86.9838 | 275.4959 |
| Low SDI | Male | AD | 2000 | 56.0577 | 30.1469 | 94.1297 |
| Low SDI | Female | AD | 2000 | 65.2079 | 34.9109 | 108.5094 |
| Low SDI | Both | AD | 2000 | 60.5903 | 32.6578 | 101.2824 |
| High-middle SDI | Male | AD | 2001 | 90.1259 | 48.1517 | 152.2521 |
| High-middle SDI | Female | AD | 2001 | 139.2921 | 74.4296 | 235.1402 |
| High-middle SDI | Both | AD | 2001 | 114.4113 | 60.8951 | 192.7638 |
| High SDI | Male | AD | 2001 | 137.1171 | 72.9481 | 231.7834 |
| High SDI | Female | AD | 2001 | 189.5960 | 100.9933 | 319.7293 |
| High SDI | Both | AD | 2001 | 162.9215 | 86.6127 | 274.7565 |
| Low-middle SDI | Male | AD | 2001 | 75.6725 | 40.5827 | 125.9321 |
| Low-middle SDI | Female | AD | 2001 | 96.2769 | 51.8070 | 160.8841 |
| Low-middle SDI | Both | AD | 2001 | 85.8701 | 46.2031 | 144.2687 |
| Middle SDI | Male | AD | 2001 | 79.1912 | 42.3337 | 132.5115 |
| Middle SDI | Female | AD | 2001 | 128.3580 | 68.4079 | 215.5279 |
| Middle SDI | Both | AD | 2001 | 103.5165 | 55.2338 | 173.9322 |
| High SDI | Male | AD | 2003 | 135.6114 | 72.3048 | 229.3281 |
| High SDI | Female | AD | 2003 | 188.0595 | 100.1984 | 316.7178 |
| High SDI | Both | AD | 2003 | 161.3971 | 85.9671 | 273.1187 |
| High-middle SDI | Male | AD | 2002 | 90.4005 | 48.3876 | 152.6303 |
| High-middle SDI | Female | AD | 2002 | 139.7641 | 74.2117 | 235.7192 |
| High-middle SDI | Both | AD | 2002 | 114.7720 | 61.1431 | 193.9133 |
| Low-middle SDI | Male | AD | 2003 | 75.6101 | 40.6506 | 126.2361 |
| Low-middle SDI | Female | AD | 2003 | 96.0711 | 51.8181 | 160.5855 |
| Low-middle SDI | Both | AD | 2003 | 85.7519 | 46.1952 | 143.4711 |
| High SDI | Male | AD | 2002 | 136.4486 | 73.0296 | 230.2073 |
| High SDI | Female | AD | 2002 | 188.9502 | 100.6905 | 318.6903 |
| High SDI | Both | AD | 2002 | 162.2620 | 86.5459 | 274.4939 |
| Low SDI | Male | AD | 2003 | 55.9512 | 30.0436 | 93.7941 |
| Low SDI | Female | AD | 2003 | 65.1991 | 35.0995 | 109.3294 |
| Low SDI | Both | AD | 2003 | 60.5342 | 32.5500 | 101.3005 |
| Low-middle SDI | Male | AD | 2002 | 75.6263 | 40.8152 | 126.6538 |
| Low-middle SDI | Female | AD | 2002 | 96.2047 | 51.6263 | 160.6399 |
| Low-middle SDI | Both | AD | 2002 | 85.8179 | 45.9619 | 143.8354 |
| Low SDI | Male | AD | 2002 | 55.9737 | 30.1761 | 94.4727 |
| Low SDI | Female | AD | 2002 | 65.2049 | 35.0039 | 109.1910 |
| Low SDI | Both | AD | 2002 | 60.5473 | 32.5702 | 101.1609 |
| Middle SDI | Male | AD | 2002 | 79.2412 | 42.5682 | 132.7206 |
| Middle SDI | Female | AD | 2002 | 128.2463 | 68.5471 | 216.7067 |
| Middle SDI | Both | AD | 2002 | 103.5043 | 55.3034 | 174.1819 |
| Middle SDI | Male | AD | 2003 | 79.2966 | 42.5693 | 132.6050 |
| Middle SDI | Female | AD | 2003 | 128.1800 | 68.4263 | 216.2664 |
| Middle SDI | Both | AD | 2003 | 103.5152 | 55.3678 | 174.6203 |
| High-middle SDI | Male | AD | 2003 | 90.8203 | 48.7103 | 152.7939 |
| High-middle SDI | Female | AD | 2003 | 140.2944 | 74.6396 | 235.8922 |
| High-middle SDI | Both | AD | 2003 | 115.2353 | 61.5020 | 194.6542 |
| Middle SDI | Male | AD | 2004 | 79.3661 | 42.4870 | 133.4351 |
| Middle SDI | Female | AD | 2004 | 128.0931 | 68.4076 | 215.7360 |
| Middle SDI | Both | AD | 2004 | 103.5211 | 55.2429 | 174.6645 |
| High-middle SDI | Male | AD | 2004 | 91.2551 | 48.5716 | 153.4229 |
| High-middle SDI | Female | AD | 2004 | 140.8990 | 75.0719 | 237.1362 |
| High-middle SDI | Both | AD | 2004 | 115.7406 | 61.6124 | 194.5713 |
| Low-middle SDI | Male | AD | 2004 | 75.5783 | 40.6729 | 125.8475 |
| Low-middle SDI | Female | AD | 2004 | 95.9659 | 51.4600 | 160.0374 |
| Low-middle SDI | Both | AD | 2004 | 85.6906 | 45.9586 | 143.1891 |
| High SDI | Male | AD | 2004 | 134.7521 | 72.1964 | 228.0665 |
| High SDI | Female | AD | 2004 | 187.1691 | 99.5398 | 314.8725 |
| High SDI | Both | AD | 2004 | 160.5192 | 85.6454 | 270.4928 |
| Low SDI | Male | AD | 2004 | 55.9215 | 30.1742 | 93.8896 |
| Low SDI | Female | AD | 2004 | 65.2217 | 34.8996 | 108.7435 |
| Low SDI | Both | AD | 2004 | 60.5316 | 32.5800 | 100.8201 |
| Middle SDI | Male | AD | 2005 | 79.3731 | 42.5262 | 133.6264 |
| Middle SDI | Female | AD | 2005 | 127.9655 | 68.2838 | 215.5202 |
| Middle SDI | Both | AD | 2005 | 103.4742 | 55.4368 | 174.2993 |
| High-middle SDI | Male | AD | 2005 | 91.6824 | 48.8362 | 154.1792 |
| High-middle SDI | Female | AD | 2005 | 141.4967 | 75.3095 | 239.0625 |
| High-middle SDI | Both | AD | 2005 | 116.2380 | 61.8830 | 195.8564 |
| High SDI | Male | AD | 2005 | 134.0923 | 71.7655 | 226.1373 |
| High SDI | Female | AD | 2005 | 186.4402 | 99.5152 | 314.4746 |
| High SDI | Both | AD | 2005 | 159.8211 | 85.4080 | 270.0027 |
| Low-middle SDI | Male | AD | 2005 | 75.5008 | 40.8258 | 125.5923 |
| Low-middle SDI | Female | AD | 2005 | 95.8118 | 51.4257 | 160.1378 |
| Low-middle SDI | Both | AD | 2005 | 85.5826 | 46.0905 | 143.7469 |
| Low SDI | Male | AD | 2005 | 55.8756 | 30.1014 | 94.7944 |
| Low SDI | Female | AD | 2005 | 65.2006 | 34.9458 | 108.7828 |
| Low SDI | Both | AD | 2005 | 60.4991 | 32.5057 | 101.8268 |
| High-middle SDI | Male | AD | 2006 | 92.0205 | 49.2981 | 155.3943 |
| High-middle SDI | Female | AD | 2006 | 142.0536 | 75.5966 | 239.4029 |
| High-middle SDI | Both | AD | 2006 | 116.6665 | 61.9623 | 196.8363 |
| High SDI | Male | AD | 2006 | 133.5106 | 71.2263 | 225.6452 |
| High SDI | Female | AD | 2006 | 185.7679 | 99.0244 | 312.7042 |
| High SDI | Both | AD | 2006 | 159.1815 | 84.8815 | 268.8499 |
| Low-middle SDI | Male | AD | 2006 | 75.4637 | 40.5835 | 125.8724 |
| Low-middle SDI | Female | AD | 2006 | 95.6695 | 51.1441 | 159.3780 |
| Low-middle SDI | Both | AD | 2006 | 85.5002 | 45.8314 | 143.4081 |
| Low SDI | Male | AD | 2006 | 55.8386 | 29.8904 | 93.3217 |
| Low SDI | Female | AD | 2006 | 65.2006 | 34.9949 | 109.0618 |
| Low SDI | Both | AD | 2006 | 60.4822 | 32.4243 | 101.4704 |
| Middle SDI | Male | AD | 2006 | 79.3846 | 42.7030 | 133.6963 |
| Middle SDI | Female | AD | 2006 | 127.8410 | 68.1651 | 214.5568 |
| Middle SDI | Both | AD | 2006 | 103.4270 | 55.2155 | 174.0817 |
| Middle SDI | Male | AD | 2007 | 79.4062 | 42.5229 | 133.3726 |
| Middle SDI | Female | AD | 2007 | 127.7879 | 67.9963 | 215.8338 |
| Middle SDI | Both | AD | 2007 | 103.4174 | 55.0450 | 174.4888 |
| High-middle SDI | Male | AD | 2007 | 92.3660 | 49.1311 | 155.9948 |
| High-middle SDI | Female | AD | 2007 | 142.5986 | 75.6836 | 239.8994 |
| High-middle SDI | Both | AD | 2007 | 117.0915 | 62.1960 | 197.5285 |
| High SDI | Male | AD | 2007 | 132.8722 | 71.0812 | 223.9658 |
| High SDI | Female | AD | 2007 | 184.8970 | 99.0578 | 310.5417 |
| High SDI | Both | AD | 2007 | 158.4043 | 84.6025 | 266.3515 |
| Low-middle SDI | Male | AD | 2007 | 75.4374 | 40.6073 | 125.8229 |
| Low-middle SDI | Female | AD | 2007 | 95.5680 | 51.2744 | 159.0320 |
| Low-middle SDI | Both | AD | 2007 | 85.4434 | 45.9918 | 143.1999 |
| Low SDI | Male | AD | 2007 | 55.8186 | 30.1857 | 93.7644 |
| Low SDI | Female | AD | 2007 | 65.1560 | 34.8674 | 108.7525 |
| Low SDI | Both | AD | 2007 | 60.4519 | 32.6829 | 101.2833 |
| High SDI | Male | AD | 2008 | 132.2285 | 70.5997 | 223.5278 |
| High SDI | Female | AD | 2008 | 184.0226 | 98.3751 | 310.6614 |
| High SDI | Both | AD | 2008 | 157.6123 | 84.1602 | 266.5770 |
| Low-middle SDI | Male | AD | 2008 | 75.3790 | 40.5690 | 125.5775 |
| Low-middle SDI | Female | AD | 2008 | 95.4370 | 51.2085 | 160.5393 |
| Low-middle SDI | Both | AD | 2008 | 85.3559 | 45.8444 | 143.1592 |
| Low SDI | Male | AD | 2008 | 55.7726 | 30.2594 | 93.2568 |
| Low SDI | Female | AD | 2008 | 65.0910 | 35.0812 | 108.5839 |
| Low SDI | Both | AD | 2008 | 60.3988 | 32.6866 | 100.7474 |
| Middle SDI | Male | AD | 2008 | 79.4280 | 42.5773 | 133.7979 |
| Middle SDI | Female | AD | 2008 | 127.6966 | 67.9242 | 215.5332 |
| Middle SDI | Both | AD | 2008 | 103.3911 | 55.0742 | 174.9128 |
| High-middle SDI | Male | AD | 2008 | 92.7887 | 49.6983 | 156.1819 |
| High-middle SDI | Female | AD | 2008 | 143.2296 | 76.2074 | 241.6301 |
| High-middle SDI | Both | AD | 2008 | 117.5997 | 62.5732 | 198.2281 |
| Middle SDI | Male | AD | 2009 | 79.4148 | 42.6159 | 133.5335 |
| Middle SDI | Female | AD | 2009 | 127.5710 | 67.8554 | 215.3849 |
| Middle SDI | Both | AD | 2009 | 103.3327 | 55.1517 | 174.5381 |
| High-middle SDI | Male | AD | 2009 | 93.2474 | 49.6880 | 157.2915 |
| High-middle SDI | Female | AD | 2009 | 143.9251 | 76.0867 | 242.5656 |
| High-middle SDI | Both | AD | 2009 | 118.1575 | 62.6663 | 199.5586 |
| High-middle SDI | Male | AD | 2010 | 93.7325 | 49.9802 | 157.5275 |
| High-middle SDI | Female | AD | 2010 | 144.5926 | 76.5804 | 243.8257 |
| High-middle SDI | Both | AD | 2010 | 118.7136 | 62.9291 | 199.9105 |
| High SDI | Male | AD | 2010 | 131.3807 | 70.1247 | 221.3845 |
| High SDI | Female | AD | 2010 | 182.9676 | 97.5835 | 308.3038 |
| High SDI | Both | AD | 2010 | 156.6251 | 83.4020 | 264.0207 |
| High SDI | Male | AD | 2009 | 131.6977 | 70.2895 | 223.0197 |
| High SDI | Female | AD | 2009 | 183.3341 | 97.7885 | 309.1550 |
| High SDI | Both | AD | 2009 | 156.9755 | 83.6881 | 265.3570 |
| Low-middle SDI | Male | AD | 2010 | 75.2570 | 40.4212 | 125.2166 |
| Low-middle SDI | Female | AD | 2010 | 95.1980 | 51.2133 | 159.1597 |
| Low-middle SDI | Both | AD | 2010 | 85.1891 | 45.8674 | 142.8631 |
| Low-middle SDI | Male | AD | 2009 | 75.3459 | 40.3000 | 126.4833 |
| Low-middle SDI | Female | AD | 2009 | 95.3278 | 51.3025 | 158.8523 |
| Low-middle SDI | Both | AD | 2009 | 85.2923 | 45.8968 | 143.4894 |
| Low SDI | Male | AD | 2010 | 55.6592 | 29.8935 | 93.5676 |
| Low SDI | Female | AD | 2010 | 65.0372 | 34.8727 | 108.9129 |
| Low SDI | Both | AD | 2010 | 60.3197 | 32.4870 | 101.2710 |
| Low SDI | Male | AD | 2009 | 55.7323 | 30.0090 | 93.8129 |
| Low SDI | Female | AD | 2009 | 65.0881 | 35.0789 | 108.2772 |
| Low SDI | Both | AD | 2009 | 60.3791 | 32.5919 | 100.9112 |
| Middle SDI | Male | AD | 2010 | 79.4196 | 42.7651 | 133.9470 |
| Middle SDI | Female | AD | 2010 | 127.4242 | 67.7622 | 214.4382 |
| Middle SDI | Both | AD | 2010 | 103.2707 | 55.1920 | 174.6186 |
| Middle SDI | Male | AD | 2011 | 79.3748 | 42.4457 | 133.6201 |
| Middle SDI | Female | AD | 2011 | 127.2515 | 67.7617 | 214.4122 |
| Middle SDI | Both | AD | 2011 | 103.1700 | 55.0310 | 174.3674 |
| High-middle SDI | Male | AD | 2011 | 94.1497 | 50.4207 | 158.8924 |
| High-middle SDI | Female | AD | 2011 | 145.1998 | 77.4193 | 244.5822 |
| High-middle SDI | Both | AD | 2011 | 119.2007 | 63.3567 | 201.1182 |
| High SDI | Male | AD | 2011 | 131.1358 | 69.8586 | 221.7543 |
| High SDI | Female | AD | 2011 | 182.6949 | 97.6819 | 308.1431 |
| High SDI | Both | AD | 2011 | 156.3589 | 83.2357 | 263.9505 |
| Low-middle SDI | Male | AD | 2011 | 75.1319 | 40.2797 | 125.5289 |
| Low-middle SDI | Female | AD | 2011 | 94.9649 | 50.9766 | 160.1344 |
| Low-middle SDI | Both | AD | 2011 | 85.0165 | 45.6854 | 142.6081 |
| Low SDI | Male | AD | 2011 | 55.5677 | 29.6867 | 92.9141 |
| Low SDI | Female | AD | 2011 | 64.9803 | 34.6532 | 108.7030 |
| Low SDI | Both | AD | 2011 | 60.2478 | 32.1554 | 100.9310 |
| Middle SDI | Male | AD | 2012 | 79.3183 | 42.8600 | 133.2894 |
| Middle SDI | Female | AD | 2012 | 127.0388 | 67.6061 | 213.0606 |
| Middle SDI | Both | AD | 2012 | 103.0435 | 55.1675 | 173.6112 |
| High-middle SDI | Male | AD | 2012 | 94.4809 | 50.4328 | 159.3699 |
| High-middle SDI | Female | AD | 2012 | 145.6305 | 77.3868 | 245.4023 |
| High-middle SDI | Both | AD | 2012 | 119.5542 | 63.6382 | 201.9724 |
| Low-middle SDI | Male | AD | 2012 | 74.8376 | 40.4724 | 124.9370 |
| Low-middle SDI | Female | AD | 2012 | 94.7067 | 50.8238 | 157.7946 |
| Low-middle SDI | Both | AD | 2012 | 84.7444 | 45.6440 | 142.1381 |
| High SDI | Male | AD | 2012 | 130.7979 | 69.6032 | 219.6380 |
| High SDI | Female | AD | 2012 | 182.2034 | 97.3358 | 307.1394 |
| High SDI | Both | AD | 2012 | 155.9361 | 83.1206 | 262.9751 |
| Low SDI | Male | AD | 2012 | 55.4310 | 29.5463 | 93.2244 |
| Low SDI | Female | AD | 2012 | 64.8998 | 34.5461 | 108.4140 |
| Low SDI | Both | AD | 2012 | 60.1411 | 32.0319 | 101.1331 |
| High SDI | Male | AD | 2014 | 130.1440 | 69.3203 | 219.4935 |
| High SDI | Female | AD | 2014 | 181.2370 | 96.9550 | 304.5724 |
| High SDI | Both | AD | 2014 | 155.1083 | 82.9205 | 260.9971 |
| High-middle SDI | Male | AD | 2014 | 94.9322 | 50.6360 | 160.3137 |
| High-middle SDI | Female | AD | 2014 | 146.2633 | 77.6639 | 246.9159 |
| High-middle SDI | Both | AD | 2014 | 120.0501 | 63.7792 | 202.8101 |
| High SDI | Male | AD | 2013 | 130.4487 | 69.5992 | 220.0631 |
| High SDI | Female | AD | 2013 | 181.6811 | 97.3507 | 305.0412 |
| High SDI | Both | AD | 2013 | 155.4916 | 83.1607 | 261.4801 |
| High-middle SDI | Male | AD | 2013 | 94.7001 | 50.5553 | 159.4120 |
| High-middle SDI | Female | AD | 2013 | 145.9874 | 77.1805 | 245.7907 |
| High-middle SDI | Both | AD | 2013 | 119.8169 | 63.5024 | 201.9972 |
| Low SDI | Male | AD | 2014 | 55.2061 | 29.3808 | 92.7801 |
| Low SDI | Female | AD | 2014 | 64.7123 | 34.6297 | 107.5688 |
| Low SDI | Both | AD | 2014 | 59.9395 | 32.0053 | 99.9373 |
| Low-middle SDI | Male | AD | 2013 | 74.5713 | 40.1680 | 124.9996 |
| Low-middle SDI | Female | AD | 2013 | 94.3714 | 50.4995 | 156.7465 |
| Low-middle SDI | Both | AD | 2013 | 84.4488 | 45.3700 | 141.4458 |
| Low-middle SDI | Male | AD | 2014 | 74.3309 | 39.9300 | 124.4071 |
| Low-middle SDI | Female | AD | 2014 | 94.0571 | 50.1559 | 156.5988 |
| Low-middle SDI | Both | AD | 2014 | 84.1762 | 45.3087 | 141.2987 |
| Low SDI | Male | AD | 2013 | 55.3283 | 29.6771 | 92.9427 |
| Low SDI | Female | AD | 2013 | 64.8250 | 34.5262 | 108.7506 |
| Low SDI | Both | AD | 2013 | 60.0547 | 32.0445 | 100.5316 |
| Middle SDI | Male | AD | 2013 | 79.2153 | 42.2287 | 133.3574 |
| Middle SDI | Female | AD | 2013 | 126.8078 | 67.4277 | 213.3874 |
| Middle SDI | Both | AD | 2013 | 102.8830 | 54.7609 | 173.8184 |
| Middle SDI | Male | AD | 2014 | 79.1257 | 42.4633 | 133.2579 |
| Middle SDI | Female | AD | 2014 | 126.5881 | 67.2841 | 213.1743 |
| Middle SDI | Both | AD | 2014 | 102.7324 | 54.8104 | 173.3844 |
| High-middle SDI | Male | AD | 2015 | 95.1825 | 50.9683 | 161.0338 |
| High-middle SDI | Female | AD | 2015 | 146.5385 | 77.4195 | 247.7985 |
| High-middle SDI | Both | AD | 2015 | 120.2920 | 63.9013 | 203.0627 |
| High SDI | Male | AD | 2015 | 129.9718 | 69.7172 | 218.5963 |
| High SDI | Female | AD | 2015 | 180.9854 | 97.2202 | 304.9311 |
| High SDI | Both | AD | 2015 | 154.8866 | 83.1553 | 261.1850 |
| Low-middle SDI | Male | AD | 2015 | 74.1547 | 39.8600 | 124.2192 |
| Low-middle SDI | Female | AD | 2015 | 93.9045 | 50.0282 | 156.2197 |
| Low-middle SDI | Both | AD | 2015 | 84.0143 | 45.1353 | 140.1956 |
| Low SDI | Male | AD | 2015 | 55.1141 | 29.3964 | 93.0278 |
| Low SDI | Female | AD | 2015 | 64.6494 | 34.7396 | 107.8136 |
| Low SDI | Both | AD | 2015 | 59.8637 | 32.0943 | 100.4771 |
| Middle SDI | Male | AD | 2015 | 79.0587 | 42.2502 | 133.1234 |
| Middle SDI | Female | AD | 2015 | 126.3756 | 67.1352 | 212.8709 |
| Middle SDI | Both | AD | 2015 | 102.5936 | 54.5696 | 173.1165 |
| Middle SDI | Male | AD | 2016 | 79.0124 | 42.2121 | 133.1003 |
| Middle SDI | Female | AD | 2016 | 126.1967 | 67.2062 | 212.7709 |
| Middle SDI | Both | AD | 2016 | 102.4803 | 54.6424 | 172.7997 |
| High-middle SDI | Male | AD | 2016 | 95.2376 | 50.8041 | 161.3073 |
| High-middle SDI | Female | AD | 2016 | 146.6451 | 77.6481 | 247.0101 |
| High-middle SDI | Both | AD | 2016 | 120.3485 | 63.9131 | 202.8256 |
| High SDI | Male | AD | 2016 | 129.9176 | 69.5708 | 219.2809 |
| High SDI | Female | AD | 2016 | 180.9761 | 96.9566 | 304.2824 |
| High SDI | Both | AD | 2016 | 154.8444 | 82.8930 | 261.1115 |
| Low-middle SDI | Male | AD | 2016 | 74.0672 | 39.7404 | 123.8773 |
| Low-middle SDI | Female | AD | 2016 | 93.7341 | 50.1758 | 156.7869 |
| Low-middle SDI | Both | AD | 2016 | 83.8886 | 45.0859 | 140.5593 |
| Low SDI | Male | AD | 2016 | 55.0791 | 29.3332 | 92.3591 |
| Low SDI | Female | AD | 2016 | 64.6426 | 34.3844 | 107.6963 |
| Low SDI | Both | AD | 2016 | 59.8442 | 31.8451 | 100.0503 |
| Middle SDI | Male | AD | 2017 | 78.9590 | 42.2213 | 133.2217 |
| Middle SDI | Female | AD | 2017 | 125.9589 | 67.0611 | 212.0744 |
| Middle SDI | Both | AD | 2017 | 102.3319 | 54.5720 | 171.7667 |
| Low-middle SDI | Male | AD | 2018 | 73.9218 | 39.6559 | 123.4107 |
| Low-middle SDI | Female | AD | 2018 | 93.4274 | 49.6737 | 155.5304 |
| Low-middle SDI | Both | AD | 2018 | 83.6684 | 44.6673 | 139.4447 |
| Low-middle SDI | Male | AD | 2017 | 73.9810 | 39.7246 | 124.2794 |
| Low-middle SDI | Female | AD | 2017 | 93.5574 | 49.8103 | 156.0970 |
| Low-middle SDI | Both | AD | 2017 | 83.7606 | 44.8242 | 140.0527 |
| High SDI | Male | AD | 2017 | 129.8682 | 69.3392 | 218.3663 |
| High SDI | Female | AD | 2017 | 180.9497 | 96.6589 | 304.6112 |
| High SDI | Both | AD | 2017 | 154.7975 | 82.6770 | 260.4302 |
| Low SDI | Male | AD | 2018 | 54.9467 | 29.1833 | 92.8799 |
| Low SDI | Female | AD | 2018 | 64.5780 | 34.7230 | 106.9986 |
| Low SDI | Both | AD | 2018 | 59.7473 | 31.9266 | 99.9682 |
| Low SDI | Male | AD | 2017 | 55.0456 | 29.3644 | 92.7694 |
| Low SDI | Female | AD | 2017 | 64.6476 | 34.7454 | 107.7179 |
| Low SDI | Both | AD | 2017 | 59.8309 | 32.0153 | 100.2512 |
| High-middle SDI | Male | AD | 2017 | 95.1199 | 50.7105 | 160.9400 |
| High-middle SDI | Female | AD | 2017 | 146.5163 | 78.0604 | 246.4447 |
| High-middle SDI | Both | AD | 2017 | 120.2041 | 64.0514 | 203.0353 |
| High-middle SDI | Male | AD | 2018 | 95.1970 | 50.8244 | 160.1189 |
| High-middle SDI | Female | AD | 2018 | 146.5005 | 77.5943 | 246.0115 |
| High-middle SDI | Both | AD | 2018 | 120.2176 | 63.8612 | 202.6070 |
| High SDI | Male | AD | 2018 | 129.8819 | 69.5264 | 219.5036 |
| High SDI | Female | AD | 2018 | 181.3827 | 97.4583 | 305.2268 |
| High SDI | Both | AD | 2018 | 155.0074 | 83.1523 | 260.7252 |
| Middle SDI | Male | AD | 2018 | 78.9596 | 42.2586 | 132.2389 |
| Middle SDI | Female | AD | 2018 | 125.7678 | 66.8990 | 211.9982 |
| Middle SDI | Both | AD | 2018 | 102.2333 | 54.3358 | 171.7092 |
| Middle SDI | Male | AD | 2019 | 79.0778 | 42.1330 | 132.3027 |
| Middle SDI | Female | AD | 2019 | 125.6938 | 66.9636 | 210.9003 |
| Middle SDI | Both | AD | 2019 | 102.2516 | 54.2922 | 171.6548 |
| High-middle SDI | Male | AD | 2019 | 95.4297 | 50.8194 | 159.6917 |
| High-middle SDI | Female | AD | 2019 | 146.7301 | 77.9871 | 246.9473 |
| High-middle SDI | Both | AD | 2019 | 120.4296 | 63.8046 | 202.1894 |
| High SDI | Male | AD | 2019 | 129.9786 | 69.5909 | 219.3316 |
| High SDI | Female | AD | 2019 | 182.3910 | 98.0570 | 307.3115 |
| High SDI | Both | AD | 2019 | 155.5406 | 83.4644 | 261.9977 |
| Low-middle SDI | Male | AD | 2019 | 73.8530 | 39.5751 | 123.5061 |
| Low-middle SDI | Female | AD | 2019 | 93.3111 | 49.6542 | 155.4591 |
| Low-middle SDI | Both | AD | 2019 | 83.5774 | 44.7767 | 139.8255 |
| Low SDI | Male | AD | 2019 | 54.8877 | 29.3733 | 91.9758 |
| Low SDI | Female | AD | 2019 | 64.4972 | 34.7426 | 107.7339 |
| Low SDI | Both | AD | 2019 | 59.6780 | 31.9642 | 100.1477 |
|  |  |  |  |  |  |  |

**Table12 Incidence of AD in 2019, Page 49-53**

| **location_name** | **sex_name** | **cause_name** | **year** | **Age-standardised incidence rate (per 100 000 person-years)** | **95%CI(lower)** | **95%CI(upper)** |
| --- | --- | --- | --- | --- | --- | --- |
| Philippines | Both | AD | 2019 | 512.8515 | 491.6040 | 532.9934 |
| Belarus | Both | AD | 2019 | 204.7027 | 187.9342 | 222.4419 |
| Taiwan (Province of China) | Both | AD | 2019 | 529.3977 | 495.4278 | 566.7413 |
| Azerbaijan | Both | AD | 2019 | 605.1250 | 534.7115 | 685.1474 |
| Malaysia | Both | AD | 2019 | 502.5902 | 471.2854 | 536.3363 |
| Serbia | Both | AD | 2019 | 202.9921 | 188.2793 | 218.5522 |
| Cambodia | Both | AD | 2019 | 510.7616 | 478.7848 | 544.4487 |
| Kyrgyzstan | Both | AD | 2019 | 612.2716 | 541.4535 | 692.7604 |
| Tonga | Both | AD | 2019 | 490.6763 | 456.9786 | 523.4535 |
| Maldives | Both | AD | 2019 | 475.3574 | 445.9955 | 506.7868 |
| Kazakhstan | Both | AD | 2019 | 613.0807 | 542.2786 | 693.5351 |
| Georgia | Both | AD | 2019 | 602.4748 | 527.4787 | 684.7861 |
| Cyprus | Both | AD | 2019 | 433.4166 | 391.8262 | 477.8563 |
| Estonia | Both | AD | 2019 | 308.1448 | 277.9625 | 340.1174 |
| China | Both | AD | 2019 | 411.1283 | 394.1355 | 427.1707 |
| Slovakia | Both | AD | 2019 | 203.5341 | 188.7558 | 219.0763 |
| Mongolia | Both | AD | 2019 | 613.4804 | 542.4961 | 694.0930 |
| Latvia | Both | AD | 2019 | 144.5952 | 132.7281 | 158.7638 |
| Democratic People's Republic of Korea | Both | AD | 2019 | 456.7203 | 427.2044 | 485.7612 |
| Vanuatu | Both | AD | 2019 | 488.6927 | 455.1061 | 521.3369 |
| Indonesia | Both | AD | 2019 | 565.7525 | 540.5181 | 589.6943 |
| USA | Both | AD | 2019 | 300.4178 | 284.0146 | 317.2582 |
| Oman | Both | AD | 2019 | 189.2594 | 176.5835 | 202.9376 |
| Slovenia | Both | AD | 2019 | 203.0200 | 188.2252 | 218.5667 |
| Canada | Both | AD | 2019 | 244.9375 | 231.3828 | 258.7584 |
| Lithuania | Both | AD | 2019 | 204.7196 | 187.9549 | 222.4608 |
| Timor-Leste | Both | AD | 2019 | 508.7038 | 476.8093 | 542.6844 |
| Lao People's Democratic Republic | Both | AD | 2019 | 508.3297 | 476.4448 | 542.2380 |
| Sri Lanka | Both | AD | 2019 | 470.5690 | 437.8781 | 502.9209 |
| Denmark | Both | AD | 2019 | 518.1345 | 469.8271 | 570.8019 |
| Uzbekistan | Both | AD | 2019 | 610.7558 | 540.0437 | 691.2149 |
| Republic of Moldova | Both | AD | 2019 | 204.5599 | 187.7942 | 222.2900 |
| Japan | Both | AD | 2019 | 582.4873 | 545.5188 | 618.6264 |
| Viet Nam | Both | AD | 2019 | 508.2483 | 476.4683 | 541.9296 |
| Qatar | Both | AD | 2019 | 185.9055 | 172.8932 | 199.3481 |
| Thailand | Both | AD | 2019 | 510.0116 | 477.9369 | 543.8396 |
| Finland | Both | AD | 2019 | 447.9959 | 414.2191 | 484.2981 |
| Fiji | Both | AD | 2019 | 485.7331 | 452.4096 | 518.3373 |
| Armenia | Both | AD | 2019 | 607.1020 | 536.5337 | 687.1424 |
| Russian Federation | Both | AD | 2019 | 285.6658 | 267.0573 | 304.7543 |
| Czechia | Both | AD | 2019 | 203.3733 | 188.6119 | 218.9096 |
| Kiribati | Both | AD | 2019 | 492.0846 | 458.4215 | 525.0195 |
| Micronesia (Federated States of) | Both | AD | 2019 | 485.2413 | 451.9229 | 517.9065 |
| Albania | Both | AD | 2019 | 203.1034 | 188.3651 | 218.6589 |
| Haiti | Both | AD | 2019 | 274.3953 | 251.8873 | 296.3150 |
| Antigua and Barbuda | Both | AD | 2019 | 274.0090 | 251.5300 | 295.9140 |
| Norway | Both | AD | 2019 | 496.9742 | 466.2516 | 529.3964 |
| Ukraine | Both | AD | 2019 | 218.2058 | 203.6222 | 233.4835 |
| Costa Rica | Both | AD | 2019 | 215.7621 | 199.8312 | 232.7423 |
| Bulgaria | Both | AD | 2019 | 203.3336 | 188.5379 | 218.8701 |
| Hungary | Both | AD | 2019 | 250.5139 | 228.9936 | 275.7087 |
| Marshall Islands | Both | AD | 2019 | 484.8601 | 451.4372 | 517.4713 |
| Israel | Both | AD | 2019 | 448.4971 | 414.8461 | 484.8304 |
| Iceland | Both | AD | 2019 | 490.5566 | 446.1411 | 541.6412 |
| Bahamas | Both | AD | 2019 | 274.4226 | 251.9067 | 296.3293 |
| Djibouti | Both | AD | 2019 | 152.2277 | 142.6090 | 162.4720 |
| North Macedonia | Both | AD | 2019 | 202.9473 | 188.1391 | 218.4904 |
| Ireland | Both | AD | 2019 | 449.0417 | 415.4167 | 485.4290 |
| Papua New Guinea | Both | AD | 2019 | 484.4148 | 451.0862 | 517.0021 |
| Portugal | Both | AD | 2019 | 449.8174 | 416.1117 | 486.2339 |
| Bosnia and Herzegovina | Both | AD | 2019 | 203.7382 | 188.9846 | 219.2922 |
| Croatia | Both | AD | 2019 | 203.5383 | 188.7710 | 219.0887 |
| Myanmar | Both | AD | 2019 | 514.7220 | 482.3318 | 548.9123 |
| El Salvador | Both | AD | 2019 | 215.9607 | 200.0700 | 232.8897 |
| Barbados | Both | AD | 2019 | 273.9304 | 251.4417 | 295.8253 |
| Suriname | Both | AD | 2019 | 273.8884 | 251.4435 | 295.8141 |
| Cabo Verde | Both | AD | 2019 | 161.6294 | 152.3680 | 171.5997 |
| Sweden | Both | AD | 2019 | 460.8398 | 433.4924 | 488.7577 |
| Grenada | Both | AD | 2019 | 273.4552 | 251.0792 | 295.3399 |
| Palau | Both | AD | 2019 | 470.6077 | 438.4960 | 501.8012 |
| Saint Lucia | Both | AD | 2019 | 273.8520 | 251.4168 | 295.7455 |
| Jamaica | Both | AD | 2019 | 273.8969 | 251.4746 | 295.7841 |
| Kenya | Both | AD | 2019 | 141.1020 | 133.0654 | 148.9286 |
| Saudi Arabia | Both | AD | 2019 | 190.7776 | 177.8355 | 204.4240 |
| Honduras | Both | AD | 2019 | 215.7732 | 199.8311 | 232.7399 |
| Chad | Both | AD | 2019 | 161.6136 | 152.2622 | 171.6558 |
| Spain | Both | AD | 2019 | 423.7026 | 386.6444 | 466.6545 |
| Eritrea | Both | AD | 2019 | 153.2099 | 143.5133 | 163.5683 |
| Guyana | Both | AD | 2019 | 274.0526 | 251.5998 | 295.9566 |
| Brunei Darussalam | Both | AD | 2019 | 471.5669 | 435.3773 | 509.5298 |
| Somalia | Both | AD | 2019 | 153.0546 | 143.3812 | 163.3660 |
| Ethiopia | Both | AD | 2019 | 143.1518 | 135.7175 | 151.3951 |
| Bangladesh | Both | AD | 2019 | 276.7683 | 253.2002 | 305.5682 |
| Guatemala | Both | AD | 2019 | 215.8649 | 199.9503 | 232.8400 |
| Turkey | Both | AD | 2019 | 297.0058 | 274.8792 | 320.5669 |
| Republic of Korea | Both | AD | 2019 | 475.6479 | 437.0903 | 517.9013 |
| Benin | Both | AD | 2019 | 161.8143 | 152.4517 | 171.8153 |
| India | Both | AD | 2019 | 277.8960 | 261.2949 | 295.8806 |
| Bermuda | Both | AD | 2019 | 274.1068 | 251.6667 | 295.9987 |
| Mexico | Both | AD | 2019 | 218.5272 | 206.1042 | 230.3678 |
| Solomon Islands | Both | AD | 2019 | 487.2564 | 453.8123 | 519.8079 |
| Luxembourg | Both | AD | 2019 | 447.5901 | 413.8082 | 483.8309 |
| Montenegro | Both | AD | 2019 | 203.2276 | 188.4145 | 218.7865 |
| Switzerland | Both | AD | 2019 | 447.4948 | 413.7983 | 483.7829 |
| Burundi | Both | AD | 2019 | 153.1902 | 143.4921 | 163.6021 |
| Iraq | Both | AD | 2019 | 193.3951 | 177.6170 | 209.6602 |
| Malta | Both | AD | 2019 | 446.8142 | 413.0836 | 483.1027 |
| Italy | Both | AD | 2019 | 507.7042 | 476.3061 | 540.3699 |
| American Samoa | Both | AD | 2019 | 488.0393 | 454.5741 | 520.7350 |
| Puerto Rico | Both | AD | 2019 | 273.9309 | 251.4380 | 295.8137 |
| Tunisia | Both | AD | 2019 | 193.7263 | 180.5081 | 207.6832 |
| Trinidad and Tobago | Both | AD | 2019 | 273.7244 | 251.3256 | 295.6072 |
| Saint Kitts and Nevis | Both | AD | 2019 | 273.9132 | 251.5201 | 295.7941 |
| Namibia | Both | AD | 2019 | 159.7076 | 146.9766 | 174.2104 |
| United Republic of Tanzania | Both | AD | 2019 | 192.0802 | 180.7121 | 204.4375 |
| Jordan | Both | AD | 2019 | 192.3829 | 179.3146 | 206.1901 |
| Togo | Both | AD | 2019 | 161.8368 | 152.4546 | 171.7906 |
| Netherlands | Both | AD | 2019 | 448.2772 | 414.5311 | 484.6323 |
| Saint Vincent and the Grenadines | Both | AD | 2019 | 273.6295 | 251.2888 | 295.5156 |
| Greece | Both | AD | 2019 | 386.1700 | 348.6992 | 427.9977 |
| Cameroon | Both | AD | 2019 | 178.5944 | 168.8552 | 188.7942 |
| Argentina | Both | AD | 2019 | 401.9844 | 371.1008 | 435.5718 |
| Germany | Both | AD | 2019 | 438.8054 | 404.5495 | 475.1276 |
| Burkina Faso | Both | AD | 2019 | 161.9249 | 152.5478 | 171.9231 |
| United Kingdom | Both | AD | 2019 | 497.6735 | 467.3726 | 529.2968 |
| South Sudan | Both | AD | 2019 | 152.9236 | 143.2525 | 163.2082 |
| Samoa | Both | AD | 2019 | 484.3898 | 451.1170 | 516.8828 |
| Singapore | Both | AD | 2019 | 440.7755 | 400.2279 | 482.4798 |
| Poland | Both | AD | 2019 | 188.6580 | 179.6103 | 198.3716 |
| C么te d'Ivoire | Both | AD | 2019 | 161.1861 | 151.8863 | 171.1626 |
| Greenland | Both | AD | 2019 | 243.5613 | 229.9249 | 257.3162 |
| Bhutan | Both | AD | 2019 | 276.0103 | 252.3673 | 304.7779 |
| Syrian Arab Republic | Both | AD | 2019 | 194.7326 | 181.3666 | 208.7692 |
| France | Both | AD | 2019 | 550.9810 | 505.2847 | 603.3319 |
| Panama | Both | AD | 2019 | 215.3947 | 199.5150 | 232.3813 |
| Kuwait | Both | AD | 2019 | 192.5099 | 179.3841 | 206.3333 |
| Cuba | Both | AD | 2019 | 273.4730 | 251.0540 | 295.3471 |
| United Arab Emirates | Both | AD | 2019 | 188.1017 | 175.3475 | 202.0668 |
| Romania | Both | AD | 2019 | 152.7001 | 141.6490 | 163.2766 |
| Nicaragua | Both | AD | 2019 | 215.6727 | 199.7660 | 232.6774 |
| Tajikistan | Both | AD | 2019 | 610.4033 | 539.5303 | 691.0575 |
| Turkmenistan | Both | AD | 2019 | 608.3290 | 537.2618 | 688.6020 |
| Niger | Both | AD | 2019 | 161.7104 | 152.3729 | 171.7121 |
| Sudan | Both | AD | 2019 | 193.5798 | 180.2981 | 207.6327 |
| Angola | Both | AD | 2019 | 159.6861 | 146.9557 | 174.2223 |
| Mauritania | Both | AD | 2019 | 161.7165 | 152.3641 | 171.7399 |
| Cook Islands | Both | AD | 2019 | 494.5990 | 460.3684 | 527.7969 |
| Belize | Both | AD | 2019 | 273.8893 | 251.4687 | 295.7532 |
| Comoros | Both | AD | 2019 | 153.1290 | 143.4183 | 163.4519 |
| Egypt | Both | AD | 2019 | 135.6675 | 125.3169 | 146.2820 |
| Guam | Both | AD | 2019 | 482.1135 | 448.8738 | 514.6366 |
| Libya | Both | AD | 2019 | 193.1717 | 180.0526 | 207.0348 |
| Algeria | Both | AD | 2019 | 193.5187 | 180.3640 | 207.4908 |
| Nepal | Both | AD | 2019 | 277.3500 | 253.7279 | 306.1151 |
| Bolivia (Plurinational State of) | Both | AD | 2019 | 263.3253 | 247.3633 | 280.0525 |
| Bahrain | Both | AD | 2019 | 190.7058 | 177.6585 | 204.3829 |
| Australia | Both | AD | 2019 | 345.2736 | 316.7061 | 375.8931 |
| Dominica | Both | AD | 2019 | 273.5541 | 251.2030 | 295.4590 |
| Senegal | Both | AD | 2019 | 161.5312 | 152.1861 | 171.4936 |
| Gambia | Both | AD | 2019 | 161.7564 | 152.3951 | 171.7780 |
| Austria | Both | AD | 2019 | 447.4944 | 413.8629 | 483.7625 |
| Eswatini | Both | AD | 2019 | 159.4383 | 146.7163 | 174.0433 |
| Central African Republic | Both | AD | 2019 | 159.6059 | 146.8912 | 174.1313 |
| Uganda | Both | AD | 2019 | 193.5827 | 179.1354 | 209.7383 |
| Nigeria | Both | AD | 2019 | 162.9174 | 154.4362 | 171.9801 |
| Pakistan | Both | AD | 2019 | 277.6930 | 261.1182 | 295.7021 |
| Sierra Leone | Both | AD | 2019 | 161.7464 | 152.3973 | 171.7609 |
| Democratic Republic of the Congo | Both | AD | 2019 | 159.3956 | 146.6843 | 173.8998 |
| Lebanon | Both | AD | 2019 | 193.8023 | 180.6086 | 207.8274 |
| United States Virgin Islands | Both | AD | 2019 | 274.1954 | 251.6878 | 296.1132 |
| San Marino | Both | AD | 2019 | 450.6693 | 416.9698 | 487.1979 |
| New Zealand | Both | AD | 2019 | 441.3419 | 415.1122 | 468.9518 |
| Sao Tome and Principe | Both | AD | 2019 | 161.6392 | 152.3412 | 171.6194 |
| Chile | Both | AD | 2019 | 401.2708 | 370.4124 | 434.7535 |
| Congo | Both | AD | 2019 | 159.4474 | 146.7490 | 173.9606 |
| Madagascar | Both | AD | 2019 | 153.1086 | 143.4205 | 163.4458 |
| Iran (Islamic Republic of) | Both | AD | 2019 | 198.1347 | 187.6879 | 209.0400 |
| Belgium | Both | AD | 2019 | 448.5428 | 414.8452 | 484.8948 |
| Zambia | Both | AD | 2019 | 153.2796 | 143.5872 | 163.6740 |
| Monaco | Both | AD | 2019 | 450.3108 | 416.3331 | 486.9118 |
| Uruguay | Both | AD | 2019 | 401.8463 | 371.1436 | 435.3353 |
| Tokelau | Both | AD | 2019 | 484.1524 | 450.9369 | 516.5488 |
| Malawi | Both | AD | 2019 | 153.4944 | 143.8412 | 163.9610 |
| Venezuela (Bolivarian Republic of) | Both | AD | 2019 | 215.6229 | 199.6888 | 232.6254 |
| Yemen | Both | AD | 2019 | 193.7014 | 180.5360 | 207.6333 |
| Nauru | Both | AD | 2019 | 487.9532 | 454.3993 | 520.6728 |
| Morocco | Both | AD | 2019 | 193.7042 | 180.5383 | 207.6802 |
| Botswana | Both | AD | 2019 | 159.4800 | 146.7619 | 174.0057 |
| Andorra | Both | AD | 2019 | 447.3810 | 413.4852 | 483.7603 |
| Zimbabwe | Both | AD | 2019 | 159.7915 | 147.0503 | 174.3042 |
| Equatorial Guinea | Both | AD | 2019 | 158.4587 | 145.7492 | 172.9112 |
| Tuvalu | Both | AD | 2019 | 480.9261 | 448.0137 | 513.5842 |
| Mauritius | Both | AD | 2019 | 508.5404 | 476.7255 | 542.4050 |
| Ecuador | Both | AD | 2019 | 278.9629 | 261.2762 | 297.3689 |
| Dominican Republic | Both | AD | 2019 | 273.7655 | 251.3479 | 295.6343 |
| Peru | Both | AD | 2019 | 247.3383 | 232.0394 | 263.2171 |
| Lesotho | Both | AD | 2019 | 159.5640 | 146.8207 | 174.0955 |
| Ghana | Both | AD | 2019 | 137.5524 | 130.1230 | 145.6984 |
| Niue | Both | AD | 2019 | 485.6711 | 452.4566 | 518.1229 |
| Guinea | Both | AD | 2019 | 161.8433 | 152.4638 | 171.8771 |
| South Africa | Both | AD | 2019 | 160.8861 | 151.4532 | 170.7724 |
| Colombia | Both | AD | 2019 | 215.6021 | 199.7144 | 232.6007 |
| Palestine | Both | AD | 2019 | 193.5033 | 180.3886 | 207.4603 |
| Guinea-Bissau | Both | AD | 2019 | 161.9254 | 152.5435 | 171.9265 |
| Gabon | Both | AD | 2019 | 159.6777 | 146.9585 | 174.2769 |
| Northern Mariana Islands | Both | AD | 2019 | 482.6623 | 449.6046 | 514.8109 |
| Mozambique | Both | AD | 2019 | 153.5734 | 143.9243 | 164.0338 |
| Rwanda | Both | AD | 2019 | 112.0677 | 104.2422 | 120.4800 |
| Brazil | Both | AD | 2019 | 320.0770 | 303.6047 | 337.4196 |
| Afghanistan | Both | AD | 2019 | 193.3534 | 180.2469 | 207.2305 |
| Liberia | Both | AD | 2019 | 161.4447 | 152.1190 | 171.4169 |
| Paraguay | Both | AD | 2019 | 319.3212 | 294.7296 | 344.3303 |
| Mali | Both | AD | 2019 | 169.3764 | 158.2789 | 181.5618 |
| Seychelles | Both | AD | 2019 | 497.1469 | 466.2185 | 529.9816 |
|  |  |  |  |  |  |  |

**Table13 DALYs of AD in 2019, Page 54-58**

| **location_name** | **sex_name** | **cause_name** | **year** | **Age-standardised DALYs rate (per 100 000 person-years)** | **95%CI(lower)** | **95%CI(upper)** |
| --- | --- | --- | --- | --- | --- | --- |
| Philippines | Both | AD | 2019 | 141.3532 | 75.7861 | 237.5394 |
| Sri Lanka | Both | AD | 2019 | 127.0602 | 68.3619 | 213.0274 |
| Thailand | Both | AD | 2019 | 142.4982 | 76.7981 | 239.3470 |
| Maldives | Both | AD | 2019 | 131.0342 | 69.8257 | 218.0604 |
| Malaysia | Both | AD | 2019 | 139.5904 | 75.2735 | 235.7639 |
| Kazakhstan | Both | AD | 2019 | 208.3384 | 110.8335 | 348.5629 |
| Myanmar | Both | AD | 2019 | 143.0706 | 76.9797 | 241.2484 |
| Argentina | Both | AD | 2019 | 134.6639 | 73.0541 | 226.1928 |
| Trinidad and Tobago | Both | AD | 2019 | 84.8598 | 45.8341 | 143.3571 |
| Kyrgyzstan | Both | AD | 2019 | 207.9644 | 110.2635 | 349.9069 |
| Suriname | Both | AD | 2019 | 84.7909 | 46.0463 | 142.6546 |
| Mongolia | Both | AD | 2019 | 208.5840 | 111.2118 | 347.7779 |
| China | Both | AD | 2019 | 107.4699 | 57.7952 | 178.0790 |
| Latvia | Both | AD | 2019 | 59.9913 | 32.1940 | 103.5671 |
| Italy | Both | AD | 2019 | 188.9148 | 100.3505 | 320.1750 |
| Belgium | Both | AD | 2019 | 164.0346 | 87.2716 | 273.2930 |
| Bosnia and Herzegovina | Both | AD | 2019 | 87.5724 | 46.9696 | 146.3021 |
| Lithuania | Both | AD | 2019 | 92.6741 | 49.8341 | 157.9494 |
| Tajikistan | Both | AD | 2019 | 207.1405 | 110.7998 | 349.2935 |
| Lao People's Democratic Republic | Both | AD | 2019 | 141.1956 | 75.7484 | 237.0741 |
| Chile | Both | AD | 2019 | 134.4234 | 72.2998 | 227.4833 |
| Fiji | Both | AD | 2019 | 132.2590 | 70.4216 | 221.2186 |
| Iran (Islamic Republic of) | Both | AD | 2019 | 59.9957 | 32.4316 | 101.5625 |
| Republic of Moldova | Both | AD | 2019 | 92.4990 | 49.1849 | 154.8628 |
| Luxembourg | Both | AD | 2019 | 163.6744 | 86.7595 | 274.5228 |
| Turkmenistan | Both | AD | 2019 | 206.5644 | 110.2267 | 348.1541 |
| Iraq | Both | AD | 2019 | 58.9083 | 31.3801 | 100.8352 |
| Iceland | Both | AD | 2019 | 186.1422 | 98.4809 | 315.5426 |
| Democratic People's Republic of Korea | Both | AD | 2019 | 123.0216 | 67.0105 | 205.8380 |
| Kiribati | Both | AD | 2019 | 134.1656 | 72.3786 | 224.1593 |
| Malta | Both | AD | 2019 | 163.4532 | 88.4401 | 274.8097 |
| Uzbekistan | Both | AD | 2019 | 206.9185 | 110.7253 | 348.0905 |
| Brunei Darussalam | Both | AD | 2019 | 177.0678 | 93.9631 | 298.4929 |
| Uruguay | Both | AD | 2019 | 134.6739 | 72.1974 | 226.7250 |
| Samoa | Both | AD | 2019 | 132.5738 | 69.7936 | 222.0787 |
| Bolivia (Plurinational State of) | Both | AD | 2019 | 84.3595 | 45.7199 | 142.1122 |
| Jordan | Both | AD | 2019 | 58.8028 | 31.3979 | 100.2299 |
| Pakistan | Both | AD | 2019 | 80.1612 | 43.3378 | 133.3823 |
| Ireland | Both | AD | 2019 | 164.1561 | 88.9323 | 276.3154 |
| Taiwan (Province of China) | Both | AD | 2019 | 150.5710 | 80.3031 | 253.0785 |
| Marshall Islands | Both | AD | 2019 | 131.8823 | 70.1568 | 222.2567 |
| Armenia | Both | AD | 2019 | 206.3793 | 110.2342 | 348.3353 |
| Barbados | Both | AD | 2019 | 85.1432 | 45.5296 | 143.8908 |
| Slovakia | Both | AD | 2019 | 87.7070 | 46.8551 | 147.5578 |
| Solomon Islands | Both | AD | 2019 | 132.9909 | 71.3710 | 223.5250 |
| Ecuador | Both | AD | 2019 | 92.6707 | 49.1306 | 155.3901 |
| Japan | Both | AD | 2019 | 236.1474 | 126.3096 | 396.9329 |
| Israel | Both | AD | 2019 | 164.1936 | 87.7176 | 275.3663 |
| Dominica | Both | AD | 2019 | 84.8691 | 45.3754 | 142.8093 |
| Micronesia (Federated States of) | Both | AD | 2019 | 132.5032 | 70.7479 | 224.3051 |
| Montenegro | Both | AD | 2019 | 87.7029 | 47.0459 | 146.4711 |
| Azerbaijan | Both | AD | 2019 | 205.8631 | 109.4196 | 349.0203 |
| Equatorial Guinea | Both | AD | 2019 | 47.2188 | 25.2055 | 79.8929 |
| Bulgaria | Both | AD | 2019 | 87.5765 | 46.5641 | 147.0981 |
| Bermuda | Both | AD | 2019 | 85.3590 | 45.4273 | 145.3867 |
| Georgia | Both | AD | 2019 | 204.4079 | 109.0166 | 345.6397 |
| Haiti | Both | AD | 2019 | 84.4938 | 45.4681 | 141.6153 |
| Cyprus | Both | AD | 2019 | 153.9140 | 83.4283 | 265.1215 |
| Slovenia | Both | AD | 2019 | 87.4795 | 47.0710 | 148.7266 |
| Tonga | Both | AD | 2019 | 134.6930 | 72.8230 | 225.7564 |
| Russian Federation | Both | AD | 2019 | 153.7014 | 81.7486 | 259.8660 |
| Timor-Leste | Both | AD | 2019 | 141.0398 | 75.8586 | 236.8216 |
| Cuba | Both | AD | 2019 | 84.8220 | 46.3250 | 141.3748 |
| Guyana | Both | AD | 2019 | 84.6020 | 45.6851 | 141.2976 |
| Tuvalu | Both | AD | 2019 | 131.6453 | 69.7827 | 219.4458 |
| United Arab Emirates | Both | AD | 2019 | 57.4521 | 30.7356 | 95.3835 |
| Sao Tome and Principe | Both | AD | 2019 | 48.6782 | 26.5139 | 82.5175 |
| Vanuatu | Both | AD | 2019 | 133.2352 | 71.3237 | 222.8284 |
| Grenada | Both | AD | 2019 | 84.7129 | 46.0912 | 141.8507 |
| Papua New Guinea | Both | AD | 2019 | 131.7470 | 70.0872 | 221.8565 |
| Republic of Korea | Both | AD | 2019 | 178.7920 | 96.7023 | 302.5268 |
| Senegal | Both | AD | 2019 | 48.3159 | 25.5614 | 82.3443 |
| Poland | Both | AD | 2019 | 81.9431 | 43.9490 | 137.8203 |
| Romania | Both | AD | 2019 | 61.9324 | 32.6398 | 104.3473 |
| Malawi | Both | AD | 2019 | 45.7322 | 24.6452 | 77.5977 |
| Gabon | Both | AD | 2019 | 47.5582 | 25.6043 | 80.6056 |
| Paraguay | Both | AD | 2019 | 106.0232 | 56.6648 | 179.3613 |
| San Marino | Both | AD | 2019 | 164.8374 | 87.3738 | 276.8515 |
| France | Both | AD | 2019 | 224.5440 | 118.3761 | 380.8369 |
| Brazil | Both | AD | 2019 | 103.6791 | 55.4050 | 172.9981 |
| Panama | Both | AD | 2019 | 63.8509 | 35.0181 | 106.9884 |
| Greenland | Both | AD | 2019 | 86.8607 | 46.0086 | 148.4952 |
| Mauritania | Both | AD | 2019 | 48.4674 | 25.8366 | 82.1101 |
| Albania | Both | AD | 2019 | 87.7175 | 47.0460 | 148.3811 |
| Norway | Both | AD | 2019 | 183.9373 | 97.7998 | 309.0104 |
| Eswatini | Both | AD | 2019 | 47.6798 | 25.4437 | 79.2199 |
| Viet Nam | Both | AD | 2019 | 142.0819 | 76.0670 | 235.6949 |
| Rwanda | Both | AD | 2019 | 30.0303 | 16.0893 | 50.7356 |
| Netherlands | Both | AD | 2019 | 164.2275 | 88.4382 | 274.4218 |
| Lebanon | Both | AD | 2019 | 59.3195 | 32.1069 | 99.3867 |
| Hungary | Both | AD | 2019 | 120.0194 | 64.4981 | 204.1400 |
| Libya | Both | AD | 2019 | 58.9793 | 31.9134 | 98.8368 |
| Saint Kitts and Nevis | Both | AD | 2019 | 85.1165 | 45.7258 | 144.2737 |
| Ghana | Both | AD | 2019 | 38.6571 | 20.7609 | 65.8550 |
| Denmark | Both | AD | 2019 | 204.2002 | 107.5317 | 344.3806 |
| Tokelau | Both | AD | 2019 | 132.7037 | 70.9989 | 222.4123 |
| Bahamas | Both | AD | 2019 | 85.2501 | 45.5782 | 144.1975 |
| Yemen | Both | AD | 2019 | 58.7110 | 31.8217 | 97.6110 |
| Qatar | Both | AD | 2019 | 56.8827 | 30.2003 | 96.1936 |
| Nepal | Both | AD | 2019 | 80.7778 | 42.5271 | 134.5860 |
| Gambia | Both | AD | 2019 | 48.4057 | 25.7987 | 81.6625 |
| Puerto Rico | Both | AD | 2019 | 85.1626 | 45.4057 | 142.4128 |
| Mauritius | Both | AD | 2019 | 140.6351 | 75.8683 | 235.5081 |
| Niger | Both | AD | 2019 | 48.3057 | 26.0492 | 80.8497 |
| Dominican Republic | Both | AD | 2019 | 85.1219 | 45.6931 | 143.2435 |
| Jamaica | Both | AD | 2019 | 84.9514 | 46.0248 | 144.2332 |
| Afghanistan | Both | AD | 2019 | 58.5668 | 31.8565 | 99.2375 |
| United Republic of Tanzania | Both | AD | 2019 | 63.4250 | 34.1755 | 107.2581 |
| Czechia | Both | AD | 2019 | 87.5314 | 46.3248 | 150.6408 |
| Saudi Arabia | Both | AD | 2019 | 58.3551 | 31.5464 | 99.8195 |
| Sweden | Both | AD | 2019 | 165.3982 | 89.1684 | 276.6266 |
| Antigua and Barbuda | Both | AD | 2019 | 85.0559 | 46.1163 | 140.9338 |
| Croatia | Both | AD | 2019 | 87.6606 | 46.9586 | 147.2336 |
| India | Both | AD | 2019 | 80.1125 | 42.9803 | 134.1143 |
| Austria | Both | AD | 2019 | 163.5719 | 87.9494 | 273.9430 |
| Peru | Both | AD | 2019 | 77.1285 | 41.5320 | 129.5188 |
| Costa Rica | Both | AD | 2019 | 63.9999 | 34.2709 | 109.5032 |
| New Zealand | Both | AD | 2019 | 149.1620 | 79.7490 | 250.3366 |
| C么te d'Ivoire | Both | AD | 2019 | 48.1345 | 26.0854 | 80.9354 |
| Germany | Both | AD | 2019 | 160.0458 | 84.8055 | 268.0570 |
| Congo | Both | AD | 2019 | 47.5180 | 25.4536 | 80.0143 |
| Australia | Both | AD | 2019 | 110.4788 | 58.9953 | 186.9873 |
| Bhutan | Both | AD | 2019 | 80.0084 | 42.4449 | 132.6566 |
| Kenya | Both | AD | 2019 | 40.4694 | 21.9450 | 67.9899 |
| South Africa | Both | AD | 2019 | 47.8485 | 25.3588 | 80.7976 |
| Kuwait | Both | AD | 2019 | 58.9774 | 31.7422 | 100.0462 |
| Bangladesh | Both | AD | 2019 | 80.7704 | 43.1498 | 135.1517 |
| Estonia | Both | AD | 2019 | 212.9040 | 113.1750 | 359.9958 |
| Lesotho | Both | AD | 2019 | 47.6077 | 25.5680 | 80.6904 |
| United Kingdom | Both | AD | 2019 | 187.3057 | 100.4144 | 313.9783 |
| Egypt | Both | AD | 2019 | 37.7030 | 20.4226 | 64.1700 |
| Venezuela (Bolivarian Republic of) | Both | AD | 2019 | 63.8336 | 34.3093 | 107.4166 |
| Ukraine | Both | AD | 2019 | 95.2065 | 51.2564 | 162.1088 |
| Portugal | Both | AD | 2019 | 164.3103 | 88.0667 | 276.4971 |
| Cook Islands | Both | AD | 2019 | 135.3353 | 73.4098 | 227.7658 |
| Belize | Both | AD | 2019 | 85.0193 | 45.8208 | 142.7687 |
| El Salvador | Both | AD | 2019 | 63.8682 | 34.6687 | 107.3286 |
| Democratic Republic of the Congo | Both | AD | 2019 | 47.4721 | 25.5397 | 80.5727 |
| Mexico | Both | AD | 2019 | 64.4661 | 34.9441 | 108.0418 |
| Central African Republic | Both | AD | 2019 | 47.4660 | 25.8320 | 80.4204 |
| North Macedonia | Both | AD | 2019 | 87.4596 | 47.2955 | 147.3713 |
| Nicaragua | Both | AD | 2019 | 63.8803 | 34.4572 | 107.4257 |
| Uganda | Both | AD | 2019 | 63.8755 | 34.5594 | 107.0753 |
| Togo | Both | AD | 2019 | 48.4172 | 26.0597 | 82.1250 |
| Mozambique | Both | AD | 2019 | 45.5986 | 24.7316 | 76.4476 |
| Greece | Both | AD | 2019 | 130.6071 | 70.6727 | 223.6795 |
| Oman | Both | AD | 2019 | 57.8925 | 31.2595 | 97.7608 |
| Spain | Both | AD | 2019 | 149.1566 | 80.4904 | 252.6850 |
| Cambodia | Both | AD | 2019 | 142.0115 | 75.6823 | 238.5349 |
| Botswana | Both | AD | 2019 | 47.6801 | 25.6384 | 80.1402 |
| Angola | Both | AD | 2019 | 47.8624 | 25.1719 | 80.6442 |
| Honduras | Both | AD | 2019 | 63.7528 | 34.0997 | 107.5540 |
| Sudan | Both | AD | 2019 | 59.0269 | 31.8313 | 99.1403 |
| Switzerland | Both | AD | 2019 | 163.7963 | 87.4876 | 276.3320 |
| Morocco | Both | AD | 2019 | 59.1848 | 31.7047 | 99.6468 |
| Palau | Both | AD | 2019 | 128.1297 | 68.5570 | 213.8304 |
| Turkey | Both | AD | 2019 | 108.2500 | 57.4083 | 181.5264 |
| Sierra Leone | Both | AD | 2019 | 48.3316 | 26.1661 | 81.6922 |
| Belarus | Both | AD | 2019 | 92.6235 | 49.4971 | 156.6959 |
| Serbia | Both | AD | 2019 | 87.6531 | 46.9738 | 148.2000 |
| Syrian Arab Republic | Both | AD | 2019 | 59.3255 | 31.9611 | 99.7169 |
| Djibouti | Both | AD | 2019 | 45.4376 | 24.2617 | 76.3873 |
| Madagascar | Both | AD | 2019 | 45.7042 | 24.2004 | 77.2919 |
| USA | Both | AD | 2019 | 137.1715 | 73.1511 | 230.1534 |
| Guinea | Both | AD | 2019 | 48.4458 | 25.8198 | 81.9522 |
| Andorra | Both | AD | 2019 | 163.7098 | 87.3282 | 275.6145 |
| Guatemala | Both | AD | 2019 | 63.7473 | 34.6363 | 107.2714 |
| Colombia | Both | AD | 2019 | 63.8037 | 34.4502 | 106.0695 |
| United States Virgin Islands | Both | AD | 2019 | 85.3065 | 45.8430 | 143.0783 |
| Indonesia | Both | AD | 2019 | 162.0031 | 86.7037 | 271.7256 |
| Singapore | Both | AD | 2019 | 159.8013 | 86.3599 | 270.3244 |
| Saint Lucia | Both | AD | 2019 | 84.9595 | 46.1466 | 143.5748 |
| Saint Vincent and the Grenadines | Both | AD | 2019 | 84.7981 | 45.3415 | 142.4975 |
| Zambia | Both | AD | 2019 | 45.5171 | 24.3252 | 75.9900 |
| Guam | Both | AD | 2019 | 132.2441 | 70.3925 | 222.3005 |
| Bahrain | Both | AD | 2019 | 58.1826 | 31.5336 | 98.8961 |
| Canada | Both | AD | 2019 | 87.5385 | 46.6985 | 147.9257 |
| Finland | Both | AD | 2019 | 163.8553 | 86.6453 | 276.2563 |
| Tunisia | Both | AD | 2019 | 59.3880 | 32.1047 | 99.6242 |
| Eritrea | Both | AD | 2019 | 45.5742 | 24.6582 | 77.2845 |
| Namibia | Both | AD | 2019 | 47.8112 | 25.3636 | 79.8977 |
| Nigeria | Both | AD | 2019 | 48.5641 | 26.0316 | 81.8588 |
| Burundi | Both | AD | 2019 | 45.6105 | 24.2512 | 76.4857 |
| Palestine | Both | AD | 2019 | 58.9635 | 31.7442 | 98.0187 |
| Ethiopia | Both | AD | 2019 | 41.2123 | 21.8523 | 69.4700 |
| Comoros | Both | AD | 2019 | 45.7535 | 24.3105 | 77.3083 |
| Monaco | Both | AD | 2019 | 164.6342 | 87.9268 | 278.0872 |
| Seychelles | Both | AD | 2019 | 137.6228 | 73.7066 | 228.7243 |
| Guinea-Bissau | Both | AD | 2019 | 48.4798 | 25.8854 | 82.3777 |
| Zimbabwe | Both | AD | 2019 | 47.9751 | 25.2440 | 81.5665 |
| Somalia | Both | AD | 2019 | 45.4484 | 24.1454 | 77.0571 |
| Chad | Both | AD | 2019 | 48.2952 | 26.0973 | 82.2153 |
| Algeria | Both | AD | 2019 | 59.2149 | 31.5134 | 98.7093 |
| Liberia | Both | AD | 2019 | 48.2087 | 26.0177 | 81.2661 |
| Burkina Faso | Both | AD | 2019 | 48.3817 | 26.2323 | 82.3033 |
| Nauru | Both | AD | 2019 | 133.7200 | 70.1086 | 224.5667 |
| Mali | Both | AD | 2019 | 51.7722 | 27.8687 | 88.0611 |
| Niue | Both | AD | 2019 | 132.8282 | 71.0256 | 223.1428 |
| Benin | Both | AD | 2019 | 48.5050 | 25.9703 | 82.4399 |
| American Samoa | Both | AD | 2019 | 133.1873 | 70.7742 | 223.4661 |
| Cameroon | Both | AD | 2019 | 56.5758 | 30.6615 | 95.2240 |
| Northern Mariana Islands | Both | AD | 2019 | 132.0370 | 71.1184 | 223.0104 |
| Cabo Verde | Both | AD | 2019 | 48.6880 | 26.1252 | 82.7689 |
| South Sudan | Both | AD | 2019 | 45.3707 | 23.8381 | 75.6683 |
|  |  |  |  |  |  |  |

**Table14 Incidence of CD stratified by sex in 1990~2019, Page 59-61**

| **location_name** | **sex_name** | **cause_name** | **year** | **Age-standardised incidence rate (per 100 000 person-years)** | **95%CI(lower)** | **95%CI(upper)** |
| --- | --- | --- | --- | --- | --- | --- |
| Global | Male | CD | 1990 | 2774.1544 | 2169.1887 | 3395.0065 |
| Global | Female | CD | 1990 | 3333.5760 | 2626.4224 | 4085.6360 |
| Global | Both | CD | 1990 | 3053.2513 | 2401.4428 | 3741.1057 |
| Global | Male | CD | 1993 | 2750.1482 | 2150.8778 | 3361.8992 |
| Global | Female | CD | 1993 | 3294.3089 | 2594.3110 | 4037.0366 |
| Global | Both | CD | 1993 | 3021.3173 | 2378.3815 | 3696.4403 |
| Global | Male | CD | 1992 | 2756.2668 | 2155.4930 | 3370.7891 |
| Global | Female | CD | 1992 | 3304.8284 | 2602.8592 | 4052.1173 |
| Global | Both | CD | 1992 | 3029.6938 | 2384.4471 | 3708.6402 |
| Global | Male | CD | 1994 | 2747.1605 | 2148.8078 | 3358.6482 |
| Global | Female | CD | 1994 | 3287.9376 | 2589.1369 | 4027.5215 |
| Global | Both | CD | 1994 | 3016.6312 | 2375.0448 | 3688.9839 |
| Global | Male | CD | 1991 | 2764.6827 | 2161.9605 | 3382.5060 |
| Global | Female | CD | 1991 | 3318.3696 | 2613.9396 | 4067.4662 |
| Global | Both | CD | 1991 | 3040.7683 | 2392.4908 | 3724.1324 |
| Global | Male | CD | 1995 | 2748.0584 | 2149.5762 | 3359.8115 |
| Global | Female | CD | 1995 | 3286.9751 | 2588.0428 | 4025.3893 |
| Global | Both | CD | 1995 | 3016.6461 | 2375.4466 | 3687.8294 |
| Global | Male | CD | 1996 | 2751.2718 | 2152.0424 | 3363.6652 |
| Global | Female | CD | 1996 | 3289.3001 | 2589.7353 | 4028.7165 |
| Global | Both | CD | 1996 | 3019.4934 | 2377.6037 | 3691.5227 |
| Global | Male | CD | 1998 | 2756.8628 | 2156.7099 | 3370.9019 |
| Global | Female | CD | 1998 | 3293.8229 | 2593.4540 | 4035.5334 |
| Global | Both | CD | 1998 | 3024.7890 | 2381.7751 | 3699.5477 |
| Global | Male | CD | 1997 | 2754.1169 | 2154.3719 | 3367.4075 |
| Global | Female | CD | 1997 | 3291.5414 | 2591.5395 | 4032.0191 |
| Global | Both | CD | 1997 | 3022.1483 | 2379.6155 | 3695.3822 |
| Global | Male | CD | 1999 | 2759.6533 | 2158.9798 | 3374.1936 |
| Global | Female | CD | 1999 | 3296.0794 | 2595.3458 | 4039.1047 |
| Global | Both | CD | 1999 | 3027.4407 | 2383.9948 | 3703.6518 |
| Global | Male | CD | 2000 | 2762.0764 | 2160.8496 | 3376.8484 |
| Global | Female | CD | 2000 | 3298.0440 | 2596.9584 | 4042.1742 |
| Global | Both | CD | 2000 | 3029.7717 | 2385.6847 | 3707.1497 |
| Global | Male | CD | 2001 | 2764.5226 | 2162.5373 | 3378.8305 |
| Global | Female | CD | 2001 | 3300.0142 | 2598.4141 | 4045.1275 |
| Global | Both | CD | 2001 | 3032.1113 | 2387.1889 | 3710.2630 |
| Global | Male | CD | 2002 | 2766.7415 | 2164.3162 | 3380.6609 |
| Global | Female | CD | 2002 | 3301.7023 | 2599.6275 | 4046.8240 |
| Global | Both | CD | 2002 | 3034.1943 | 2388.3230 | 3713.1585 |
| Global | Male | CD | 2004 | 2771.0344 | 2167.2494 | 3383.4343 |
| Global | Female | CD | 2004 | 3304.8427 | 2601.7779 | 4049.3055 |
| Global | Both | CD | 2004 | 3038.1394 | 2390.7594 | 3717.9992 |
| Global | Male | CD | 2003 | 2768.9433 | 2165.8973 | 3382.2964 |
| Global | Female | CD | 2003 | 3303.3472 | 2600.7422 | 4048.2660 |
| Global | Both | CD | 2003 | 3036.2404 | 2389.4643 | 3715.7878 |
| Global | Male | CD | 2006 | 2775.0024 | 2169.0895 | 3388.8512 |
| Global | Female | CD | 2006 | 3307.6496 | 2603.9193 | 4054.6827 |
| Global | Both | CD | 2006 | 3041.7112 | 2392.5883 | 3721.8244 |
| Global | Male | CD | 2007 | 2777.0813 | 2170.0188 | 3392.2958 |
| Global | Female | CD | 2007 | 3309.0218 | 2604.9408 | 4057.3042 |
| Global | Both | CD | 2007 | 3043.4927 | 2393.5569 | 3724.7296 |
| Global | Male | CD | 2005 | 2772.8906 | 2168.3082 | 3385.6566 |
| Global | Female | CD | 2005 | 3306.1212 | 2602.7246 | 4051.9401 |
| Global | Both | CD | 2005 | 3039.8141 | 2391.7712 | 3719.8787 |
| Global | Male | CD | 2008 | 2779.0463 | 2170.8854 | 3395.6986 |
| Global | Female | CD | 2008 | 3310.3430 | 2606.0009 | 4059.4934 |
| Global | Both | CD | 2008 | 3045.1923 | 2394.2071 | 3727.5129 |
| Global | Male | CD | 2011 | 2785.3299 | 2173.4135 | 3405.8248 |
| Global | Female | CD | 2011 | 3314.6459 | 2609.4114 | 4062.6331 |
| Global | Both | CD | 2011 | 3050.7401 | 2395.7477 | 3735.8672 |
| Global | Male | CD | 2010 | 2782.9998 | 2172.4793 | 3402.3105 |
| Global | Female | CD | 2010 | 3312.9613 | 2608.1376 | 4061.5414 |
| Global | Both | CD | 2010 | 3048.6551 | 2395.0663 | 3732.8318 |
| Global | Male | CD | 2009 | 2780.9791 | 2171.6171 | 3398.9850 |
| Global | Female | CD | 2009 | 3311.6523 | 2607.1015 | 4060.4185 |
| Global | Both | CD | 2009 | 3046.8926 | 2394.5223 | 3730.0091 |
| Global | Male | CD | 2012 | 2787.5576 | 2174.3355 | 3409.1085 |
| Global | Female | CD | 2012 | 3316.2071 | 2610.6027 | 4063.6190 |
| Global | Both | CD | 2012 | 3052.7088 | 2396.4095 | 3738.5064 |
| Global | Male | CD | 2013 | 2789.8271 | 2175.3892 | 3412.2351 |
| Global | Female | CD | 2013 | 3317.8578 | 2611.2626 | 4064.5362 |
| Global | Both | CD | 2013 | 3054.7446 | 2397.2306 | 3741.2038 |
| Global | Male | CD | 2014 | 2792.0409 | 2176.4797 | 3415.0929 |
| Global | Female | CD | 2014 | 3319.4649 | 2612.6596 | 4065.3423 |
| Global | Both | CD | 2014 | 3056.7238 | 2398.1493 | 3743.8446 |
| Global | Male | CD | 2015 | 2794.1409 | 2177.6485 | 3417.7316 |
| Global | Female | CD | 2015 | 3320.8623 | 2614.4037 | 4066.0566 |
| Global | Both | CD | 2015 | 3058.5307 | 2399.2855 | 3746.0797 |
| Global | Male | CD | 2016 | 2796.3493 | 2179.5766 | 3420.2965 |
| Global | Female | CD | 2016 | 3322.5089 | 2616.0574 | 4067.2030 |
| Global | Both | CD | 2016 | 3060.5074 | 2400.9523 | 3748.6680 |
| Global | Male | CD | 2019 | 2802.7859 | 2182.8659 | 3426.8620 |
| Global | Female | CD | 2019 | 3326.8553 | 2618.9106 | 4070.2312 |
| Global | Both | CD | 2019 | 3066.0421 | 2405.3768 | 3755.3806 |
| Global | Male | CD | 2018 | 2800.7195 | 2181.5732 | 3424.7219 |
| Global | Female | CD | 2018 | 3325.4851 | 2617.8017 | 4068.6483 |
| Global | Both | CD | 2018 | 3064.2790 | 2403.8359 | 3753.1442 |
| Global | Male | CD | 2017 | 2798.5387 | 2180.3186 | 3422.5866 |
| Global | Female | CD | 2017 | 3324.0104 | 2616.5891 | 4067.8576 |
| Global | Both | CD | 2017 | 3062.4013 | 2402.3791 | 3750.8906 |
|  |  |  |  |  |  |  |

**Table15 Incidence of CD stratified by SDI in 1990~2019, Page 62-72**

| **location_name** | **sex_name** | **cause_name** | **year** | **Age-standardised incidence rate (per 100 000 person-years)** | **95%CI(lower)** | **95%CI(upper)** |
| --- | --- | --- | --- | --- | --- | --- |
| Low-middle SDI | Male | CD | 1990 | 2942.3690 | 2287.0978 | 3610.2043 |
| Low-middle SDI | Female | CD | 1990 | 3398.5032 | 2666.1627 | 4184.6200 |
| Low-middle SDI | Both | CD | 1990 | 3167.3349 | 2468.3855 | 3895.8755 |
| High-middle SDI | Male | CD | 1990 | 2611.7843 | 2047.1502 | 3191.7962 |
| High-middle SDI | Female | CD | 1990 | 3435.2111 | 2712.4927 | 4192.0039 |
| High-middle SDI | Both | CD | 1990 | 3027.5031 | 2385.9513 | 3692.8321 |
| High SDI | Male | CD | 1990 | 2309.5629 | 1833.6548 | 2810.7542 |
| High SDI | Female | CD | 1990 | 2945.3435 | 2313.8490 | 3629.0688 |
| High SDI | Both | CD | 1990 | 2625.4592 | 2068.7619 | 3216.9210 |
| Middle SDI | Male | CD | 1990 | 3188.1593 | 2476.5316 | 3915.0785 |
| Middle SDI | Female | CD | 1990 | 3699.5362 | 2913.4639 | 4526.6603 |
| Middle SDI | Both | CD | 1990 | 3443.6662 | 2689.3846 | 4205.0973 |
| Low SDI | Male | CD | 1990 | 2727.3707 | 2130.9103 | 3329.1432 |
| Low SDI | Female | CD | 1990 | 3120.0442 | 2445.4662 | 3824.6287 |
| Low SDI | Both | CD | 1990 | 2922.8883 | 2291.0991 | 3579.7625 |
| High-middle SDI | Male | CD | 1991 | 2605.1133 | 2041.9066 | 3183.6882 |
| High-middle SDI | Female | CD | 1991 | 3432.6327 | 2711.6862 | 4186.0602 |
| High-middle SDI | Both | CD | 1991 | 3022.5588 | 2383.2850 | 3684.8595 |
| Middle SDI | Male | CD | 1991 | 2769.0588 | 2153.8623 | 3390.0186 |
| Middle SDI | Female | CD | 1991 | 3698.7599 | 2912.7605 | 4525.7109 |
| Middle SDI | Both | CD | 1991 | 3233.1676 | 2526.9286 | 3946.4557 |
| High SDI | Male | CD | 1991 | 2245.5271 | 1786.7347 | 2730.1359 |
| High SDI | Female | CD | 1991 | 2861.8071 | 2249.0787 | 3525.0222 |
| High SDI | Both | CD | 1991 | 2551.4410 | 2012.0363 | 3128.8577 |
| Low-middle SDI | Male | CD | 1991 | 2899.6347 | 2253.0295 | 3557.4915 |
| Low-middle SDI | Female | CD | 1991 | 3398.4538 | 2666.2330 | 4184.1362 |
| Low-middle SDI | Both | CD | 1991 | 3145.7596 | 2451.6274 | 3868.9118 |
| Low SDI | Male | CD | 1991 | 2410.0081 | 1883.2665 | 2945.0156 |
| Low SDI | Female | CD | 1991 | 3119.6610 | 2445.0317 | 3823.7797 |
| Low SDI | Both | CD | 1991 | 2763.5158 | 2165.8046 | 3384.0850 |
| High-middle SDI | Male | CD | 1992 | 2608.2874 | 2044.6593 | 3186.9688 |
| High-middle SDI | Female | CD | 1992 | 3430.6596 | 2711.5243 | 4181.1673 |
| High-middle SDI | Both | CD | 1992 | 3022.9120 | 2384.4553 | 3682.9893 |
| High SDI | Male | CD | 1992 | 2187.3246 | 1741.4321 | 2661.2219 |
| High SDI | Female | CD | 1992 | 2785.7788 | 2191.4667 | 3424.3122 |
| High SDI | Both | CD | 1992 | 2484.1535 | 1962.8068 | 3046.7233 |
| Low-middle SDI | Male | CD | 1992 | 2899.7460 | 2252.9101 | 3557.6065 |
| Low-middle SDI | Female | CD | 1992 | 3398.3661 | 2666.2596 | 4183.6388 |
| Low-middle SDI | Both | CD | 1992 | 3145.8687 | 2451.8805 | 3868.7902 |
| Middle SDI | Male | CD | 1993 | 2782.0065 | 2163.7233 | 3406.9631 |
| Middle SDI | Female | CD | 1993 | 3697.2096 | 2911.3214 | 4523.2660 |
| Middle SDI | Both | CD | 1993 | 3238.8294 | 2531.3283 | 3953.8354 |
| High-middle SDI | Male | CD | 1993 | 2611.6653 | 2047.5722 | 3189.8404 |
| High-middle SDI | Female | CD | 1993 | 3429.1062 | 2711.2649 | 4177.5155 |
| High-middle SDI | Both | CD | 1993 | 3023.6753 | 2385.7510 | 3681.7316 |
| Low SDI | Male | CD | 1992 | 2410.2930 | 1883.4987 | 2945.1901 |
| Low SDI | Female | CD | 1992 | 3119.5144 | 2444.8125 | 3823.1016 |
| Low SDI | Both | CD | 1992 | 2763.4236 | 2165.6734 | 3383.9958 |
| High SDI | Male | CD | 1993 | 2140.4389 | 1704.7411 | 2605.6679 |
| High SDI | Female | CD | 1993 | 2724.2732 | 2142.4144 | 3342.8534 |
| High SDI | Both | CD | 1993 | 2429.8106 | 1923.0898 | 2974.0198 |
| Middle SDI | Male | CD | 1992 | 2804.1933 | 2180.8843 | 3434.3577 |
| Middle SDI | Female | CD | 1992 | 3697.9646 | 2912.0330 | 4524.5933 |
| Middle SDI | Both | CD | 1992 | 3250.2468 | 2540.2662 | 3967.4751 |
| Low-middle SDI | Male | CD | 1993 | 2899.3371 | 2252.5141 | 3557.0178 |
| Low-middle SDI | Female | CD | 1993 | 3398.2417 | 2666.1620 | 4183.0963 |
| Low-middle SDI | Both | CD | 1993 | 3145.7295 | 2451.8927 | 3868.4052 |
| Low SDI | Male | CD | 1993 | 2410.3857 | 1883.5485 | 2945.1791 |
| Low SDI | Female | CD | 1993 | 3119.5201 | 2444.8026 | 3822.6841 |
| Low SDI | Both | CD | 1993 | 2763.3315 | 2165.5204 | 3383.8565 |
| High-middle SDI | Male | CD | 1994 | 2615.8692 | 2050.9718 | 3194.0582 |
| High-middle SDI | Female | CD | 1994 | 3428.1081 | 2711.2553 | 4175.0500 |
| High-middle SDI | Both | CD | 1994 | 3025.1740 | 2387.4785 | 3683.1357 |
| High SDI | Male | CD | 1994 | 2109.7553 | 1680.2334 | 2569.2631 |
| High SDI | Female | CD | 1994 | 2683.9936 | 2114.4200 | 3292.8726 |
| High SDI | Both | CD | 1994 | 2394.2091 | 1894.8948 | 2926.2885 |
| Low-middle SDI | Male | CD | 1994 | 2899.3678 | 2252.7074 | 3556.9556 |
| Low-middle SDI | Female | CD | 1994 | 3398.1233 | 2665.9693 | 4182.6098 |
| Low-middle SDI | Both | CD | 1994 | 3145.8789 | 2452.0946 | 3868.4216 |
| Low SDI | Male | CD | 1994 | 2410.1300 | 1883.3625 | 2944.7478 |
| Low SDI | Female | CD | 1994 | 3119.1271 | 2444.4661 | 3821.9839 |
| Low SDI | Both | CD | 1994 | 2763.0844 | 2165.3690 | 3383.3739 |
| Middle SDI | Male | CD | 1994 | 2818.8337 | 2192.1934 | 3453.4648 |
| Middle SDI | Female | CD | 1994 | 3696.4094 | 2910.5843 | 4521.6736 |
| Middle SDI | Both | CD | 1994 | 3256.8561 | 2545.1340 | 3976.0157 |
| Low SDI | Male | CD | 1995 | 2409.7528 | 1883.0925 | 2944.0380 |
| Low SDI | Female | CD | 1995 | 3118.5108 | 2444.0086 | 3820.8081 |
| Low SDI | Both | CD | 1995 | 2762.7723 | 2165.2573 | 3382.8220 |
| Middle SDI | Male | CD | 1995 | 2827.2677 | 2198.6914 | 3464.3658 |
| Middle SDI | Female | CD | 1995 | 3695.5012 | 2910.0299 | 4519.8352 |
| Middle SDI | Both | CD | 1995 | 3260.7956 | 2548.1464 | 3981.2030 |
| Low-middle SDI | Male | CD | 1995 | 2899.1472 | 2252.7506 | 3556.4599 |
| Low-middle SDI | Female | CD | 1995 | 3398.0496 | 2666.2088 | 4182.1039 |
| Low-middle SDI | Both | CD | 1995 | 3145.9648 | 2452.2782 | 3868.4008 |
| High-middle SDI | Male | CD | 1995 | 2620.4711 | 2053.5088 | 3197.6773 |
| High-middle SDI | Female | CD | 1995 | 3427.6942 | 2711.5499 | 4174.0538 |
| High-middle SDI | Both | CD | 1995 | 3027.2759 | 2389.3184 | 3686.2768 |
| High SDI | Male | CD | 1995 | 2100.9957 | 1672.9931 | 2556.2014 |
| High SDI | Female | CD | 1995 | 2672.1414 | 2106.8831 | 3281.4747 |
| High SDI | Both | CD | 1995 | 2383.7556 | 1885.2221 | 2915.6104 |
| High-middle SDI | Male | CD | 1996 | 2625.4113 | 2056.8214 | 3202.3993 |
| High-middle SDI | Female | CD | 1996 | 3428.1796 | 2711.5376 | 4174.8802 |
| High-middle SDI | Both | CD | 1996 | 3029.9947 | 2390.9966 | 3690.3701 |
| High SDI | Male | CD | 1996 | 2104.8507 | 1675.7201 | 2561.5118 |
| High SDI | Female | CD | 1996 | 2676.8466 | 2110.8203 | 3287.3755 |
| High SDI | Both | CD | 1996 | 2387.8860 | 1887.4601 | 2920.3417 |
| Low SDI | Male | CD | 1997 | 2408.7862 | 1882.2240 | 2942.9582 |
| Low SDI | Female | CD | 1997 | 3117.5543 | 2443.7094 | 3819.5406 |
| Low SDI | Both | CD | 1997 | 2762.2280 | 2165.2485 | 3381.7885 |
| Low-middle SDI | Male | CD | 1996 | 2898.6364 | 2252.6169 | 3555.5316 |
| Low-middle SDI | Female | CD | 1996 | 3397.9646 | 2666.6461 | 4181.6075 |
| Low-middle SDI | Both | CD | 1996 | 3145.9338 | 2452.3391 | 3868.3391 |
| Low SDI | Male | CD | 1996 | 2409.3829 | 1882.7689 | 2943.5831 |
| Low SDI | Female | CD | 1996 | 3117.9540 | 2443.8273 | 3820.0235 |
| Low SDI | Both | CD | 1996 | 2762.5011 | 2165.2524 | 3382.2741 |
| Middle SDI | Male | CD | 1997 | 2814.2748 | 2188.8450 | 3449.3776 |
| Middle SDI | Female | CD | 1997 | 3693.6051 | 2909.0987 | 4516.0113 |
| Middle SDI | Both | CD | 1997 | 3253.9810 | 2542.7508 | 3973.9138 |
| Middle SDI | Male | CD | 1996 | 2806.3825 | 2182.5817 | 3439.1208 |
| Middle SDI | Female | CD | 1996 | 3694.5325 | 2909.5070 | 4517.8693 |
| Middle SDI | Both | CD | 1996 | 3250.1943 | 2539.8232 | 3968.7377 |
| High-middle SDI | Male | CD | 1998 | 2633.7561 | 2062.9507 | 3213.4598 |
| High-middle SDI | Female | CD | 1998 | 3429.3474 | 2712.0017 | 4177.1036 |
| High-middle SDI | Both | CD | 1998 | 3034.7679 | 2394.0706 | 3696.8308 |
| High-middle SDI | Male | CD | 1997 | 2629.8894 | 2059.9585 | 3208.2765 |
| High-middle SDI | Female | CD | 1997 | 3428.8612 | 2711.9163 | 4175.8682 |
| High-middle SDI | Both | CD | 1997 | 3032.5893 | 2392.5798 | 3693.9599 |
| High SDI | Male | CD | 1997 | 2108.8892 | 1678.3124 | 2567.2423 |
| High SDI | Female | CD | 1997 | 2681.7828 | 2114.7295 | 3293.1837 |
| High SDI | Both | CD | 1997 | 2392.2633 | 1889.7919 | 2926.6570 |
| High SDI | Male | CD | 1998 | 2113.1825 | 1680.9800 | 2573.2835 |
| High SDI | Female | CD | 1998 | 2686.9916 | 2118.9276 | 3299.1776 |
| High SDI | Both | CD | 1998 | 2396.9109 | 1893.3733 | 2935.5005 |
| Low-middle SDI | Male | CD | 1997 | 2898.2587 | 2252.6023 | 3554.7743 |
| Low-middle SDI | Female | CD | 1997 | 3397.8290 | 2666.9401 | 4181.1412 |
| Low-middle SDI | Both | CD | 1997 | 3145.9730 | 2452.4566 | 3868.4717 |
| Low-middle SDI | Male | CD | 1998 | 2897.7984 | 2252.5172 | 3554.0196 |
| Low-middle SDI | Female | CD | 1998 | 3397.6463 | 2667.1074 | 4180.6439 |
| Low-middle SDI | Both | CD | 1998 | 3145.9680 | 2452.5504 | 3868.6037 |
| Low SDI | Male | CD | 1998 | 2408.0431 | 1881.5314 | 2942.0565 |
| Low SDI | Female | CD | 1998 | 3117.3439 | 2443.6535 | 3819.3065 |
| Low SDI | Both | CD | 1998 | 2762.0049 | 2165.2613 | 3381.2913 |
| Middle SDI | Male | CD | 1998 | 2821.4606 | 2194.6714 | 3458.6863 |
| Middle SDI | Female | CD | 1998 | 3692.7218 | 2908.7521 | 4514.3135 |
| Middle SDI | Both | CD | 1998 | 3257.4949 | 2545.4743 | 3978.7785 |
| High-middle SDI | Male | CD | 1999 | 2637.7549 | 2066.0760 | 3218.7642 |
| High-middle SDI | Female | CD | 1999 | 3429.7806 | 2712.0510 | 4178.3503 |
| High-middle SDI | Both | CD | 1999 | 3036.9295 | 2395.9377 | 3699.6731 |
| High-middle SDI | Male | CD | 2000 | 2641.1298 | 2068.5857 | 3222.9971 |
| High-middle SDI | Female | CD | 2000 | 3429.3927 | 2711.4620 | 4178.5188 |
| High-middle SDI | Both | CD | 2000 | 3038.4042 | 2397.3037 | 3701.9678 |
| High SDI | Male | CD | 1999 | 2117.5908 | 1683.8459 | 2579.1950 |
| High SDI | Female | CD | 1999 | 2692.3513 | 2123.3518 | 3305.4078 |
| High SDI | Both | CD | 1999 | 2401.7405 | 1898.1868 | 2944.7114 |
| High SDI | Male | CD | 2000 | 2121.8108 | 1686.2261 | 2584.0025 |
| High SDI | Female | CD | 2000 | 2697.6520 | 2127.6314 | 3311.3580 |
| High SDI | Both | CD | 2000 | 2406.4322 | 1902.4129 | 2953.6433 |
| Low-middle SDI | Male | CD | 1999 | 2897.5191 | 2252.5691 | 3553.5401 |
| Low-middle SDI | Female | CD | 1999 | 3397.4530 | 2667.2698 | 4180.0987 |
| Low-middle SDI | Both | CD | 1999 | 3146.0356 | 2452.6561 | 3868.8254 |
| Low SDI | Male | CD | 1999 | 2407.3129 | 1880.8620 | 2941.0548 |
| Low SDI | Female | CD | 1999 | 3117.1729 | 2443.6099 | 3819.2013 |
| Low SDI | Both | CD | 1999 | 2761.8300 | 2165.2480 | 3381.1275 |
| Low-middle SDI | Male | CD | 2000 | 2896.7006 | 2252.1483 | 3552.4696 |
| Low-middle SDI | Female | CD | 2000 | 3397.1985 | 2667.4319 | 4179.4235 |
| Low-middle SDI | Both | CD | 2000 | 3145.8012 | 2452.4999 | 3868.6107 |
| Middle SDI | Male | CD | 1999 | 2828.0104 | 2200.1392 | 3467.2501 |
| Middle SDI | Female | CD | 1999 | 3691.7502 | 2908.2852 | 4512.6475 |
| Middle SDI | Both | CD | 1999 | 3260.6373 | 2547.8685 | 3982.8982 |
| Low SDI | Male | CD | 2000 | 2406.6466 | 1880.2655 | 2940.4675 |
| Low SDI | Female | CD | 2000 | 3117.0173 | 2443.6016 | 3819.0875 |
| Low SDI | Both | CD | 2000 | 2761.7158 | 2165.2802 | 3380.9226 |
| Middle SDI | Male | CD | 2000 | 2834.0313 | 2205.0463 | 3474.9072 |
| Middle SDI | Female | CD | 2000 | 3690.6744 | 2907.7754 | 4510.8679 |
| Middle SDI | Both | CD | 2000 | 3263.4815 | 2550.0750 | 3986.7845 |
| Low SDI | Male | CD | 2001 | 2406.2919 | 1879.9066 | 2939.9065 |
| Low SDI | Female | CD | 2001 | 3116.9673 | 2443.6857 | 3819.1401 |
| Low SDI | Both | CD | 2001 | 2761.7657 | 2165.4351 | 3380.9043 |
| Middle SDI | Male | CD | 2001 | 2839.0331 | 2209.0815 | 3480.9385 |
| Middle SDI | Female | CD | 2001 | 3689.6070 | 2907.3160 | 4509.2034 |
| Middle SDI | Both | CD | 2001 | 3265.7962 | 2552.0080 | 3990.0172 |
| High-middle SDI | Male | CD | 2001 | 2644.4413 | 2070.4648 | 3227.3459 |
| High-middle SDI | Female | CD | 2001 | 3428.8644 | 2710.1871 | 4177.9151 |
| High-middle SDI | Both | CD | 2001 | 3039.7342 | 2398.7764 | 3703.4838 |
| High SDI | Male | CD | 2001 | 2125.7959 | 1688.0538 | 2590.2222 |
| High SDI | Female | CD | 2001 | 2702.8687 | 2131.6588 | 3318.9764 |
| High SDI | Both | CD | 2001 | 2410.9701 | 1906.4766 | 2960.6779 |
| Low-middle SDI | Male | CD | 2001 | 2896.0071 | 2251.8316 | 3551.4661 |
| Low-middle SDI | Female | CD | 2001 | 3396.8563 | 2667.4981 | 4178.6481 |
| Low-middle SDI | Both | CD | 2001 | 3145.6029 | 2452.5156 | 3868.3775 |
| High-middle SDI | Male | CD | 2002 | 2647.4003 | 2072.0059 | 3231.2367 |
| High-middle SDI | Female | CD | 2002 | 3428.1047 | 2708.9834 | 4176.5642 |
| High-middle SDI | Both | CD | 2002 | 3040.7499 | 2400.1234 | 3704.1721 |
| High SDI | Male | CD | 2002 | 2130.1491 | 1690.4587 | 2596.8831 |
| High SDI | Female | CD | 2002 | 2708.4434 | 2135.3361 | 3328.3083 |
| High SDI | Both | CD | 2002 | 2415.8919 | 1910.7627 | 2967.0793 |
| Low SDI | Male | CD | 2003 | 2406.0400 | 1879.5745 | 2939.6323 |
| Low SDI | Female | CD | 2003 | 3116.8972 | 2443.7682 | 3819.2324 |
| Low SDI | Both | CD | 2003 | 2762.0194 | 2165.9791 | 3381.0974 |
| Low-middle SDI | Male | CD | 2002 | 2895.4331 | 2251.6586 | 3550.6148 |
| Low-middle SDI | Female | CD | 2002 | 3396.4374 | 2667.4940 | 4177.7654 |
| Low-middle SDI | Both | CD | 2002 | 3145.4143 | 2452.4645 | 3868.0990 |
| Low SDI | Male | CD | 2002 | 2406.1377 | 1879.6779 | 2939.5565 |
| Low SDI | Female | CD | 2002 | 3116.9112 | 2443.7173 | 3819.1702 |
| Low SDI | Both | CD | 2002 | 2761.8522 | 2165.6114 | 3380.8798 |
| Middle SDI | Male | CD | 2003 | 2876.1779 | 2237.6899 | 3525.6411 |
| Middle SDI | Female | CD | 2003 | 3687.4556 | 2905.9666 | 4506.0225 |
| Middle SDI | Both | CD | 2003 | 3283.7250 | 2566.5694 | 4012.7197 |
| Middle SDI | Male | CD | 2002 | 2872.9944 | 2235.3382 | 3521.2065 |
| Middle SDI | Female | CD | 2002 | 3688.5282 | 2906.8330 | 4507.6280 |
| Middle SDI | Both | CD | 2002 | 3282.3903 | 2565.1432 | 4010.6379 |
| Low-middle SDI | Male | CD | 2005 | 2892.5265 | 2250.2697 | 3546.8948 |
| Low-middle SDI | Female | CD | 2005 | 3394.4748 | 2666.2432 | 4174.4749 |
| Low-middle SDI | Both | CD | 2005 | 3143.8884 | 2451.3868 | 3865.2692 |
| High-middle SDI | Male | CD | 2003 | 2649.7770 | 2072.8960 | 3234.1214 |
| High-middle SDI | Female | CD | 2003 | 3426.9634 | 2707.5859 | 4174.3461 |
| High-middle SDI | Both | CD | 2003 | 3041.2642 | 2401.1679 | 3704.1036 |
| High SDI | Male | CD | 2003 | 2135.1159 | 1693.6457 | 2602.9886 |
| High SDI | Female | CD | 2003 | 2714.4870 | 2140.1191 | 3338.4318 |
| High SDI | Both | CD | 2003 | 2421.3570 | 1915.3034 | 2974.5787 |
| Low SDI | Male | CD | 2005 | 2406.3898 | 1879.9235 | 2939.7691 |
| Low SDI | Female | CD | 2005 | 3116.9714 | 2443.8410 | 3819.7475 |
| Low SDI | Both | CD | 2005 | 2762.7045 | 2166.7652 | 3381.8615 |
| Low-middle SDI | Male | CD | 2003 | 2894.5535 | 2251.2359 | 3549.4131 |
| Low-middle SDI | Female | CD | 2003 | 3395.8915 | 2667.3825 | 4176.7433 |
| Low-middle SDI | Both | CD | 2003 | 3145.0093 | 2452.2282 | 3867.3841 |
| Middle SDI | Male | CD | 2005 | 2851.2114 | 2216.9313 | 3496.8343 |
| Middle SDI | Female | CD | 2005 | 3685.2179 | 2904.0787 | 4502.9021 |
| Middle SDI | Both | CD | 2005 | 3270.8255 | 2557.6175 | 3997.6323 |
| High-middle SDI | Male | CD | 2004 | 2652.0675 | 2075.3378 | 3236.7056 |
| High-middle SDI | Female | CD | 2004 | 3425.4074 | 2705.9444 | 4171.1606 |
| High-middle SDI | Both | CD | 2004 | 3041.4726 | 2400.6475 | 3704.0599 |
| High-middle SDI | Male | CD | 2005 | 2654.0469 | 2077.6639 | 3238.8131 |
| High-middle SDI | Female | CD | 2005 | 3423.3416 | 2704.7683 | 4170.4375 |
| High-middle SDI | Both | CD | 2005 | 3041.2676 | 2400.1933 | 3704.8893 |
| High SDI | Male | CD | 2005 | 2144.6387 | 1699.6999 | 2615.1546 |
| High SDI | Female | CD | 2005 | 2726.5257 | 2149.9196 | 3357.8168 |
| High SDI | Both | CD | 2005 | 2432.0472 | 1923.9477 | 2987.6302 |
| High SDI | Male | CD | 2004 | 2139.8613 | 1696.5308 | 2608.8747 |
| High SDI | Female | CD | 2004 | 2720.5172 | 2145.1364 | 3348.7512 |
| High SDI | Both | CD | 2004 | 2426.7043 | 1919.7365 | 2981.6195 |
| Low-middle SDI | Male | CD | 2004 | 2893.6018 | 2250.8445 | 3548.1620 |
| Low-middle SDI | Female | CD | 2004 | 3395.2438 | 2666.9739 | 4175.6186 |
| Low-middle SDI | Both | CD | 2004 | 3144.5136 | 2451.8511 | 3866.4343 |
| Low SDI | Male | CD | 2004 | 2406.1032 | 1879.6778 | 2939.5841 |
| Low SDI | Female | CD | 2004 | 3116.9204 | 2443.8337 | 3819.3237 |
| Low SDI | Both | CD | 2004 | 2762.3037 | 2166.3767 | 3381.3785 |
| Middle SDI | Male | CD | 2004 | 2878.8220 | 2239.2619 | 3529.6317 |
| Middle SDI | Female | CD | 2004 | 3686.3729 | 2905.1291 | 4504.3624 |
| Middle SDI | Both | CD | 2004 | 3284.7476 | 2567.9938 | 4014.3305 |
| High-middle SDI | Male | CD | 2006 | 2656.6378 | 2079.7881 | 3241.6039 |
| High-middle SDI | Female | CD | 2006 | 3421.9883 | 2704.0638 | 4170.8935 |
| High-middle SDI | Both | CD | 2006 | 3041.6625 | 2400.5699 | 3705.4370 |
| High SDI | Male | CD | 2006 | 2149.6137 | 1703.0786 | 2621.7110 |
| High SDI | Female | CD | 2006 | 2732.6744 | 2154.8080 | 3366.0616 |
| High SDI | Both | CD | 2006 | 2437.4539 | 1927.7093 | 2993.3655 |
| Low-middle SDI | Male | CD | 2007 | 2889.9916 | 2248.2923 | 3544.2601 |
| Low-middle SDI | Female | CD | 2007 | 3392.5764 | 2664.3980 | 4172.0356 |
| Low-middle SDI | Both | CD | 2007 | 3142.2535 | 2450.1241 | 3862.4087 |
| Low-middle SDI | Male | CD | 2006 | 2891.3310 | 2249.4368 | 3545.5980 |
| Low-middle SDI | Female | CD | 2006 | 3393.5751 | 2665.3796 | 4173.2568 |
| Low-middle SDI | Both | CD | 2006 | 3143.1334 | 2450.8127 | 3863.9471 |
| Low SDI | Male | CD | 2007 | 2407.2734 | 1880.6184 | 2941.0443 |
| Low SDI | Female | CD | 2007 | 3117.0280 | 2443.7688 | 3820.0362 |
| Low SDI | Both | CD | 2007 | 2763.6424 | 2167.9790 | 3382.5640 |
| Low SDI | Male | CD | 2006 | 2406.8175 | 1880.2196 | 2940.3850 |
| Low SDI | Female | CD | 2006 | 3117.0176 | 2443.8204 | 3819.9461 |
| Low SDI | Both | CD | 2006 | 2763.1718 | 2167.4323 | 3382.2740 |
| Middle SDI | Male | CD | 2007 | 2885.5251 | 2242.8609 | 3539.9532 |
| Middle SDI | Female | CD | 2007 | 3682.8985 | 2901.7291 | 4500.3939 |
| Middle SDI | Both | CD | 2007 | 3287.0339 | 2570.4665 | 4017.6222 |
| Middle SDI | Male | CD | 2006 | 2853.4378 | 2218.0960 | 3499.6855 |
| Middle SDI | Female | CD | 2006 | 3684.0562 | 2902.9435 | 4501.6746 |
| Middle SDI | Both | CD | 2006 | 3271.5871 | 2558.5860 | 3998.7053 |
| High-middle SDI | Male | CD | 2007 | 2659.5504 | 2082.3420 | 3245.1144 |
| High-middle SDI | Female | CD | 2007 | 3420.7775 | 2703.4377 | 4171.1685 |
| High-middle SDI | Both | CD | 2007 | 3042.2567 | 2400.9786 | 3707.1838 |
| High SDI | Male | CD | 2007 | 2155.3513 | 1707.1290 | 2629.1626 |
| High SDI | Female | CD | 2007 | 2739.3226 | 2159.9503 | 3375.0393 |
| High SDI | Both | CD | 2007 | 2443.3420 | 1931.6921 | 3001.3941 |
| High-middle SDI | Male | CD | 2008 | 2662.3814 | 2084.2132 | 3248.5946 |
| High-middle SDI | Female | CD | 2008 | 3419.6304 | 2702.9182 | 4171.5156 |
| High-middle SDI | Both | CD | 2008 | 3042.8580 | 2401.4468 | 3706.9679 |
| High SDI | Male | CD | 2008 | 2161.7134 | 1711.8480 | 2636.6780 |
| High SDI | Female | CD | 2008 | 2746.2592 | 2165.3429 | 3384.3810 |
| High SDI | Both | CD | 2008 | 2449.5312 | 1935.4078 | 3010.2709 |
| Low-middle SDI | Male | CD | 2008 | 2888.4826 | 2247.0296 | 3542.7769 |
| Low-middle SDI | Female | CD | 2008 | 3391.4611 | 2663.3476 | 4170.7246 |
| Low-middle SDI | Both | CD | 2008 | 3141.2391 | 2449.3520 | 3860.6662 |
| Low SDI | Male | CD | 2008 | 2449.1376 | 1913.6433 | 2993.2824 |
| Low SDI | Female | CD | 2008 | 3117.0322 | 2443.8254 | 3820.0832 |
| Low SDI | Both | CD | 2008 | 2784.6924 | 2184.3368 | 3408.7097 |
| Middle SDI | Male | CD | 2008 | 2887.0389 | 2243.7966 | 3542.8285 |
| Middle SDI | Female | CD | 2008 | 3681.7493 | 2900.5028 | 4499.0367 |
| Middle SDI | Both | CD | 2008 | 3287.4571 | 2570.7066 | 4018.3701 |
| Low-middle SDI | Male | CD | 2009 | 2886.8516 | 2245.7529 | 3541.1710 |
| Low-middle SDI | Female | CD | 2009 | 3390.2906 | 2662.2837 | 4169.4939 |
| Low-middle SDI | Both | CD | 2009 | 3140.1493 | 2448.5365 | 3858.8569 |
| High-middle SDI | Male | CD | 2009 | 2665.4288 | 2085.8882 | 3253.7424 |
| High-middle SDI | Female | CD | 2009 | 3418.7023 | 2702.5344 | 4171.7825 |
| High-middle SDI | Both | CD | 2009 | 3043.6593 | 2402.0513 | 3707.7734 |
| Low SDI | Male | CD | 2009 | 2450.0828 | 1914.5907 | 2994.4061 |
| Low SDI | Female | CD | 2009 | 3117.0047 | 2443.8105 | 3819.9460 |
| Low SDI | Both | CD | 2009 | 2785.3958 | 2184.8919 | 3409.2889 |
| Middle SDI | Male | CD | 2009 | 2893.7802 | 2249.0611 | 3551.6030 |
| Middle SDI | Female | CD | 2009 | 3680.5849 | 2899.2251 | 4497.7356 |
| Middle SDI | Both | CD | 2009 | 3290.5041 | 2573.0269 | 4022.6065 |
| High SDI | Male | CD | 2009 | 2168.1032 | 1716.3858 | 2644.6347 |
| High SDI | Female | CD | 2009 | 2753.2585 | 2170.7221 | 3393.6853 |
| High SDI | Both | CD | 2009 | 2455.8471 | 1938.9997 | 3019.0190 |
| High-middle SDI | Male | CD | 2010 | 2668.5574 | 2087.7549 | 3257.8223 |
| High-middle SDI | Female | CD | 2010 | 3417.8868 | 2702.3183 | 4172.0215 |
| High-middle SDI | Both | CD | 2010 | 3044.5267 | 2402.6409 | 3709.0397 |
| Low-middle SDI | Male | CD | 2011 | 2883.4353 | 2243.0827 | 3537.8740 |
| Low-middle SDI | Female | CD | 2011 | 3387.8148 | 2659.7272 | 4166.8292 |
| Low-middle SDI | Both | CD | 2011 | 3137.7850 | 2446.5157 | 3854.8822 |
| High SDI | Male | CD | 2010 | 2174.1619 | 1720.9365 | 2652.1953 |
| High SDI | Female | CD | 2010 | 2760.0526 | 2176.3849 | 3402.8979 |
| High SDI | Both | CD | 2010 | 2462.1320 | 1942.5521 | 3027.0935 |
| Low-middle SDI | Male | CD | 2010 | 2885.1750 | 2244.4850 | 3539.5334 |
| Low-middle SDI | Female | CD | 2010 | 3389.0789 | 2661.0920 | 4168.2236 |
| Low-middle SDI | Both | CD | 2010 | 3139.0092 | 2447.6267 | 3856.9320 |
| Low SDI | Male | CD | 2011 | 2452.1958 | 1916.6020 | 2996.9827 |
| Low SDI | Female | CD | 2011 | 3117.0743 | 2443.8888 | 3819.6986 |
| Low SDI | Both | CD | 2011 | 2786.9952 | 2185.6782 | 3410.8123 |
| Low SDI | Male | CD | 2010 | 2451.1277 | 1915.5353 | 2995.5939 |
| Low SDI | Female | CD | 2010 | 3117.0424 | 2443.7475 | 3819.9066 |
| Low SDI | Both | CD | 2010 | 2786.1920 | 2185.2617 | 3410.0283 |
| Middle SDI | Male | CD | 2011 | 2897.0045 | 2250.9224 | 3556.0300 |
| Middle SDI | Female | CD | 2011 | 3678.1085 | 2896.5570 | 4495.0050 |
| Middle SDI | Both | CD | 2011 | 3291.4754 | 2573.4580 | 4023.8919 |
| High-middle SDI | Male | CD | 2011 | 2672.5017 | 2089.8879 | 3264.7370 |
| High-middle SDI | Female | CD | 2011 | 3418.3131 | 2703.0659 | 4173.4756 |
| High-middle SDI | Both | CD | 2011 | 3046.3550 | 2403.3319 | 3712.2984 |
| Middle SDI | Male | CD | 2010 | 2889.8457 | 2245.5111 | 3546.9112 |
| Middle SDI | Female | CD | 2010 | 3679.3319 | 2897.8809 | 4496.2974 |
| Middle SDI | Both | CD | 2010 | 3288.2426 | 2571.1043 | 4019.7916 |
| High SDI | Male | CD | 2011 | 2179.9637 | 1725.5424 | 2659.4996 |
| High SDI | Female | CD | 2011 | 2766.6200 | 2182.0106 | 3411.8694 |
| High SDI | Both | CD | 2011 | 2468.1651 | 1945.9746 | 3034.1699 |
| High-middle SDI | Male | CD | 2012 | 2676.9308 | 2092.7381 | 3271.7891 |
| High-middle SDI | Female | CD | 2012 | 3419.3137 | 2704.3829 | 4174.7551 |
| High-middle SDI | Both | CD | 2012 | 3048.6635 | 2404.2664 | 3715.9010 |
| High SDI | Male | CD | 2012 | 2185.3774 | 1729.9192 | 2666.3699 |
| High SDI | Female | CD | 2012 | 2773.0843 | 2187.5193 | 3418.3148 |
| High SDI | Both | CD | 2012 | 2473.9468 | 1950.2764 | 3040.1569 |
| Low-middle SDI | Male | CD | 2012 | 2881.6123 | 2241.6863 | 3535.8631 |
| Low-middle SDI | Female | CD | 2012 | 3386.5260 | 2658.2592 | 4165.4690 |
| Low-middle SDI | Both | CD | 2012 | 3136.4787 | 2445.2644 | 3852.7713 |
| Low SDI | Male | CD | 2012 | 2453.2254 | 1917.5927 | 2998.3407 |
| Low SDI | Female | CD | 2012 | 3117.0472 | 2443.9503 | 3819.3341 |
| Low SDI | Both | CD | 2012 | 2787.7225 | 2186.1433 | 3411.4340 |
| Middle SDI | Male | CD | 2012 | 2893.2526 | 2247.6889 | 3551.2484 |
| Middle SDI | Female | CD | 2012 | 3676.9931 | 2895.3325 | 4493.8817 |
| Middle SDI | Both | CD | 2012 | 3289.3535 | 2571.6275 | 4020.7840 |
| High SDI | Male | CD | 2013 | 2190.4510 | 1733.8531 | 2672.8473 |
| High SDI | Female | CD | 2013 | 2779.3201 | 2192.8533 | 3424.1811 |
| High SDI | Both | CD | 2013 | 2479.4142 | 1955.4959 | 3045.1788 |
| Low-middle SDI | Male | CD | 2013 | 2879.7340 | 2240.2860 | 3533.2922 |
| Low-middle SDI | Female | CD | 2013 | 3385.2064 | 2656.7691 | 4164.1210 |
| Low-middle SDI | Both | CD | 2013 | 3135.1229 | 2443.9451 | 3850.7025 |
| Low SDI | Male | CD | 2013 | 2454.1903 | 1918.4837 | 2999.4616 |
| Low SDI | Female | CD | 2013 | 3117.0002 | 2443.9823 | 3819.1055 |
| Low SDI | Both | CD | 2013 | 2788.4035 | 2186.6384 | 3412.1364 |
| High-middle SDI | Male | CD | 2014 | 2685.9522 | 2100.7712 | 3284.8817 |
| High-middle SDI | Female | CD | 2014 | 3422.7193 | 2708.2138 | 4175.3794 |
| High-middle SDI | Both | CD | 2014 | 3054.2196 | 2407.8245 | 3724.8870 |
| Middle SDI | Male | CD | 2013 | 2894.8218 | 2248.7325 | 3552.9179 |
| Middle SDI | Female | CD | 2013 | 3675.9507 | 2894.1531 | 4493.0909 |
| Middle SDI | Both | CD | 2013 | 3289.8996 | 2571.8680 | 4020.8715 |
| High SDI | Male | CD | 2014 | 2194.8845 | 1736.9743 | 2678.7080 |
| High SDI | Female | CD | 2014 | 2785.1956 | 2197.8690 | 3429.7761 |
| High SDI | Both | CD | 2014 | 2484.3951 | 1960.4693 | 3049.3894 |
| High-middle SDI | Male | CD | 2013 | 2681.3974 | 2097.3239 | 3278.5758 |
| High-middle SDI | Female | CD | 2013 | 3420.8720 | 2706.2389 | 4175.8564 |
| High-middle SDI | Both | CD | 2013 | 3051.3382 | 2405.8645 | 3720.1330 |
| Low-middle SDI | Male | CD | 2014 | 2877.8282 | 2238.9086 | 3530.6924 |
| Low-middle SDI | Female | CD | 2014 | 3383.8833 | 2655.2614 | 4162.8082 |
| Low-middle SDI | Both | CD | 2014 | 3133.7487 | 2442.5502 | 3848.6533 |
| Low SDI | Male | CD | 2014 | 2455.0801 | 1919.2162 | 3000.4217 |
| Low SDI | Female | CD | 2014 | 3116.8972 | 2443.9133 | 3818.6757 |
| Low SDI | Both | CD | 2014 | 2789.0054 | 2187.1009 | 3412.7304 |
| High SDI | Male | CD | 2015 | 2199.0789 | 1740.5158 | 2683.6964 |
| High SDI | Female | CD | 2015 | 2790.5961 | 2202.6378 | 3435.4315 |
| High SDI | Both | CD | 2015 | 2489.0237 | 1965.0678 | 3053.0289 |
| Middle SDI | Male | CD | 2014 | 2896.3396 | 2249.7243 | 3553.3488 |
| Middle SDI | Female | CD | 2014 | 3674.8893 | 2892.9455 | 4492.5627 |
| Middle SDI | Both | CD | 2014 | 3290.3581 | 2572.0047 | 4020.7606 |
| Middle SDI | Male | CD | 2015 | 2898.1152 | 2250.9930 | 3555.6980 |
| Middle SDI | Female | CD | 2015 | 3673.7147 | 2891.6274 | 4491.6640 |
| Middle SDI | Both | CD | 2015 | 3290.8827 | 2572.1154 | 4020.8918 |
| Low SDI | Male | CD | 2015 | 2455.8775 | 1919.8003 | 3001.3019 |
| Low SDI | Female | CD | 2015 | 3116.7658 | 2443.6724 | 3817.8706 |
| Low SDI | Both | CD | 2015 | 2789.5180 | 2187.5187 | 3413.0636 |
| High-middle SDI | Male | CD | 2016 | 2694.4197 | 2106.2918 | 3294.3407 |
| High-middle SDI | Female | CD | 2016 | 3426.5725 | 2714.1263 | 4177.6898 |
| High-middle SDI | Both | CD | 2016 | 3059.6772 | 2411.3458 | 3733.8582 |
| Low-middle SDI | Male | CD | 2015 | 2875.9581 | 2237.5792 | 3528.0534 |
| Low-middle SDI | Female | CD | 2015 | 3382.5628 | 2653.8008 | 4161.1738 |
| Low-middle SDI | Both | CD | 2015 | 3132.3545 | 2441.2188 | 3846.6347 |
| High-middle SDI | Male | CD | 2015 | 2690.1488 | 2103.2306 | 3289.7648 |
| High-middle SDI | Female | CD | 2015 | 3424.3255 | 2710.3276 | 4175.4306 |
| High-middle SDI | Both | CD | 2015 | 3056.7867 | 2409.2655 | 3728.9429 |
| High SDI | Male | CD | 2016 | 2202.9727 | 1744.1267 | 2686.3983 |
| High SDI | Female | CD | 2016 | 2795.7567 | 2205.8747 | 3441.1061 |
| High SDI | Both | CD | 2016 | 2493.3948 | 1969.2233 | 3056.2947 |
| Low-middle SDI | Male | CD | 2016 | 2874.1726 | 2236.3602 | 3525.3959 |
| Low-middle SDI | Female | CD | 2016 | 3381.3173 | 2652.4395 | 4159.2992 |
| Low-middle SDI | Both | CD | 2016 | 3131.0099 | 2439.9933 | 3844.7027 |
| Low SDI | Male | CD | 2016 | 2456.5770 | 1920.3075 | 3002.0628 |
| Low SDI | Female | CD | 2016 | 3116.6562 | 2443.5418 | 3817.5160 |
| Low SDI | Both | CD | 2016 | 2789.9772 | 2187.9430 | 3413.4045 |
| Middle SDI | Male | CD | 2016 | 2899.8255 | 2252.0476 | 3556.6827 |
| Middle SDI | Female | CD | 2016 | 3672.4432 | 2890.3149 | 4490.3218 |
| Middle SDI | Both | CD | 2016 | 3291.2943 | 2572.0321 | 4021.0268 |
| High SDI | Male | CD | 2017 | 2206.8781 | 1747.8534 | 2690.8843 |
| High SDI | Female | CD | 2017 | 2800.8206 | 2208.2516 | 3445.2282 |
| High SDI | Both | CD | 2017 | 2497.7438 | 1972.9618 | 3058.9874 |
| Low-middle SDI | Male | CD | 2017 | 2872.4705 | 2235.2066 | 3522.7400 |
| Low-middle SDI | Female | CD | 2017 | 3380.1414 | 2651.5433 | 4157.5720 |
| Low-middle SDI | Both | CD | 2017 | 3129.7327 | 2438.8823 | 3842.9344 |
| Low SDI | Male | CD | 2017 | 2457.1496 | 1920.7023 | 3002.7276 |
| Low SDI | Female | CD | 2017 | 3116.4241 | 2443.3447 | 3816.6594 |
| Low SDI | Both | CD | 2017 | 2790.2741 | 2188.1913 | 3413.7256 |
| High-middle SDI | Male | CD | 2017 | 2698.4708 | 2110.8128 | 3297.8301 |
| High-middle SDI | Female | CD | 2017 | 3428.8017 | 2716.7661 | 4180.0157 |
| High-middle SDI | Both | CD | 2017 | 3062.4569 | 2414.6906 | 3737.5334 |
| Middle SDI | Male | CD | 2017 | 2906.8384 | 2257.0593 | 3564.2521 |
| Middle SDI | Female | CD | 2017 | 3671.1252 | 2889.2712 | 4488.6662 |
| Middle SDI | Both | CD | 2017 | 3294.2586 | 2573.8830 | 4024.4717 |
| High-middle SDI | Male | CD | 2018 | 2702.1639 | 2113.1864 | 3300.5241 |
| High-middle SDI | Female | CD | 2018 | 3431.1964 | 2719.0589 | 4180.8015 |
| High-middle SDI | Both | CD | 2018 | 3065.1983 | 2416.2168 | 3739.8446 |
| High SDI | Male | CD | 2018 | 2210.5098 | 1751.3661 | 2696.9630 |
| High SDI | Female | CD | 2018 | 2805.4681 | 2210.0742 | 3448.6156 |
| High SDI | Both | CD | 2018 | 2501.7483 | 1976.5008 | 3061.2360 |
| Low-middle SDI | Male | CD | 2018 | 2870.7902 | 2234.0549 | 3520.0981 |
| Low-middle SDI | Female | CD | 2018 | 3378.9368 | 2650.8704 | 4155.9774 |
| Low-middle SDI | Both | CD | 2018 | 3128.4382 | 2437.8098 | 3841.2324 |
| Low SDI | Male | CD | 2018 | 2457.5797 | 1920.9682 | 3003.1437 |
| Low SDI | Female | CD | 2018 | 3116.1174 | 2443.0209 | 3815.7038 |
| Low SDI | Both | CD | 2018 | 2790.4514 | 2188.2251 | 3413.9246 |
| Middle SDI | Male | CD | 2018 | 2908.2113 | 2257.3954 | 3564.8615 |
| Middle SDI | Female | CD | 2018 | 3669.8512 | 2888.2816 | 4486.9856 |
| Middle SDI | Both | CD | 2018 | 3294.4440 | 2573.5656 | 4024.3481 |
| High SDI | Male | CD | 2019 | 2213.7913 | 1754.3458 | 2702.3922 |
| High SDI | Female | CD | 2019 | 2809.8276 | 2212.4774 | 3452.3443 |
| High SDI | Both | CD | 2019 | 2505.4542 | 1979.8450 | 3063.7478 |
| Low-middle SDI | Male | CD | 2019 | 2869.1086 | 2232.8756 | 3517.4702 |
| Low-middle SDI | Female | CD | 2019 | 3377.6994 | 2650.1604 | 4154.3920 |
| Low-middle SDI | Both | CD | 2019 | 3127.1088 | 2436.7674 | 3839.4989 |
| Low SDI | Male | CD | 2019 | 2457.8661 | 1921.1145 | 3003.2924 |
| Low SDI | Female | CD | 2019 | 3115.7809 | 2442.6691 | 3814.8103 |
| Low SDI | Both | CD | 2019 | 2790.5367 | 2188.2161 | 3414.0224 |
| Middle SDI | Male | CD | 2019 | 2909.5856 | 2258.5382 | 3566.4022 |
| Middle SDI | Female | CD | 2019 | 3668.5758 | 2887.2509 | 4485.3101 |
| Middle SDI | Both | CD | 2019 | 3294.5941 | 2573.1487 | 4024.3112 |
| High-middle SDI | Male | CD | 2019 | 2705.5831 | 2114.9019 | 3302.5082 |
| High-middle SDI | Female | CD | 2019 | 3433.5658 | 2720.4812 | 4182.1921 |
| High-middle SDI | Both | CD | 2019 | 3067.7851 | 2417.5886 | 3741.9077 |
|  |  |  |  |  |  |  |

**Table16 DALYs of CD stratified by sex in 1990~2019, Page 73-75**

| **location_name** | **sex_name** | **cause_name** | **year** | **Age-standardised DALYs rate (per 100 000 person-years)** | **95%CI(lower)** | **95%CI(upper)** |
| --- | --- | --- | --- | --- | --- | --- |
| Global | Male | CD | 1992 | 25.4502 | 15.9170 | 37.8878 |
| Global | Female | CD | 1992 | 30.1889 | 19.1312 | 44.6273 |
| Global | Both | CD | 1992 | 27.8096 | 17.4737 | 41.3084 |
| Global | Male | CD | 1993 | 25.3753 | 15.9301 | 37.7387 |
| Global | Female | CD | 1993 | 30.0618 | 19.0269 | 44.5730 |
| Global | Both | CD | 1993 | 27.7081 | 17.3996 | 41.1430 |
| Global | Male | CD | 1991 | 25.5575 | 15.9762 | 38.0919 |
| Global | Female | CD | 1991 | 30.3381 | 19.1635 | 44.8543 |
| Global | Both | CD | 1991 | 27.9388 | 17.5429 | 41.5677 |
| Global | Male | CD | 1990 | 25.6677 | 16.0898 | 38.1026 |
| Global | Female | CD | 1990 | 30.5172 | 19.2735 | 44.9476 |
| Global | Both | CD | 1990 | 28.0846 | 17.6198 | 41.6829 |
| Global | Male | CD | 1994 | 25.3309 | 15.8609 | 37.7734 |
| Global | Female | CD | 1994 | 29.9864 | 19.0062 | 44.2995 |
| Global | Both | CD | 1994 | 27.6482 | 17.3438 | 41.0534 |
| Global | Male | CD | 1995 | 25.3354 | 15.7951 | 37.7613 |
| Global | Female | CD | 1995 | 29.9699 | 18.9796 | 44.3983 |
| Global | Both | CD | 1995 | 27.6426 | 17.3748 | 41.1272 |
| Global | Male | CD | 1998 | 25.4118 | 15.8583 | 37.8527 |
| Global | Female | CD | 1998 | 30.0335 | 19.0392 | 44.4860 |
| Global | Both | CD | 1998 | 27.7153 | 17.4204 | 41.2779 |
| Global | Male | CD | 1999 | 25.4361 | 15.9344 | 37.8915 |
| Global | Female | CD | 1999 | 30.0531 | 19.0494 | 44.6144 |
| Global | Both | CD | 1999 | 27.7383 | 17.4162 | 41.2555 |
| Global | Male | CD | 2002 | 25.4955 | 15.9419 | 38.0248 |
| Global | Female | CD | 2002 | 30.1009 | 19.0771 | 44.7510 |
| Global | Both | CD | 2002 | 27.7953 | 17.5067 | 41.3179 |
| Global | Male | CD | 1997 | 25.3894 | 15.8128 | 37.7754 |
| Global | Female | CD | 1997 | 30.0095 | 18.9940 | 44.4940 |
| Global | Both | CD | 1997 | 27.6911 | 17.3915 | 41.1055 |
| Global | Male | CD | 1996 | 25.3631 | 15.8403 | 37.7720 |
| Global | Female | CD | 1996 | 29.9905 | 18.9756 | 44.3377 |
| Global | Both | CD | 1996 | 27.6674 | 17.3884 | 41.1150 |
| Global | Male | CD | 2000 | 25.4557 | 15.8697 | 38.0073 |
| Global | Female | CD | 2000 | 30.0711 | 19.0450 | 44.7217 |
| Global | Both | CD | 2000 | 27.7584 | 17.4740 | 41.2582 |
| Global | Male | CD | 2006 | 25.5720 | 15.9596 | 38.1537 |
| Global | Female | CD | 2006 | 30.1538 | 19.1220 | 44.6082 |
| Global | Both | CD | 2006 | 27.8633 | 17.5238 | 41.4692 |
| Global | Male | CD | 2001 | 25.4738 | 15.8702 | 37.9959 |
| Global | Female | CD | 2001 | 30.0894 | 19.1305 | 44.5655 |
| Global | Both | CD | 2001 | 27.7776 | 17.4895 | 41.3028 |
| Global | Male | CD | 2003 | 25.5177 | 15.9260 | 38.0641 |
| Global | Female | CD | 2003 | 30.1169 | 19.0767 | 44.6379 |
| Global | Both | CD | 2003 | 27.8152 | 17.4947 | 41.3427 |
| Global | Male | CD | 2007 | 25.5974 | 15.9966 | 38.1901 |
| Global | Female | CD | 2007 | 30.1649 | 19.0416 | 44.5880 |
| Global | Both | CD | 2007 | 27.8820 | 17.4972 | 41.3758 |
| Global | Male | CD | 2005 | 25.5533 | 15.9074 | 38.0812 |
| Global | Female | CD | 2005 | 30.1343 | 19.0191 | 44.6096 |
| Global | Both | CD | 2005 | 27.8436 | 17.4548 | 41.4132 |
| Global | Male | CD | 2010 | 25.6543 | 16.0159 | 38.3404 |
| Global | Female | CD | 2010 | 30.2078 | 19.1447 | 44.6472 |
| Global | Both | CD | 2010 | 27.9341 | 17.5944 | 41.4840 |
| Global | Male | CD | 2004 | 25.5374 | 15.9616 | 38.0061 |
| Global | Female | CD | 2004 | 30.1254 | 19.0867 | 44.6052 |
| Global | Both | CD | 2004 | 27.8302 | 17.4795 | 41.3119 |
| Global | Male | CD | 2014 | 25.7367 | 16.0613 | 38.3320 |
| Global | Female | CD | 2014 | 30.2507 | 19.1749 | 44.8997 |
| Global | Both | CD | 2014 | 27.9993 | 17.5858 | 41.6784 |
| Global | Male | CD | 2009 | 25.6389 | 15.9821 | 38.3055 |
| Global | Female | CD | 2009 | 30.1978 | 19.1873 | 44.7271 |
| Global | Both | CD | 2009 | 27.9205 | 17.5627 | 41.5213 |
| Global | Male | CD | 2008 | 25.6149 | 16.0483 | 38.2025 |
| Global | Female | CD | 2008 | 30.1855 | 19.1582 | 44.6016 |
| Global | Both | CD | 2008 | 27.9016 | 17.5650 | 41.4047 |
| Global | Male | CD | 2011 | 25.6731 | 16.0191 | 38.3394 |
| Global | Female | CD | 2011 | 30.2165 | 19.1149 | 44.8034 |
| Global | Both | CD | 2011 | 27.9484 | 17.5530 | 41.5110 |
| Global | Male | CD | 2012 | 25.6966 | 16.0309 | 38.3723 |
| Global | Female | CD | 2012 | 30.2306 | 19.1245 | 44.8368 |
| Global | Both | CD | 2012 | 27.9678 | 17.5764 | 41.6363 |
| Global | Male | CD | 2018 | 25.7972 | 16.0348 | 38.5930 |
| Global | Female | CD | 2018 | 30.2771 | 19.1276 | 44.8854 |
| Global | Both | CD | 2018 | 28.0444 | 17.5843 | 41.7567 |
| Global | Male | CD | 2013 | 25.7127 | 16.0287 | 38.4379 |
| Global | Female | CD | 2013 | 30.2433 | 19.1502 | 44.8388 |
| Global | Both | CD | 2013 | 27.9830 | 17.6161 | 41.6532 |
| Global | Male | CD | 2015 | 25.7516 | 16.0904 | 38.4233 |
| Global | Female | CD | 2015 | 30.2580 | 19.1080 | 44.8149 |
| Global | Both | CD | 2015 | 28.0108 | 17.6032 | 41.5691 |
| Global | Male | CD | 2016 | 25.7655 | 16.0361 | 38.5153 |
| Global | Female | CD | 2016 | 30.2667 | 19.1220 | 44.8037 |
| Global | Both | CD | 2016 | 28.0225 | 17.5868 | 41.7422 |
| Global | Male | CD | 2017 | 25.7862 | 16.0471 | 38.5758 |
| Global | Female | CD | 2017 | 30.2734 | 19.0902 | 44.7566 |
| Global | Both | CD | 2017 | 28.0366 | 17.5983 | 41.6581 |
| Global | Male | CD | 2019 | 25.8156 | 16.0938 | 38.5597 |
| Global | Female | CD | 2019 | 30.2843 | 19.1718 | 44.8530 |
| Global | Both | CD | 2019 | 28.0575 | 17.6212 | 41.7765 |
|  |  |  |  |  |  |  |

**Table17 DALYs of CD stratified by SDI in 1990~2019, Page 76-86**

| **location_name** | **sex_name** | **cause_name** | **year** | **Age-standardised DALYs rate (per 100 000 person-years)** | **95%CI(lower)** | **95%CI(upper)** |
| --- | --- | --- | --- | --- | --- | --- |
| High-middle SDI | Male | CD | 1990 | 23.8807 | 14.9475 | 35.4827 |
| High-middle SDI | Female | CD | 1990 | 31.0776 | 19.6504 | 45.9572 |
| High-middle SDI | Both | CD | 1990 | 27.5114 | 17.3189 | 40.9649 |
| High SDI | Male | CD | 1990 | 23.5734 | 15.1080 | 34.9922 |
| High SDI | Female | CD | 1990 | 29.5340 | 19.1000 | 43.8251 |
| High SDI | Both | CD | 1990 | 26.5322 | 17.0857 | 39.4403 |
| Middle SDI | Male | CD | 1990 | 29.0921 | 18.1759 | 43.7703 |
| Middle SDI | Female | CD | 1990 | 33.3361 | 21.1210 | 49.0917 |
| Middle SDI | Both | CD | 1990 | 31.2113 | 19.6627 | 46.4362 |
| Low-middle SDI | Male | CD | 1990 | 26.5946 | 16.6811 | 39.5501 |
| Low-middle SDI | Female | CD | 1990 | 30.2637 | 19.0539 | 44.5378 |
| Low-middle SDI | Both | CD | 1990 | 28.4041 | 17.8710 | 42.1621 |
| Low SDI | Male | CD | 1990 | 24.5654 | 15.3519 | 36.6304 |
| Low SDI | Female | CD | 1990 | 27.7263 | 17.4057 | 41.3211 |
| Low SDI | Both | CD | 1990 | 26.1379 | 16.4102 | 39.0012 |
| Low-middle SDI | Male | CD | 1991 | 26.5989 | 16.6571 | 39.5783 |
| Low-middle SDI | Female | CD | 1991 | 30.2708 | 19.1839 | 44.6086 |
| Low-middle SDI | Both | CD | 1991 | 28.4104 | 17.8940 | 42.0747 |
| High SDI | Male | CD | 1991 | 22.8272 | 14.7226 | 33.9050 |
| High SDI | Female | CD | 1991 | 28.5647 | 18.4096 | 42.3260 |
| High SDI | Both | CD | 1991 | 25.6729 | 16.5361 | 38.0612 |
| Low SDI | Male | CD | 1991 | 24.5565 | 15.4028 | 36.4979 |
| Low SDI | Female | CD | 1991 | 27.7170 | 17.3793 | 41.3188 |
| Low SDI | Both | CD | 1991 | 26.1284 | 16.4285 | 38.9430 |
| High-middle SDI | Male | CD | 1992 | 23.9634 | 15.0537 | 35.7476 |
| High-middle SDI | Female | CD | 1992 | 31.0535 | 19.6906 | 46.2142 |
| High-middle SDI | Both | CD | 1992 | 27.5344 | 17.3288 | 41.1826 |
| High-middle SDI | Male | CD | 1991 | 23.9272 | 14.9581 | 35.7643 |
| High-middle SDI | Female | CD | 1991 | 31.0569 | 19.6767 | 45.9512 |
| High-middle SDI | Both | CD | 1991 | 27.5205 | 17.3320 | 41.2377 |
| Middle SDI | Male | CD | 1991 | 29.1031 | 18.0868 | 43.6644 |
| Middle SDI | Female | CD | 1991 | 33.3318 | 20.9788 | 49.4222 |
| Middle SDI | Both | CD | 1991 | 31.2141 | 19.6660 | 46.5151 |
| High SDI | Male | CD | 1992 | 22.1387 | 14.1649 | 32.9454 |
| High SDI | Female | CD | 1992 | 27.6834 | 17.8083 | 41.0330 |
| High SDI | Both | CD | 1992 | 24.8866 | 15.9998 | 36.8515 |
| Low-middle SDI | Male | CD | 1992 | 26.5971 | 16.6480 | 39.5902 |
| Low-middle SDI | Female | CD | 1992 | 30.2795 | 18.9674 | 44.4432 |
| Low-middle SDI | Both | CD | 1992 | 28.4146 | 17.8024 | 42.0408 |
| High-middle SDI | Male | CD | 1993 | 24.0090 | 15.1655 | 35.9116 |
| High-middle SDI | Female | CD | 1993 | 31.0364 | 19.8074 | 45.9440 |
| High-middle SDI | Both | CD | 1993 | 27.5475 | 17.4718 | 41.1587 |
| Low SDI | Male | CD | 1992 | 24.5536 | 15.4021 | 36.3419 |
| Low SDI | Female | CD | 1992 | 27.7192 | 17.2523 | 41.2321 |
| Low SDI | Both | CD | 1992 | 26.1275 | 16.3707 | 38.8950 |
| High SDI | Male | CD | 1993 | 21.5866 | 13.8034 | 32.0424 |
| High SDI | Female | CD | 1993 | 26.9703 | 17.3287 | 40.0126 |
| High SDI | Both | CD | 1993 | 24.2532 | 15.5524 | 36.1004 |
| Low-middle SDI | Male | CD | 1993 | 26.6103 | 16.6471 | 39.6494 |
| Low-middle SDI | Female | CD | 1993 | 30.2773 | 19.0933 | 44.5207 |
| Low-middle SDI | Both | CD | 1993 | 28.4210 | 17.8346 | 42.1588 |
| Low SDI | Male | CD | 1993 | 24.5521 | 15.3055 | 36.2446 |
| Low SDI | Female | CD | 1993 | 27.7116 | 17.3112 | 41.0921 |
| Low SDI | Both | CD | 1993 | 26.1225 | 16.3227 | 38.8862 |
| Middle SDI | Male | CD | 1992 | 29.0986 | 18.1105 | 43.7836 |
| Middle SDI | Female | CD | 1992 | 33.3486 | 21.0551 | 49.1970 |
| Middle SDI | Both | CD | 1992 | 31.2195 | 19.7221 | 46.4790 |
| Middle SDI | Male | CD | 1994 | 29.0865 | 18.0736 | 43.6838 |
| Middle SDI | Female | CD | 1994 | 33.3517 | 20.9338 | 49.5861 |
| Middle SDI | Both | CD | 1994 | 31.2143 | 19.6628 | 46.4330 |
| Middle SDI | Male | CD | 1993 | 29.0976 | 18.1100 | 43.8097 |
| Middle SDI | Female | CD | 1993 | 33.3472 | 21.1186 | 49.7531 |
| Middle SDI | Both | CD | 1993 | 31.2179 | 19.6346 | 46.4490 |
| High-middle SDI | Male | CD | 1994 | 24.0647 | 15.0839 | 36.1748 |
| High-middle SDI | Female | CD | 1994 | 31.0407 | 19.7463 | 46.0092 |
| High-middle SDI | Both | CD | 1994 | 27.5764 | 17.4166 | 41.1830 |
| High SDI | Male | CD | 1994 | 21.2294 | 13.7534 | 31.4870 |
| High SDI | Female | CD | 1994 | 26.4951 | 17.0176 | 39.1838 |
| High SDI | Both | CD | 1994 | 23.8360 | 15.3608 | 35.3265 |
| Low-middle SDI | Male | CD | 1994 | 26.6049 | 16.6670 | 39.4745 |
| Low-middle SDI | Female | CD | 1994 | 30.2730 | 18.9950 | 44.6314 |
| Low-middle SDI | Both | CD | 1994 | 28.4177 | 17.8201 | 42.0877 |
| Low SDI | Male | CD | 1994 | 24.5577 | 15.3094 | 36.5305 |
| Low SDI | Female | CD | 1994 | 27.7257 | 17.3112 | 41.3314 |
| Low SDI | Both | CD | 1994 | 26.1327 | 16.3862 | 38.9638 |
| Middle SDI | Male | CD | 1995 | 29.0966 | 17.9960 | 43.8421 |
| Middle SDI | Female | CD | 1995 | 33.3425 | 20.9061 | 49.2826 |
| Middle SDI | Both | CD | 1995 | 31.2152 | 19.6770 | 46.5335 |
| High-middle SDI | Male | CD | 1995 | 24.1036 | 15.0838 | 36.0261 |
| High-middle SDI | Female | CD | 1995 | 31.0362 | 19.7926 | 45.9971 |
| High-middle SDI | Both | CD | 1995 | 27.5940 | 17.4308 | 41.1136 |
| High SDI | Male | CD | 1995 | 21.1257 | 13.6449 | 31.2418 |
| High SDI | Female | CD | 1995 | 26.3600 | 16.9419 | 39.0346 |
| High SDI | Both | CD | 1995 | 23.7153 | 15.2687 | 35.3722 |
| Low-middle SDI | Male | CD | 1995 | 26.6143 | 16.6540 | 39.6741 |
| Low-middle SDI | Female | CD | 1995 | 30.2841 | 19.0754 | 44.7005 |
| Low-middle SDI | Both | CD | 1995 | 28.4296 | 17.9047 | 42.2829 |
| Low SDI | Male | CD | 1995 | 24.5474 | 15.2775 | 36.3777 |
| Low SDI | Female | CD | 1995 | 27.7100 | 17.3807 | 40.9778 |
| Low SDI | Both | CD | 1995 | 26.1209 | 16.3499 | 38.8611 |
| Middle SDI | Male | CD | 1996 | 29.0877 | 18.1893 | 43.9237 |
| Middle SDI | Female | CD | 1996 | 33.3375 | 20.9746 | 49.3389 |
| Middle SDI | Both | CD | 1996 | 31.2092 | 19.6641 | 46.3837 |
| Low-middle SDI | Male | CD | 1996 | 26.6102 | 16.8480 | 39.7437 |
| Low-middle SDI | Female | CD | 1996 | 30.2798 | 18.8940 | 44.7872 |
| Low-middle SDI | Both | CD | 1996 | 28.4273 | 17.8164 | 42.3541 |
| High-middle SDI | Male | CD | 1996 | 24.1576 | 15.1096 | 36.0269 |
| High-middle SDI | Female | CD | 1996 | 31.0453 | 19.8049 | 46.0414 |
| High-middle SDI | Both | CD | 1996 | 27.6257 | 17.5343 | 41.2203 |
| Low SDI | Male | CD | 1996 | 24.5389 | 15.3794 | 36.3772 |
| Low SDI | Female | CD | 1996 | 27.7105 | 17.3804 | 41.1885 |
| Low SDI | Both | CD | 1996 | 26.1179 | 16.3435 | 38.8517 |
| High SDI | Male | CD | 1996 | 21.1788 | 13.6776 | 31.3087 |
| High SDI | Female | CD | 1996 | 26.4207 | 16.9411 | 39.2989 |
| High SDI | Both | CD | 1996 | 23.7710 | 15.3084 | 35.2677 |
| High-middle SDI | Male | CD | 1997 | 24.1997 | 15.1626 | 36.4259 |
| High-middle SDI | Female | CD | 1997 | 31.0496 | 19.8080 | 45.9526 |
| High-middle SDI | Both | CD | 1997 | 27.6494 | 17.4873 | 41.2106 |
| High SDI | Male | CD | 1997 | 21.2126 | 13.6397 | 31.4260 |
| High SDI | Female | CD | 1997 | 26.4725 | 16.9542 | 39.2495 |
| High SDI | Both | CD | 1997 | 23.8128 | 15.3289 | 35.5588 |
| Low-middle SDI | Male | CD | 1997 | 26.6164 | 16.5818 | 39.6431 |
| Low-middle SDI | Female | CD | 1997 | 30.2977 | 19.0065 | 44.5535 |
| Low-middle SDI | Both | CD | 1997 | 28.4414 | 17.8389 | 42.3032 |
| Low SDI | Male | CD | 1997 | 24.5431 | 15.3411 | 36.6046 |
| Low SDI | Female | CD | 1997 | 27.7037 | 17.3700 | 41.2305 |
| Low SDI | Both | CD | 1997 | 26.1176 | 16.3812 | 38.9001 |
| Middle SDI | Male | CD | 1997 | 29.0903 | 18.0920 | 43.5744 |
| Middle SDI | Female | CD | 1997 | 33.3281 | 20.9731 | 49.3659 |
| Middle SDI | Both | CD | 1997 | 31.2070 | 19.5868 | 46.4355 |
| High-middle SDI | Male | CD | 1998 | 24.2324 | 15.2026 | 36.3779 |
| High-middle SDI | Female | CD | 1998 | 31.0603 | 19.7910 | 46.0546 |
| High-middle SDI | Both | CD | 1998 | 27.6712 | 17.4429 | 41.4093 |
| High SDI | Male | CD | 1998 | 21.2716 | 13.6268 | 31.8211 |
| High SDI | Female | CD | 1998 | 26.5326 | 17.0714 | 39.4621 |
| High SDI | Both | CD | 1998 | 23.8713 | 15.3414 | 35.7295 |
| Low-middle SDI | Male | CD | 1998 | 26.6184 | 16.6290 | 39.6618 |
| Low-middle SDI | Female | CD | 1998 | 30.3045 | 18.9781 | 44.7119 |
| Low-middle SDI | Both | CD | 1998 | 28.4481 | 17.9084 | 42.3693 |
| Low SDI | Male | CD | 1998 | 24.5279 | 15.4667 | 36.4971 |
| Low SDI | Female | CD | 1998 | 27.7194 | 17.3765 | 41.2101 |
| Low SDI | Both | CD | 1998 | 26.1190 | 16.3447 | 39.0028 |
| Middle SDI | Male | CD | 1998 | 29.0804 | 18.0760 | 43.7019 |
| Middle SDI | Female | CD | 1998 | 33.3277 | 20.9016 | 49.3574 |
| Middle SDI | Both | CD | 1998 | 31.2033 | 19.6132 | 46.5102 |
| Middle SDI | Male | CD | 1999 | 29.0741 | 18.0747 | 44.0433 |
| Middle SDI | Female | CD | 1999 | 33.3207 | 21.0402 | 49.1195 |
| Middle SDI | Both | CD | 1999 | 31.1982 | 19.5949 | 46.3795 |
| High-middle SDI | Male | CD | 1999 | 24.2642 | 15.1620 | 36.2815 |
| High-middle SDI | Female | CD | 1999 | 31.0615 | 19.7945 | 46.1882 |
| High-middle SDI | Both | CD | 1999 | 27.6873 | 17.5016 | 41.2945 |
| High SDI | Male | CD | 1999 | 21.3200 | 13.7202 | 31.7340 |
| High SDI | Female | CD | 1999 | 26.5909 | 17.0809 | 39.5217 |
| High SDI | Both | CD | 1999 | 23.9241 | 15.4519 | 35.7427 |
| High-middle SDI | Male | CD | 2000 | 24.2988 | 15.2364 | 36.3628 |
| High-middle SDI | Female | CD | 2000 | 31.0558 | 19.7763 | 46.2541 |
| High-middle SDI | Both | CD | 2000 | 27.7019 | 17.5055 | 41.4381 |
| Low-middle SDI | Male | CD | 1999 | 26.6280 | 16.6680 | 39.7145 |
| Low-middle SDI | Female | CD | 1999 | 30.3158 | 19.0156 | 44.7387 |
| Low-middle SDI | Both | CD | 1999 | 28.4607 | 17.8262 | 42.2390 |
| Low SDI | Male | CD | 1999 | 24.5247 | 15.4037 | 36.2751 |
| Low SDI | Female | CD | 1999 | 27.7176 | 17.4112 | 41.4250 |
| Low SDI | Both | CD | 1999 | 26.1176 | 16.3879 | 38.9608 |
| High SDI | Male | CD | 2000 | 21.3659 | 13.7715 | 31.8372 |
| High SDI | Female | CD | 2000 | 26.6401 | 17.0047 | 39.6831 |
| High SDI | Both | CD | 2000 | 23.9716 | 15.3954 | 35.7996 |
| Low-middle SDI | Male | CD | 2000 | 26.6200 | 16.6021 | 39.9064 |
| Low-middle SDI | Female | CD | 2000 | 30.3180 | 19.0693 | 44.8419 |
| Low-middle SDI | Both | CD | 2000 | 28.4598 | 17.8653 | 42.3435 |
| Low SDI | Male | CD | 2000 | 24.5333 | 15.3811 | 36.6029 |
| Low SDI | Female | CD | 2000 | 27.7305 | 17.3976 | 41.2870 |
| Low SDI | Both | CD | 2000 | 26.1299 | 16.4248 | 39.0000 |
| Middle SDI | Male | CD | 2000 | 29.0653 | 18.0822 | 43.7107 |
| Middle SDI | Female | CD | 2000 | 33.3149 | 20.9139 | 49.4757 |
| Middle SDI | Both | CD | 2000 | 31.1925 | 19.6148 | 46.5312 |
| Low-middle SDI | Male | CD | 2001 | 26.6127 | 16.6288 | 39.9536 |
| Low-middle SDI | Female | CD | 2001 | 30.3170 | 19.0015 | 44.6839 |
| Low-middle SDI | Both | CD | 2001 | 28.4579 | 17.7866 | 42.5378 |
| Low SDI | Male | CD | 2001 | 24.5291 | 15.2756 | 36.6305 |
| Low SDI | Female | CD | 2001 | 27.7132 | 17.4436 | 41.1389 |
| Low SDI | Both | CD | 2001 | 26.1203 | 16.3862 | 39.0382 |
| High-middle SDI | Male | CD | 2001 | 24.3282 | 15.2194 | 36.4612 |
| High-middle SDI | Female | CD | 2001 | 31.0591 | 19.7395 | 46.2523 |
| High-middle SDI | Both | CD | 2001 | 27.7175 | 17.5000 | 41.5292 |
| High SDI | Male | CD | 2001 | 21.4009 | 13.7557 | 31.8111 |
| High SDI | Female | CD | 2001 | 26.7067 | 17.1300 | 39.7366 |
| High SDI | Both | CD | 2001 | 24.0216 | 15.4374 | 35.7668 |
| Middle SDI | Male | CD | 2001 | 29.0577 | 18.0846 | 43.8124 |
| Middle SDI | Female | CD | 2001 | 33.3122 | 20.8916 | 49.3173 |
| Middle SDI | Both | CD | 2001 | 31.1888 | 19.6418 | 46.3377 |
| High-middle SDI | Male | CD | 2002 | 24.3612 | 15.2414 | 36.3581 |
| High-middle SDI | Female | CD | 2002 | 31.0330 | 19.8008 | 46.0877 |
| High-middle SDI | Both | CD | 2002 | 27.7200 | 17.4293 | 41.3558 |
| High SDI | Male | CD | 2002 | 21.4492 | 13.8059 | 31.9925 |
| High SDI | Female | CD | 2002 | 26.7645 | 17.1622 | 39.7545 |
| High SDI | Both | CD | 2002 | 24.0746 | 15.4624 | 36.0206 |
| Low-middle SDI | Male | CD | 2002 | 26.6214 | 16.6261 | 39.9197 |
| Low-middle SDI | Female | CD | 2002 | 30.3299 | 19.1119 | 44.7696 |
| Low-middle SDI | Both | CD | 2002 | 28.4709 | 17.8463 | 42.3650 |
| Low SDI | Male | CD | 2002 | 24.5138 | 15.2917 | 36.6902 |
| Low SDI | Female | CD | 2002 | 27.7189 | 17.4701 | 41.4168 |
| Low SDI | Both | CD | 2002 | 26.1164 | 16.3793 | 39.0132 |
| Middle SDI | Male | CD | 2003 | 29.0463 | 17.9745 | 43.4910 |
| Middle SDI | Female | CD | 2003 | 33.2921 | 20.8620 | 49.3866 |
| Middle SDI | Both | CD | 2003 | 31.1760 | 19.6138 | 46.3303 |
| Middle SDI | Male | CD | 2002 | 29.0500 | 18.1993 | 43.7961 |
| Middle SDI | Female | CD | 2002 | 33.3003 | 20.9050 | 49.1299 |
| Middle SDI | Both | CD | 2002 | 31.1808 | 19.6549 | 46.4417 |
| Middle SDI | Male | CD | 2004 | 29.0379 | 18.0824 | 43.6512 |
| Middle SDI | Female | CD | 2004 | 33.2850 | 20.8799 | 49.2067 |
| Middle SDI | Both | CD | 2004 | 31.1695 | 19.6270 | 46.4617 |
| High SDI | Male | CD | 2003 | 21.5023 | 13.8943 | 31.9904 |
| High SDI | Female | CD | 2003 | 26.8285 | 17.2675 | 39.8763 |
| High SDI | Both | CD | 2003 | 24.1327 | 15.5353 | 36.1330 |
| Low-middle SDI | Male | CD | 2003 | 26.6202 | 16.6284 | 39.7398 |
| Low-middle SDI | Female | CD | 2003 | 30.3299 | 19.0991 | 44.7439 |
| Low-middle SDI | Both | CD | 2003 | 28.4726 | 17.9232 | 42.3575 |
| Low SDI | Male | CD | 2003 | 24.5228 | 15.2595 | 36.2694 |
| Low SDI | Female | CD | 2003 | 27.7354 | 17.4440 | 41.6057 |
| Low SDI | Both | CD | 2003 | 26.1302 | 16.3762 | 38.9352 |
| High-middle SDI | Male | CD | 2004 | 24.4082 | 15.3058 | 36.6800 |
| High-middle SDI | Female | CD | 2004 | 31.0072 | 19.7610 | 46.1504 |
| High-middle SDI | Both | CD | 2004 | 27.7278 | 17.4555 | 41.4016 |
| High SDI | Male | CD | 2004 | 21.5540 | 13.9283 | 32.2239 |
| High SDI | Female | CD | 2004 | 26.8832 | 17.3140 | 39.9248 |
| High SDI | Both | CD | 2004 | 24.1858 | 15.6351 | 36.0127 |
| Low-middle SDI | Male | CD | 2004 | 26.6237 | 16.5917 | 39.8965 |
| Low-middle SDI | Female | CD | 2004 | 30.3243 | 19.1896 | 44.6549 |
| Low-middle SDI | Both | CD | 2004 | 28.4738 | 17.8058 | 42.4783 |
| High-middle SDI | Male | CD | 2003 | 24.3859 | 15.3050 | 36.3610 |
| High-middle SDI | Female | CD | 2003 | 31.0269 | 19.5611 | 46.1737 |
| High-middle SDI | Both | CD | 2003 | 27.7282 | 17.4075 | 41.3125 |
| Low SDI | Male | CD | 2004 | 24.5245 | 15.2781 | 36.5676 |
| Low SDI | Female | CD | 2004 | 27.7406 | 17.5009 | 41.3272 |
| Low SDI | Both | CD | 2004 | 26.1347 | 16.3544 | 39.0343 |
| High-middle SDI | Male | CD | 2005 | 24.4353 | 15.3455 | 36.2674 |
| High-middle SDI | Female | CD | 2005 | 30.9890 | 19.7735 | 45.9624 |
| High-middle SDI | Both | CD | 2005 | 27.7310 | 17.4771 | 41.4901 |
| High SDI | Male | CD | 2005 | 21.5967 | 13.9007 | 32.1407 |
| High SDI | Female | CD | 2005 | 26.9368 | 17.2384 | 39.8643 |
| High SDI | Both | CD | 2005 | 24.2335 | 15.7077 | 36.2644 |
| Low-middle SDI | Male | CD | 2005 | 26.6150 | 16.7093 | 39.9127 |
| Low-middle SDI | Female | CD | 2005 | 30.3143 | 19.0633 | 44.7884 |
| Low-middle SDI | Both | CD | 2005 | 28.4666 | 17.7529 | 42.4679 |
| Low SDI | Male | CD | 2005 | 24.5316 | 15.2150 | 36.4115 |
| Low SDI | Female | CD | 2005 | 27.7573 | 17.4218 | 41.4808 |
| Low SDI | Both | CD | 2005 | 26.1477 | 16.3305 | 39.0627 |
| Middle SDI | Male | CD | 2005 | 29.0263 | 18.0365 | 43.6562 |
| Middle SDI | Female | CD | 2005 | 33.2759 | 20.9784 | 49.0919 |
| Middle SDI | Both | CD | 2005 | 31.1604 | 19.5357 | 46.4513 |
| Middle SDI | Male | CD | 2006 | 29.0173 | 18.0115 | 43.9943 |
| Middle SDI | Female | CD | 2006 | 33.2683 | 20.9501 | 49.1376 |
| Middle SDI | Both | CD | 2006 | 31.1533 | 19.5072 | 46.4211 |
| Low SDI | Male | CD | 2006 | 24.5304 | 15.2973 | 36.4985 |
| Low SDI | Female | CD | 2006 | 27.7694 | 17.4200 | 41.3037 |
| Low SDI | Both | CD | 2006 | 26.1542 | 16.4359 | 38.9523 |
| High-middle SDI | Male | CD | 2006 | 24.4643 | 15.4179 | 36.3845 |
| High-middle SDI | Female | CD | 2006 | 30.9915 | 19.7489 | 45.9508 |
| High-middle SDI | Both | CD | 2006 | 27.7448 | 17.5941 | 41.2908 |
| Low-middle SDI | Male | CD | 2006 | 26.6163 | 16.7196 | 39.6874 |
| Low-middle SDI | Female | CD | 2006 | 30.3248 | 18.9535 | 44.7301 |
| Low-middle SDI | Both | CD | 2006 | 28.4746 | 17.7988 | 42.4469 |
| High SDI | Male | CD | 2006 | 21.6385 | 13.9770 | 32.3748 |
| High SDI | Female | CD | 2006 | 26.9964 | 17.4051 | 40.3341 |
| High SDI | Both | CD | 2006 | 24.2832 | 15.7020 | 36.2195 |
| High-middle SDI | Male | CD | 2007 | 24.5029 | 15.3329 | 36.5387 |
| High-middle SDI | Female | CD | 2007 | 30.9833 | 19.4438 | 45.7040 |
| High-middle SDI | Both | CD | 2007 | 27.7578 | 17.4319 | 41.3825 |
| High SDI | Male | CD | 2008 | 21.7411 | 14.0510 | 32.4631 |
| High SDI | Female | CD | 2008 | 27.1122 | 17.4139 | 40.4037 |
| High SDI | Both | CD | 2008 | 24.3854 | 15.7184 | 36.4862 |
| Middle SDI | Male | CD | 2007 | 29.0196 | 18.1230 | 43.5187 |
| Middle SDI | Female | CD | 2007 | 33.2636 | 20.9996 | 49.1736 |
| Middle SDI | Both | CD | 2007 | 31.1530 | 19.5816 | 46.4920 |
| High SDI | Male | CD | 2007 | 21.6895 | 13.9620 | 32.3709 |
| High SDI | Female | CD | 2007 | 27.0556 | 17.4636 | 40.3666 |
| High SDI | Both | CD | 2007 | 24.3354 | 15.7145 | 36.4214 |
| Low-middle SDI | Male | CD | 2008 | 26.6112 | 16.6680 | 39.6649 |
| Low-middle SDI | Female | CD | 2008 | 30.3231 | 19.0953 | 44.9841 |
| Low-middle SDI | Both | CD | 2008 | 28.4755 | 17.8967 | 42.3575 |
| Low-middle SDI | Male | CD | 2007 | 26.6170 | 16.5844 | 39.9103 |
| Low-middle SDI | Female | CD | 2007 | 30.3212 | 19.1440 | 44.8296 |
| Low-middle SDI | Both | CD | 2007 | 28.4752 | 17.9458 | 42.5525 |
| Low SDI | Male | CD | 2008 | 24.5556 | 15.3264 | 36.3711 |
| Low SDI | Female | CD | 2008 | 27.7788 | 17.4995 | 41.3827 |
| Low SDI | Both | CD | 2008 | 26.1737 | 16.4636 | 38.8231 |
| Low SDI | Male | CD | 2007 | 24.5463 | 15.4069 | 36.4106 |
| Low SDI | Female | CD | 2007 | 27.7724 | 17.3283 | 41.5457 |
| Low SDI | Both | CD | 2007 | 26.1647 | 16.3634 | 39.0530 |
| Middle SDI | Male | CD | 2008 | 29.0044 | 18.0732 | 43.5204 |
| Middle SDI | Female | CD | 2008 | 33.2699 | 20.8842 | 49.1199 |
| Middle SDI | Both | CD | 2008 | 31.1497 | 19.5621 | 46.4542 |
| High-middle SDI | Male | CD | 2008 | 24.5443 | 15.3253 | 36.6328 |
| High-middle SDI | Female | CD | 2008 | 31.0013 | 19.7354 | 45.9484 |
| High-middle SDI | Both | CD | 2008 | 27.7854 | 17.6486 | 41.4959 |
| Middle SDI | Male | CD | 2009 | 28.9956 | 18.0449 | 43.5036 |
| Middle SDI | Female | CD | 2009 | 33.2725 | 21.0060 | 49.2238 |
| Middle SDI | Both | CD | 2009 | 31.1481 | 19.6679 | 46.4720 |
| High-middle SDI | Male | CD | 2009 | 24.5976 | 15.4138 | 36.7275 |
| High-middle SDI | Female | CD | 2009 | 30.9831 | 19.7000 | 46.0356 |
| High-middle SDI | Both | CD | 2009 | 27.8009 | 17.5859 | 41.4511 |
| High SDI | Male | CD | 2009 | 21.7860 | 13.9815 | 32.3516 |
| High SDI | Female | CD | 2009 | 27.1738 | 17.4430 | 40.5073 |
| High SDI | Both | CD | 2009 | 24.4350 | 15.8066 | 36.5216 |
| Low-middle SDI | Male | CD | 2009 | 26.6151 | 16.7107 | 39.8273 |
| Low-middle SDI | Female | CD | 2009 | 30.3240 | 19.1150 | 45.0384 |
| Low-middle SDI | Both | CD | 2009 | 28.4802 | 17.8535 | 42.4665 |
| High-middle SDI | Male | CD | 2010 | 24.6174 | 15.3922 | 36.8297 |
| High-middle SDI | Female | CD | 2010 | 30.9913 | 19.7685 | 46.2308 |
| High-middle SDI | Both | CD | 2010 | 27.8124 | 17.5945 | 41.3738 |
| Low SDI | Male | CD | 2009 | 24.5719 | 15.3771 | 36.7707 |
| Low SDI | Female | CD | 2009 | 27.7923 | 17.3512 | 41.3845 |
| Low SDI | Both | CD | 2009 | 26.1899 | 16.3866 | 39.0825 |
| High SDI | Male | CD | 2010 | 21.8472 | 14.0595 | 32.5522 |
| High SDI | Female | CD | 2010 | 27.2455 | 17.3934 | 40.4553 |
| High SDI | Both | CD | 2010 | 24.5001 | 15.8485 | 36.5219 |
| Low-middle SDI | Male | CD | 2010 | 26.6058 | 16.7341 | 39.6454 |
| Low-middle SDI | Female | CD | 2010 | 30.3036 | 18.9568 | 44.6081 |
| Low-middle SDI | Both | CD | 2010 | 28.4676 | 17.8880 | 42.5101 |
| Low SDI | Male | CD | 2010 | 24.5830 | 15.3524 | 36.7027 |
| Low SDI | Female | CD | 2010 | 27.7947 | 17.4853 | 41.3700 |
| Low SDI | Both | CD | 2010 | 26.1978 | 16.4370 | 39.0788 |
| Middle SDI | Male | CD | 2011 | 28.9656 | 17.9566 | 43.7015 |
| Middle SDI | Female | CD | 2011 | 33.2418 | 20.9891 | 49.1374 |
| Middle SDI | Both | CD | 2011 | 31.1205 | 19.5292 | 46.3133 |
| Middle SDI | Male | CD | 2010 | 28.9858 | 18.0315 | 43.8181 |
| Middle SDI | Female | CD | 2010 | 33.2627 | 21.0704 | 49.2613 |
| Middle SDI | Both | CD | 2010 | 31.1396 | 19.5658 | 46.3427 |
| High-middle SDI | Male | CD | 2011 | 24.6710 | 15.4771 | 36.8793 |
| High-middle SDI | Female | CD | 2011 | 30.9969 | 19.6706 | 45.8450 |
| High-middle SDI | Both | CD | 2011 | 27.8387 | 17.5915 | 41.6112 |
| Low-middle SDI | Male | CD | 2011 | 26.5993 | 16.6471 | 39.8203 |
| Low-middle SDI | Female | CD | 2011 | 30.3022 | 18.9707 | 44.8871 |
| Low-middle SDI | Both | CD | 2011 | 28.4657 | 17.8356 | 42.3919 |
| Low SDI | Male | CD | 2011 | 24.5819 | 15.3960 | 36.5378 |
| Low SDI | Female | CD | 2011 | 27.7996 | 17.4338 | 41.4441 |
| Low SDI | Both | CD | 2011 | 26.2009 | 16.4668 | 39.2187 |
| High SDI | Male | CD | 2011 | 21.8961 | 14.1155 | 32.4811 |
| High SDI | Female | CD | 2011 | 27.3014 | 17.4801 | 40.5767 |
| High SDI | Both | CD | 2011 | 24.5513 | 15.8395 | 36.7047 |
| Middle SDI | Male | CD | 2012 | 28.9604 | 17.9832 | 43.6729 |
| Middle SDI | Female | CD | 2012 | 33.2378 | 21.0207 | 49.2322 |
| Middle SDI | Both | CD | 2012 | 31.1172 | 19.5210 | 46.4311 |
| High-middle SDI | Male | CD | 2012 | 24.7257 | 15.4802 | 36.9800 |
| High-middle SDI | Female | CD | 2012 | 31.0106 | 19.7501 | 46.0209 |
| High-middle SDI | Both | CD | 2012 | 27.8695 | 17.5984 | 41.7166 |
| High SDI | Male | CD | 2013 | 21.9918 | 14.1410 | 32.7002 |
| High SDI | Female | CD | 2013 | 27.4174 | 17.6243 | 40.8885 |
| High SDI | Both | CD | 2013 | 24.6540 | 15.8997 | 36.8029 |
| Low-middle SDI | Male | CD | 2013 | 26.5783 | 16.6074 | 39.7699 |
| Low-middle SDI | Female | CD | 2013 | 30.2873 | 19.0626 | 44.8973 |
| Low-middle SDI | Both | CD | 2013 | 28.4515 | 17.8952 | 42.4462 |
| High SDI | Male | CD | 2012 | 21.9546 | 14.1203 | 32.4762 |
| High SDI | Female | CD | 2012 | 27.3575 | 17.5192 | 40.8589 |
| High SDI | Both | CD | 2012 | 24.6073 | 15.8982 | 36.6836 |
| Low SDI | Male | CD | 2013 | 24.5935 | 15.3220 | 36.6846 |
| Low SDI | Female | CD | 2013 | 27.8131 | 17.3935 | 41.3959 |
| Low SDI | Both | CD | 2013 | 26.2160 | 16.3571 | 39.1937 |
| Low-middle SDI | Male | CD | 2012 | 26.5939 | 16.5565 | 39.8043 |
| Low-middle SDI | Female | CD | 2012 | 30.2971 | 19.0327 | 44.8141 |
| Low-middle SDI | Both | CD | 2012 | 28.4624 | 17.8535 | 42.3558 |
| Low SDI | Male | CD | 2012 | 24.5800 | 15.2708 | 36.7269 |
| Low SDI | Female | CD | 2012 | 27.8047 | 17.5147 | 41.5306 |
| Low SDI | Both | CD | 2012 | 26.2039 | 16.3780 | 39.2653 |
| Middle SDI | Male | CD | 2013 | 28.9434 | 18.0171 | 43.5053 |
| Middle SDI | Female | CD | 2013 | 33.2285 | 20.8295 | 49.0353 |
| Middle SDI | Both | CD | 2013 | 31.1054 | 19.5685 | 46.2095 |
| High-middle SDI | Male | CD | 2013 | 24.7778 | 15.4864 | 36.9419 |
| High-middle SDI | Female | CD | 2013 | 31.0327 | 19.6766 | 46.1640 |
| High-middle SDI | Both | CD | 2013 | 27.9039 | 17.5828 | 41.6422 |
| Middle SDI | Male | CD | 2014 | 28.9410 | 17.9854 | 43.6191 |
| Middle SDI | Female | CD | 2014 | 33.2147 | 20.8830 | 49.2885 |
| Middle SDI | Both | CD | 2014 | 31.0982 | 19.5012 | 46.2107 |
| High-middle SDI | Male | CD | 2014 | 24.8285 | 15.4892 | 37.1399 |
| High-middle SDI | Female | CD | 2014 | 31.0554 | 19.7340 | 46.0707 |
| High-middle SDI | Both | CD | 2014 | 27.9379 | 17.5648 | 41.6796 |
| Low-middle SDI | Male | CD | 2014 | 26.5774 | 16.6026 | 39.8369 |
| Low-middle SDI | Female | CD | 2014 | 30.2682 | 18.9898 | 44.8230 |
| Low-middle SDI | Both | CD | 2014 | 28.4432 | 17.8187 | 42.3421 |
| High SDI | Male | CD | 2014 | 22.0405 | 14.1505 | 32.6392 |
| High SDI | Female | CD | 2014 | 27.4640 | 17.7666 | 40.9905 |
| High SDI | Both | CD | 2014 | 24.7003 | 15.9604 | 36.6854 |
| High-middle SDI | Male | CD | 2015 | 24.8879 | 15.5935 | 37.0876 |
| High-middle SDI | Female | CD | 2015 | 31.0688 | 19.6413 | 46.2642 |
| High-middle SDI | Both | CD | 2015 | 27.9712 | 17.6143 | 41.8095 |
| Low SDI | Male | CD | 2014 | 24.5964 | 15.4186 | 36.7713 |
| Low SDI | Female | CD | 2014 | 27.8176 | 17.5248 | 41.5837 |
| Low SDI | Both | CD | 2014 | 26.2204 | 16.3882 | 39.1946 |
| High SDI | Male | CD | 2015 | 22.0700 | 14.1726 | 32.8586 |
| High SDI | Female | CD | 2015 | 27.5121 | 17.7694 | 40.9276 |
| High SDI | Both | CD | 2015 | 24.7373 | 15.9991 | 36.8935 |
| Low-middle SDI | Male | CD | 2015 | 26.5623 | 16.6411 | 39.7684 |
| Low-middle SDI | Female | CD | 2015 | 30.2643 | 19.0900 | 44.8421 |
| Low-middle SDI | Both | CD | 2015 | 28.4351 | 17.8325 | 42.2748 |
| Low SDI | Male | CD | 2015 | 24.6112 | 15.3345 | 36.7207 |
| Low SDI | Female | CD | 2015 | 27.8064 | 17.3599 | 41.5814 |
| Low SDI | Both | CD | 2015 | 26.2231 | 16.3922 | 39.2553 |
| Middle SDI | Male | CD | 2015 | 28.9171 | 17.9797 | 43.3848 |
| Middle SDI | Female | CD | 2015 | 33.2033 | 20.9289 | 49.1583 |
| Middle SDI | Both | CD | 2015 | 31.0816 | 19.5727 | 46.1554 |
| High-middle SDI | Male | CD | 2016 | 24.9297 | 15.5161 | 37.3678 |
| High-middle SDI | Female | CD | 2016 | 31.0928 | 19.5943 | 46.3143 |
| High-middle SDI | Both | CD | 2016 | 28.0010 | 17.5617 | 41.7110 |
| High SDI | Male | CD | 2016 | 22.0984 | 14.2263 | 32.8315 |
| High SDI | Female | CD | 2016 | 27.5506 | 17.6931 | 41.0705 |
| High SDI | Both | CD | 2016 | 24.7692 | 16.0113 | 36.9701 |
| Middle SDI | Male | CD | 2016 | 28.9022 | 17.9416 | 43.5772 |
| Middle SDI | Female | CD | 2016 | 33.1874 | 20.9251 | 49.1340 |
| Middle SDI | Both | CD | 2016 | 31.0668 | 19.6313 | 46.1912 |
| Low-middle SDI | Male | CD | 2016 | 26.5510 | 16.5855 | 39.7091 |
| Low-middle SDI | Female | CD | 2016 | 30.2496 | 18.9642 | 44.7758 |
| Low-middle SDI | Both | CD | 2016 | 28.4234 | 17.7763 | 42.4618 |
| Low SDI | Male | CD | 2016 | 24.6053 | 15.2797 | 36.6329 |
| Low SDI | Female | CD | 2016 | 27.8223 | 17.3064 | 41.5936 |
| Low SDI | Both | CD | 2016 | 26.2290 | 16.3754 | 39.2583 |
| High SDI | Male | CD | 2017 | 22.1261 | 14.2343 | 33.0134 |
| High SDI | Female | CD | 2017 | 27.5762 | 17.7010 | 40.8917 |
| High SDI | Both | CD | 2017 | 24.7946 | 16.0311 | 37.1477 |
| Low SDI | Male | CD | 2018 | 24.5935 | 15.3729 | 36.8864 |
| Low SDI | Female | CD | 2018 | 27.8217 | 17.3940 | 41.3813 |
| Low SDI | Both | CD | 2018 | 26.2242 | 16.3355 | 39.1413 |
| Middle SDI | Male | CD | 2017 | 28.8875 | 17.9400 | 43.3893 |
| Middle SDI | Female | CD | 2017 | 33.1690 | 20.9140 | 49.0264 |
| Middle SDI | Both | CD | 2017 | 31.0508 | 19.5195 | 46.2068 |
| Low SDI | Male | CD | 2017 | 24.5941 | 15.3569 | 36.6179 |
| Low SDI | Female | CD | 2017 | 27.8221 | 17.4170 | 41.4987 |
| Low SDI | Both | CD | 2017 | 26.2240 | 16.3721 | 39.2111 |
| Low-middle SDI | Male | CD | 2018 | 26.5346 | 16.5602 | 39.6072 |
| Low-middle SDI | Female | CD | 2018 | 30.2280 | 18.9111 | 44.5691 |
| Low-middle SDI | Both | CD | 2018 | 28.4067 | 17.7564 | 42.3073 |
| High-middle SDI | Male | CD | 2017 | 24.9839 | 15.7332 | 37.3374 |
| High-middle SDI | Female | CD | 2017 | 31.1170 | 19.7711 | 46.0853 |
| High-middle SDI | Both | CD | 2017 | 28.0373 | 17.6286 | 41.8246 |
| Low-middle SDI | Male | CD | 2017 | 26.5570 | 16.4735 | 39.6443 |
| Low-middle SDI | Female | CD | 2017 | 30.2480 | 18.8399 | 44.5607 |
| Low-middle SDI | Both | CD | 2017 | 28.4269 | 17.7527 | 42.2781 |
| High-middle SDI | Male | CD | 2018 | 25.0248 | 15.6376 | 37.5259 |
| High-middle SDI | Female | CD | 2018 | 31.1430 | 19.8249 | 46.1701 |
| High-middle SDI | Both | CD | 2018 | 28.0681 | 17.8140 | 42.0101 |
| High SDI | Male | CD | 2018 | 22.1402 | 14.2490 | 33.0159 |
| High SDI | Female | CD | 2018 | 27.5970 | 17.7910 | 40.9925 |
| High SDI | Both | CD | 2018 | 24.8107 | 16.0748 | 36.9414 |
| Middle SDI | Male | CD | 2018 | 28.8719 | 17.9672 | 43.3234 |
| Middle SDI | Female | CD | 2018 | 33.1543 | 20.7222 | 49.2152 |
| Middle SDI | Both | CD | 2018 | 31.0361 | 19.5495 | 46.1761 |
| High-middle SDI | Male | CD | 2019 | 25.0755 | 15.7233 | 37.4380 |
| High-middle SDI | Female | CD | 2019 | 31.1622 | 19.7450 | 46.2027 |
| High-middle SDI | Both | CD | 2019 | 28.1006 | 17.6757 | 41.8087 |
| High SDI | Male | CD | 2019 | 22.1527 | 14.2803 | 32.9251 |
| High SDI | Female | CD | 2019 | 27.6082 | 17.7813 | 41.0198 |
| High SDI | Both | CD | 2019 | 24.8216 | 16.0310 | 37.0273 |
| Low-middle SDI | Male | CD | 2019 | 26.5294 | 16.6221 | 39.7729 |
| Low-middle SDI | Female | CD | 2019 | 30.2236 | 19.0038 | 44.7158 |
| Low-middle SDI | Both | CD | 2019 | 28.4028 | 17.8174 | 42.1527 |
| Middle SDI | Male | CD | 2019 | 28.8576 | 17.8901 | 43.5244 |
| Middle SDI | Female | CD | 2019 | 33.1461 | 20.7863 | 49.3044 |
| Middle SDI | Both | CD | 2019 | 31.0249 | 19.5010 | 46.1529 |
| Low SDI | Male | CD | 2019 | 24.6029 | 15.3313 | 36.7997 |
| Low SDI | Female | CD | 2019 | 27.8282 | 17.2965 | 41.4135 |
| Low SDI | Both | CD | 2019 | 26.2326 | 16.4093 | 39.1330 |
|  |  |  |  |  |  |  |

**Table18 Incidence of CD in 2019, Page 87-91**

| **location_name** | **sex_name** | **cause_name** | **year** | **Age-standardised incidence rate (per 100 000 person-years)** | **95%CI(lower)** | **95%CI(upper)** |
| --- | --- | --- | --- | --- | --- | --- |
| China | Both | CD | 2019 | 3574.5324 | 2803.8133 | 4400.4634 |
| Democratic People's Republic of Korea | Both | CD | 2019 | 3126.0359 | 2432.7776 | 3837.7789 |
| Tonga | Both | CD | 2019 | 3126.7212 | 2429.4847 | 3836.9277 |
| Philippines | Both | CD | 2019 | 3845.6832 | 2987.8697 | 4710.7685 |
| Serbia | Both | CD | 2019 | 3597.7788 | 2826.2801 | 4407.1251 |
| Vanuatu | Both | CD | 2019 | 3114.3434 | 2421.2013 | 3826.3891 |
| Slovakia | Both | CD | 2019 | 3596.5416 | 2824.9869 | 4405.7308 |
| Armenia | Both | CD | 2019 | 3634.8719 | 2855.7587 | 4450.8805 |
| Guyana | Both | CD | 2019 | 2979.9291 | 2314.7651 | 3643.4258 |
| Uzbekistan | Both | CD | 2019 | 3620.8619 | 2845.5075 | 4435.2553 |
| Taiwan (Province of China) | Both | CD | 2019 | 3121.0280 | 2425.8552 | 3832.1566 |
| Bhutan | Both | CD | 2019 | 2734.9425 | 2162.6694 | 3351.3240 |
| Slovenia | Both | CD | 2019 | 3577.3961 | 2808.4191 | 4386.3165 |
| Qatar | Both | CD | 2019 | 2812.0091 | 2188.8865 | 3416.5662 |
| Oman | Both | CD | 2019 | 2868.7762 | 2234.3769 | 3488.1309 |
| Albania | Both | CD | 2019 | 3602.6033 | 2830.2190 | 4417.0313 |
| Cambodia | Both | CD | 2019 | 3353.7478 | 2627.0169 | 4135.8137 |
| Canada | Both | CD | 2019 | 3285.9838 | 2556.0795 | 4063.7748 |
| Sri Lanka | Both | CD | 2019 | 3530.4878 | 2757.2066 | 4335.9678 |
| Norway | Both | CD | 2019 | 1210.7310 | 940.1170 | 1510.9817 |
| Viet Nam | Both | CD | 2019 | 3347.7099 | 2619.9132 | 4129.6897 |
| USA | Both | CD | 2019 | 4408.2936 | 3514.1266 | 5389.1386 |
| Timor-Leste | Both | CD | 2019 | 3336.5949 | 2608.8653 | 4119.7912 |
| Algeria | Both | CD | 2019 | 2925.5526 | 2283.3065 | 3562.6463 |
| Bosnia and Herzegovina | Both | CD | 2019 | 3607.3917 | 2835.5586 | 4419.7823 |
| Portugal | Both | CD | 2019 | 811.9272 | 635.5502 | 1008.6948 |
| India | Both | CD | 2019 | 3174.8502 | 2470.0701 | 3922.5275 |
| Indonesia | Both | CD | 2019 | 3844.1557 | 2986.3384 | 4706.2890 |
| Thailand | Both | CD | 2019 | 3345.2068 | 2617.2242 | 4127.3316 |
| Comoros | Both | CD | 2019 | 2665.2773 | 2105.2794 | 3247.4071 |
| Brunei Darussalam | Both | CD | 2019 | 898.9626 | 709.6566 | 1096.3278 |
| Bulgaria | Both | CD | 2019 | 3591.8071 | 2821.2856 | 4399.7467 |
| Azerbaijan | Both | CD | 2019 | 3614.4639 | 2839.4704 | 4426.7541 |
| Bahrain | Both | CD | 2019 | 2878.9725 | 2243.2940 | 3504.1446 |
| Lao People's Democratic Republic | Both | CD | 2019 | 3339.7819 | 2613.8274 | 4120.7763 |
| Singapore | Both | CD | 2019 | 900.9221 | 712.2810 | 1098.5860 |
| Croatia | Both | CD | 2019 | 3598.7635 | 2826.8988 | 4409.7858 |
| Mauritania | Both | CD | 2019 | 2684.2790 | 2105.0660 | 3304.9553 |
| Greece | Both | CD | 2019 | 811.0107 | 634.8803 | 1007.6347 |
| Japan | Both | CD | 2019 | 1034.8840 | 821.8084 | 1273.8224 |
| Czechia | Both | CD | 2019 | 3589.1372 | 2819.9023 | 4399.0353 |
| Nepal | Both | CD | 2019 | 2758.2584 | 2176.1017 | 3384.7717 |
| Belarus | Both | CD | 2019 | 3355.6996 | 2646.4135 | 4073.6122 |
| Haiti | Both | CD | 2019 | 2984.0119 | 2316.5071 | 3646.8315 |
| Marshall Islands | Both | CD | 2019 | 3112.2619 | 2420.3443 | 3823.2394 |
| Cyprus | Both | CD | 2019 | 811.3547 | 635.1235 | 1007.8270 |
| Djibouti | Both | CD | 2019 | 2648.2150 | 2094.2370 | 3223.1981 |
| Saint Lucia | Both | CD | 2019 | 2976.3506 | 2312.1634 | 3639.2924 |
| Fiji | Both | CD | 2019 | 3120.6904 | 2424.7524 | 3829.5500 |
| Georgia | Both | CD | 2019 | 3615.0525 | 2839.7992 | 4421.9953 |
| Iceland | Both | CD | 2019 | 808.9874 | 633.2398 | 1005.0054 |
| Republic of Korea | Both | CD | 2019 | 900.5295 | 711.1904 | 1098.6214 |
| Pakistan | Both | CD | 2019 | 3155.7724 | 2460.3996 | 3870.5793 |
| Spain | Both | CD | 2019 | 810.1741 | 634.1907 | 1006.4629 |
| Hungary | Both | CD | 2019 | 3606.0139 | 2833.1335 | 4415.2136 |
| Australia | Both | CD | 2019 | 906.0157 | 715.1804 | 1104.4798 |
| Micronesia (Federated States of) | Both | CD | 2019 | 3120.7498 | 2425.5949 | 3829.7024 |
| Niger | Both | CD | 2019 | 2685.5569 | 2105.9986 | 3305.6612 |
| Denmark | Both | CD | 2019 | 395.7862 | 308.7401 | 488.5793 |
| Costa Rica | Both | CD | 2019 | 2983.3710 | 2315.8037 | 3645.4503 |
| Iran (Islamic Republic of) | Both | CD | 2019 | 3372.9383 | 2630.8077 | 4134.1716 |
| Saint Vincent and the Grenadines | Both | CD | 2019 | 2967.9856 | 2307.5562 | 3630.3975 |
| Latvia | Both | CD | 2019 | 3349.2249 | 2641.4667 | 4063.2548 |
| Antigua and Barbuda | Both | CD | 2019 | 2980.5671 | 2314.0405 | 3641.8524 |
| Cameroon | Both | CD | 2019 | 2684.1993 | 2105.1208 | 3302.6778 |
| Kiribati | Both | CD | 2019 | 3135.9703 | 2441.7956 | 3847.1165 |
| Eritrea | Both | CD | 2019 | 2670.6811 | 2111.7959 | 3252.0174 |
| Estonia | Both | CD | 2019 | 3334.3160 | 2630.1516 | 4041.4745 |
| Ireland | Both | CD | 2019 | 811.1369 | 634.9595 | 1008.0283 |
| North Macedonia | Both | CD | 2019 | 3580.1524 | 2811.5999 | 4387.8364 |
| United Republic of Tanzania | Both | CD | 2019 | 2669.1544 | 2109.0187 | 3249.8852 |
| Jamaica | Both | CD | 2019 | 2976.3340 | 2312.4834 | 3639.0747 |
| Kazakhstan | Both | CD | 2019 | 3633.1139 | 2855.2016 | 4449.3948 |
| New Zealand | Both | CD | 2019 | 1040.8344 | 826.6668 | 1281.1145 |
| Papua New Guinea | Both | CD | 2019 | 3110.2122 | 2416.9999 | 3820.4344 |
| Malaysia | Both | CD | 2019 | 3330.4596 | 2604.6766 | 4108.7629 |
| Egypt | Both | CD | 2019 | 2915.7607 | 2274.4635 | 3552.2378 |
| Lithuania | Both | CD | 2019 | 3352.7270 | 2644.4858 | 4069.1639 |
| Nigeria | Both | CD | 2019 | 3152.6123 | 2432.4516 | 3874.1454 |
| Trinidad and Tobago | Both | CD | 2019 | 2973.9835 | 2311.2606 | 3637.4028 |
| Suriname | Both | CD | 2019 | 2980.2049 | 2315.4985 | 3644.5118 |
| Cabo Verde | Both | CD | 2019 | 2685.2685 | 2107.3780 | 3306.1893 |
| Bahamas | Both | CD | 2019 | 2984.1376 | 2316.6304 | 3647.0763 |
| Maldives | Both | CD | 2019 | 3292.5534 | 2570.3147 | 4055.5606 |
| Somalia | Both | CD | 2019 | 2669.7711 | 2109.5587 | 3251.0341 |
| Finland | Both | CD | 2019 | 809.2217 | 633.2984 | 1005.1991 |
| Sweden | Both | CD | 2019 | 906.5479 | 708.9264 | 1127.5964 |
| Andorra | Both | CD | 2019 | 808.4759 | 632.1512 | 1004.4092 |
| Palau | Both | CD | 2019 | 3099.5578 | 2409.1575 | 3801.8747 |
| Ethiopia | Both | CD | 2019 | 3055.2747 | 2386.7107 | 3727.8262 |
| Switzerland | Both | CD | 2019 | 809.3602 | 633.5130 | 1005.5943 |
| Uganda | Both | CD | 2019 | 2674.4035 | 2113.6536 | 3259.3136 |
| Samoa | Both | CD | 2019 | 3115.0876 | 2421.2610 | 3825.2227 |
| El Salvador | Both | CD | 2019 | 2996.7638 | 2322.0647 | 3657.5164 |
| Kuwait | Both | CD | 2019 | 2903.9615 | 2269.0354 | 3541.1932 |
| Kenya | Both | CD | 2019 | 3062.2384 | 2391.5637 | 3733.7582 |
| Republic of Moldova | Both | CD | 2019 | 3353.0296 | 2648.8726 | 4064.6916 |
| Saudi Arabia | Both | CD | 2019 | 2885.1304 | 2249.8199 | 3511.3892 |
| Israel | Both | CD | 2019 | 810.5766 | 634.4737 | 1007.1056 |
| San Marino | Both | CD | 2019 | 813.3013 | 636.9335 | 1010.6499 |
| Uruguay | Both | CD | 2019 | 908.2955 | 716.5352 | 1107.6830 |
| Guam | Both | CD | 2019 | 3113.5213 | 2419.2394 | 3820.8310 |
| Namibia | Both | CD | 2019 | 2674.7951 | 2113.2431 | 3259.7481 |
| Angola | Both | CD | 2019 | 2673.7846 | 2113.0930 | 3257.7745 |
| Russian Federation | Both | CD | 2019 | 3168.2304 | 2587.7884 | 3740.5205 |
| Sao Tome and Principe | Both | CD | 2019 | 2681.6762 | 2103.1979 | 3300.5352 |
| Central African Republic | Both | CD | 2019 | 2672.2751 | 2113.4004 | 3254.0414 |
| France | Both | CD | 2019 | 1449.0332 | 1109.9946 | 1829.7963 |
| Iraq | Both | CD | 2019 | 2926.3578 | 2285.2059 | 3562.7202 |
| Kyrgyzstan | Both | CD | 2019 | 3625.6314 | 2849.3792 | 4439.6840 |
| Chad | Both | CD | 2019 | 2677.6240 | 2099.2169 | 3295.4732 |
| Senegal | Both | CD | 2019 | 2685.3042 | 2107.1467 | 3304.5182 |
| Lesotho | Both | CD | 2019 | 2673.4103 | 2114.0642 | 3254.5494 |
| Saint Kitts and Nevis | Both | CD | 2019 | 2974.5949 | 2312.0035 | 3638.6560 |
| Puerto Rico | Both | CD | 2019 | 2984.7830 | 2316.7328 | 3647.3698 |
| United Kingdom | Both | CD | 2019 | 896.0841 | 703.0000 | 1111.8681 |
| Ecuador | Both | CD | 2019 | 3150.9615 | 2469.8173 | 3859.5666 |
| Syrian Arab Republic | Both | CD | 2019 | 2938.2386 | 2290.6475 | 3581.8544 |
| Chile | Both | CD | 2019 | 906.7211 | 715.4158 | 1106.0177 |
| Tajikistan | Both | CD | 2019 | 3605.8252 | 2832.8443 | 4419.9304 |
| Equatorial Guinea | Both | CD | 2019 | 2667.2371 | 2108.0461 | 3248.7133 |
| Sudan | Both | CD | 2019 | 2923.2287 | 2281.6188 | 3558.3887 |
| Jordan | Both | CD | 2019 | 2910.8609 | 2274.1192 | 3542.7797 |
| Italy | Both | CD | 2019 | 881.2916 | 690.3683 | 1098.4143 |
| Mongolia | Both | CD | 2019 | 3626.4407 | 2850.6406 | 4439.7010 |
| Belgium | Both | CD | 2019 | 810.0718 | 634.0215 | 1006.3837 |
| Congo | Both | CD | 2019 | 2664.0150 | 2105.8466 | 3244.0280 |
| Tuvalu | Both | CD | 2019 | 3115.7544 | 2421.6807 | 3825.3438 |
| Colombia | Both | CD | 2019 | 2982.7288 | 2314.9553 | 3644.6337 |
| Grenada | Both | CD | 2019 | 2971.9814 | 2310.7395 | 3636.1272 |
| Montenegro | Both | CD | 2019 | 3602.0597 | 2830.8467 | 4414.2324 |
| Paraguay | Both | CD | 2019 | 2974.1060 | 2310.9930 | 3637.0968 |
| Turkmenistan | Both | CD | 2019 | 3581.9562 | 2809.6348 | 4386.2956 |
| Argentina | Both | CD | 2019 | 907.3606 | 716.0556 | 1106.9870 |
| Bolivia (Plurinational State of) | Both | CD | 2019 | 3150.1803 | 2468.9295 | 3858.8166 |
| Guatemala | Both | CD | 2019 | 2988.6390 | 2317.3036 | 3648.5150 |
| Barbados | Both | CD | 2019 | 2982.9831 | 2315.6333 | 3645.3709 |
| Togo | Both | CD | 2019 | 2696.1314 | 2116.6652 | 3316.8466 |
| Sierra Leone | Both | CD | 2019 | 2680.6937 | 2101.8616 | 3298.7475 |
| Democratic Republic of the Congo | Both | CD | 2019 | 2666.8299 | 2107.5486 | 3247.7940 |
| Lebanon | Both | CD | 2019 | 2938.7035 | 2295.8473 | 3576.4286 |
| Tokelau | Both | CD | 2019 | 3113.7752 | 2419.7826 | 3823.1255 |
| Malta | Both | CD | 2019 | 808.9610 | 633.1598 | 1004.8963 |
| Poland | Both | CD | 2019 | 4113.0609 | 3229.3043 | 5002.2911 |
| Romania | Both | CD | 2019 | 3596.1647 | 2825.5972 | 4404.4325 |
| Luxembourg | Both | CD | 2019 | 809.0995 | 632.8004 | 1005.1015 |
| Peru | Both | CD | 2019 | 3268.8191 | 2584.3221 | 4005.2623 |
| Solomon Islands | Both | CD | 2019 | 3115.8358 | 2423.1050 | 3828.0070 |
| Bangladesh | Both | CD | 2019 | 2745.2326 | 2167.7597 | 3365.9772 |
| Germany | Both | CD | 2019 | 1331.8075 | 1045.8916 | 1637.0071 |
| Botswana | Both | CD | 2019 | 2672.1866 | 2110.6674 | 3254.4411 |
| Palestine | Both | CD | 2019 | 2929.2206 | 2288.9477 | 3565.0704 |
| Ukraine | Both | CD | 2019 | 3758.8231 | 2950.4768 | 4570.3032 |
| Austria | Both | CD | 2019 | 809.6912 | 633.6377 | 1006.1307 |
| Malawi | Both | CD | 2019 | 2672.2635 | 2111.9795 | 3254.6528 |
| Belize | Both | CD | 2019 | 2973.4163 | 2309.3883 | 3634.5515 |
| United States Virgin Islands | Both | CD | 2019 | 2987.2237 | 2319.1445 | 3650.8165 |
| Honduras | Both | CD | 2019 | 2984.2434 | 2316.9406 | 3647.6775 |
| Madagascar | Both | CD | 2019 | 2663.6897 | 2104.8769 | 3243.9121 |
| Zambia | Both | CD | 2019 | 2664.2382 | 2105.2410 | 3244.0281 |
| South Sudan | Both | CD | 2019 | 2662.0551 | 2102.5795 | 3245.0126 |
| Netherlands | Both | CD | 2019 | 613.7026 | 482.4319 | 754.1270 |
| Eswatini | Both | CD | 2019 | 2679.3139 | 2120.2817 | 3263.0053 |
| Monaco | Both | CD | 2019 | 811.0977 | 635.3481 | 1007.8416 |
| Zimbabwe | Both | CD | 2019 | 2678.9534 | 2116.6615 | 3265.3067 |
| Libya | Both | CD | 2019 | 2921.6477 | 2280.7562 | 3558.6399 |
| Mexico | Both | CD | 2019 | 3431.5576 | 2701.3184 | 4212.7357 |
| Cuba | Both | CD | 2019 | 2975.2168 | 2310.8379 | 3637.4869 |
| Mauritius | Both | CD | 2019 | 3342.6939 | 2614.8986 | 4125.0391 |
| South Africa | Both | CD | 2019 | 3069.2588 | 2401.8302 | 3742.8678 |
| Panama | Both | CD | 2019 | 2973.6375 | 2310.4226 | 3635.8814 |
| Myanmar | Both | CD | 2019 | 3354.6931 | 2625.3546 | 4136.7515 |
| C么te d'Ivoire | Both | CD | 2019 | 2672.6833 | 2093.9334 | 3289.7493 |
| Nicaragua | Both | CD | 2019 | 2983.9848 | 2315.6347 | 3646.0033 |
| Dominica | Both | CD | 2019 | 2969.2452 | 2308.5059 | 3632.6597 |
| Nauru | Both | CD | 2019 | 3126.4774 | 2433.0684 | 3837.3016 |
| Venezuela (Bolivarian Republic of) | Both | CD | 2019 | 2980.7492 | 2315.8864 | 3644.9549 |
| Gabon | Both | CD | 2019 | 2669.6353 | 2109.5959 | 3250.8630 |
| Morocco | Both | CD | 2019 | 2929.2801 | 2286.8810 | 3566.4211 |
| Tunisia | Both | CD | 2019 | 2932.4913 | 2289.0337 | 3569.9385 |
| Burundi | Both | CD | 2019 | 2657.0030 | 2098.6864 | 3232.4667 |
| Dominican Republic | Both | CD | 2019 | 2974.4515 | 2311.1681 | 3637.4502 |
| Gambia | Both | CD | 2019 | 2684.5831 | 2105.4276 | 3303.6389 |
| Benin | Both | CD | 2019 | 2688.6344 | 2109.5311 | 3308.6207 |
| Turkey | Both | CD | 2019 | 2929.4285 | 2288.0900 | 3565.7171 |
| Brazil | Both | CD | 2019 | 3432.1517 | 2701.7761 | 4214.5675 |
| Burkina Faso | Both | CD | 2019 | 2694.3127 | 2115.1003 | 3316.0252 |
| United Arab Emirates | Both | CD | 2019 | 2834.8619 | 2205.3034 | 3449.5582 |
| Cook Islands | Both | CD | 2019 | 3126.6546 | 2431.3908 | 3839.2957 |
| American Samoa | Both | CD | 2019 | 3121.4535 | 2425.7254 | 3831.4312 |
| Mozambique | Both | CD | 2019 | 2676.1545 | 2115.0573 | 3260.5535 |
| Greenland | Both | CD | 2019 | 3254.6756 | 2531.3279 | 4024.9324 |
| Rwanda | Both | CD | 2019 | 2675.9514 | 2114.2933 | 3261.2936 |
| Bermuda | Both | CD | 2019 | 2980.4811 | 2315.5877 | 3645.2739 |
| Yemen | Both | CD | 2019 | 2929.5147 | 2287.2086 | 3566.2360 |
| Afghanistan | Both | CD | 2019 | 2928.5015 | 2287.8618 | 3567.3628 |
| Ghana | Both | CD | 2019 | 2694.5154 | 2115.2842 | 3316.7257 |
| Guinea | Both | CD | 2019 | 2687.4418 | 2107.6957 | 3307.7849 |
| Guinea-Bissau | Both | CD | 2019 | 2693.1491 | 2113.7182 | 3314.0391 |
| Liberia | Both | CD | 2019 | 2677.2975 | 2098.7288 | 3294.5268 |
| Seychelles | Both | CD | 2019 | 3326.5825 | 2602.1384 | 4103.5356 |
| Mali | Both | CD | 2019 | 2680.7392 | 2102.3648 | 3300.6671 |
| Niue | Both | CD | 2019 | 3121.5492 | 2426.3816 | 3830.2787 |
| Northern Mariana Islands | Both | CD | 2019 | 3110.4417 | 2416.6756 | 3817.8967 |
|  |  |  |  |  |  |  |

**Table19 DALYs of CD in 2019, Page 92-96**

| **location_name** | **sex_name** | **cause_name** | **year** | **Age-standardised DALYs rate (per 100 000 person-years)** | **95%CI(lower)** | **95%CI(upper)** |
| --- | --- | --- | --- | --- | --- | --- |
| Argentina | Both | CD | 2019 | 8.1277 | 5.0904 | 12.2754 |
| Malaysia | Both | CD | 2019 | 30.8219 | 19.3335 | 46.0845 |
| Suriname | Both | CD | 2019 | 26.9820 | 16.9640 | 40.8881 |
| Myanmar | Both | CD | 2019 | 30.9625 | 19.5047 | 46.0670 |
| Kazakhstan | Both | CD | 2019 | 33.4782 | 21.2439 | 49.8834 |
| Trinidad and Tobago | Both | CD | 2019 | 27.0298 | 17.0739 | 40.4020 |
| Kyrgyzstan | Both | CD | 2019 | 33.5961 | 21.2026 | 50.2109 |
| Papua New Guinea | Both | CD | 2019 | 28.1333 | 17.5387 | 41.8580 |
| Micronesia (Federated States of) | Both | CD | 2019 | 28.3366 | 17.6141 | 42.1178 |
| China | Both | CD | 2019 | 32.5782 | 20.4091 | 48.7323 |
| Maldives | Both | CD | 2019 | 30.6208 | 19.1493 | 45.7152 |
| Lao People's Democratic Republic | Both | CD | 2019 | 30.9448 | 19.3138 | 46.2389 |
| Azerbaijan | Both | CD | 2019 | 33.4558 | 21.2614 | 49.5887 |
| Latvia | Both | CD | 2019 | 30.6874 | 19.6193 | 46.4795 |
| Italy | Both | CD | 2019 | 8.1497 | 5.1556 | 12.4353 |
| Samoa | Both | CD | 2019 | 28.3596 | 17.5498 | 42.6388 |
| Hungary | Both | CD | 2019 | 33.2455 | 20.7782 | 49.9217 |
| Philippines | Both | CD | 2019 | 34.9405 | 21.8821 | 51.6823 |
| Luxembourg | Both | CD | 2019 | 7.2335 | 4.4846 | 10.9760 |
| Mongolia | Both | CD | 2019 | 33.5352 | 20.9988 | 49.6302 |
| Czechia | Both | CD | 2019 | 33.0002 | 21.0389 | 49.0418 |
| Solomon Islands | Both | CD | 2019 | 28.3144 | 17.7270 | 42.8547 |
| Armenia | Both | CD | 2019 | 33.6166 | 21.1243 | 49.9851 |
| Mexico | Both | CD | 2019 | 30.7232 | 19.1279 | 46.0770 |
| Sri Lanka | Both | CD | 2019 | 33.1592 | 20.6812 | 49.2109 |
| Georgia | Both | CD | 2019 | 33.3914 | 20.8572 | 49.7096 |
| Belize | Both | CD | 2019 | 27.0933 | 16.8390 | 40.5851 |
| Fiji | Both | CD | 2019 | 28.2550 | 17.7337 | 42.3099 |
| Uruguay | Both | CD | 2019 | 8.1232 | 5.0861 | 12.2126 |
| Belgium | Both | CD | 2019 | 7.2335 | 4.4678 | 11.1263 |
| Cuba | Both | CD | 2019 | 27.0545 | 16.7410 | 40.3453 |
| Lithuania | Both | CD | 2019 | 30.7050 | 19.2902 | 46.2374 |
| Malta | Both | CD | 2019 | 7.2310 | 4.5309 | 11.1176 |
| Chile | Both | CD | 2019 | 8.1212 | 5.1296 | 12.3619 |
| Tonga | Both | CD | 2019 | 28.4782 | 17.9972 | 42.6942 |
| Iceland | Both | CD | 2019 | 7.2771 | 4.5568 | 11.2003 |
| Iran (Islamic Republic of) | Both | CD | 2019 | 30.2232 | 18.7822 | 45.0243 |
| Belarus | Both | CD | 2019 | 30.7256 | 19.5472 | 46.3938 |
| North Macedonia | Both | CD | 2019 | 33.0276 | 20.9789 | 49.3215 |
| Nicaragua | Both | CD | 2019 | 27.1142 | 17.0160 | 40.3973 |
| Barbados | Both | CD | 2019 | 27.2080 | 16.9995 | 41.0740 |
| Iraq | Both | CD | 2019 | 26.4205 | 16.5089 | 39.0918 |
| Malawi | Both | CD | 2019 | 24.1575 | 14.9415 | 36.5304 |
| Thailand | Both | CD | 2019 | 31.0769 | 19.3672 | 46.3438 |
| Estonia | Both | CD | 2019 | 30.5586 | 19.4469 | 45.5639 |
| Australia | Both | CD | 2019 | 8.0644 | 5.0498 | 12.0316 |
| Montenegro | Both | CD | 2019 | 33.2007 | 20.7847 | 49.4149 |
| Bermuda | Both | CD | 2019 | 27.2641 | 16.8905 | 41.0206 |
| Syrian Arab Republic | Both | CD | 2019 | 26.5725 | 16.5104 | 39.7448 |
| Democratic People's Republic of Korea | Both | CD | 2019 | 28.7630 | 18.0667 | 43.1562 |
| Republic of Moldova | Both | CD | 2019 | 30.6996 | 19.5992 | 46.0383 |
| Kiribati | Both | CD | 2019 | 28.3903 | 17.6348 | 42.5398 |
| Pakistan | Both | CD | 2019 | 28.1892 | 17.7432 | 42.0692 |
| Bosnia and Herzegovina | Both | CD | 2019 | 33.2365 | 21.0549 | 49.2157 |
| Panama | Both | CD | 2019 | 27.1002 | 16.9597 | 40.6348 |
| Ireland | Both | CD | 2019 | 7.2532 | 4.4669 | 11.2227 |
| Bolivia (Plurinational State of) | Both | CD | 2019 | 28.9066 | 18.2453 | 43.2959 |
| Jordan | Both | CD | 2019 | 26.5204 | 16.6767 | 39.7189 |
| Poland | Both | CD | 2019 | 37.5654 | 23.4460 | 56.3927 |
| Taiwan (Province of China) | Both | CD | 2019 | 28.7320 | 17.8734 | 43.2449 |
| Marshall Islands | Both | CD | 2019 | 28.1744 | 17.2869 | 42.2742 |
| New Zealand | Both | CD | 2019 | 9.2741 | 5.8168 | 13.7536 |
| Ecuador | Both | CD | 2019 | 28.9699 | 18.1037 | 43.5313 |
| Cook Islands | Both | CD | 2019 | 28.4080 | 17.7828 | 42.7142 |
| Tunisia | Both | CD | 2019 | 26.6485 | 16.4875 | 40.0266 |
| Tajikistan | Both | CD | 2019 | 33.3534 | 21.0913 | 49.3970 |
| Romania | Both | CD | 2019 | 33.2688 | 20.8596 | 49.7049 |
| Dominica | Both | CD | 2019 | 27.0145 | 16.8359 | 40.2742 |
| C么te d'Ivoire | Both | CD | 2019 | 24.2909 | 15.0745 | 36.7552 |
| Israel | Both | CD | 2019 | 7.2780 | 4.5350 | 11.3263 |
| Peru | Both | CD | 2019 | 30.5428 | 19.0890 | 45.5493 |
| Bahrain | Both | CD | 2019 | 26.0898 | 16.2308 | 38.4566 |
| Andorra | Both | CD | 2019 | 7.2346 | 4.4750 | 10.9962 |
| Turkmenistan | Both | CD | 2019 | 33.2178 | 20.7058 | 49.6126 |
| Dominican Republic | Both | CD | 2019 | 27.0756 | 17.0080 | 40.6829 |
| United Kingdom | Both | CD | 2019 | 8.0386 | 5.0152 | 12.1454 |
| Puerto Rico | Both | CD | 2019 | 27.1571 | 16.9676 | 40.7190 |
| Turkey | Both | CD | 2019 | 26.6738 | 16.5933 | 39.6015 |
| Slovakia | Both | CD | 2019 | 33.1514 | 20.9343 | 49.2872 |
| Egypt | Both | CD | 2019 | 26.5432 | 16.6884 | 39.8833 |
| Brunei Darussalam | Both | CD | 2019 | 8.0625 | 5.1533 | 12.1928 |
| France | Both | CD | 2019 | 16.0359 | 9.8073 | 24.5340 |
| Saint Kitts and Nevis | Both | CD | 2019 | 27.0235 | 16.9289 | 40.5531 |
| Austria | Both | CD | 2019 | 7.2273 | 4.4916 | 11.0182 |
| Gambia | Both | CD | 2019 | 24.3871 | 15.1866 | 36.4239 |
| Slovenia | Both | CD | 2019 | 32.9671 | 20.6069 | 49.4267 |
| Uzbekistan | Both | CD | 2019 | 33.5005 | 21.1945 | 49.8594 |
| Grenada | Both | CD | 2019 | 27.0311 | 16.9643 | 40.1409 |
| Japan | Both | CD | 2019 | 9.3380 | 5.8411 | 13.9401 |
| Bulgaria | Both | CD | 2019 | 33.1578 | 21.0513 | 49.7360 |
| Djibouti | Both | CD | 2019 | 24.1023 | 15.1339 | 36.5818 |
| Cambodia | Both | CD | 2019 | 30.9476 | 19.2824 | 46.4589 |
| Greenland | Both | CD | 2019 | 35.8402 | 22.7148 | 53.0661 |
| Sao Tome and Principe | Both | CD | 2019 | 24.4594 | 15.1697 | 37.0576 |
| Lesotho | Both | CD | 2019 | 23.9200 | 15.0374 | 35.8978 |
| Croatia | Both | CD | 2019 | 33.1428 | 21.0622 | 49.0968 |
| Vanuatu | Both | CD | 2019 | 28.2825 | 17.5212 | 42.7405 |
| Germany | Both | CD | 2019 | 13.9792 | 8.7897 | 20.9128 |
| Indonesia | Both | CD | 2019 | 34.9215 | 21.6149 | 51.9338 |
| United Republic of Tanzania | Both | CD | 2019 | 24.2082 | 15.0038 | 36.5455 |
| Eritrea | Both | CD | 2019 | 24.1245 | 15.0680 | 36.7099 |
| Kuwait | Both | CD | 2019 | 26.4130 | 16.3725 | 39.5410 |
| Greece | Both | CD | 2019 | 7.2739 | 4.4898 | 11.2589 |
| Senegal | Both | CD | 2019 | 24.4234 | 15.3036 | 36.6962 |
| American Samoa | Both | CD | 2019 | 28.2627 | 17.5613 | 42.3240 |
| Venezuela (Bolivarian Republic of) | Both | CD | 2019 | 27.1451 | 16.9062 | 40.7849 |
| Guam | Both | CD | 2019 | 28.4454 | 17.7975 | 42.3694 |
| Bhutan | Both | CD | 2019 | 24.7292 | 15.5512 | 37.3042 |
| Uganda | Both | CD | 2019 | 24.1970 | 14.8765 | 36.4786 |
| Viet Nam | Both | CD | 2019 | 31.1366 | 19.5678 | 46.5151 |
| Burkina Faso | Both | CD | 2019 | 24.5377 | 15.4438 | 36.8860 |
| Russian Federation | Both | CD | 2019 | 28.8194 | 18.8657 | 42.3733 |
| Nepal | Both | CD | 2019 | 24.8721 | 15.4667 | 36.8593 |
| India | Both | CD | 2019 | 28.2736 | 17.6081 | 41.9905 |
| Timor-Leste | Both | CD | 2019 | 30.7668 | 19.0503 | 46.3094 |
| Gabon | Both | CD | 2019 | 24.1021 | 15.1319 | 36.6798 |
| South Africa | Both | CD | 2019 | 27.3067 | 17.0842 | 41.1286 |
| Norway | Both | CD | 2019 | 11.2270 | 6.9965 | 16.7783 |
| Singapore | Both | CD | 2019 | 8.1324 | 5.0585 | 12.3174 |
| Ukraine | Both | CD | 2019 | 34.3664 | 21.7586 | 51.2869 |
| Eswatini | Both | CD | 2019 | 23.9441 | 14.9416 | 36.5002 |
| Lebanon | Both | CD | 2019 | 26.6288 | 16.6782 | 39.7757 |
| Serbia | Both | CD | 2019 | 33.1695 | 20.6302 | 49.3768 |
| Netherlands | Both | CD | 2019 | 5.2048 | 3.2148 | 7.9462 |
| Republic of Korea | Both | CD | 2019 | 8.1076 | 5.0311 | 12.1420 |
| Portugal | Both | CD | 2019 | 7.2674 | 4.4960 | 11.1198 |
| Antigua and Barbuda | Both | CD | 2019 | 27.1457 | 17.0674 | 40.6417 |
| Sierra Leone | Both | CD | 2019 | 24.4004 | 15.5455 | 36.9793 |
| Tuvalu | Both | CD | 2019 | 28.3571 | 17.7133 | 42.6184 |
| Ethiopia | Both | CD | 2019 | 27.4656 | 17.0940 | 40.9646 |
| Equatorial Guinea | Both | CD | 2019 | 24.0764 | 14.8530 | 36.4724 |
| Guinea-Bissau | Both | CD | 2019 | 24.4682 | 15.2739 | 36.8496 |
| Honduras | Both | CD | 2019 | 27.1230 | 16.9164 | 40.8277 |
| Brazil | Both | CD | 2019 | 30.7337 | 19.3527 | 45.8308 |
| United Arab Emirates | Both | CD | 2019 | 25.7129 | 16.2953 | 38.5827 |
| Nigeria | Both | CD | 2019 | 28.7257 | 17.9254 | 42.5162 |
| Benin | Both | CD | 2019 | 24.4720 | 15.2148 | 37.0390 |
| Seychelles | Both | CD | 2019 | 30.7747 | 19.2060 | 46.1646 |
| Chad | Both | CD | 2019 | 24.2943 | 15.1905 | 36.9689 |
| Bahamas | Both | CD | 2019 | 27.1716 | 17.2809 | 40.6096 |
| Mauritania | Both | CD | 2019 | 24.5534 | 15.4841 | 37.0662 |
| Mauritius | Both | CD | 2019 | 30.7314 | 19.3670 | 45.9042 |
| Palau | Both | CD | 2019 | 28.1065 | 17.4605 | 42.0377 |
| Yemen | Both | CD | 2019 | 26.5022 | 16.5754 | 39.7147 |
| USA | Both | CD | 2019 | 44.2739 | 28.7410 | 64.7999 |
| Zambia | Both | CD | 2019 | 24.0237 | 14.8978 | 35.6484 |
| Namibia | Both | CD | 2019 | 24.1054 | 15.1712 | 36.0592 |
| Qatar | Both | CD | 2019 | 25.4718 | 16.0435 | 38.0651 |
| Congo | Both | CD | 2019 | 24.0570 | 15.0895 | 36.3641 |
| Mali | Both | CD | 2019 | 24.4401 | 15.1825 | 37.1192 |
| Guatemala | Both | CD | 2019 | 27.0874 | 17.0121 | 40.3236 |
| Libya | Both | CD | 2019 | 26.4402 | 16.7158 | 39.4772 |
| San Marino | Both | CD | 2019 | 7.2906 | 4.6625 | 11.0802 |
| Cyprus | Both | CD | 2019 | 7.2710 | 4.3666 | 11.1253 |
| Albania | Both | CD | 2019 | 33.3626 | 20.9018 | 49.7281 |
| Burundi | Both | CD | 2019 | 23.9798 | 14.7579 | 36.4037 |
| Somalia | Both | CD | 2019 | 24.1334 | 15.0935 | 36.1673 |
| Comoros | Both | CD | 2019 | 24.2395 | 15.0104 | 36.9685 |
| Canada | Both | CD | 2019 | 36.4499 | 22.7135 | 54.3795 |
| Spain | Both | CD | 2019 | 7.2820 | 4.4972 | 11.0280 |
| Colombia | Both | CD | 2019 | 27.2149 | 16.9546 | 40.7718 |
| Paraguay | Both | CD | 2019 | 27.0513 | 16.8672 | 40.4796 |
| Bangladesh | Both | CD | 2019 | 24.8854 | 15.4202 | 36.9390 |
| Oman | Both | CD | 2019 | 26.0039 | 16.3368 | 38.8016 |
| Niue | Both | CD | 2019 | 28.2722 | 17.7594 | 42.6858 |
| Zimbabwe | Both | CD | 2019 | 24.1635 | 14.9548 | 36.7396 |
| Liberia | Both | CD | 2019 | 24.0949 | 14.9889 | 35.9710 |
| Nauru | Both | CD | 2019 | 28.4082 | 17.4997 | 42.7966 |
| Sweden | Both | CD | 2019 | 8.1917 | 5.1214 | 12.3016 |
| Afghanistan | Both | CD | 2019 | 26.1338 | 16.3423 | 38.6805 |
| Haiti | Both | CD | 2019 | 26.9614 | 16.8481 | 40.2710 |
| Rwanda | Both | CD | 2019 | 24.1899 | 14.9445 | 36.5979 |
| Guyana | Both | CD | 2019 | 26.8368 | 17.0658 | 40.4270 |
| Tokelau | Both | CD | 2019 | 28.3597 | 17.6579 | 42.6195 |
| Cameroon | Both | CD | 2019 | 24.3958 | 15.1727 | 37.1888 |
| Finland | Both | CD | 2019 | 7.2463 | 4.5117 | 11.1989 |
| Togo | Both | CD | 2019 | 24.5536 | 15.3766 | 37.1853 |
| Kenya | Both | CD | 2019 | 27.4719 | 17.0906 | 41.4179 |
| Mozambique | Both | CD | 2019 | 24.0379 | 14.9937 | 36.3579 |
| Switzerland | Both | CD | 2019 | 7.2429 | 4.4832 | 11.1031 |
| Denmark | Both | CD | 2019 | 3.1671 | 1.9858 | 4.9095 |
| Costa Rica | Both | CD | 2019 | 27.1725 | 17.2038 | 40.7130 |
| Saudi Arabia | Both | CD | 2019 | 26.0734 | 16.1523 | 38.9155 |
| Niger | Both | CD | 2019 | 24.5031 | 15.2336 | 36.6338 |
| Jamaica | Both | CD | 2019 | 27.0698 | 17.1297 | 40.2206 |
| Central African Republic | Both | CD | 2019 | 24.0447 | 14.9977 | 36.1193 |
| Saint Lucia | Both | CD | 2019 | 26.9393 | 16.8704 | 40.1965 |
| Angola | Both | CD | 2019 | 24.1862 | 15.2501 | 36.8403 |
| United States Virgin Islands | Both | CD | 2019 | 27.1988 | 16.9897 | 40.8321 |
| Botswana | Both | CD | 2019 | 23.9631 | 14.8808 | 36.0717 |
| Northern Mariana Islands | Both | CD | 2019 | 28.3755 | 17.7174 | 42.2578 |
| El Salvador | Both | CD | 2019 | 27.1803 | 16.9059 | 40.8754 |
| Algeria | Both | CD | 2019 | 26.6049 | 16.4940 | 40.0031 |
| Cabo Verde | Both | CD | 2019 | 24.5470 | 15.1885 | 37.1232 |
| Sudan | Both | CD | 2019 | 26.4985 | 16.7835 | 39.7756 |
| Madagascar | Both | CD | 2019 | 24.1385 | 15.0025 | 36.3168 |
| Saint Vincent and the Grenadines | Both | CD | 2019 | 26.9534 | 16.7976 | 40.1877 |
| Ghana | Both | CD | 2019 | 24.5442 | 15.2812 | 36.7255 |
| Guinea | Both | CD | 2019 | 24.4544 | 15.3418 | 37.0562 |
| Democratic Republic of the Congo | Both | CD | 2019 | 24.0281 | 15.1229 | 36.3480 |
| Monaco | Both | CD | 2019 | 7.2691 | 4.5382 | 11.1463 |
| South Sudan | Both | CD | 2019 | 23.8574 | 14.9441 | 35.5313 |
| Morocco | Both | CD | 2019 | 26.5492 | 16.6671 | 40.0829 |
| Palestine | Both | CD | 2019 | 26.4235 | 16.5414 | 39.9329 |
|  |  |  |  |  |  |  |

**Table20 Incidence of SD stratified by sex in 1990~2019, Page 97-99**

| **location_name** | **sex_name** | **cause_name** | **year** | **Age-standardised incidence rate (per 100 000 person-years)** | **95%CI(lower)** | **95%CI(upper)** |
| --- | --- | --- | --- | --- | --- | --- |
| Global | Male | SD | 1990 | 1808.5163 | 1671.4640 | 1949.3779 |
| Global | Female | SD | 1990 | 1809.2371 | 1665.2418 | 1946.7970 |
| Global | Both | SD | 1990 | 1808.8728 | 1668.9329 | 1945.8972 |
| Global | Male | SD | 1992 | 1809.5651 | 1672.2279 | 1950.5085 |
| Global | Female | SD | 1992 | 1806.9260 | 1663.1572 | 1943.9399 |
| Global | Both | SD | 1992 | 1808.2822 | 1668.7724 | 1945.0516 |
| Global | Male | SD | 1994 | 1811.2548 | 1673.6548 | 1952.4376 |
| Global | Female | SD | 1994 | 1805.7760 | 1661.8982 | 1941.9956 |
| Global | Both | SD | 1994 | 1808.6077 | 1669.2657 | 1945.4492 |
| Global | Male | SD | 1991 | 1809.0307 | 1671.8702 | 1949.9139 |
| Global | Female | SD | 1991 | 1808.0549 | 1664.2281 | 1945.3953 |
| Global | Both | SD | 1991 | 1808.5568 | 1668.8618 | 1945.4266 |
| Global | Male | SD | 1995 | 1812.7128 | 1675.0453 | 1954.0779 |
| Global | Female | SD | 1995 | 1806.1669 | 1662.1172 | 1942.1822 |
| Global | Both | SD | 1995 | 1809.5613 | 1670.1317 | 1946.5765 |
| Global | Male | SD | 1993 | 1810.2481 | 1672.7263 | 1951.2750 |
| Global | Female | SD | 1993 | 1806.0558 | 1662.2536 | 1942.6566 |
| Global | Both | SD | 1993 | 1808.2147 | 1668.8382 | 1944.9841 |
| Global | Male | SD | 1998 | 1817.1442 | 1679.1825 | 1959.0643 |
| Global | Female | SD | 1998 | 1808.0410 | 1663.8715 | 1944.0229 |
| Global | Both | SD | 1998 | 1812.8099 | 1672.7567 | 1950.6278 |
| Global | Male | SD | 1996 | 1814.3038 | 1676.4187 | 1955.9498 |
| Global | Female | SD | 1996 | 1806.8364 | 1662.7017 | 1942.6982 |
| Global | Both | SD | 1996 | 1810.7219 | 1671.0994 | 1948.0154 |
| Global | Male | SD | 1997 | 1815.7910 | 1677.8057 | 1957.6313 |
| Global | Female | SD | 1997 | 1807.4628 | 1663.2802 | 1943.3063 |
| Global | Both | SD | 1997 | 1811.8119 | 1672.0203 | 1949.3812 |
| Global | Male | SD | 1999 | 1818.4568 | 1680.6161 | 1960.3775 |
| Global | Female | SD | 1999 | 1808.7497 | 1664.6343 | 1945.0114 |
| Global | Both | SD | 1999 | 1813.8509 | 1673.4900 | 1951.8996 |
| Global | Male | SD | 2000 | 1819.8664 | 1682.1511 | 1961.7593 |
| Global | Female | SD | 2000 | 1809.6896 | 1665.6546 | 1946.2953 |
| Global | Both | SD | 2000 | 1815.0524 | 1674.4238 | 1953.3341 |
| Global | Male | SD | 2001 | 1821.3730 | 1683.8031 | 1963.2320 |
| Global | Female | SD | 2001 | 1810.8549 | 1666.8699 | 1947.8711 |
| Global | Both | SD | 2001 | 1816.4112 | 1675.6118 | 1954.9506 |
| Global | Male | SD | 2002 | 1823.0027 | 1685.5372 | 1965.1044 |
| Global | Female | SD | 2002 | 1812.1141 | 1668.2173 | 1949.6062 |
| Global | Both | SD | 2002 | 1817.8748 | 1677.0003 | 1956.6687 |
| Global | Male | SD | 2003 | 1824.6751 | 1687.2688 | 1966.9952 |
| Global | Female | SD | 2003 | 1813.4639 | 1669.6520 | 1951.4361 |
| Global | Both | SD | 2003 | 1819.3996 | 1678.5144 | 1958.4371 |
| Global | Male | SD | 2006 | 1829.9696 | 1692.3036 | 1972.9494 |
| Global | Female | SD | 2006 | 1818.5910 | 1675.1065 | 1956.3750 |
| Global | Both | SD | 2006 | 1824.6184 | 1683.0604 | 1964.2785 |
| Global | Male | SD | 2004 | 1826.4001 | 1688.9033 | 1968.9568 |
| Global | Female | SD | 2004 | 1815.0087 | 1671.2720 | 1952.8672 |
| Global | Both | SD | 2004 | 1821.0417 | 1680.1142 | 1960.3272 |
| Global | Male | SD | 2005 | 1828.1918 | 1690.5387 | 1970.9521 |
| Global | Female | SD | 2005 | 1816.7300 | 1673.1059 | 1954.2594 |
| Global | Both | SD | 2005 | 1822.8009 | 1681.5714 | 1962.2880 |
| Global | Male | SD | 2007 | 1831.7289 | 1694.2251 | 1974.9444 |
| Global | Female | SD | 2007 | 1820.4310 | 1677.1090 | 1958.3601 |
| Global | Both | SD | 2007 | 1826.4128 | 1684.5064 | 1966.2292 |
| Global | Male | SD | 2009 | 1835.3222 | 1697.3260 | 1978.9492 |
| Global | Female | SD | 2009 | 1824.4857 | 1680.5741 | 1962.3084 |
| Global | Both | SD | 2009 | 1830.2198 | 1687.4584 | 1970.3109 |
| Global | Male | SD | 2010 | 1837.1449 | 1698.8378 | 1981.0875 |
| Global | Female | SD | 2010 | 1826.6214 | 1682.2644 | 1964.7138 |
| Global | Both | SD | 2010 | 1832.1898 | 1688.9756 | 1972.4573 |
| Global | Male | SD | 2008 | 1833.5134 | 1695.8035 | 1976.9435 |
| Global | Female | SD | 2008 | 1822.3743 | 1678.9335 | 1960.1633 |
| Global | Both | SD | 2008 | 1828.2693 | 1685.9541 | 1968.2269 |
| Global | Male | SD | 2012 | 1840.7045 | 1701.8054 | 1985.6256 |
| Global | Female | SD | 2012 | 1830.8664 | 1685.7525 | 1969.4609 |
| Global | Both | SD | 2012 | 1836.0716 | 1692.6354 | 1976.7609 |
| Global | Male | SD | 2011 | 1838.9398 | 1700.3028 | 1983.3665 |
| Global | Female | SD | 2011 | 1828.7601 | 1683.9936 | 1967.1151 |
| Global | Both | SD | 2011 | 1834.1463 | 1690.7794 | 1974.6079 |
| Global | Male | SD | 2013 | 1842.4929 | 1703.3947 | 1987.9193 |
| Global | Female | SD | 2013 | 1832.9461 | 1687.5343 | 1971.3207 |
| Global | Both | SD | 2013 | 1837.9967 | 1694.4654 | 1978.9385 |
| Global | Male | SD | 2014 | 1844.3286 | 1705.0753 | 1990.2577 |
| Global | Female | SD | 2014 | 1835.0634 | 1689.3939 | 1973.2045 |
| Global | Both | SD | 2014 | 1839.9654 | 1696.3236 | 1981.1958 |
| Global | Male | SD | 2015 | 1846.2284 | 1706.8596 | 1992.6547 |
| Global | Female | SD | 2015 | 1837.1888 | 1691.2913 | 1975.1573 |
| Global | Both | SD | 2015 | 1841.9713 | 1698.2059 | 1983.5342 |
| Global | Male | SD | 2017 | 1850.2572 | 1710.7151 | 1997.5740 |
| Global | Female | SD | 2017 | 1841.4896 | 1694.6717 | 1980.1469 |
| Global | Both | SD | 2017 | 1846.1280 | 1702.1388 | 1988.5011 |
| Global | Male | SD | 2016 | 1848.2094 | 1708.7463 | 1995.0995 |
| Global | Female | SD | 2016 | 1839.3276 | 1693.2229 | 1977.4945 |
| Global | Both | SD | 2016 | 1844.0262 | 1700.1466 | 1985.9738 |
| Global | Male | SD | 2019 | 1854.5344 | 1714.9051 | 2002.6224 |
| Global | Female | SD | 2019 | 1845.8525 | 1697.6565 | 1985.5326 |
| Global | Both | SD | 2019 | 1850.4431 | 1706.2477 | 1993.7403 |
| Global | Male | SD | 2018 | 1852.3694 | 1712.7620 | 2000.0809 |
| Global | Female | SD | 2018 | 1843.6629 | 1696.1414 | 1982.8397 |
| Global | Both | SD | 2018 | 1848.2684 | 1704.1809 | 1991.0989 |
|  |  |  |  |  |  |  |

**Table21 Incidence of SD stratified by SDI in 1990~2019, Page 100-110**

| **location_name** | **sex_name** | **cause_name** | **year** | **Age-standardised incidence rate (per 100 000 person-years)** | **95%CI(lower)** | **95%CI(upper)** |
| --- | --- | --- | --- | --- | --- | --- |
| High-middle SDI | Male | SD | 1990 | 1622.4946 | 1500.0818 | 1746.5157 |
| High-middle SDI | Female | SD | 1990 | 1559.8736 | 1435.6816 | 1677.3380 |
| High-middle SDI | Both | SD | 1990 | 1591.4814 | 1469.0632 | 1711.0480 |
| High-middle SDI | Male | SD | 1992 | 1620.4878 | 1497.9950 | 1744.3324 |
| High-middle SDI | Female | SD | 1992 | 1562.8715 | 1438.4874 | 1680.6613 |
| High-middle SDI | Both | SD | 1992 | 1591.9626 | 1468.7045 | 1711.5692 |
| Middle SDI | Male | SD | 1991 | 1602.6245 | 1476.5272 | 1731.6510 |
| Middle SDI | Female | SD | 1991 | 1671.7653 | 1536.2179 | 1800.6103 |
| Middle SDI | Both | SD | 1991 | 1636.9753 | 1508.0483 | 1759.5319 |
| High SDI | Male | SD | 1990 | 1846.4924 | 1705.3308 | 1987.9326 |
| High SDI | Female | SD | 1990 | 2182.9046 | 2005.4739 | 2357.2282 |
| High SDI | Both | SD | 1990 | 2011.7882 | 1852.8128 | 2168.6014 |
| High SDI | Male | SD | 1992 | 1829.7377 | 1690.1054 | 1970.1742 |
| High SDI | Female | SD | 1992 | 2161.8956 | 1987.3148 | 2334.3680 |
| High SDI | Both | SD | 1992 | 1992.7956 | 1835.6883 | 2147.3458 |
| Low-middle SDI | Male | SD | 1992 | 1730.2181 | 1595.0423 | 1870.8489 |
| Low-middle SDI | Female | SD | 1992 | 1801.1985 | 1658.0254 | 1944.6353 |
| Low-middle SDI | Both | SD | 1992 | 1765.1759 | 1629.7265 | 1906.4790 |
| Low-middle SDI | Male | SD | 1990 | 1773.2841 | 1634.9859 | 1918.5724 |
| Low-middle SDI | Female | SD | 1990 | 1799.3423 | 1656.1549 | 1942.7831 |
| Low-middle SDI | Both | SD | 1990 | 1786.1887 | 1649.2612 | 1929.3157 |
| Low SDI | Male | SD | 1990 | 2182.2174 | 2010.3481 | 2365.3859 |
| Low SDI | Female | SD | 1990 | 2197.5170 | 2014.1548 | 2382.2616 |
| Low SDI | Both | SD | 1990 | 2190.1337 | 2005.6770 | 2374.4215 |
| Low SDI | Male | SD | 1992 | 1910.9671 | 1758.9279 | 2071.8587 |
| Low SDI | Female | SD | 1992 | 2195.2095 | 2011.8001 | 2379.7456 |
| Low SDI | Both | SD | 1992 | 2053.0263 | 1879.7518 | 2224.9115 |
| High SDI | Male | SD | 1991 | 1837.8054 | 1697.3985 | 1978.7285 |
| High SDI | Female | SD | 1991 | 2172.0308 | 1996.2075 | 2345.4946 |
| High SDI | Both | SD | 1991 | 2001.9469 | 1844.0251 | 2157.5905 |
| Middle SDI | Male | SD | 1992 | 1623.6593 | 1495.7901 | 1753.9587 |
| Middle SDI | Female | SD | 1992 | 1671.9049 | 1536.3910 | 1800.4431 |
| Middle SDI | Both | SD | 1992 | 1647.7568 | 1518.0901 | 1771.3643 |
| Low-middle SDI | Male | SD | 1991 | 1728.9974 | 1593.9102 | 1869.4894 |
| Low-middle SDI | Female | SD | 1991 | 1800.2835 | 1657.0781 | 1943.6863 |
| Low-middle SDI | Both | SD | 1991 | 1764.1117 | 1628.7558 | 1905.3434 |
| Low SDI | Male | SD | 1991 | 1910.3083 | 1758.5722 | 2071.4938 |
| Low SDI | Female | SD | 1991 | 2197.0540 | 2013.5542 | 2381.8117 |
| Low SDI | Both | SD | 1991 | 2053.7312 | 1880.5013 | 2225.5323 |
| Middle SDI | Male | SD | 1990 | 1815.5282 | 1673.7405 | 1958.8471 |
| Middle SDI | Female | SD | 1990 | 1671.6547 | 1536.2508 | 1800.7019 |
| Middle SDI | Both | SD | 1990 | 1745.1101 | 1607.0420 | 1877.4338 |
| High-middle SDI | Male | SD | 1991 | 1618.3803 | 1495.9515 | 1741.9741 |
| High-middle SDI | Female | SD | 1991 | 1561.3295 | 1436.9937 | 1678.9700 |
| High-middle SDI | Both | SD | 1991 | 1590.1245 | 1467.3314 | 1709.5368 |
| Middle SDI | Male | SD | 1993 | 1612.4746 | 1485.6488 | 1741.9013 |
| Middle SDI | Female | SD | 1993 | 1671.9695 | 1536.7271 | 1800.0669 |
| Middle SDI | Both | SD | 1993 | 1642.1499 | 1512.8466 | 1765.0168 |
| High-middle SDI | Male | SD | 1993 | 1622.5055 | 1499.7713 | 1746.7625 |
| High-middle SDI | Female | SD | 1993 | 1564.2860 | 1439.9421 | 1682.2181 |
| High-middle SDI | Both | SD | 1993 | 1593.6942 | 1470.3518 | 1713.4407 |
| High-middle SDI | Male | SD | 1994 | 1624.5374 | 1501.3782 | 1749.2736 |
| High-middle SDI | Female | SD | 1994 | 1565.7883 | 1441.4526 | 1683.8224 |
| High-middle SDI | Both | SD | 1994 | 1595.4800 | 1472.0781 | 1715.3858 |
| High SDI | Male | SD | 1994 | 1818.6265 | 1680.0834 | 1957.9520 |
| High SDI | Female | SD | 1994 | 2147.3781 | 1975.1449 | 2318.2131 |
| High SDI | Both | SD | 1994 | 1979.9274 | 1824.2251 | 2133.1119 |
| High SDI | Male | SD | 1993 | 1823.0473 | 1684.0456 | 1963.0010 |
| High SDI | Female | SD | 1993 | 2153.3612 | 1979.7904 | 2324.6360 |
| High SDI | Both | SD | 1993 | 1985.1472 | 1828.9360 | 2138.8304 |
| Low SDI | Male | SD | 1995 | 1913.6815 | 1761.3157 | 2074.7629 |
| Low SDI | Female | SD | 1995 | 2195.2091 | 2011.5088 | 2380.0543 |
| Low SDI | Both | SD | 1995 | 2054.2321 | 1880.6353 | 2226.0729 |
| Low-middle SDI | Male | SD | 1994 | 1731.8972 | 1596.7411 | 1872.9231 |
| Low-middle SDI | Female | SD | 1994 | 1802.9797 | 1659.9599 | 1946.7025 |
| Low-middle SDI | Both | SD | 1994 | 1766.9131 | 1631.3976 | 1908.3686 |
| Low-middle SDI | Male | SD | 1993 | 1730.8733 | 1595.7101 | 1871.6469 |
| Low-middle SDI | Female | SD | 1993 | 1802.1039 | 1658.9871 | 1945.6877 |
| Low-middle SDI | Both | SD | 1993 | 1765.9552 | 1630.4676 | 1907.3182 |
| Low SDI | Male | SD | 1994 | 1912.4643 | 1760.1629 | 2073.4900 |
| Low SDI | Female | SD | 1994 | 2194.6626 | 2011.0715 | 2379.3275 |
| Low SDI | Both | SD | 1994 | 2053.3628 | 1879.8830 | 2225.2110 |
| High-middle SDI | Male | SD | 1995 | 1626.6413 | 1502.9029 | 1751.9419 |
| High-middle SDI | Female | SD | 1995 | 1567.5284 | 1443.0547 | 1685.6453 |
| High-middle SDI | Both | SD | 1995 | 1597.4206 | 1473.9414 | 1717.4748 |
| Middle SDI | Male | SD | 1995 | 1638.8532 | 1509.8937 | 1770.1304 |
| Middle SDI | Female | SD | 1995 | 1672.0111 | 1536.7514 | 1799.1743 |
| Middle SDI | Both | SD | 1995 | 1655.6958 | 1525.4613 | 1780.1095 |
| Low SDI | Male | SD | 1993 | 1911.4823 | 1759.2660 | 2072.3143 |
| Low SDI | Female | SD | 1993 | 2194.2527 | 2010.8115 | 2378.7477 |
| Low SDI | Both | SD | 1993 | 2052.6958 | 1879.3633 | 2224.5546 |
| Middle SDI | Male | SD | 1994 | 1633.6947 | 1505.0374 | 1764.6835 |
| Middle SDI | Female | SD | 1994 | 1671.9753 | 1536.7664 | 1799.5501 |
| Middle SDI | Both | SD | 1994 | 1652.9879 | 1522.8989 | 1776.8921 |
| High SDI | Male | SD | 1995 | 1817.2622 | 1678.8785 | 1955.6730 |
| High SDI | Female | SD | 1995 | 2144.7371 | 1973.5538 | 2315.1920 |
| High SDI | Both | SD | 1995 | 1977.9166 | 1822.3748 | 2130.9976 |
| Low-middle SDI | Male | SD | 1995 | 1732.5988 | 1597.5118 | 1873.7918 |
| Low-middle SDI | Female | SD | 1995 | 1803.8469 | 1660.9632 | 1947.7155 |
| Low-middle SDI | Both | SD | 1995 | 1767.7078 | 1632.1957 | 1909.2233 |
| Middle SDI | Male | SD | 1997 | 1632.2943 | 1504.1719 | 1762.9414 |
| Middle SDI | Female | SD | 1997 | 1672.0631 | 1536.7780 | 1798.8544 |
| Middle SDI | Both | SD | 1997 | 1652.5428 | 1522.4526 | 1777.2158 |
| High SDI | Male | SD | 1996 | 1817.6872 | 1679.2071 | 1956.1106 |
| High SDI | Female | SD | 1996 | 2144.0734 | 1972.7739 | 2314.4263 |
| High SDI | Both | SD | 1996 | 1977.7849 | 1822.2669 | 2130.7929 |
| High SDI | Male | SD | 1998 | 1818.3538 | 1679.8516 | 1956.9646 |
| High SDI | Female | SD | 1998 | 2142.7670 | 1971.2021 | 2312.4460 |
| High SDI | Both | SD | 1998 | 1977.4554 | 1822.1024 | 2130.1940 |
| Low-middle SDI | Male | SD | 1996 | 1733.0274 | 1598.0256 | 1874.2623 |
| Low-middle SDI | Female | SD | 1996 | 1804.6644 | 1661.8938 | 1948.7176 |
| Low-middle SDI | Both | SD | 1996 | 1768.3430 | 1632.8440 | 1909.8590 |
| Low SDI | Male | SD | 1997 | 1916.4694 | 1764.0155 | 2077.8964 |
| Low SDI | Female | SD | 1997 | 2195.9714 | 2012.2624 | 2381.1064 |
| Low SDI | Both | SD | 1997 | 2055.9921 | 1882.0237 | 2227.9393 |
| High-middle SDI | Male | SD | 1996 | 1628.4506 | 1504.2716 | 1754.1578 |
| High-middle SDI | Female | SD | 1996 | 1569.1976 | 1444.5701 | 1687.3408 |
| High-middle SDI | Both | SD | 1996 | 1599.1809 | 1475.6487 | 1719.3885 |
| Low-middle SDI | Male | SD | 1998 | 1734.1845 | 1599.3547 | 1875.4177 |
| Low-middle SDI | Female | SD | 1998 | 1806.2414 | 1663.2474 | 1950.7296 |
| Low-middle SDI | Both | SD | 1998 | 1769.7531 | 1634.2196 | 1911.5445 |
| Low SDI | Male | SD | 1996 | 1915.2132 | 1762.7848 | 2076.4369 |
| Low SDI | Female | SD | 1996 | 2195.6628 | 2011.9240 | 2380.7216 |
| Low SDI | Both | SD | 1996 | 2055.2128 | 1881.4110 | 2227.0829 |
| High-middle SDI | Male | SD | 1998 | 1631.8164 | 1507.1725 | 1758.0145 |
| High-middle SDI | Female | SD | 1998 | 1572.5365 | 1447.9609 | 1690.7457 |
| High-middle SDI | Both | SD | 1998 | 1602.5919 | 1478.8727 | 1723.3554 |
| High-middle SDI | Male | SD | 1997 | 1630.1674 | 1505.5223 | 1756.1659 |
| High-middle SDI | Female | SD | 1997 | 1570.9431 | 1446.1403 | 1689.1003 |
| High-middle SDI | Both | SD | 1997 | 1600.9397 | 1477.3498 | 1721.3640 |
| Middle SDI | Male | SD | 1998 | 1636.5379 | 1508.2533 | 1767.2492 |
| Middle SDI | Female | SD | 1998 | 1672.1030 | 1537.0122 | 1798.9110 |
| Middle SDI | Both | SD | 1998 | 1654.7992 | 1524.4782 | 1779.8010 |
| Low SDI | Male | SD | 1998 | 1917.2911 | 1764.8527 | 2078.9520 |
| Low SDI | Female | SD | 1998 | 2196.4867 | 2012.8225 | 2381.5938 |
| Low SDI | Both | SD | 1998 | 2056.6730 | 1882.5791 | 2228.9222 |
| Middle SDI | Male | SD | 1996 | 1627.6903 | 1499.6798 | 1758.2459 |
| Middle SDI | Female | SD | 1996 | 1672.0504 | 1536.7490 | 1798.9585 |
| Middle SDI | Both | SD | 1996 | 1650.1220 | 1520.2416 | 1774.3916 |
| Low-middle SDI | Male | SD | 1997 | 1733.6425 | 1598.7148 | 1874.9001 |
| Low-middle SDI | Female | SD | 1997 | 1805.4385 | 1662.6248 | 1949.6980 |
| Low-middle SDI | Both | SD | 1997 | 1769.0570 | 1633.5468 | 1910.6963 |
| Low SDI | Male | SD | 1999 | 1917.8934 | 1765.4688 | 2079.8278 |
| Low SDI | Female | SD | 1999 | 2197.1565 | 2013.5244 | 2382.1953 |
| Low SDI | Both | SD | 1999 | 2057.3396 | 1883.1741 | 2229.8216 |
| High SDI | Male | SD | 1997 | 1818.0477 | 1679.5346 | 1956.5587 |
| High SDI | Female | SD | 1997 | 2143.4254 | 1972.0144 | 2313.4489 |
| High SDI | Both | SD | 1997 | 1977.6369 | 1822.1866 | 2130.5123 |
| High-middle SDI | Male | SD | 2000 | 1635.0289 | 1510.4904 | 1761.2869 |
| High-middle SDI | Female | SD | 2000 | 1575.8714 | 1451.2949 | 1694.1875 |
| High-middle SDI | Both | SD | 2000 | 1605.9328 | 1481.8488 | 1727.3642 |
| Middle SDI | Male | SD | 1999 | 1640.6133 | 1512.1844 | 1771.7345 |
| Middle SDI | Female | SD | 1999 | 1672.2563 | 1537.2565 | 1799.1985 |
| Middle SDI | Both | SD | 1999 | 1657.0286 | 1526.4262 | 1782.3111 |
| High SDI | Male | SD | 2000 | 1819.0075 | 1680.5599 | 1957.1413 |
| High SDI | Female | SD | 2000 | 2141.4164 | 1969.5597 | 2310.5263 |
| High SDI | Both | SD | 2000 | 1977.1236 | 1821.9568 | 2129.5807 |
| Low-middle SDI | Male | SD | 2000 | 1735.1510 | 1600.5405 | 1876.2732 |
| Low-middle SDI | Female | SD | 2000 | 1808.0325 | 1664.6895 | 1952.9934 |
| Low-middle SDI | Both | SD | 2000 | 1771.1809 | 1635.5713 | 1913.2408 |
| High-middle SDI | Male | SD | 1999 | 1633.3523 | 1508.9810 | 1759.5963 |
| High-middle SDI | Female | SD | 1999 | 1574.0964 | 1449.5821 | 1692.3546 |
| High-middle SDI | Both | SD | 1999 | 1604.1728 | 1480.2782 | 1725.2713 |
| Low SDI | Male | SD | 2000 | 1918.4698 | 1766.0203 | 2080.6594 |
| Low SDI | Female | SD | 2000 | 2197.5354 | 2014.0441 | 2382.4868 |
| Low SDI | Both | SD | 2000 | 2057.8450 | 1883.6499 | 2230.5306 |
| Middle SDI | Male | SD | 2000 | 1644.4264 | 1515.8100 | 1775.7994 |
| Middle SDI | Female | SD | 2000 | 1672.4999 | 1537.4872 | 1799.6652 |
| Middle SDI | Both | SD | 2000 | 1659.1658 | 1528.3282 | 1784.6188 |
| High SDI | Male | SD | 1999 | 1818.6817 | 1680.1996 | 1956.9630 |
| High SDI | Female | SD | 1999 | 2142.0986 | 1970.3864 | 2311.4681 |
| High SDI | Both | SD | 1999 | 1977.2982 | 1822.0359 | 2129.9009 |
| Low-middle SDI | Male | SD | 1999 | 1734.9174 | 1600.1617 | 1876.1229 |
| Low-middle SDI | Female | SD | 1999 | 1807.0907 | 1663.9208 | 1951.8182 |
| Low-middle SDI | Both | SD | 1999 | 1770.5710 | 1634.9824 | 1912.5198 |
| Low SDI | Male | SD | 2001 | 1919.2423 | 1766.7664 | 2081.6754 |
| Low SDI | Female | SD | 2001 | 2197.5184 | 2014.2245 | 2382.2869 |
| Low SDI | Both | SD | 2001 | 2058.2353 | 1884.0720 | 2231.1547 |
| High SDI | Male | SD | 2002 | 1819.6395 | 1681.3734 | 1957.7565 |
| High SDI | Female | SD | 2002 | 2140.1069 | 1968.0097 | 2308.8701 |
| High SDI | Both | SD | 2002 | 1976.7788 | 1821.8513 | 2129.1532 |
| High-middle SDI | Male | SD | 2002 | 1638.8137 | 1514.1673 | 1765.0093 |
| High-middle SDI | Female | SD | 2002 | 1579.8512 | 1454.3855 | 1698.0144 |
| High-middle SDI | Both | SD | 2002 | 1609.8925 | 1485.3547 | 1731.8670 |
| Low-middle SDI | Male | SD | 2002 | 1736.2781 | 1601.9863 | 1877.2410 |
| Low-middle SDI | Female | SD | 2002 | 1810.3784 | 1666.6329 | 1955.8614 |
| Low-middle SDI | Both | SD | 2002 | 1772.9568 | 1637.3014 | 1915.2893 |
| Low SDI | Male | SD | 2002 | 1920.0576 | 1767.5077 | 2082.7605 |
| Low SDI | Female | SD | 2002 | 2197.0805 | 2014.0358 | 2381.6199 |
| Low SDI | Both | SD | 2002 | 2058.4230 | 1884.3757 | 2231.5470 |
| Low-middle SDI | Male | SD | 2001 | 1735.5907 | 1601.1409 | 1876.6198 |
| Low-middle SDI | Female | SD | 2001 | 1809.1268 | 1665.5873 | 1954.3452 |
| Low-middle SDI | Both | SD | 2001 | 1771.9683 | 1636.3489 | 1914.1554 |
| Middle SDI | Male | SD | 2001 | 1647.9082 | 1519.1283 | 1779.6845 |
| Middle SDI | Female | SD | 2001 | 1672.7928 | 1537.6557 | 1800.2861 |
| Middle SDI | Both | SD | 2001 | 1661.1466 | 1530.1595 | 1786.7281 |
| High-middle SDI | Male | SD | 2001 | 1636.8075 | 1512.2282 | 1763.0362 |
| High-middle SDI | Female | SD | 2001 | 1577.7817 | 1452.8624 | 1696.0564 |
| High-middle SDI | Both | SD | 2001 | 1607.8150 | 1483.4961 | 1729.5276 |
| Middle SDI | Male | SD | 2002 | 1667.2777 | 1536.9605 | 1800.6239 |
| Middle SDI | Female | SD | 2002 | 1673.1159 | 1537.7690 | 1801.0261 |
| Middle SDI | Both | SD | 2002 | 1671.1580 | 1539.5602 | 1797.4261 |
| High SDI | Male | SD | 2001 | 1819.3326 | 1681.0121 | 1957.4857 |
| High SDI | Female | SD | 2001 | 2140.7793 | 1968.7692 | 2309.6515 |
| High SDI | Both | SD | 2001 | 1976.9625 | 1821.9143 | 2129.2820 |
| Low SDI | Male | SD | 2003 | 1920.6881 | 1768.0632 | 2083.3370 |
| Low SDI | Female | SD | 2003 | 2197.0147 | 2014.1420 | 2381.5202 |
| Low SDI | Both | SD | 2003 | 2058.7174 | 1884.7946 | 2232.0091 |
| Middle SDI | Male | SD | 2003 | 1670.2796 | 1539.8135 | 1803.7246 |
| Middle SDI | Female | SD | 2003 | 1673.4313 | 1538.0951 | 1801.8194 |
| Middle SDI | Both | SD | 2003 | 1672.8841 | 1541.2728 | 1799.4141 |
| High-middle SDI | Male | SD | 2004 | 1643.0408 | 1518.3901 | 1769.2286 |
| High-middle SDI | Female | SD | 2004 | 1583.8228 | 1456.9184 | 1702.1114 |
| High-middle SDI | Both | SD | 2004 | 1614.0676 | 1489.0797 | 1736.5721 |
| High SDI | Male | SD | 2004 | 1820.2672 | 1682.1417 | 1958.6589 |
| High SDI | Female | SD | 2004 | 2138.5857 | 1966.7233 | 2307.2174 |
| High SDI | Both | SD | 2004 | 1976.3129 | 1821.6590 | 2129.0401 |
| High-middle SDI | Male | SD | 2003 | 1640.9067 | 1516.2462 | 1767.0675 |
| High-middle SDI | Female | SD | 2003 | 1581.8435 | 1455.6742 | 1700.1307 |
| High-middle SDI | Both | SD | 2003 | 1611.9744 | 1487.2224 | 1734.2274 |
| Low-middle SDI | Male | SD | 2004 | 1737.1632 | 1603.2314 | 1877.9902 |
| Low-middle SDI | Female | SD | 2004 | 1813.1312 | 1668.9549 | 1959.1437 |
| Low-middle SDI | Both | SD | 2004 | 1774.8022 | 1638.7714 | 1917.3381 |
| High SDI | Male | SD | 2003 | 1819.9711 | 1681.7905 | 1958.2056 |
| High SDI | Female | SD | 2003 | 2139.3900 | 1967.4013 | 2308.0603 |
| High SDI | Both | SD | 2003 | 1976.5839 | 1821.7992 | 2129.1279 |
| Low-middle SDI | Male | SD | 2003 | 1736.7204 | 1602.6069 | 1877.6109 |
| Low-middle SDI | Female | SD | 2003 | 1811.7234 | 1667.7625 | 1957.4743 |
| Low-middle SDI | Both | SD | 2003 | 1773.8647 | 1638.0218 | 1916.3425 |
| Low SDI | Male | SD | 2004 | 1921.2362 | 1768.5454 | 2083.5719 |
| Low SDI | Female | SD | 2004 | 2197.1109 | 2014.3884 | 2381.4793 |
| Low SDI | Both | SD | 2004 | 2059.0596 | 1885.2660 | 2232.3068 |
| Middle SDI | Male | SD | 2004 | 1673.1841 | 1542.5666 | 1806.7410 |
| Middle SDI | Female | SD | 2004 | 1673.7966 | 1538.4397 | 1802.6815 |
| Middle SDI | Both | SD | 2004 | 1674.5815 | 1543.0084 | 1801.5708 |
| High SDI | Male | SD | 2006 | 1820.5568 | 1682.4537 | 1958.8374 |
| High SDI | Female | SD | 2006 | 2136.3786 | 1964.8629 | 2305.0604 |
| High SDI | Both | SD | 2006 | 1975.2113 | 1820.9869 | 2127.9387 |
| High-middle SDI | Male | SD | 2006 | 1647.2662 | 1521.9585 | 1773.5980 |
| High-middle SDI | Female | SD | 2006 | 1587.6987 | 1459.8751 | 1706.1147 |
| High-middle SDI | Both | SD | 2006 | 1618.1904 | 1492.7827 | 1741.1348 |
| Middle SDI | Male | SD | 2005 | 1659.6984 | 1530.0631 | 1792.1498 |
| Middle SDI | Female | SD | 2005 | 1674.2396 | 1538.8375 | 1803.5670 |
| Middle SDI | Both | SD | 2005 | 1668.0469 | 1536.9898 | 1794.5824 |
| Low-middle SDI | Male | SD | 2005 | 1737.6087 | 1603.8269 | 1878.4062 |
| Low-middle SDI | Female | SD | 2005 | 1814.6215 | 1670.2141 | 1960.9162 |
| Low-middle SDI | Both | SD | 2005 | 1775.7804 | 1639.7337 | 1918.3756 |
| Low SDI | Male | SD | 2005 | 1921.9168 | 1769.1575 | 2084.0234 |
| Low SDI | Female | SD | 2005 | 2197.2095 | 2014.6538 | 2381.4971 |
| Low SDI | Both | SD | 2005 | 2059.4719 | 1885.7160 | 2232.7258 |
| Low-middle SDI | Male | SD | 2006 | 1738.0743 | 1604.4181 | 1878.8740 |
| Low-middle SDI | Female | SD | 2006 | 1816.2458 | 1671.5864 | 1962.8588 |
| Low-middle SDI | Both | SD | 2006 | 1776.8328 | 1640.7519 | 1919.5419 |
| Low SDI | Male | SD | 2006 | 1922.7672 | 1769.9215 | 2084.8134 |
| Low SDI | Female | SD | 2006 | 2197.8995 | 2015.3556 | 2382.2325 |
| Low SDI | Both | SD | 2006 | 2060.2788 | 1886.5698 | 2233.6069 |
| High-middle SDI | Male | SD | 2005 | 1645.2738 | 1520.4711 | 1771.5222 |
| High-middle SDI | Female | SD | 2005 | 1585.8163 | 1458.2479 | 1704.1725 |
| High-middle SDI | Both | SD | 2005 | 1616.2187 | 1491.0135 | 1738.9462 |
| High SDI | Male | SD | 2005 | 1820.4995 | 1682.3948 | 1958.9941 |
| High SDI | Female | SD | 2005 | 2137.6217 | 1965.8729 | 2306.2400 |
| High SDI | Both | SD | 2005 | 1975.9161 | 1821.4289 | 2128.8096 |
| Middle SDI | Male | SD | 2006 | 1662.3448 | 1532.2625 | 1795.0089 |
| Middle SDI | Female | SD | 2006 | 1674.6373 | 1539.1731 | 1804.2879 |
| Middle SDI | Both | SD | 2006 | 1669.6225 | 1538.5492 | 1796.5167 |
| Low-middle SDI | Male | SD | 2007 | 1738.5608 | 1605.0043 | 1879.4202 |
| Low-middle SDI | Female | SD | 2007 | 1817.9735 | 1673.0318 | 1964.9132 |
| Low-middle SDI | Both | SD | 2007 | 1777.9450 | 1641.7942 | 1920.8306 |
| Low SDI | Male | SD | 2007 | 1923.6498 | 1770.7016 | 2085.7679 |
| Low SDI | Female | SD | 2007 | 2198.4318 | 2015.8493 | 2382.8107 |
| Low SDI | Both | SD | 2007 | 2061.0277 | 1887.3302 | 2234.4800 |
| High-middle SDI | Male | SD | 2008 | 1650.7450 | 1524.8551 | 1777.2648 |
| High-middle SDI | Female | SD | 2008 | 1591.3467 | 1463.1203 | 1710.2874 |
| High-middle SDI | Both | SD | 2008 | 1621.8101 | 1496.1238 | 1745.0058 |
| Middle SDI | Male | SD | 2007 | 1681.0433 | 1549.4077 | 1815.2635 |
| Middle SDI | Female | SD | 2007 | 1674.9782 | 1539.4275 | 1804.8169 |
| Middle SDI | Both | SD | 2007 | 1679.2566 | 1547.6516 | 1807.3280 |
| High SDI | Male | SD | 2008 | 1819.7779 | 1681.6112 | 1957.6807 |
| High SDI | Female | SD | 2008 | 2133.0458 | 1961.8108 | 2301.4389 |
| High SDI | Both | SD | 2008 | 1972.6158 | 1818.8594 | 2124.8094 |
| Low-middle SDI | Male | SD | 2009 | 1739.5707 | 1606.1090 | 1880.6843 |
| Low-middle SDI | Female | SD | 2009 | 1821.7543 | 1676.1516 | 1969.3659 |
| Low-middle SDI | Both | SD | 2009 | 1780.3464 | 1643.9897 | 1923.6222 |
| Low-middle SDI | Male | SD | 2008 | 1739.0623 | 1605.5711 | 1880.0267 |
| Low-middle SDI | Female | SD | 2008 | 1819.8083 | 1674.5511 | 1967.0821 |
| Low-middle SDI | Both | SD | 2008 | 1779.1171 | 1642.8689 | 1922.2376 |
| High-middle SDI | Male | SD | 2007 | 1649.0913 | 1523.4677 | 1775.5098 |
| High-middle SDI | Female | SD | 2007 | 1589.5691 | 1461.5233 | 1708.1893 |
| High-middle SDI | Both | SD | 2007 | 1620.0675 | 1494.4996 | 1743.1687 |
| Low SDI | Male | SD | 2009 | 1967.3938 | 1810.8584 | 2134.0443 |
| Low SDI | Female | SD | 2009 | 2199.5384 | 2016.8115 | 2384.0593 |
| Low SDI | Both | SD | 2009 | 2083.5881 | 1907.6288 | 2259.4104 |
| Low SDI | Male | SD | 2008 | 1966.1020 | 1809.7062 | 2132.5898 |
| Low SDI | Female | SD | 2008 | 2198.9220 | 2016.2752 | 2383.3582 |
| Low SDI | Both | SD | 2008 | 2082.5876 | 1906.6894 | 2258.2034 |
| Middle SDI | Male | SD | 2009 | 1689.2498 | 1557.3656 | 1824.6320 |
| Middle SDI | Female | SD | 2009 | 1675.6999 | 1540.0732 | 1805.6372 |
| Middle SDI | Both | SD | 2009 | 1683.8568 | 1552.1262 | 1812.6111 |
| High SDI | Male | SD | 2007 | 1820.3185 | 1682.2311 | 1958.4773 |
| High SDI | Female | SD | 2007 | 2134.7804 | 1963.4106 | 2303.4373 |
| High SDI | Both | SD | 2007 | 1974.0743 | 1820.0738 | 2126.5317 |
| High SDI | Male | SD | 2009 | 1819.1288 | 1680.8668 | 1956.7133 |
| High SDI | Female | SD | 2009 | 2131.5660 | 1960.4797 | 2299.6070 |
| High SDI | Both | SD | 2009 | 1971.2766 | 1817.7336 | 2123.2518 |
| Middle SDI | Male | SD | 2008 | 1683.4429 | 1551.5816 | 1818.2958 |
| Middle SDI | Female | SD | 2008 | 1675.3268 | 1539.6502 | 1805.2468 |
| Middle SDI | Both | SD | 2008 | 1680.6835 | 1549.0598 | 1809.0395 |
| High-middle SDI | Male | SD | 2009 | 1652.1184 | 1526.4803 | 1778.7903 |
| High-middle SDI | Female | SD | 2009 | 1592.9918 | 1464.6802 | 1712.3331 |
| High-middle SDI | Both | SD | 2009 | 1623.3420 | 1497.6039 | 1746.6195 |
| High-middle SDI | Male | SD | 2010 | 1653.2600 | 1528.0465 | 1780.1528 |
| High-middle SDI | Female | SD | 2010 | 1594.4407 | 1466.1360 | 1713.7399 |
| High-middle SDI | Both | SD | 2010 | 1624.6592 | 1498.9218 | 1747.9912 |
| High SDI | Male | SD | 2010 | 1818.6519 | 1680.2225 | 1955.9587 |
| High SDI | Female | SD | 2010 | 2130.5343 | 1959.5850 | 2298.3710 |
| High SDI | Both | SD | 2010 | 1970.4198 | 1817.0685 | 2122.2903 |
| Low-middle SDI | Male | SD | 2010 | 1740.1067 | 1606.6457 | 1881.4073 |
| Low-middle SDI | Female | SD | 2010 | 1823.7788 | 1677.8137 | 1971.7159 |
| Low-middle SDI | Both | SD | 2010 | 1781.6263 | 1645.1611 | 1925.0287 |
| Low-middle SDI | Male | SD | 2011 | 1740.7103 | 1607.2120 | 1882.2434 |
| Low-middle SDI | Female | SD | 2011 | 1825.8666 | 1679.7940 | 1974.1082 |
| Low-middle SDI | Both | SD | 2011 | 1782.9705 | 1646.3988 | 1926.5071 |
| Low SDI | Male | SD | 2010 | 1968.6718 | 1812.0262 | 2135.4454 |
| Low SDI | Female | SD | 2010 | 2200.0347 | 2017.2423 | 2384.6405 |
| Low SDI | Both | SD | 2010 | 2084.5276 | 1908.5309 | 2260.5355 |
| High-middle SDI | Male | SD | 2011 | 1654.1670 | 1529.3784 | 1781.3451 |
| High-middle SDI | Female | SD | 2011 | 1595.4807 | 1467.5184 | 1714.7562 |
| High-middle SDI | Both | SD | 2011 | 1625.6560 | 1499.9674 | 1749.0248 |
| Low SDI | Male | SD | 2011 | 1969.9033 | 1813.1876 | 2136.7737 |
| Low SDI | Female | SD | 2011 | 2200.6141 | 2017.7402 | 2385.2164 |
| Low SDI | Both | SD | 2011 | 2085.4869 | 1909.3673 | 2261.6806 |
| Middle SDI | Male | SD | 2010 | 1687.9639 | 1556.4788 | 1823.4359 |
| Middle SDI | Female | SD | 2010 | 1676.0643 | 1540.5446 | 1805.9412 |
| Middle SDI | Both | SD | 2010 | 1683.4303 | 1551.7771 | 1812.1562 |
| Middle SDI | Male | SD | 2011 | 1693.5362 | 1562.0744 | 1829.5670 |
| Middle SDI | Female | SD | 2011 | 1676.4807 | 1541.0546 | 1806.2190 |
| Middle SDI | Both | SD | 2011 | 1686.5026 | 1554.6190 | 1815.5950 |
| High SDI | Male | SD | 2011 | 1818.3000 | 1679.7122 | 1955.3252 |
| High SDI | Female | SD | 2011 | 2129.6953 | 1958.7882 | 2297.4443 |
| High SDI | Both | SD | 2011 | 1969.7304 | 1816.6110 | 2121.5143 |
| High SDI | Male | SD | 2013 | 1817.8823 | 1679.0526 | 1954.8193 |
| High SDI | Female | SD | 2013 | 2128.3533 | 1957.5940 | 2296.0133 |
| High SDI | Both | SD | 2013 | 1968.6489 | 1815.9346 | 2120.4480 |
| Low-middle SDI | Male | SD | 2013 | 1742.1252 | 1608.3668 | 1883.6634 |
| Low-middle SDI | Female | SD | 2013 | 1830.0796 | 1683.8021 | 1978.8333 |
| Low-middle SDI | Both | SD | 2013 | 1785.7799 | 1649.0584 | 1929.5765 |
| Low SDI | Male | SD | 2013 | 1972.0177 | 1815.2755 | 2139.3339 |
| Low SDI | Female | SD | 2013 | 2201.7410 | 2018.7208 | 2386.2372 |
| Low SDI | Both | SD | 2013 | 2087.2142 | 1910.8568 | 2263.6717 |
| High-middle SDI | Male | SD | 2012 | 1654.8592 | 1529.9039 | 1782.3609 |
| High-middle SDI | Female | SD | 2012 | 1596.0763 | 1468.5977 | 1715.3692 |
| High-middle SDI | Both | SD | 2012 | 1626.3264 | 1500.7008 | 1749.6650 |
| Middle SDI | Male | SD | 2013 | 1693.6984 | 1562.7534 | 1830.2498 |
| Middle SDI | Female | SD | 2013 | 1677.5603 | 1542.2952 | 1807.5353 |
| Middle SDI | Both | SD | 2013 | 1687.1921 | 1554.9906 | 1816.4547 |
| High SDI | Male | SD | 2012 | 1818.0490 | 1679.3307 | 1954.9193 |
| High SDI | Female | SD | 2012 | 2128.9830 | 1958.1689 | 2296.6635 |
| High SDI | Both | SD | 2012 | 1969.1491 | 1816.2249 | 2120.9146 |
| Low-middle SDI | Male | SD | 2012 | 1741.3838 | 1607.7853 | 1882.9848 |
| Low-middle SDI | Female | SD | 2012 | 1827.9705 | 1681.7965 | 1976.5138 |
| Low-middle SDI | Both | SD | 2012 | 1784.3575 | 1647.6990 | 1928.0276 |
| Low SDI | Male | SD | 2012 | 1971.0161 | 1814.2716 | 2138.0185 |
| Low SDI | Female | SD | 2012 | 2201.1575 | 2018.2097 | 2385.7226 |
| Low SDI | Both | SD | 2012 | 2086.3665 | 1910.1281 | 2262.7155 |
| High-middle SDI | Male | SD | 2013 | 1655.3149 | 1529.8985 | 1783.1594 |
| High-middle SDI | Female | SD | 2013 | 1596.4184 | 1469.4428 | 1715.6349 |
| High-middle SDI | Both | SD | 2013 | 1626.7494 | 1501.1716 | 1750.3235 |
| Middle SDI | Male | SD | 2012 | 1691.9446 | 1560.8802 | 1828.1110 |
| Middle SDI | Female | SD | 2012 | 1677.0088 | 1541.6655 | 1806.7762 |
| Middle SDI | Both | SD | 2012 | 1685.9932 | 1554.0325 | 1815.0592 |
| High SDI | Male | SD | 2015 | 1817.7332 | 1678.7457 | 1954.7656 |
| High SDI | Female | SD | 2015 | 2127.3124 | 1956.7377 | 2294.6209 |
| High SDI | Both | SD | 2015 | 1967.8733 | 1815.5147 | 2119.4855 |
| Low-middle SDI | Male | SD | 2015 | 1743.7820 | 1609.5900 | 1885.3278 |
| Low-middle SDI | Female | SD | 2015 | 1834.2856 | 1687.8326 | 1982.9689 |
| Low-middle SDI | Both | SD | 2015 | 1788.7003 | 1651.8916 | 1932.7256 |
| High-middle SDI | Male | SD | 2014 | 1655.4544 | 1529.6587 | 1783.6201 |
| High-middle SDI | Female | SD | 2014 | 1596.7436 | 1470.1466 | 1715.8524 |
| High-middle SDI | Both | SD | 2014 | 1626.9966 | 1501.4560 | 1750.7297 |
| Low SDI | Male | SD | 2015 | 1973.7170 | 1816.8881 | 2141.6349 |
| Low SDI | Female | SD | 2015 | 2203.4044 | 2020.2058 | 2387.8349 |
| Low SDI | Both | SD | 2015 | 2089.0119 | 1912.4571 | 2265.6521 |
| High-middle SDI | Male | SD | 2015 | 1655.3031 | 1529.2288 | 1783.7739 |
| High-middle SDI | Female | SD | 2015 | 1597.1086 | 1470.7897 | 1716.0794 |
| High-middle SDI | Both | SD | 2015 | 1627.1101 | 1501.5227 | 1750.8277 |
| High SDI | Male | SD | 2014 | 1817.8111 | 1678.8990 | 1954.7552 |
| High SDI | Female | SD | 2014 | 2127.8212 | 1957.1281 | 2295.3741 |
| High SDI | Both | SD | 2014 | 1968.2528 | 1815.7249 | 2120.0496 |
| High SDI | Male | SD | 2017 | 1817.6002 | 1678.6110 | 1954.8035 |
| High SDI | Female | SD | 2017 | 2126.5526 | 1956.1854 | 2293.5095 |
| High SDI | Both | SD | 2017 | 1967.2760 | 1815.1560 | 2118.6390 |
| Middle SDI | Male | SD | 2015 | 1697.4505 | 1566.5751 | 1834.0223 |
| Middle SDI | Female | SD | 2015 | 1678.4743 | 1543.4214 | 1808.3686 |
| Middle SDI | Both | SD | 2015 | 1689.6218 | 1557.2491 | 1819.1920 |
| Low-middle SDI | Male | SD | 2017 | 1745.5501 | 1610.9321 | 1887.1373 |
| Low-middle SDI | Female | SD | 2017 | 1838.6045 | 1692.1703 | 1987.3948 |
| Low-middle SDI | Both | SD | 2017 | 1791.7413 | 1654.8264 | 1935.8789 |
| High SDI | Male | SD | 2016 | 1817.6720 | 1678.6682 | 1954.7851 |
| High SDI | Female | SD | 2016 | 2126.8840 | 1956.4486 | 2293.9947 |
| High SDI | Both | SD | 2016 | 1967.5468 | 1815.3684 | 2119.0222 |
| Low-middle SDI | Male | SD | 2014 | 1742.9230 | 1608.9607 | 1884.4473 |
| Low-middle SDI | Female | SD | 2014 | 1832.1938 | 1685.8171 | 1980.8945 |
| Low-middle SDI | Both | SD | 2014 | 1787.2309 | 1650.4632 | 1931.1509 |
| Low SDI | Male | SD | 2017 | 1975.8002 | 1818.6782 | 2144.1018 |
| Low SDI | Female | SD | 2017 | 2205.0275 | 2021.7590 | 2389.3765 |
| Low SDI | Both | SD | 2017 | 2090.9392 | 1914.3118 | 2267.7218 |
| Low-middle SDI | Male | SD | 2016 | 1744.6722 | 1610.2521 | 1886.2364 |
| Low-middle SDI | Female | SD | 2016 | 1836.4083 | 1689.9404 | 1985.1215 |
| Low-middle SDI | Both | SD | 2016 | 1790.2043 | 1653.3441 | 1934.2951 |
| Low SDI | Male | SD | 2014 | 1972.9111 | 1816.1986 | 2140.5342 |
| Low SDI | Female | SD | 2014 | 2202.5487 | 2019.4374 | 2387.0040 |
| Low SDI | Both | SD | 2014 | 2088.1241 | 1911.6502 | 2264.6832 |
| Middle SDI | Male | SD | 2017 | 1704.8676 | 1573.4678 | 1841.4651 |
| Middle SDI | Female | SD | 2017 | 1679.0707 | 1544.3992 | 1808.8430 |
| Middle SDI | Both | SD | 2017 | 1693.7393 | 1561.0932 | 1823.6712 |
| Low SDI | Male | SD | 2016 | 1974.6177 | 1817.6403 | 2142.7719 |
| Low SDI | Female | SD | 2016 | 2204.1531 | 2020.9248 | 2388.5300 |
| Low SDI | Both | SD | 2016 | 2089.8809 | 1913.2830 | 2266.5794 |
| Middle SDI | Male | SD | 2014 | 1695.5057 | 1564.6323 | 1832.3948 |
| Middle SDI | Female | SD | 2014 | 1678.0579 | 1542.8790 | 1808.0858 |
| Middle SDI | Both | SD | 2014 | 1688.3924 | 1555.9450 | 1817.8564 |
| Middle SDI | Male | SD | 2016 | 1699.4388 | 1568.4056 | 1835.6630 |
| Middle SDI | Female | SD | 2016 | 1678.8207 | 1543.9426 | 1808.6369 |
| Middle SDI | Both | SD | 2016 | 1690.8332 | 1558.6231 | 1820.5024 |
| High-middle SDI | Male | SD | 2016 | 1654.8829 | 1528.6799 | 1783.6265 |
| High-middle SDI | Female | SD | 2016 | 1597.4833 | 1471.3653 | 1716.3362 |
| High-middle SDI | Both | SD | 2016 | 1627.0881 | 1501.7218 | 1750.8507 |
| High-middle SDI | Male | SD | 2017 | 1654.1205 | 1527.9890 | 1783.1146 |
| High-middle SDI | Female | SD | 2017 | 1597.8262 | 1471.8372 | 1716.7753 |
| High-middle SDI | Both | SD | 2017 | 1626.8705 | 1501.6689 | 1750.7301 |
| High-middle SDI | Male | SD | 2018 | 1653.1049 | 1527.2002 | 1782.3403 |
| High-middle SDI | Female | SD | 2018 | 1598.0935 | 1472.2141 | 1717.1262 |
| High-middle SDI | Both | SD | 2018 | 1626.4804 | 1501.3864 | 1750.4932 |
| Low-middle SDI | Male | SD | 2019 | 1747.4078 | 1612.4401 | 1889.1749 |
| Low-middle SDI | Female | SD | 2019 | 1842.8992 | 1696.5472 | 1991.8582 |
| Low-middle SDI | Both | SD | 2019 | 1794.8035 | 1657.8558 | 1938.7151 |
| High SDI | Male | SD | 2018 | 1817.5431 | 1678.5924 | 1954.8287 |
| High SDI | Female | SD | 2018 | 2126.2868 | 1956.1319 | 2293.1287 |
| High SDI | Both | SD | 2018 | 1967.0508 | 1814.9584 | 2118.3921 |
| Low-middle SDI | Male | SD | 2018 | 1746.4566 | 1611.6570 | 1888.1080 |
| Low-middle SDI | Female | SD | 2018 | 1840.7722 | 1694.4059 | 1989.6453 |
| Low-middle SDI | Both | SD | 2018 | 1793.2732 | 1656.3356 | 1937.4087 |
| High SDI | Male | SD | 2019 | 1817.5285 | 1678.6262 | 1954.8855 |
| High SDI | Female | SD | 2019 | 2126.0645 | 1956.1540 | 2292.8086 |
| High SDI | Both | SD | 2019 | 1966.8788 | 1814.8731 | 2118.0944 |
| Low SDI | Male | SD | 2019 | 1978.3801 | 1820.9936 | 2146.8748 |
| Low SDI | Female | SD | 2019 | 2207.1046 | 2023.7570 | 2391.4746 |
| Low SDI | Both | SD | 2019 | 2093.3100 | 1916.5147 | 2270.2560 |
| Middle SDI | Male | SD | 2019 | 1708.6721 | 1577.2521 | 1845.8997 |
| Middle SDI | Female | SD | 2019 | 1679.3751 | 1545.1900 | 1809.1533 |
| Middle SDI | Both | SD | 2019 | 1695.8649 | 1563.1093 | 1825.9285 |
| Low SDI | Male | SD | 2018 | 1977.1005 | 1819.8401 | 2145.5035 |
| Low SDI | Female | SD | 2018 | 2206.0463 | 2022.7331 | 2390.4008 |
| Low SDI | Both | SD | 2018 | 2092.1223 | 1915.4802 | 2268.9969 |
| High-middle SDI | Male | SD | 2019 | 1651.9318 | 1526.2844 | 1781.3818 |
| High-middle SDI | Female | SD | 2019 | 1598.2933 | 1472.4693 | 1717.3891 |
| High-middle SDI | Both | SD | 2019 | 1625.9720 | 1500.9530 | 1749.8647 |
| Middle SDI | Male | SD | 2018 | 1706.7557 | 1575.3273 | 1843.6357 |
| Middle SDI | Female | SD | 2018 | 1679.2378 | 1544.8002 | 1808.9957 |
| Middle SDI | Both | SD | 2018 | 1694.8044 | 1561.9335 | 1824.7915 |
|  |  |  |  |  |  |  |

**Table22 DALYs of SD stratified by sex in 1990~2019, Page 111-113**

| **location_name** | **sex_name** | **cause_name** | **year** | **Age-standardised DALYs rate (per 100 000 person-years)** | **95%CI(lower)** | **95%CI(upper)** |
| --- | --- | --- | --- | --- | --- | --- |
| Global | Male | SD | 1990 | 3.9199 | 2.2286 | 6.2093 |
| Global | Female | SD | 1990 | 3.7550 | 2.1350 | 5.9491 |
| Global | Both | SD | 1990 | 3.8252 | 2.1781 | 6.0547 |
| Global | Male | SD | 1992 | 3.9153 | 2.2292 | 6.2264 |
| Global | Female | SD | 1992 | 3.7443 | 2.1341 | 5.9583 |
| Global | Both | SD | 1992 | 3.8181 | 2.1762 | 6.0501 |
| Global | Male | SD | 1994 | 3.9152 | 2.2301 | 6.2123 |
| Global | Female | SD | 1994 | 3.7406 | 2.1347 | 5.9608 |
| Global | Both | SD | 1994 | 3.8168 | 2.1778 | 6.0447 |
| Global | Male | SD | 1991 | 3.9170 | 2.2254 | 6.2310 |
| Global | Female | SD | 1991 | 3.7489 | 2.1354 | 5.9682 |
| Global | Both | SD | 1991 | 3.8210 | 2.1790 | 6.0662 |
| Global | Male | SD | 1995 | 3.9177 | 2.2260 | 6.2450 |
| Global | Female | SD | 1995 | 3.7416 | 2.1307 | 5.9475 |
| Global | Both | SD | 1995 | 3.8189 | 2.1757 | 6.0672 |
| Global | Male | SD | 1993 | 3.9150 | 2.2331 | 6.2056 |
| Global | Female | SD | 1993 | 3.7405 | 2.1304 | 5.9395 |
| Global | Both | SD | 1993 | 3.8163 | 2.1774 | 6.0658 |
| Global | Male | SD | 1999 | 3.9305 | 2.2329 | 6.2422 |
| Global | Female | SD | 1999 | 3.7543 | 2.1356 | 5.9441 |
| Global | Both | SD | 1999 | 3.8328 | 2.1840 | 6.0773 |
| Global | Male | SD | 1996 | 3.9213 | 2.2349 | 6.2267 |
| Global | Female | SD | 1996 | 3.7450 | 2.1315 | 5.9303 |
| Global | Both | SD | 1996 | 3.8226 | 2.1801 | 6.0509 |
| Global | Male | SD | 1997 | 3.9246 | 2.2275 | 6.2338 |
| Global | Female | SD | 1997 | 3.7476 | 2.1290 | 5.9547 |
| Global | Both | SD | 1997 | 3.8259 | 2.1731 | 6.0663 |
| Global | Male | SD | 1998 | 3.9278 | 2.2350 | 6.2495 |
| Global | Female | SD | 1998 | 3.7514 | 2.1410 | 5.9546 |
| Global | Both | SD | 1998 | 3.8297 | 2.1816 | 6.0710 |
| Global | Male | SD | 2000 | 3.9345 | 2.2361 | 6.2564 |
| Global | Female | SD | 2000 | 3.7581 | 2.1429 | 5.9535 |
| Global | Both | SD | 2000 | 3.8370 | 2.1858 | 6.0902 |
| Global | Male | SD | 2002 | 3.9412 | 2.2432 | 6.2627 |
| Global | Female | SD | 2002 | 3.7672 | 2.1477 | 5.9845 |
| Global | Both | SD | 2002 | 3.8454 | 2.1891 | 6.0937 |
| Global | Male | SD | 2001 | 3.9383 | 2.2363 | 6.2621 |
| Global | Female | SD | 2001 | 3.7634 | 2.1443 | 5.9668 |
| Global | Both | SD | 2001 | 3.8418 | 2.1975 | 6.0856 |
| Global | Male | SD | 2004 | 3.9518 | 2.2412 | 6.2972 |
| Global | Female | SD | 2004 | 3.7773 | 2.1549 | 6.0121 |
| Global | Both | SD | 2004 | 3.8562 | 2.1959 | 6.1205 |
| Global | Male | SD | 2006 | 3.9613 | 2.2532 | 6.2918 |
| Global | Female | SD | 2006 | 3.7890 | 2.1597 | 5.9828 |
| Global | Both | SD | 2006 | 3.8672 | 2.2131 | 6.0903 |
| Global | Male | SD | 2007 | 3.9661 | 2.2629 | 6.2926 |
| Global | Female | SD | 2007 | 3.7941 | 2.1556 | 6.0289 |
| Global | Both | SD | 2007 | 3.8723 | 2.2150 | 6.1321 |
| Global | Male | SD | 2003 | 3.9461 | 2.2483 | 6.2551 |
| Global | Female | SD | 2003 | 3.7721 | 2.1380 | 5.9843 |
| Global | Both | SD | 2003 | 3.8505 | 2.1949 | 6.0890 |
| Global | Male | SD | 2005 | 3.9573 | 2.2400 | 6.3009 |
| Global | Female | SD | 2005 | 3.7838 | 2.1494 | 6.0085 |
| Global | Both | SD | 2005 | 3.8623 | 2.2064 | 6.1375 |
| Global | Male | SD | 2009 | 3.9753 | 2.2558 | 6.3200 |
| Global | Female | SD | 2009 | 3.8078 | 2.1705 | 6.0595 |
| Global | Both | SD | 2009 | 3.8841 | 2.2149 | 6.1614 |
| Global | Male | SD | 2012 | 3.9879 | 2.2566 | 6.3699 |
| Global | Female | SD | 2012 | 3.8233 | 2.1805 | 6.0769 |
| Global | Both | SD | 2012 | 3.8985 | 2.2264 | 6.1995 |
| Global | Male | SD | 2011 | 3.9844 | 2.2649 | 6.3511 |
| Global | Female | SD | 2011 | 3.8179 | 2.1787 | 6.0816 |
| Global | Both | SD | 2011 | 3.8939 | 2.2202 | 6.1744 |
| Global | Male | SD | 2008 | 3.9712 | 2.2528 | 6.3338 |
| Global | Female | SD | 2008 | 3.8010 | 2.1642 | 6.0398 |
| Global | Both | SD | 2008 | 3.8785 | 2.2121 | 6.1635 |
| Global | Male | SD | 2010 | 3.9802 | 2.2593 | 6.3335 |
| Global | Female | SD | 2010 | 3.8129 | 2.1751 | 6.0509 |
| Global | Both | SD | 2010 | 3.8892 | 2.2208 | 6.1566 |
| Global | Male | SD | 2017 | 4.0073 | 2.2688 | 6.3830 |
| Global | Female | SD | 2017 | 3.8464 | 2.1889 | 6.1037 |
| Global | Both | SD | 2017 | 3.9203 | 2.2317 | 6.2205 |
| Global | Male | SD | 2014 | 3.9956 | 2.2689 | 6.3744 |
| Global | Female | SD | 2014 | 3.8333 | 2.1762 | 6.1108 |
| Global | Both | SD | 2014 | 3.9076 | 2.2263 | 6.2138 |
| Global | Male | SD | 2016 | 4.0032 | 2.2675 | 6.4051 |
| Global | Female | SD | 2016 | 3.8429 | 2.1938 | 6.1054 |
| Global | Both | SD | 2016 | 3.9164 | 2.2325 | 6.2160 |
| Global | Male | SD | 2013 | 3.9924 | 2.2644 | 6.3657 |
| Global | Female | SD | 2013 | 3.8279 | 2.1722 | 6.0926 |
| Global | Both | SD | 2013 | 3.9032 | 2.2254 | 6.2058 |
| Global | Male | SD | 2015 | 3.9996 | 2.2724 | 6.3764 |
| Global | Female | SD | 2015 | 3.8376 | 2.1854 | 6.1009 |
| Global | Both | SD | 2015 | 3.9118 | 2.2245 | 6.2341 |
| Global | Male | SD | 2019 | 4.0146 | 2.2798 | 6.4224 |
| Global | Female | SD | 2019 | 3.8547 | 2.1929 | 6.1368 |
| Global | Both | SD | 2019 | 3.9284 | 2.2410 | 6.2459 |
| Global | Male | SD | 2018 | 4.0101 | 2.2704 | 6.4123 |
| Global | Female | SD | 2018 | 3.8500 | 2.1833 | 6.1182 |
| Global | Both | SD | 2018 | 3.9237 | 2.2296 | 6.2285 |
|  |  |  |  |  |  |  |

**Table23 DALYs of SD by SDI in 1990~2019, Page 114-124**

| **location_name** | **sex_name** | **cause_name** | **year** | **Age-standardised DALYs rate (per 100 000 person-years)** | **95%CI(lower)** | **95%CI(upper)** |
| --- | --- | --- | --- | --- | --- | --- |
| High-middle SDI | Male | SD | 1990 | 3.3203 | 1.8733 | 5.3067 |
| High-middle SDI | Female | SD | 1990 | 3.0328 | 1.7169 | 4.8180 |
| High-middle SDI | Both | SD | 1990 | 3.1652 | 1.7980 | 5.0345 |
| Low-middle SDI | Male | SD | 1991 | 3.8034 | 2.1621 | 5.9957 |
| Low-middle SDI | Female | SD | 1991 | 3.7924 | 2.1528 | 5.9795 |
| Low-middle SDI | Both | SD | 1991 | 3.7961 | 2.1668 | 5.9793 |
| Low SDI | Male | SD | 1991 | 4.7877 | 2.7145 | 7.6723 |
| Low SDI | Female | SD | 1991 | 4.8771 | 2.7930 | 7.6840 |
| Low SDI | Both | SD | 1991 | 4.8324 | 2.7575 | 7.6736 |
| High SDI | Male | SD | 1990 | 4.2775 | 2.4412 | 6.8334 |
| High SDI | Female | SD | 1990 | 4.6474 | 2.6426 | 7.3986 |
| High SDI | Both | SD | 1990 | 4.4234 | 2.5162 | 7.0154 |
| Low-middle SDI | Male | SD | 1990 | 3.8006 | 2.1541 | 6.0114 |
| Low-middle SDI | Female | SD | 1990 | 3.7896 | 2.1576 | 5.9967 |
| Low-middle SDI | Both | SD | 1990 | 3.7934 | 2.1545 | 6.0064 |
| Low SDI | Male | SD | 1990 | 4.7847 | 2.7201 | 7.5809 |
| Low SDI | Female | SD | 1990 | 4.8785 | 2.7957 | 7.7269 |
| Low SDI | Both | SD | 1990 | 4.8315 | 2.7649 | 7.6447 |
| Middle SDI | Male | SD | 1990 | 3.7728 | 2.1355 | 6.0280 |
| Middle SDI | Female | SD | 1990 | 3.3620 | 1.9081 | 5.3246 |
| Middle SDI | Both | SD | 1990 | 3.5709 | 2.0210 | 5.6727 |
| Middle SDI | Male | SD | 1991 | 3.7784 | 2.1489 | 6.0518 |
| Middle SDI | Female | SD | 1991 | 3.3625 | 1.9155 | 5.3500 |
| Middle SDI | Both | SD | 1991 | 3.5741 | 2.0386 | 5.6961 |
| High-middle SDI | Male | SD | 1992 | 3.3312 | 1.8959 | 5.3455 |
| High-middle SDI | Female | SD | 1992 | 3.0403 | 1.7255 | 4.8716 |
| High-middle SDI | Both | SD | 1992 | 3.1747 | 1.8016 | 5.0516 |
| High-middle SDI | Male | SD | 1991 | 3.3239 | 1.8819 | 5.2935 |
| High-middle SDI | Female | SD | 1991 | 3.0351 | 1.7285 | 4.8084 |
| High-middle SDI | Both | SD | 1991 | 3.1683 | 1.8003 | 5.0408 |
| High SDI | Male | SD | 1992 | 4.2126 | 2.3894 | 6.6906 |
| High SDI | Female | SD | 1992 | 4.5680 | 2.5931 | 7.2564 |
| High SDI | Both | SD | 1992 | 4.3521 | 2.4855 | 6.9016 |
| High SDI | Male | SD | 1991 | 4.2424 | 2.4193 | 6.7260 |
| High SDI | Female | SD | 1991 | 4.6074 | 2.6049 | 7.2994 |
| High SDI | Both | SD | 1991 | 4.3862 | 2.4950 | 6.9493 |
| Low-middle SDI | Male | SD | 1992 | 3.8033 | 2.1680 | 6.0231 |
| Low-middle SDI | Female | SD | 1992 | 3.7920 | 2.1630 | 5.9695 |
| Low-middle SDI | Both | SD | 1992 | 3.7958 | 2.1644 | 5.9866 |
| Low SDI | Male | SD | 1992 | 4.7934 | 2.7341 | 7.6267 |
| Low SDI | Female | SD | 1992 | 4.8742 | 2.7856 | 7.7179 |
| Low SDI | Both | SD | 1992 | 4.8338 | 2.7618 | 7.6925 |
| Middle SDI | Male | SD | 1992 | 3.7827 | 2.1243 | 5.9990 |
| Middle SDI | Female | SD | 1992 | 3.3673 | 1.9092 | 5.3652 |
| Middle SDI | Both | SD | 1992 | 3.5788 | 2.0316 | 5.6809 |
| High SDI | Male | SD | 1993 | 4.1863 | 2.3925 | 6.6380 |
| High SDI | Female | SD | 1993 | 4.5369 | 2.5803 | 7.2033 |
| High SDI | Both | SD | 1993 | 4.3238 | 2.4704 | 6.8652 |
| Low SDI | Male | SD | 1993 | 4.7963 | 2.7192 | 7.5682 |
| Low SDI | Female | SD | 1993 | 4.8741 | 2.7902 | 7.7419 |
| Low SDI | Both | SD | 1993 | 4.8351 | 2.7605 | 7.6646 |
| High-middle SDI | Male | SD | 1993 | 3.3370 | 1.8978 | 5.3397 |
| High-middle SDI | Female | SD | 1993 | 3.0446 | 1.7287 | 4.8439 |
| High-middle SDI | Both | SD | 1993 | 3.1799 | 1.8064 | 5.0501 |
| Middle SDI | Male | SD | 1993 | 3.7878 | 2.1513 | 6.0342 |
| Middle SDI | Female | SD | 1993 | 3.3656 | 1.9044 | 5.3752 |
| Middle SDI | Both | SD | 1993 | 3.5807 | 2.0374 | 5.7042 |
| Low-middle SDI | Male | SD | 1993 | 3.8063 | 2.1630 | 6.0016 |
| Low-middle SDI | Female | SD | 1993 | 3.7960 | 2.1536 | 6.0035 |
| Low-middle SDI | Both | SD | 1993 | 3.7994 | 2.1654 | 5.9995 |
| Middle SDI | Male | SD | 1994 | 3.7906 | 2.1535 | 6.0887 |
| Middle SDI | Female | SD | 1994 | 3.3710 | 1.9219 | 5.3773 |
| Middle SDI | Both | SD | 1994 | 3.5850 | 2.0454 | 5.6986 |
| High-middle SDI | Male | SD | 1995 | 3.3505 | 1.8970 | 5.3655 |
| High-middle SDI | Female | SD | 1995 | 3.0554 | 1.7372 | 4.9013 |
| High-middle SDI | Both | SD | 1995 | 3.1927 | 1.8079 | 5.0639 |
| Middle SDI | Male | SD | 1995 | 3.7932 | 2.1489 | 6.1047 |
| Middle SDI | Female | SD | 1995 | 3.3704 | 1.9115 | 5.3604 |
| Middle SDI | Both | SD | 1995 | 3.5863 | 2.0351 | 5.7160 |
| High SDI | Male | SD | 1995 | 4.1644 | 2.3710 | 6.6181 |
| High SDI | Female | SD | 1995 | 4.5068 | 2.5642 | 7.1891 |
| High SDI | Both | SD | 1995 | 4.2989 | 2.4490 | 6.8540 |
| High-middle SDI | Male | SD | 1994 | 3.3423 | 1.9049 | 5.3411 |
| High-middle SDI | Female | SD | 1994 | 3.0494 | 1.7423 | 4.8933 |
| High-middle SDI | Both | SD | 1994 | 3.1853 | 1.8136 | 5.0337 |
| High SDI | Male | SD | 1994 | 4.1700 | 2.3660 | 6.6238 |
| High SDI | Female | SD | 1994 | 4.5158 | 2.5664 | 7.1866 |
| High SDI | Both | SD | 1994 | 4.3057 | 2.4510 | 6.8610 |
| Low-middle SDI | Male | SD | 1995 | 3.8085 | 2.1786 | 6.0807 |
| Low-middle SDI | Female | SD | 1995 | 3.7998 | 2.1681 | 6.0206 |
| Low-middle SDI | Both | SD | 1995 | 3.8023 | 2.1681 | 6.0409 |
| Low-middle SDI | Male | SD | 1994 | 3.8087 | 2.1743 | 6.0143 |
| Low-middle SDI | Female | SD | 1994 | 3.7977 | 2.1647 | 5.9914 |
| Low-middle SDI | Both | SD | 1994 | 3.8014 | 2.1779 | 5.9775 |
| Low SDI | Male | SD | 1995 | 4.7987 | 2.7444 | 7.6058 |
| Low SDI | Female | SD | 1995 | 4.8730 | 2.7941 | 7.7062 |
| Low SDI | Both | SD | 1995 | 4.8357 | 2.7761 | 7.6922 |
| Low SDI | Male | SD | 1994 | 4.7967 | 2.7265 | 7.6384 |
| Low SDI | Female | SD | 1994 | 4.8743 | 2.7974 | 7.7525 |
| Low SDI | Both | SD | 1994 | 4.8354 | 2.7550 | 7.6788 |
| Low-middle SDI | Male | SD | 1996 | 3.8114 | 2.1719 | 6.0211 |
| Low-middle SDI | Female | SD | 1996 | 3.8013 | 2.1626 | 5.9907 |
| Low-middle SDI | Both | SD | 1996 | 3.8045 | 2.1702 | 5.9912 |
| Low SDI | Male | SD | 1996 | 4.8028 | 2.7452 | 7.5902 |
| Low SDI | Female | SD | 1996 | 4.8748 | 2.7848 | 7.7601 |
| Low SDI | Both | SD | 1996 | 4.8386 | 2.7585 | 7.6625 |
| Middle SDI | Male | SD | 1996 | 3.7954 | 2.1562 | 6.0307 |
| Middle SDI | Female | SD | 1996 | 3.3732 | 1.9088 | 5.3640 |
| Middle SDI | Both | SD | 1996 | 3.5889 | 2.0370 | 5.6953 |
| High-middle SDI | Male | SD | 1996 | 3.3548 | 1.9123 | 5.3231 |
| High-middle SDI | Female | SD | 1996 | 3.0615 | 1.7426 | 4.8791 |
| High-middle SDI | Both | SD | 1996 | 3.1980 | 1.8235 | 5.0623 |
| High SDI | Male | SD | 1996 | 4.1659 | 2.3793 | 6.6208 |
| High SDI | Female | SD | 1996 | 4.5065 | 2.5630 | 7.1757 |
| High SDI | Both | SD | 1996 | 4.3001 | 2.4558 | 6.8280 |
| High-middle SDI | Male | SD | 1998 | 3.3649 | 1.9064 | 5.3647 |
| High-middle SDI | Female | SD | 1998 | 3.0731 | 1.7486 | 4.9098 |
| High-middle SDI | Both | SD | 1998 | 3.2096 | 1.8214 | 5.1194 |
| High-middle SDI | Male | SD | 1997 | 3.3586 | 1.9030 | 5.3515 |
| High-middle SDI | Female | SD | 1997 | 3.0667 | 1.7385 | 4.8769 |
| High-middle SDI | Both | SD | 1997 | 3.2030 | 1.8081 | 5.1014 |
| Low SDI | Male | SD | 1998 | 4.8092 | 2.7285 | 7.6220 |
| Low SDI | Female | SD | 1998 | 4.8783 | 2.7872 | 7.7579 |
| Low SDI | Both | SD | 1998 | 4.8435 | 2.7590 | 7.6996 |
| High SDI | Male | SD | 1998 | 4.1672 | 2.3623 | 6.6305 |
| High SDI | Female | SD | 1998 | 4.5030 | 2.5644 | 7.1509 |
| High SDI | Both | SD | 1998 | 4.3001 | 2.4439 | 6.8332 |
| Low-middle SDI | Male | SD | 1998 | 3.8180 | 2.1819 | 5.9938 |
| Low-middle SDI | Female | SD | 1998 | 3.8065 | 2.1516 | 6.0563 |
| Low-middle SDI | Both | SD | 1998 | 3.8102 | 2.1667 | 6.0062 |
| Low-middle SDI | Male | SD | 1997 | 3.8162 | 2.1639 | 6.0535 |
| Low-middle SDI | Female | SD | 1997 | 3.8026 | 2.1664 | 6.0079 |
| Low-middle SDI | Both | SD | 1997 | 3.8075 | 2.1716 | 6.0174 |
| Middle SDI | Male | SD | 1999 | 3.7999 | 2.1590 | 6.0728 |
| Middle SDI | Female | SD | 1999 | 3.3770 | 1.9167 | 5.3993 |
| Middle SDI | Both | SD | 1999 | 3.5935 | 2.0491 | 5.7180 |
| High SDI | Male | SD | 1997 | 4.1657 | 2.3720 | 6.6185 |
| High SDI | Female | SD | 1997 | 4.5051 | 2.5577 | 7.1817 |
| High SDI | Both | SD | 1997 | 4.2998 | 2.4479 | 6.8292 |
| Low SDI | Male | SD | 1997 | 4.8060 | 2.7418 | 7.5985 |
| Low SDI | Female | SD | 1997 | 4.8770 | 2.7797 | 7.7783 |
| Low SDI | Both | SD | 1997 | 4.8413 | 2.7671 | 7.7049 |
| Middle SDI | Male | SD | 1997 | 3.7963 | 2.1597 | 6.0829 |
| Middle SDI | Female | SD | 1997 | 3.3737 | 1.9085 | 5.3612 |
| Middle SDI | Both | SD | 1997 | 3.5898 | 2.0390 | 5.6888 |
| Middle SDI | Male | SD | 1998 | 3.7970 | 2.1539 | 6.0893 |
| Middle SDI | Female | SD | 1998 | 3.3756 | 1.9162 | 5.3570 |
| Middle SDI | Both | SD | 1998 | 3.5912 | 2.0365 | 5.7247 |
| High-middle SDI | Male | SD | 1999 | 3.3694 | 1.9102 | 5.3630 |
| High-middle SDI | Female | SD | 1999 | 3.0780 | 1.7581 | 4.8595 |
| High-middle SDI | Both | SD | 1999 | 3.2147 | 1.8270 | 5.1105 |
| High SDI | Male | SD | 1999 | 4.1679 | 2.3693 | 6.6196 |
| High SDI | Female | SD | 1999 | 4.5029 | 2.5704 | 7.1512 |
| High SDI | Both | SD | 1999 | 4.3011 | 2.4436 | 6.8308 |
| Low-middle SDI | Male | SD | 1999 | 3.8182 | 2.1648 | 6.0767 |
| Low-middle SDI | Female | SD | 1999 | 3.8084 | 2.1529 | 6.0290 |
| Low-middle SDI | Both | SD | 1999 | 3.8112 | 2.1651 | 6.0365 |
| Low SDI | Male | SD | 1999 | 4.8122 | 2.7604 | 7.6102 |
| Low SDI | Female | SD | 1999 | 4.8799 | 2.7955 | 7.7804 |
| Low SDI | Both | SD | 1999 | 4.8458 | 2.7764 | 7.7086 |
| High-middle SDI | Male | SD | 2000 | 3.3753 | 1.9139 | 5.4274 |
| High-middle SDI | Female | SD | 2000 | 3.0845 | 1.7593 | 4.9319 |
| High-middle SDI | Both | SD | 2000 | 3.2213 | 1.8419 | 5.1521 |
| High SDI | Male | SD | 2000 | 4.1691 | 2.3743 | 6.6449 |
| High SDI | Female | SD | 2000 | 4.5035 | 2.5618 | 7.1631 |
| High SDI | Both | SD | 2000 | 4.3028 | 2.4474 | 6.8590 |
| Low-middle SDI | Male | SD | 2000 | 3.8239 | 2.1764 | 6.0143 |
| Low-middle SDI | Female | SD | 2000 | 3.8113 | 2.1728 | 6.0386 |
| Low-middle SDI | Both | SD | 2000 | 3.8155 | 2.1715 | 6.0079 |
| Low SDI | Male | SD | 2000 | 4.8146 | 2.7469 | 7.6814 |
| Low SDI | Female | SD | 2000 | 4.8809 | 2.7907 | 7.7467 |
| Low SDI | Both | SD | 2000 | 4.8474 | 2.7729 | 7.7011 |
| Middle SDI | Male | SD | 2000 | 3.8010 | 2.1452 | 6.0533 |
| Middle SDI | Female | SD | 2000 | 3.3776 | 1.9255 | 5.3696 |
| Middle SDI | Both | SD | 2000 | 3.5945 | 2.0428 | 5.7085 |
| Low SDI | Male | SD | 2001 | 4.8188 | 2.7474 | 7.6502 |
| Low SDI | Female | SD | 2001 | 4.8822 | 2.7903 | 7.7769 |
| Low SDI | Both | SD | 2001 | 4.8501 | 2.7790 | 7.7216 |
| Low-middle SDI | Male | SD | 2001 | 3.8252 | 2.1812 | 6.0496 |
| Low-middle SDI | Female | SD | 2001 | 3.8160 | 2.1722 | 6.0576 |
| Low-middle SDI | Both | SD | 2001 | 3.8184 | 2.1827 | 6.0453 |
| High SDI | Male | SD | 2003 | 4.1731 | 2.3743 | 6.6496 |
| High SDI | Female | SD | 2003 | 4.5027 | 2.5469 | 7.2049 |
| High SDI | Both | SD | 2003 | 4.3066 | 2.4451 | 6.8876 |
| Middle SDI | Male | SD | 2003 | 3.8063 | 2.1489 | 6.0277 |
| Middle SDI | Female | SD | 2003 | 3.3848 | 1.9181 | 5.3710 |
| Middle SDI | Both | SD | 2003 | 3.6010 | 2.0442 | 5.7069 |
| High SDI | Male | SD | 2002 | 4.1703 | 2.3759 | 6.6489 |
| High SDI | Female | SD | 2002 | 4.5024 | 2.5490 | 7.1660 |
| High SDI | Both | SD | 2002 | 4.3043 | 2.4470 | 6.8440 |
| High-middle SDI | Male | SD | 2001 | 3.3827 | 1.9236 | 5.3902 |
| High-middle SDI | Female | SD | 2001 | 3.0912 | 1.7594 | 4.9208 |
| High-middle SDI | Both | SD | 2001 | 3.2288 | 1.8407 | 5.1246 |
| Middle SDI | Male | SD | 2001 | 3.8023 | 2.1522 | 6.0413 |
| Middle SDI | Female | SD | 2001 | 3.3810 | 1.9157 | 5.3737 |
| Middle SDI | Both | SD | 2001 | 3.5969 | 2.0497 | 5.7135 |
| Low-middle SDI | Male | SD | 2003 | 3.8302 | 2.1735 | 6.0425 |
| Low-middle SDI | Female | SD | 2003 | 3.8202 | 2.1717 | 6.0383 |
| Low-middle SDI | Both | SD | 2003 | 3.8229 | 2.1820 | 6.0226 |
| Low-middle SDI | Male | SD | 2002 | 3.8246 | 2.1868 | 6.0112 |
| Low-middle SDI | Female | SD | 2002 | 3.8190 | 2.1621 | 6.0563 |
| Low-middle SDI | Both | SD | 2002 | 3.8195 | 2.1775 | 6.0447 |
| High SDI | Male | SD | 2001 | 4.1697 | 2.3813 | 6.6408 |
| High SDI | Female | SD | 2001 | 4.5035 | 2.5514 | 7.2019 |
| High SDI | Both | SD | 2001 | 4.3038 | 2.4506 | 6.8518 |
| High-middle SDI | Male | SD | 2002 | 3.3880 | 1.9143 | 5.3914 |
| High-middle SDI | Female | SD | 2002 | 3.0958 | 1.7613 | 4.9208 |
| High-middle SDI | Both | SD | 2002 | 3.2341 | 1.8356 | 5.1438 |
| Low SDI | Male | SD | 2003 | 4.8260 | 2.7576 | 7.7378 |
| Low SDI | Female | SD | 2003 | 4.8855 | 2.7771 | 7.7695 |
| Low SDI | Both | SD | 2003 | 4.8553 | 2.7700 | 7.7145 |
| Low SDI | Male | SD | 2002 | 4.8236 | 2.7478 | 7.7149 |
| Low SDI | Female | SD | 2002 | 4.8836 | 2.7890 | 7.7608 |
| Low SDI | Both | SD | 2002 | 4.8532 | 2.7686 | 7.7324 |
| High-middle SDI | Male | SD | 2003 | 3.3939 | 1.9257 | 5.3782 |
| High-middle SDI | Female | SD | 2003 | 3.1017 | 1.7574 | 4.9767 |
| High-middle SDI | Both | SD | 2003 | 3.2404 | 1.8316 | 5.1665 |
| Middle SDI | Male | SD | 2002 | 3.8043 | 2.1528 | 6.0101 |
| Middle SDI | Female | SD | 2002 | 3.3818 | 1.9079 | 5.4166 |
| Middle SDI | Both | SD | 2002 | 3.5985 | 2.0431 | 5.7093 |
| Middle SDI | Male | SD | 2004 | 3.8108 | 2.1671 | 6.0620 |
| Middle SDI | Female | SD | 2004 | 3.3869 | 1.9248 | 5.3872 |
| Middle SDI | Both | SD | 2004 | 3.6044 | 2.0480 | 5.7484 |
| High-middle SDI | Male | SD | 2005 | 3.4124 | 1.9153 | 5.4368 |
| High-middle SDI | Female | SD | 2005 | 3.1123 | 1.7716 | 4.9680 |
| High-middle SDI | Both | SD | 2005 | 3.2556 | 1.8392 | 5.2020 |
| High SDI | Male | SD | 2005 | 4.1772 | 2.3737 | 6.6435 |
| High SDI | Female | SD | 2005 | 4.5008 | 2.5497 | 7.1521 |
| High SDI | Both | SD | 2005 | 4.3092 | 2.4576 | 6.8410 |
| Low-middle SDI | Male | SD | 2005 | 3.8374 | 2.2012 | 6.1062 |
| Low-middle SDI | Female | SD | 2005 | 3.8313 | 2.1786 | 6.0370 |
| Low-middle SDI | Both | SD | 2005 | 3.8318 | 2.1910 | 6.0659 |
| High-middle SDI | Male | SD | 2004 | 3.4035 | 1.9142 | 5.3659 |
| High-middle SDI | Female | SD | 2004 | 3.1068 | 1.7757 | 4.9753 |
| High-middle SDI | Both | SD | 2004 | 3.2481 | 1.8444 | 5.1566 |
| Low SDI | Male | SD | 2005 | 4.8353 | 2.7497 | 7.6694 |
| Low SDI | Female | SD | 2005 | 4.8885 | 2.7833 | 7.7941 |
| Low SDI | Both | SD | 2005 | 4.8613 | 2.7750 | 7.7256 |
| High SDI | Male | SD | 2004 | 4.1733 | 2.3887 | 6.6500 |
| High SDI | Female | SD | 2004 | 4.5010 | 2.5576 | 7.1278 |
| High SDI | Both | SD | 2004 | 4.3066 | 2.4595 | 6.8603 |
| Low-middle SDI | Male | SD | 2004 | 3.8344 | 2.1850 | 6.1056 |
| Low-middle SDI | Female | SD | 2004 | 3.8268 | 2.1751 | 6.0583 |
| Low-middle SDI | Both | SD | 2004 | 3.8282 | 2.1798 | 6.0847 |
| Low SDI | Male | SD | 2004 | 4.8313 | 2.7461 | 7.6580 |
| Low SDI | Female | SD | 2004 | 4.8834 | 2.7772 | 7.7802 |
| Low SDI | Both | SD | 2004 | 4.8568 | 2.7706 | 7.7044 |
| Middle SDI | Male | SD | 2005 | 3.8138 | 2.1415 | 6.0995 |
| Middle SDI | Female | SD | 2005 | 3.3905 | 1.9269 | 5.3952 |
| Middle SDI | Both | SD | 2005 | 3.6077 | 2.0426 | 5.7548 |
| High SDI | Male | SD | 2008 | 4.1831 | 2.3875 | 6.6357 |
| High SDI | Female | SD | 2008 | 4.4941 | 2.5614 | 7.1323 |
| High SDI | Both | SD | 2008 | 4.3100 | 2.4576 | 6.8564 |
| Low SDI | Male | SD | 2006 | 4.8370 | 2.7402 | 7.6710 |
| Low SDI | Female | SD | 2006 | 4.8904 | 2.7761 | 7.7804 |
| Low SDI | Both | SD | 2006 | 4.8631 | 2.7651 | 7.7235 |
| Low-middle SDI | Male | SD | 2008 | 3.8516 | 2.1840 | 6.1221 |
| Low-middle SDI | Female | SD | 2008 | 3.8473 | 2.1889 | 6.1146 |
| Low-middle SDI | Both | SD | 2008 | 3.8466 | 2.1881 | 6.1158 |
| Low SDI | Male | SD | 2008 | 4.8452 | 2.7610 | 7.7386 |
| Low SDI | Female | SD | 2008 | 4.8932 | 2.7894 | 7.6994 |
| Low SDI | Both | SD | 2008 | 4.8684 | 2.7756 | 7.7290 |
| High-middle SDI | Male | SD | 2007 | 3.4259 | 1.9514 | 5.4746 |
| High-middle SDI | Female | SD | 2007 | 3.1252 | 1.7669 | 4.9676 |
| High-middle SDI | Both | SD | 2007 | 3.2695 | 1.8663 | 5.2154 |
| Middle SDI | Male | SD | 2008 | 3.8214 | 2.1654 | 6.0973 |
| Middle SDI | Female | SD | 2008 | 3.3957 | 1.9251 | 5.3948 |
| Middle SDI | Both | SD | 2008 | 3.6145 | 2.0552 | 5.7453 |
| High SDI | Male | SD | 2006 | 4.1785 | 2.3863 | 6.6615 |
| High SDI | Female | SD | 2006 | 4.4988 | 2.5594 | 7.1713 |
| High SDI | Both | SD | 2006 | 4.3095 | 2.4589 | 6.8404 |
| High-middle SDI | Male | SD | 2006 | 3.4189 | 1.9398 | 5.4526 |
| High-middle SDI | Female | SD | 2006 | 3.1190 | 1.7663 | 4.9524 |
| High-middle SDI | Both | SD | 2006 | 3.2626 | 1.8524 | 5.2046 |
| Middle SDI | Male | SD | 2006 | 3.8166 | 2.1653 | 6.0893 |
| Middle SDI | Female | SD | 2006 | 3.3908 | 1.9304 | 5.3668 |
| Middle SDI | Both | SD | 2006 | 3.6094 | 2.0487 | 5.7092 |
| High SDI | Male | SD | 2007 | 4.1818 | 2.3829 | 6.6382 |
| High SDI | Female | SD | 2007 | 4.4968 | 2.5474 | 7.1223 |
| High SDI | Both | SD | 2007 | 4.3105 | 2.4592 | 6.8308 |
| High-middle SDI | Male | SD | 2008 | 3.4324 | 1.9602 | 5.4966 |
| High-middle SDI | Female | SD | 2008 | 3.1297 | 1.7734 | 5.0185 |
| High-middle SDI | Both | SD | 2008 | 3.2754 | 1.8562 | 5.2562 |
| Low-middle SDI | Male | SD | 2006 | 3.8414 | 2.1841 | 6.0293 |
| Low-middle SDI | Female | SD | 2006 | 3.8366 | 2.1805 | 6.0631 |
| Low-middle SDI | Both | SD | 2006 | 3.8364 | 2.1867 | 6.0160 |
| Middle SDI | Male | SD | 2007 | 3.8186 | 2.1600 | 6.0971 |
| Middle SDI | Female | SD | 2007 | 3.3920 | 1.9174 | 5.3799 |
| Middle SDI | Both | SD | 2007 | 3.6111 | 2.0503 | 5.7296 |
| Low-middle SDI | Male | SD | 2007 | 3.8469 | 2.1936 | 6.0600 |
| Low-middle SDI | Female | SD | 2007 | 3.8414 | 2.1865 | 6.0715 |
| Low-middle SDI | Both | SD | 2007 | 3.8414 | 2.1832 | 6.0864 |
| Low SDI | Male | SD | 2007 | 4.8387 | 2.7462 | 7.7077 |
| Low SDI | Female | SD | 2007 | 4.8887 | 2.7873 | 7.7047 |
| Low SDI | Both | SD | 2007 | 4.8631 | 2.7745 | 7.7207 |
| Middle SDI | Male | SD | 2009 | 3.8257 | 2.1797 | 6.1038 |
| Middle SDI | Female | SD | 2009 | 3.3990 | 1.9326 | 5.3965 |
| Middle SDI | Both | SD | 2009 | 3.6184 | 2.0616 | 5.7448 |
| High-middle SDI | Male | SD | 2009 | 3.4370 | 1.9456 | 5.4953 |
| High-middle SDI | Female | SD | 2009 | 3.1363 | 1.7749 | 5.0102 |
| High-middle SDI | Both | SD | 2009 | 3.2813 | 1.8618 | 5.2242 |
| High SDI | Male | SD | 2009 | 4.1828 | 2.3709 | 6.6499 |
| High SDI | Female | SD | 2009 | 4.4908 | 2.5579 | 7.1213 |
| High SDI | Both | SD | 2009 | 4.3083 | 2.4467 | 6.8451 |
| Low-middle SDI | Male | SD | 2009 | 3.8548 | 2.2025 | 6.1044 |
| Low-middle SDI | Female | SD | 2009 | 3.8532 | 2.1911 | 6.1282 |
| Low-middle SDI | Both | SD | 2009 | 3.8510 | 2.1916 | 6.0985 |
| Low SDI | Male | SD | 2009 | 4.8462 | 2.7635 | 7.6887 |
| Low SDI | Female | SD | 2009 | 4.8965 | 2.8021 | 7.7599 |
| Low SDI | Both | SD | 2009 | 4.8706 | 2.7898 | 7.7456 |
| High-middle SDI | Male | SD | 2010 | 3.4435 | 1.9420 | 5.4761 |
| High-middle SDI | Female | SD | 2010 | 3.1397 | 1.7843 | 4.9925 |
| High-middle SDI | Both | SD | 2010 | 3.2865 | 1.8651 | 5.2220 |
| High SDI | Male | SD | 2012 | 4.1823 | 2.3762 | 6.6407 |
| High SDI | Female | SD | 2012 | 4.4876 | 2.5430 | 7.1751 |
| High SDI | Both | SD | 2012 | 4.3079 | 2.4299 | 6.8883 |
| High SDI | Male | SD | 2010 | 4.1834 | 2.3680 | 6.6670 |
| High SDI | Female | SD | 2010 | 4.4897 | 2.5563 | 7.2051 |
| High SDI | Both | SD | 2010 | 4.3086 | 2.4483 | 6.8795 |
| Low-middle SDI | Male | SD | 2010 | 3.8600 | 2.1893 | 6.1375 |
| Low-middle SDI | Female | SD | 2010 | 3.8585 | 2.1919 | 6.1289 |
| Low-middle SDI | Both | SD | 2010 | 3.8561 | 2.1953 | 6.1338 |
| Low-middle SDI | Male | SD | 2012 | 3.8689 | 2.1914 | 6.1846 |
| Low-middle SDI | Female | SD | 2012 | 3.8675 | 2.2187 | 6.1594 |
| Low-middle SDI | Both | SD | 2012 | 3.8649 | 2.2105 | 6.1640 |
| Low SDI | Male | SD | 2010 | 4.8489 | 2.7648 | 7.7205 |
| Low SDI | Female | SD | 2010 | 4.8968 | 2.8058 | 7.7992 |
| Low SDI | Both | SD | 2010 | 4.8720 | 2.7845 | 7.7594 |
| High-middle SDI | Male | SD | 2012 | 3.4512 | 1.9526 | 5.5154 |
| High-middle SDI | Female | SD | 2012 | 3.1454 | 1.7819 | 4.9992 |
| High-middle SDI | Both | SD | 2012 | 3.2938 | 1.8663 | 5.2376 |
| Middle SDI | Male | SD | 2011 | 3.8310 | 2.1757 | 6.0974 |
| Middle SDI | Female | SD | 2011 | 3.3998 | 1.9341 | 5.4209 |
| Middle SDI | Both | SD | 2011 | 3.6216 | 2.0604 | 5.7631 |
| Middle SDI | Male | SD | 2010 | 3.8285 | 2.1745 | 6.1282 |
| Middle SDI | Female | SD | 2010 | 3.3989 | 1.9295 | 5.3999 |
| Middle SDI | Both | SD | 2010 | 3.6199 | 2.0622 | 5.7186 |
| Low SDI | Male | SD | 2012 | 4.8552 | 2.7726 | 7.7439 |
| Low SDI | Female | SD | 2012 | 4.9024 | 2.7952 | 7.7792 |
| Low SDI | Both | SD | 2012 | 4.8779 | 2.7813 | 7.7425 |
| Middle SDI | Male | SD | 2012 | 3.8338 | 2.1681 | 6.1168 |
| Middle SDI | Female | SD | 2012 | 3.4018 | 1.9336 | 5.4206 |
| Middle SDI | Both | SD | 2012 | 3.6241 | 2.0529 | 5.7922 |
| High-middle SDI | Male | SD | 2011 | 3.4489 | 1.9574 | 5.5069 |
| High-middle SDI | Female | SD | 2011 | 3.1441 | 1.7750 | 5.0407 |
| High-middle SDI | Both | SD | 2011 | 3.2917 | 1.8689 | 5.2673 |
| High SDI | Male | SD | 2011 | 4.1828 | 2.3723 | 6.6661 |
| High SDI | Female | SD | 2011 | 4.4876 | 2.5511 | 7.1395 |
| High SDI | Both | SD | 2011 | 4.3077 | 2.4383 | 6.8684 |
| Low-middle SDI | Male | SD | 2011 | 3.8647 | 2.1922 | 6.1261 |
| Low-middle SDI | Female | SD | 2011 | 3.8626 | 2.2016 | 6.1237 |
| Low-middle SDI | Both | SD | 2011 | 3.8604 | 2.2032 | 6.1158 |
| Low SDI | Male | SD | 2011 | 4.8533 | 2.7688 | 7.6830 |
| Low SDI | Female | SD | 2011 | 4.9004 | 2.8004 | 7.7737 |
| Low SDI | Both | SD | 2011 | 4.8759 | 2.7953 | 7.7244 |
| High SDI | Male | SD | 2013 | 4.1845 | 2.3740 | 6.6830 |
| High SDI | Female | SD | 2013 | 4.4860 | 2.5468 | 7.1002 |
| High SDI | Both | SD | 2013 | 4.3086 | 2.4494 | 6.8672 |
| Low-middle SDI | Male | SD | 2013 | 3.8743 | 2.2221 | 6.1405 |
| Low-middle SDI | Female | SD | 2013 | 3.8730 | 2.2156 | 6.1435 |
| Low-middle SDI | Both | SD | 2013 | 3.8702 | 2.2163 | 6.1483 |
| Low SDI | Male | SD | 2013 | 4.8573 | 2.7539 | 7.6999 |
| Low SDI | Female | SD | 2013 | 4.9033 | 2.7953 | 7.8194 |
| Low SDI | Both | SD | 2013 | 4.8794 | 2.7833 | 7.7401 |
| Middle SDI | Male | SD | 2013 | 3.8359 | 2.1637 | 6.1242 |
| Middle SDI | Female | SD | 2013 | 3.4028 | 1.9191 | 5.4011 |
| Middle SDI | Both | SD | 2013 | 3.6257 | 2.0639 | 5.7726 |
| High-middle SDI | Male | SD | 2013 | 3.4555 | 1.9555 | 5.5259 |
| High-middle SDI | Female | SD | 2013 | 3.1477 | 1.7767 | 5.0074 |
| High-middle SDI | Both | SD | 2013 | 3.2974 | 1.8651 | 5.2552 |
| Middle SDI | Male | SD | 2016 | 3.8439 | 2.1793 | 6.1303 |
| Middle SDI | Female | SD | 2016 | 3.4056 | 1.9309 | 5.4234 |
| Middle SDI | Both | SD | 2016 | 3.6314 | 2.0689 | 5.7787 |
| High SDI | Male | SD | 2014 | 4.1833 | 2.3737 | 6.6481 |
| High SDI | Female | SD | 2014 | 4.4839 | 2.5439 | 7.1149 |
| High SDI | Both | SD | 2014 | 4.3073 | 2.4498 | 6.8615 |
| High-middle SDI | Male | SD | 2015 | 3.4573 | 1.9658 | 5.5483 |
| High-middle SDI | Female | SD | 2015 | 3.1509 | 1.7836 | 5.0256 |
| High-middle SDI | Both | SD | 2015 | 3.3003 | 1.8750 | 5.2716 |
| High SDI | Male | SD | 2015 | 4.1842 | 2.3754 | 6.6448 |
| High SDI | Female | SD | 2015 | 4.4850 | 2.5419 | 7.1889 |
| High SDI | Both | SD | 2015 | 4.3086 | 2.4494 | 6.8613 |
| Middle SDI | Male | SD | 2015 | 3.8411 | 2.1737 | 6.1839 |
| Middle SDI | Female | SD | 2015 | 3.4050 | 1.9306 | 5.4358 |
| Middle SDI | Both | SD | 2015 | 3.6296 | 2.0642 | 5.8184 |
| Low-middle SDI | Male | SD | 2014 | 3.8791 | 2.2175 | 6.1674 |
| Low-middle SDI | Female | SD | 2014 | 3.8798 | 2.2080 | 6.1686 |
| Low-middle SDI | Both | SD | 2014 | 3.8759 | 2.2097 | 6.1684 |
| High-middle SDI | Male | SD | 2014 | 3.4555 | 1.9466 | 5.5391 |
| High-middle SDI | Female | SD | 2014 | 3.1478 | 1.7917 | 5.0472 |
| High-middle SDI | Both | SD | 2014 | 3.2976 | 1.8638 | 5.2727 |
| High-middle SDI | Male | SD | 2016 | 3.4564 | 1.9529 | 5.5363 |
| High-middle SDI | Female | SD | 2016 | 3.1531 | 1.7824 | 5.0309 |
| High-middle SDI | Both | SD | 2016 | 3.3012 | 1.8736 | 5.2758 |
| High SDI | Male | SD | 2016 | 4.1850 | 2.3751 | 6.6523 |
| High SDI | Female | SD | 2016 | 4.4826 | 2.5489 | 7.1141 |
| High SDI | Both | SD | 2016 | 4.3082 | 2.4546 | 6.8413 |
| Low-middle SDI | Male | SD | 2015 | 3.8838 | 2.2144 | 6.1648 |
| Low-middle SDI | Female | SD | 2015 | 3.8847 | 2.2051 | 6.1764 |
| Low-middle SDI | Both | SD | 2015 | 3.8806 | 2.2008 | 6.1631 |
| Middle SDI | Male | SD | 2014 | 3.8388 | 2.1717 | 6.1389 |
| Middle SDI | Female | SD | 2014 | 3.4054 | 1.9346 | 5.4267 |
| Middle SDI | Both | SD | 2014 | 3.6285 | 2.0620 | 5.7938 |
| Low-middle SDI | Male | SD | 2016 | 3.8908 | 2.2212 | 6.1925 |
| Low-middle SDI | Female | SD | 2016 | 3.8907 | 2.2220 | 6.1605 |
| Low-middle SDI | Both | SD | 2016 | 3.8869 | 2.2197 | 6.1648 |
| Low SDI | Male | SD | 2015 | 4.8610 | 2.7718 | 7.7163 |
| Low SDI | Female | SD | 2015 | 4.9080 | 2.8015 | 7.7848 |
| Low SDI | Both | SD | 2015 | 4.8835 | 2.7838 | 7.7654 |
| Low SDI | Male | SD | 2014 | 4.8592 | 2.7713 | 7.7292 |
| Low SDI | Female | SD | 2014 | 4.9068 | 2.7881 | 7.8360 |
| Low SDI | Both | SD | 2014 | 4.8820 | 2.7871 | 7.7860 |
| Low SDI | Male | SD | 2016 | 4.8610 | 2.7594 | 7.7798 |
| Low SDI | Female | SD | 2016 | 4.9121 | 2.7892 | 7.8244 |
| Low SDI | Both | SD | 2016 | 4.8856 | 2.7736 | 7.7825 |
| Low-middle SDI | Male | SD | 2018 | 3.8997 | 2.2160 | 6.1754 |
| Low-middle SDI | Female | SD | 2018 | 3.9002 | 2.2152 | 6.1638 |
| Low-middle SDI | Both | SD | 2018 | 3.8961 | 2.2139 | 6.1828 |
| High-middle SDI | Male | SD | 2017 | 3.4532 | 1.9697 | 5.5374 |
| High-middle SDI | Female | SD | 2017 | 3.1532 | 1.7936 | 5.0155 |
| High-middle SDI | Both | SD | 2017 | 3.2998 | 1.8748 | 5.2682 |
| Low SDI | Male | SD | 2018 | 4.8667 | 2.7724 | 7.7014 |
| Low SDI | Female | SD | 2018 | 4.9122 | 2.7965 | 7.8585 |
| Low SDI | Both | SD | 2018 | 4.8885 | 2.7846 | 7.7891 |
| High SDI | Male | SD | 2017 | 4.1833 | 2.3832 | 6.6635 |
| High SDI | Female | SD | 2017 | 4.4835 | 2.5491 | 7.1422 |
| High SDI | Both | SD | 2017 | 4.3080 | 2.4555 | 6.8540 |
| Low-middle SDI | Male | SD | 2017 | 3.8960 | 2.2219 | 6.1546 |
| Low-middle SDI | Female | SD | 2017 | 3.8951 | 2.2194 | 6.1706 |
| Low-middle SDI | Both | SD | 2017 | 3.8917 | 2.2147 | 6.1775 |
| Low SDI | Male | SD | 2017 | 4.8656 | 2.7772 | 7.7545 |
| Low SDI | Female | SD | 2017 | 4.9113 | 2.8147 | 7.8265 |
| Low SDI | Both | SD | 2017 | 4.8875 | 2.7942 | 7.8020 |
| High-middle SDI | Male | SD | 2018 | 3.4487 | 1.9455 | 5.5304 |
| High-middle SDI | Female | SD | 2018 | 3.1520 | 1.7918 | 5.0053 |
| High-middle SDI | Both | SD | 2018 | 3.2972 | 1.8681 | 5.2503 |
| Middle SDI | Male | SD | 2017 | 3.8485 | 2.1734 | 6.1236 |
| Middle SDI | Female | SD | 2017 | 3.4062 | 1.9298 | 5.4347 |
| Middle SDI | Both | SD | 2017 | 3.6341 | 2.0673 | 5.7741 |
| Middle SDI | Male | SD | 2018 | 3.8521 | 2.1854 | 6.1376 |
| Middle SDI | Female | SD | 2018 | 3.4061 | 1.9227 | 5.3917 |
| Middle SDI | Both | SD | 2018 | 3.6359 | 2.0564 | 5.7669 |
| High SDI | Male | SD | 2018 | 4.1822 | 2.3681 | 6.6754 |
| High SDI | Female | SD | 2018 | 4.4807 | 2.5552 | 7.1059 |
| High SDI | Both | SD | 2018 | 4.3065 | 2.4471 | 6.8320 |
| Middle SDI | Male | SD | 2019 | 3.8577 | 2.1912 | 6.2309 |
| Middle SDI | Female | SD | 2019 | 3.4056 | 1.9373 | 5.4438 |
| Middle SDI | Both | SD | 2019 | 3.6386 | 2.0704 | 5.7855 |
| High-middle SDI | Male | SD | 2019 | 3.4466 | 1.9573 | 5.5247 |
| High-middle SDI | Female | SD | 2019 | 3.1563 | 1.7966 | 5.0342 |
| High-middle SDI | Both | SD | 2019 | 3.2985 | 1.8789 | 5.2839 |
| High SDI | Male | SD | 2019 | 4.1805 | 2.3802 | 6.6599 |
| High SDI | Female | SD | 2019 | 4.4796 | 2.5574 | 7.1147 |
| High SDI | Both | SD | 2019 | 4.3053 | 2.4548 | 6.8467 |
| Low-middle SDI | Male | SD | 2019 | 3.9031 | 2.2224 | 6.2145 |
| Low-middle SDI | Female | SD | 2019 | 3.9063 | 2.2281 | 6.2122 |
| Low-middle SDI | Both | SD | 2019 | 3.9008 | 2.2224 | 6.2109 |
| Low SDI | Male | SD | 2019 | 4.8714 | 2.7956 | 7.7075 |
| Low SDI | Female | SD | 2019 | 4.9136 | 2.8051 | 7.8325 |
| Low SDI | Both | SD | 2019 | 4.8916 | 2.7974 | 7.7673 |
|  |  |  |  |  |  |  |

**Table24 Incidence of AD in 2019, Page 125-129**

| **location_name** | **sex_name** | **cause_name** | **year** | **Age-standardised incidence rate (per 100 000 person-years)** | **95%CI(lower)** | **95%CI(upper)** |
| --- | --- | --- | --- | --- | --- | --- |
| Tonga | Both | AD | 2019 | 1692.9119 | 1555.8635 | 1828.2738 |
| Serbia | Both | AD | 2019 | 1104.5165 | 1022.2936 | 1195.4304 |
| China | Both | AD | 2019 | 1729.2000 | 1590.5943 | 1862.5152 |
| Taiwan (Province of China) | Both | AD | 2019 | 1694.6455 | 1557.2915 | 1828.0503 |
| Vanuatu | Both | AD | 2019 | 1693.9032 | 1557.0979 | 1828.6906 |
| Thailand | Both | AD | 2019 | 1693.6485 | 1556.8433 | 1828.1278 |
| Democratic People's Republic of Korea | Both | AD | 2019 | 1695.7453 | 1558.9878 | 1828.3272 |
| Slovakia | Both | AD | 2019 | 1104.4473 | 1022.1627 | 1195.5998 |
| Belarus | Both | AD | 2019 | 1073.5128 | 989.3921 | 1157.9332 |
| Cambodia | Both | AD | 2019 | 1693.4952 | 1556.3496 | 1827.6615 |
| Slovenia | Both | AD | 2019 | 1105.0762 | 1022.8469 | 1196.2461 |
| Armenia | Both | AD | 2019 | 1073.2925 | 989.1194 | 1157.4991 |
| Bulgaria | Both | AD | 2019 | 1104.7050 | 1022.3479 | 1195.7874 |
| Indonesia | Both | AD | 2019 | 1728.2502 | 1590.2738 | 1860.7135 |
| Viet Nam | Both | AD | 2019 | 1694.3651 | 1557.3319 | 1827.8863 |
| Georgia | Both | AD | 2019 | 1073.8872 | 989.7092 | 1158.4778 |
| Estonia | Both | AD | 2019 | 1074.2814 | 990.1880 | 1159.0292 |
| Timor-Leste | Both | AD | 2019 | 1694.2060 | 1557.3734 | 1828.7047 |
| Uzbekistan | Both | AD | 2019 | 1073.7168 | 989.7595 | 1158.1253 |
| Azerbaijan | Both | AD | 2019 | 1073.9981 | 989.8831 | 1158.4595 |
| Croatia | Both | AD | 2019 | 1104.3965 | 1022.1429 | 1195.4565 |
| Bhutan | Both | AD | 2019 | 1882.7711 | 1731.8369 | 2038.1987 |
| Albania | Both | AD | 2019 | 1104.3962 | 1022.1161 | 1194.6432 |
| Guyana | Both | AD | 2019 | 1693.3909 | 1556.3717 | 1828.4166 |
| Fiji | Both | AD | 2019 | 1694.9187 | 1557.6002 | 1828.4013 |
| Philippines | Both | AD | 2019 | 1728.6626 | 1590.6990 | 1861.3615 |
| Canada | Both | AD | 2019 | 1973.6606 | 1816.4143 | 2133.8753 |
| Costa Rica | Both | AD | 2019 | 1692.5461 | 1555.3977 | 1828.1245 |
| United Republic of Tanzania | Both | AD | 2019 | 2421.7921 | 2212.2002 | 2630.5696 |
| Oman | Both | AD | 2019 | 1656.2443 | 1522.8851 | 1789.9236 |
| USA | Both | AD | 2019 | 2115.7534 | 1950.4111 | 2281.6797 |
| Singapore | Both | AD | 2019 | 1929.9504 | 1763.9757 | 2097.1422 |
| Algeria | Both | AD | 2019 | 1638.7296 | 1509.3167 | 1768.4636 |
| Qatar | Both | AD | 2019 | 1686.2295 | 1547.6265 | 1824.1901 |
| Mauritania | Both | AD | 2019 | 2594.0973 | 2380.0765 | 2817.5409 |
| India | Both | AD | 2019 | 1694.8708 | 1558.9417 | 1837.4463 |
| Uganda | Both | AD | 2019 | 2421.6050 | 2212.7092 | 2630.1979 |
| Australia | Both | AD | 2019 | 1933.4201 | 1766.5413 | 2103.7985 |
| Nepal | Both | AD | 2019 | 2071.5634 | 1901.9631 | 2238.8382 |
| Kiribati | Both | AD | 2019 | 1692.6109 | 1555.2329 | 1827.7776 |
| Niger | Both | AD | 2019 | 2593.8356 | 2378.8799 | 2817.8407 |
| Saudi Arabia | Both | AD | 2019 | 1661.2417 | 1529.6304 | 1793.8482 |
| Bosnia and Herzegovina | Both | AD | 2019 | 1104.1858 | 1021.9096 | 1195.0972 |
| Chile | Both | AD | 2019 | 1932.9642 | 1766.1575 | 2103.2574 |
| Argentina | Both | AD | 2019 | 1935.0472 | 1768.0040 | 2105.5874 |
| Marshall Islands | Both | AD | 2019 | 1695.0685 | 1557.5665 | 1828.3764 |
| Comoros | Both | AD | 2019 | 2423.2035 | 2215.6182 | 2630.7035 |
| Zambia | Both | AD | 2019 | 2423.4187 | 2214.3789 | 2631.5257 |
| Antigua and Barbuda | Both | AD | 2019 | 1692.9354 | 1555.8014 | 1828.3217 |
| Brunei Darussalam | Both | AD | 2019 | 1921.1680 | 1756.5191 | 2088.1030 |
| Bahrain | Both | AD | 2019 | 1647.8830 | 1515.6537 | 1778.7557 |
| Sri Lanka | Both | AD | 2019 | 1692.7816 | 1555.5445 | 1828.2324 |
| Japan | Both | AD | 2019 | 1969.0673 | 1808.6594 | 2133.9656 |
| Egypt | Both | AD | 2019 | 1689.5550 | 1551.1761 | 1825.8499 |
| Haiti | Both | AD | 2019 | 1691.8205 | 1554.5366 | 1827.1824 |
| Israel | Both | AD | 2019 | 2011.7756 | 1848.1364 | 2173.8571 |
| France | Both | AD | 2019 | 2013.9908 | 1849.7845 | 2176.2811 |
| New Zealand | Both | AD | 2019 | 1976.7832 | 1815.5108 | 2143.2157 |
| Saint Lucia | Both | AD | 2019 | 1694.0354 | 1557.1070 | 1828.4309 |
| Greece | Both | AD | 2019 | 2013.1662 | 1849.4788 | 2175.4285 |
| Italy | Both | AD | 2019 | 2054.4733 | 1880.9977 | 2225.0134 |
| Papua New Guinea | Both | AD | 2019 | 1695.0350 | 1557.5259 | 1828.7138 |
| Denmark | Both | AD | 2019 | 2010.1577 | 1847.0087 | 2172.0521 |
| Pakistan | Both | AD | 2019 | 1914.9556 | 1767.1706 | 2081.1250 |
| Spain | Both | AD | 2019 | 2011.2407 | 1848.0037 | 2173.4868 |
| Germany | Both | AD | 2019 | 2005.7468 | 1843.1630 | 2166.6597 |
| Djibouti | Both | AD | 2019 | 2424.1264 | 2217.4673 | 2630.4678 |
| Hungary | Both | AD | 2019 | 1104.1641 | 1021.8670 | 1195.0955 |
| Iraq | Both | AD | 2019 | 1640.8183 | 1511.2653 | 1770.3133 |
| North Macedonia | Both | AD | 2019 | 1105.1440 | 1022.9306 | 1196.0705 |
| Tokelau | Both | AD | 2019 | 1695.1715 | 1557.4650 | 1828.4011 |
| Iceland | Both | AD | 2019 | 2007.9456 | 1845.7221 | 2169.7868 |
| Republic of Moldova | Both | AD | 2019 | 1073.8347 | 989.8302 | 1158.3624 |
| Dominican Republic | Both | AD | 2019 | 1694.4089 | 1557.2715 | 1828.3146 |
| Ethiopia | Both | AD | 2019 | 2480.0262 | 2262.6600 | 2688.4277 |
| Syrian Arab Republic | Both | AD | 2019 | 1630.7223 | 1503.2192 | 1758.6724 |
| Cabo Verde | Both | AD | 2019 | 2594.9157 | 2378.9521 | 2819.0172 |
| Sweden | Both | AD | 2019 | 2052.0856 | 1878.8256 | 2222.8540 |
| Luxembourg | Both | AD | 2019 | 2009.4926 | 1846.7717 | 2171.6290 |
| Eritrea | Both | AD | 2019 | 2423.2097 | 2215.8643 | 2630.3903 |
| Suriname | Both | AD | 2019 | 1693.6316 | 1556.8678 | 1828.2881 |
| El Salvador | Both | AD | 2019 | 1690.4782 | 1552.9168 | 1826.2806 |
| Norway | Both | AD | 2019 | 1859.3221 | 1712.0414 | 2007.6484 |
| Republic of Korea | Both | AD | 2019 | 1924.0915 | 1759.0148 | 2091.4836 |
| Portugal | Both | AD | 2019 | 2015.5648 | 1851.2403 | 2177.6322 |
| Tajikistan | Both | AD | 2019 | 1074.1582 | 990.1110 | 1158.8299 |
| Saint Vincent and the Grenadines | Both | AD | 2019 | 1694.7099 | 1557.4239 | 1828.8460 |
| Sao Tome and Principe | Both | AD | 2019 | 2594.9042 | 2379.7356 | 2818.3598 |
| San Marino | Both | AD | 2019 | 2022.4440 | 1857.3575 | 2184.9514 |
| Austria | Both | AD | 2019 | 2009.1352 | 1845.8745 | 2170.5915 |
| Tuvalu | Both | AD | 2019 | 1696.7332 | 1559.8087 | 1829.8360 |
| Finland | Both | AD | 2019 | 2008.6030 | 1846.1908 | 2170.5455 |
| Iran (Islamic Republic of) | Both | AD | 2019 | 1666.1923 | 1534.7476 | 1808.3804 |
| C么te d'Ivoire | Both | AD | 2019 | 2595.0074 | 2379.7217 | 2817.4871 |
| Chad | Both | AD | 2019 | 2594.6414 | 2380.7775 | 2816.3256 |
| Uruguay | Both | AD | 2019 | 1935.5590 | 1768.4212 | 2106.8019 |
| Cameroon | Both | AD | 2019 | 2632.5873 | 2413.3488 | 2853.3085 |
| Bahamas | Both | AD | 2019 | 1692.7137 | 1555.4048 | 1828.0514 |
| Nigeria | Both | AD | 2019 | 2653.9613 | 2416.1582 | 2887.1327 |
| Micronesia (Federated States of) | Both | AD | 2019 | 1694.9926 | 1557.7357 | 1828.2702 |
| Czechia | Both | AD | 2019 | 1104.7274 | 1022.4597 | 1195.8685 |
| Cyprus | Both | AD | 2019 | 2013.1666 | 1849.1236 | 2173.9646 |
| Kazakhstan | Both | AD | 2019 | 1073.1836 | 989.2014 | 1157.3785 |
| Jamaica | Both | AD | 2019 | 1693.5450 | 1556.6283 | 1828.5310 |
| Kenya | Both | AD | 2019 | 2479.8451 | 2262.2560 | 2688.7669 |
| Belgium | Both | AD | 2019 | 2011.9485 | 1848.6492 | 2174.2992 |
| Paraguay | Both | AD | 2019 | 1694.6568 | 1557.3680 | 1828.2937 |
| Gambia | Both | AD | 2019 | 2594.7628 | 2380.5434 | 2817.3223 |
| Jordan | Both | AD | 2019 | 1646.8288 | 1515.7134 | 1776.9310 |
| Andorra | Both | AD | 2019 | 2008.0193 | 1845.7366 | 2169.6813 |
| Switzerland | Both | AD | 2019 | 2009.0401 | 1846.3046 | 2170.7420 |
| Turkmenistan | Both | AD | 2019 | 1075.1455 | 991.1202 | 1160.2889 |
| Grenada | Both | AD | 2019 | 1695.4464 | 1558.0387 | 1828.8090 |
| Kyrgyzstan | Both | AD | 2019 | 1073.5642 | 989.6310 | 1157.9425 |
| Madagascar | Both | AD | 2019 | 2422.7028 | 2214.8330 | 2630.4026 |
| Latvia | Both | AD | 2019 | 1073.7809 | 989.7178 | 1158.3788 |
| Ireland | Both | AD | 2019 | 2015.1661 | 1850.8237 | 2177.6220 |
| Kuwait | Both | AD | 2019 | 1643.4325 | 1513.6274 | 1772.5694 |
| United Kingdom | Both | AD | 2019 | 2056.1472 | 1885.6361 | 2223.0077 |
| Barbados | Both | AD | 2019 | 1693.1084 | 1556.1137 | 1828.0674 |
| Mongolia | Both | AD | 2019 | 1073.5127 | 989.5654 | 1157.9091 |
| Guatemala | Both | AD | 2019 | 1691.7233 | 1554.1819 | 1827.4463 |
| Lithuania | Both | AD | 2019 | 1073.6700 | 989.5738 | 1158.1417 |
| Lao People's Democratic Republic | Both | AD | 2019 | 1694.1576 | 1557.1330 | 1828.3236 |
| Burundi | Both | AD | 2019 | 2423.8710 | 2214.5302 | 2631.8630 |
| Samoa | Both | AD | 2019 | 1695.3984 | 1557.9609 | 1828.6475 |
| Malawi | Both | AD | 2019 | 2422.4767 | 2213.1205 | 2630.2258 |
| Myanmar | Both | AD | 2019 | 1692.2191 | 1554.7682 | 1827.8638 |
| Lebanon | Both | AD | 2019 | 1647.3728 | 1517.6068 | 1777.6148 |
| Senegal | Both | AD | 2019 | 2593.0030 | 2377.7586 | 2816.6547 |
| Belize | Both | AD | 2019 | 1693.2543 | 1556.5379 | 1828.5164 |
| Palestine | Both | AD | 2019 | 1639.5213 | 1510.0012 | 1769.1060 |
| Bangladesh | Both | AD | 2019 | 1887.2409 | 1736.6636 | 2042.2330 |
| Solomon Islands | Both | AD | 2019 | 1694.2399 | 1557.1353 | 1828.5888 |
| Sierra Leone | Both | AD | 2019 | 2595.1714 | 2380.5836 | 2817.7996 |
| Honduras | Both | AD | 2019 | 1691.8194 | 1554.6257 | 1827.2965 |
| Montenegro | Both | AD | 2019 | 1104.3319 | 1022.0132 | 1195.0563 |
| Palau | Both | AD | 2019 | 1700.2834 | 1562.9604 | 1834.3224 |
| Somalia | Both | AD | 2019 | 2423.0306 | 2216.3140 | 2630.3414 |
| Malaysia | Both | AD | 2019 | 1695.9700 | 1558.7828 | 1829.2015 |
| Benin | Both | AD | 2019 | 2594.1156 | 2379.9215 | 2817.7929 |
| Russian Federation | Both | AD | 2019 | 1094.8001 | 1016.2960 | 1178.6773 |
| Tunisia | Both | AD | 2019 | 1641.9066 | 1512.8628 | 1771.5917 |
| Guam | Both | AD | 2019 | 1695.9605 | 1558.2554 | 1829.3080 |
| United States Virgin Islands | Both | AD | 2019 | 1692.0753 | 1554.6390 | 1827.5758 |
| Ukraine | Both | AD | 2019 | 1094.7195 | 1016.1205 | 1178.5263 |
| Monaco | Both | AD | 2019 | 2015.4917 | 1850.9010 | 2177.3658 |
| Angola | Both | AD | 2019 | 2421.7032 | 2212.5490 | 2630.1716 |
| Mexico | Both | AD | 2019 | 1726.6252 | 1588.4946 | 1859.5752 |
| Maldives | Both | AD | 2019 | 1704.4441 | 1568.5667 | 1840.3347 |
| Turkey | Both | AD | 2019 | 1640.8844 | 1511.2231 | 1770.6171 |
| Botswana | Both | AD | 2019 | 2422.5425 | 2214.3581 | 2630.3747 |
| Ecuador | Both | AD | 2019 | 1693.6382 | 1556.8741 | 1828.3004 |
| Nauru | Both | AD | 2019 | 1694.2194 | 1557.3873 | 1827.8637 |
| United Arab Emirates | Both | AD | 2019 | 1656.9106 | 1523.0669 | 1790.1348 |
| Central African Republic | Both | AD | 2019 | 2422.6519 | 2213.0994 | 2630.2785 |
| Poland | Both | AD | 2019 | 1126.8066 | 1042.9005 | 1214.2680 |
| Nicaragua | Both | AD | 2019 | 1693.2463 | 1556.2044 | 1827.9199 |
| Burkina Faso | Both | AD | 2019 | 2593.2260 | 2378.7429 | 2816.8054 |
| Puerto Rico | Both | AD | 2019 | 1692.7820 | 1555.6224 | 1828.0468 |
| Peru | Both | AD | 2019 | 1693.9382 | 1557.0002 | 1828.2124 |
| Lesotho | Both | AD | 2019 | 2422.9446 | 2215.4220 | 2630.6112 |
| Sudan | Both | AD | 2019 | 1641.1161 | 1511.9738 | 1770.6353 |
| Trinidad and Tobago | Both | AD | 2019 | 1694.5500 | 1557.3203 | 1828.4965 |
| Congo | Both | AD | 2019 | 2423.3301 | 2214.3728 | 2631.4163 |
| Togo | Both | AD | 2019 | 2592.9484 | 2378.4904 | 2816.2286 |
| Cuba | Both | AD | 2019 | 1694.7746 | 1557.3722 | 1828.1230 |
| Yemen | Both | AD | 2019 | 1638.9856 | 1509.7566 | 1768.7056 |
| Greenland | Both | AD | 2019 | 1967.7814 | 1811.0288 | 2128.3442 |
| Saint Kitts and Nevis | Both | AD | 2019 | 1694.2099 | 1557.3880 | 1828.5973 |
| Mauritius | Both | AD | 2019 | 1694.2073 | 1557.2240 | 1828.2903 |
| Bermuda | Both | AD | 2019 | 1693.5716 | 1556.6460 | 1828.4210 |
| Namibia | Both | AD | 2019 | 2422.0613 | 2213.7330 | 2630.1194 |
| Malta | Both | AD | 2019 | 2007.4926 | 1845.2900 | 2169.3974 |
| Democratic Republic of the Congo | Both | AD | 2019 | 2423.2697 | 2215.5650 | 2630.9132 |
| Brazil | Both | AD | 2019 | 1727.0765 | 1588.9533 | 1859.8624 |
| Afghanistan | Both | AD | 2019 | 1641.2335 | 1511.3544 | 1770.7660 |
| Colombia | Both | AD | 2019 | 1693.2428 | 1556.2828 | 1827.9558 |
| Mozambique | Both | AD | 2019 | 2421.3808 | 2211.5680 | 2630.3431 |
| Rwanda | Both | AD | 2019 | 2421.6266 | 2213.6651 | 2630.2984 |
| Gabon | Both | AD | 2019 | 2422.1865 | 2212.6410 | 2630.5345 |
| South Sudan | Both | AD | 2019 | 2421.7136 | 2212.4455 | 2630.2811 |
| Romania | Both | AD | 2019 | 1112.5870 | 1028.8385 | 1197.3682 |
| Cook Islands | Both | AD | 2019 | 1691.6928 | 1554.5270 | 1827.2229 |
| Bolivia (Plurinational State of) | Both | AD | 2019 | 1693.9968 | 1557.0952 | 1828.2996 |
| Dominica | Both | AD | 2019 | 1695.0346 | 1557.5006 | 1828.8515 |
| Equatorial Guinea | Both | AD | 2019 | 2422.0819 | 2215.2939 | 2630.1325 |
| Panama | Both | AD | 2019 | 1694.3817 | 1557.2616 | 1828.3570 |
| American Samoa | Both | AD | 2019 | 1693.9967 | 1557.1074 | 1828.3692 |
| Netherlands | Both | AD | 2019 | 2011.6266 | 1848.1397 | 2173.6280 |
| Venezuela (Bolivarian Republic of) | Both | AD | 2019 | 1693.0099 | 1556.1089 | 1828.3633 |
| Libya | Both | AD | 2019 | 1640.9996 | 1510.8839 | 1770.3154 |
| South Africa | Both | AD | 2019 | 2479.5391 | 2261.5147 | 2688.2865 |
| Eswatini | Both | AD | 2019 | 2421.8323 | 2212.9148 | 2630.0351 |
| Ghana | Both | AD | 2019 | 2657.7505 | 2424.7568 | 2899.2722 |
| Morocco | Both | AD | 2019 | 1638.2715 | 1509.0835 | 1767.7610 |
| Seychelles | Both | AD | 2019 | 1697.7376 | 1560.9891 | 1831.2032 |
| Liberia | Both | AD | 2019 | 2595.1649 | 2380.5504 | 2817.4474 |
| Zimbabwe | Both | AD | 2019 | 2421.6295 | 2212.2583 | 2630.0163 |
| Guinea | Both | AD | 2019 | 2593.7187 | 2379.3807 | 2816.1903 |
| Mali | Both | AD | 2019 | 2594.4472 | 2380.7611 | 2816.7113 |
| Guinea-Bissau | Both | AD | 2019 | 2593.5897 | 2379.4727 | 2817.2032 |
| Niue | Both | AD | 2019 | 1694.7883 | 1557.4649 | 1828.4381 |
| Northern Mariana Islands | Both | AD | 2019 | 1696.1158 | 1558.8131 | 1828.4638 |
|  |  |  |  |  |  |  |

**Table25 DALYs of SD in 2019, Page 130-134**

| **location_name** | **sex_name** | **cause_name** | **year** | **Age-standardised DALYs rate (per 100 000 person-years)** | **95%CI(lower)** | **95%CI(upper)** |
| --- | --- | --- | --- | --- | --- | --- |
| Armenia | Both | SD | 2019 | 1.9226 | 1.0760 | 3.0971 |
| Belgium | Both | SD | 2019 | 4.3041 | 2.4468 | 6.8238 |
| Micronesia (Federated States of) | Both | SD | 2019 | 3.3829 | 1.8803 | 5.4254 |
| China | Both | SD | 2019 | 3.4136 | 1.9283 | 5.4862 |
| Papua New Guinea | Both | SD | 2019 | 3.3711 | 1.9329 | 5.4041 |
| Cyprus | Both | SD | 2019 | 4.3149 | 2.4128 | 6.9458 |
| Samoa | Both | SD | 2019 | 3.3833 | 1.9417 | 5.4317 |
| Philippines | Both | SD | 2019 | 3.3937 | 1.9348 | 5.4168 |
| Chile | Both | SD | 2019 | 4.0885 | 2.3230 | 6.5192 |
| Uruguay | Both | SD | 2019 | 4.0888 | 2.3262 | 6.5064 |
| Slovakia | Both | SD | 2019 | 1.9858 | 1.1354 | 3.1972 |
| Sri Lanka | Both | SD | 2019 | 3.3779 | 1.9167 | 5.4323 |
| Hungary | Both | SD | 2019 | 1.9902 | 1.1354 | 3.1790 |
| Malawi | Both | SD | 2019 | 5.3801 | 3.0718 | 8.5478 |
| Viet Nam | Both | SD | 2019 | 3.3998 | 1.9418 | 5.4375 |
| Tonga | Both | SD | 2019 | 3.3845 | 1.8935 | 5.3744 |
| Russian Federation | Both | SD | 2019 | 1.9339 | 1.0915 | 3.0562 |
| Denmark | Both | SD | 2019 | 4.3169 | 2.4276 | 6.8934 |
| Tajikistan | Both | SD | 2019 | 1.9233 | 1.0937 | 3.1010 |
| Thailand | Both | SD | 2019 | 3.3942 | 1.9183 | 5.4180 |
| Uzbekistan | Both | SD | 2019 | 1.9246 | 1.0968 | 3.1159 |
| Italy | Both | SD | 2019 | 4.3018 | 2.4375 | 6.8671 |
| Ukraine | Both | SD | 2019 | 1.9381 | 1.0891 | 3.0996 |
| North Macedonia | Both | SD | 2019 | 1.9935 | 1.1318 | 3.1956 |
| Turkmenistan | Both | SD | 2019 | 1.9267 | 1.0979 | 3.0737 |
| Bolivia (Plurinational State of) | Both | SD | 2019 | 3.3846 | 1.9197 | 5.3717 |
| Peru | Both | SD | 2019 | 3.3947 | 1.9038 | 5.4149 |
| Ecuador | Both | SD | 2019 | 3.3867 | 1.9019 | 5.3594 |
| Solomon Islands | Both | SD | 2019 | 3.3750 | 1.9228 | 5.3393 |
| New Zealand | Both | SD | 2019 | 4.0910 | 2.3149 | 6.5497 |
| Luxembourg | Both | SD | 2019 | 4.3063 | 2.4381 | 6.8441 |
| Malta | Both | SD | 2019 | 4.3100 | 2.4414 | 6.9706 |
| Kiribati | Both | SD | 2019 | 3.3611 | 1.8837 | 5.3458 |
| Albania | Both | SD | 2019 | 1.9922 | 1.1096 | 3.2163 |
| Fiji | Both | SD | 2019 | 3.3675 | 1.9166 | 5.3963 |
| Dominican Republic | Both | SD | 2019 | 3.3917 | 1.9209 | 5.3839 |
| Marshall Islands | Both | SD | 2019 | 3.3660 | 1.8933 | 5.3889 |
| Timor-Leste | Both | SD | 2019 | 3.3835 | 1.9064 | 5.3662 |
| Finland | Both | SD | 2019 | 4.3039 | 2.4294 | 6.8433 |
| Dominica | Both | SD | 2019 | 3.3801 | 1.8964 | 5.4340 |
| Central African Republic | Both | SD | 2019 | 5.3525 | 3.0400 | 8.5299 |
| C么te d'Ivoire | Both | SD | 2019 | 5.9169 | 3.3658 | 9.5658 |
| Slovenia | Both | SD | 2019 | 1.9827 | 1.1187 | 3.1539 |
| Belize | Both | SD | 2019 | 3.3903 | 1.9210 | 5.4151 |
| Lao People's Democratic Republic | Both | SD | 2019 | 3.3873 | 1.9080 | 5.3572 |
| Bosnia and Herzegovina | Both | SD | 2019 | 1.9895 | 1.1246 | 3.1911 |
| Democratic People's Republic of Korea | Both | SD | 2019 | 3.4002 | 1.9031 | 5.4664 |
| Lesotho | Both | SD | 2019 | 5.3396 | 2.9828 | 8.5972 |
| Cuba | Both | SD | 2019 | 3.3854 | 1.8927 | 5.3559 |
| Taiwan (Province of China) | Both | SD | 2019 | 3.4130 | 1.9136 | 5.4685 |
| Canada | Both | SD | 2019 | 4.3627 | 2.4325 | 7.0151 |
| Andorra | Both | SD | 2019 | 4.3198 | 2.4729 | 6.9610 |
| Sao Tome and Principe | Both | SD | 2019 | 5.9455 | 3.4044 | 9.5995 |
| Bulgaria | Both | SD | 2019 | 1.9964 | 1.1221 | 3.2120 |
| Grenada | Both | SD | 2019 | 3.3827 | 1.9273 | 5.4304 |
| Mexico | Both | SD | 2019 | 3.3855 | 1.9138 | 5.3813 |
| Kuwait | Both | SD | 2019 | 3.8050 | 2.1519 | 6.0509 |
| Congo | Both | SD | 2019 | 5.3752 | 3.0558 | 8.6739 |
| Gambia | Both | SD | 2019 | 5.9261 | 3.3783 | 9.4706 |
| USA | Both | SD | 2019 | 4.8203 | 2.7470 | 7.6188 |
| Singapore | Both | SD | 2019 | 4.1257 | 2.3482 | 6.6789 |
| Greenland | Both | SD | 2019 | 4.3495 | 2.4343 | 6.9629 |
| Croatia | Both | SD | 2019 | 1.9891 | 1.1382 | 3.1560 |
| Venezuela (Bolivarian Republic of) | Both | SD | 2019 | 3.3865 | 1.9068 | 5.3752 |
| Azerbaijan | Both | SD | 2019 | 1.9215 | 1.0767 | 3.0873 |
| Lebanon | Both | SD | 2019 | 3.8158 | 2.1476 | 6.1384 |
| Montenegro | Both | SD | 2019 | 1.9912 | 1.1308 | 3.2111 |
| Botswana | Both | SD | 2019 | 5.3451 | 3.0062 | 8.5210 |
| Nicaragua | Both | SD | 2019 | 3.3821 | 1.9264 | 5.4066 |
| Brunei Darussalam | Both | SD | 2019 | 4.0644 | 2.3274 | 6.4706 |
| Senegal | Both | SD | 2019 | 5.9138 | 3.3544 | 9.4686 |
| Malaysia | Both | SD | 2019 | 3.3934 | 1.9064 | 5.4580 |
| Guam | Both | SD | 2019 | 3.3903 | 1.8854 | 5.3899 |
| Georgia | Both | SD | 2019 | 1.9202 | 1.0675 | 3.0389 |
| Argentina | Both | SD | 2019 | 4.0970 | 2.3322 | 6.5652 |
| Cambodia | Both | SD | 2019 | 3.3895 | 1.9085 | 5.3781 |
| United Arab Emirates | Both | SD | 2019 | 3.8171 | 2.1263 | 6.0630 |
| Australia | Both | SD | 2019 | 4.0808 | 2.3195 | 6.4750 |
| Poland | Both | SD | 2019 | 2.0061 | 1.1355 | 3.1757 |
| Suriname | Both | SD | 2019 | 3.3713 | 1.9013 | 5.3072 |
| Panama | Both | SD | 2019 | 3.3903 | 1.9203 | 5.3668 |
| Japan | Both | SD | 2019 | 4.1085 | 2.3511 | 6.5435 |
| Syrian Arab Republic | Both | SD | 2019 | 3.7702 | 2.1215 | 6.0760 |
| Czechia | Both | SD | 2019 | 1.9862 | 1.1303 | 3.2261 |
| Bangladesh | Both | SD | 2019 | 4.3565 | 2.4903 | 6.9244 |
| Indonesia | Both | SD | 2019 | 3.3926 | 1.9169 | 5.3937 |
| Spain | Both | SD | 2019 | 4.3173 | 2.4589 | 6.9987 |
| Austria | Both | SD | 2019 | 4.3009 | 2.4147 | 6.9000 |
| Yemen | Both | SD | 2019 | 3.7807 | 2.1287 | 6.0721 |
| Libya | Both | SD | 2019 | 3.7842 | 2.1218 | 6.1627 |
| Romania | Both | SD | 2019 | 2.0234 | 1.1374 | 3.1929 |
| Colombia | Both | SD | 2019 | 3.3992 | 1.9033 | 5.4712 |
| Sierra Leone | Both | SD | 2019 | 5.9299 | 3.3221 | 9.4058 |
| Maldives | Both | SD | 2019 | 3.4089 | 1.9342 | 5.4040 |
| Tunisia | Both | SD | 2019 | 3.8136 | 2.1425 | 6.1995 |
| Brazil | Both | SD | 2019 | 3.3857 | 1.9174 | 5.4337 |
| Republic of Korea | Both | SD | 2019 | 4.0878 | 2.3240 | 6.5104 |
| San Marino | Both | SD | 2019 | 4.3443 | 2.4658 | 6.9841 |
| Trinidad and Tobago | Both | SD | 2019 | 3.3892 | 1.9050 | 5.4047 |
| Puerto Rico | Both | SD | 2019 | 3.3864 | 1.8991 | 5.3952 |
| Sweden | Both | SD | 2019 | 4.3119 | 2.4491 | 6.8033 |
| Mauritius | Both | SD | 2019 | 3.3772 | 1.9023 | 5.3215 |
| Netherlands | Both | SD | 2019 | 4.3252 | 2.4469 | 6.9074 |
| Honduras | Both | SD | 2019 | 3.3774 | 1.9148 | 5.3829 |
| Latvia | Both | SD | 2019 | 1.9210 | 1.0726 | 3.0573 |
| Costa Rica | Both | SD | 2019 | 3.3839 | 1.9472 | 5.3541 |
| Kazakhstan | Both | SD | 2019 | 1.9167 | 1.0898 | 3.0544 |
| Turkey | Both | SD | 2019 | 3.7951 | 2.1597 | 6.0302 |
| Switzerland | Both | SD | 2019 | 4.3089 | 2.4491 | 6.8982 |
| Equatorial Guinea | Both | SD | 2019 | 5.3651 | 3.0342 | 8.4718 |
| Myanmar | Both | SD | 2019 | 3.3746 | 1.9220 | 5.3884 |
| Saint Kitts and Nevis | Both | SD | 2019 | 3.3831 | 1.9090 | 5.4224 |
| Namibia | Both | SD | 2019 | 5.3625 | 3.0414 | 8.6473 |
| Mozambique | Both | SD | 2019 | 5.3529 | 3.0356 | 8.4687 |
| Lithuania | Both | SD | 2019 | 1.9166 | 1.0897 | 3.0555 |
| Tokelau | Both | SD | 2019 | 3.3801 | 1.8610 | 5.3362 |
| Belarus | Both | SD | 2019 | 1.9155 | 1.0755 | 3.0794 |
| Iran (Islamic Republic of) | Both | SD | 2019 | 3.7625 | 2.1315 | 6.0438 |
| Ghana | Both | SD | 2019 | 6.2480 | 3.5740 | 9.8433 |
| Norway | Both | SD | 2019 | 3.6610 | 2.0929 | 5.7932 |
| Djibouti | Both | SD | 2019 | 5.3931 | 3.0378 | 8.6840 |
| Republic of Moldova | Both | SD | 2019 | 1.9119 | 1.0725 | 3.0522 |
| Rwanda | Both | SD | 2019 | 5.3876 | 3.0511 | 8.6279 |
| Kyrgyzstan | Both | SD | 2019 | 1.9226 | 1.0932 | 3.0498 |
| South Africa | Both | SD | 2019 | 5.3325 | 3.0344 | 8.4987 |
| Estonia | Both | SD | 2019 | 1.9180 | 1.0831 | 3.0502 |
| Guinea | Both | SD | 2019 | 5.9299 | 3.3797 | 9.4574 |
| Gabon | Both | SD | 2019 | 5.3681 | 3.0660 | 8.6693 |
| Qatar | Both | SD | 2019 | 3.8860 | 2.1852 | 6.2772 |
| Iraq | Both | SD | 2019 | 3.7940 | 2.1266 | 6.1298 |
| Tuvalu | Both | SD | 2019 | 3.3802 | 1.9259 | 5.4916 |
| Bahrain | Both | SD | 2019 | 3.7983 | 2.1617 | 6.1443 |
| Eritrea | Both | SD | 2019 | 5.3769 | 3.0553 | 8.6554 |
| Kenya | Both | SD | 2019 | 5.3629 | 3.0563 | 8.5404 |
| Portugal | Both | SD | 2019 | 4.3193 | 2.4385 | 6.9602 |
| Guinea-Bissau | Both | SD | 2019 | 5.9177 | 3.3639 | 9.3499 |
| Egypt | Both | SD | 2019 | 4.1901 | 2.3935 | 6.8721 |
| Saudi Arabia | Both | SD | 2019 | 3.8538 | 2.1728 | 6.2162 |
| Eswatini | Both | SD | 2019 | 5.3389 | 3.0370 | 8.5402 |
| Jordan | Both | SD | 2019 | 3.8184 | 2.1858 | 6.2037 |
| United Kingdom | Both | SD | 2019 | 4.3269 | 2.4738 | 6.8991 |
| Togo | Both | SD | 2019 | 5.9253 | 3.3955 | 9.3892 |
| Madagascar | Both | SD | 2019 | 5.3941 | 3.0412 | 8.6560 |
| Ethiopia | Both | SD | 2019 | 5.3604 | 3.0350 | 8.5014 |
| Mongolia | Both | SD | 2019 | 1.9224 | 1.0726 | 3.0346 |
| Serbia | Both | SD | 2019 | 1.9858 | 1.1286 | 3.2372 |
| Burkina Faso | Both | SD | 2019 | 5.9245 | 3.3585 | 9.4853 |
| Bhutan | Both | SD | 2019 | 4.3264 | 2.4629 | 6.9601 |
| Guyana | Both | SD | 2019 | 3.3627 | 1.9369 | 5.1972 |
| American Samoa | Both | SD | 2019 | 3.3652 | 1.9077 | 5.3465 |
| Cameroon | Both | SD | 2019 | 6.0297 | 3.3872 | 9.5408 |
| France | Both | SD | 2019 | 4.3262 | 2.4687 | 6.9096 |
| Vanuatu | Both | SD | 2019 | 3.3756 | 1.8902 | 5.3244 |
| Pakistan | Both | SD | 2019 | 4.3109 | 2.4621 | 6.8903 |
| India | Both | SD | 2019 | 3.6167 | 2.0768 | 5.7359 |
| Cabo Verde | Both | SD | 2019 | 5.9553 | 3.3509 | 9.4874 |
| Haiti | Both | SD | 2019 | 3.3656 | 1.9019 | 5.3472 |
| Burundi | Both | SD | 2019 | 5.3688 | 3.0523 | 8.4701 |
| Germany | Both | SD | 2019 | 4.2932 | 2.4563 | 6.9239 |
| Nepal | Both | SD | 2019 | 5.1216 | 2.8751 | 8.0755 |
| Seychelles | Both | SD | 2019 | 3.3866 | 1.9409 | 5.3606 |
| Barbados | Both | SD | 2019 | 3.3913 | 1.9247 | 5.4367 |
| Saint Lucia | Both | SD | 2019 | 3.3860 | 1.8955 | 5.4141 |
| Comoros | Both | SD | 2019 | 5.3978 | 3.0515 | 8.6606 |
| Greece | Both | SD | 2019 | 4.3232 | 2.4382 | 6.9406 |
| Jamaica | Both | SD | 2019 | 3.3895 | 1.9333 | 5.4010 |
| Somalia | Both | SD | 2019 | 5.3710 | 3.0303 | 8.5118 |
| Bermuda | Both | SD | 2019 | 3.3955 | 1.9079 | 5.4445 |
| United States Virgin Islands | Both | SD | 2019 | 3.3870 | 1.9255 | 5.4424 |
| Saint Vincent and the Grenadines | Both | SD | 2019 | 3.3824 | 1.9477 | 5.3714 |
| Monaco | Both | SD | 2019 | 4.3254 | 2.4437 | 6.9681 |
| Angola | Both | SD | 2019 | 5.3802 | 3.0359 | 8.6351 |
| Cook Islands | Both | SD | 2019 | 3.3757 | 1.8914 | 5.3870 |
| United Republic of Tanzania | Both | SD | 2019 | 5.3814 | 3.0630 | 8.6109 |
| Paraguay | Both | SD | 2019 | 3.3885 | 1.9098 | 5.4978 |
| Antigua and Barbuda | Both | SD | 2019 | 3.3807 | 1.8912 | 5.3487 |
| Chad | Both | SD | 2019 | 5.9233 | 3.3174 | 9.4229 |
| Iceland | Both | SD | 2019 | 4.3146 | 2.4682 | 6.8885 |
| Bahamas | Both | SD | 2019 | 3.3859 | 1.9043 | 5.3634 |
| El Salvador | Both | SD | 2019 | 3.3752 | 1.8807 | 5.3445 |
| Afghanistan | Both | SD | 2019 | 3.7672 | 2.0788 | 6.0004 |
| Algeria | Both | SD | 2019 | 3.8014 | 2.1209 | 6.1213 |
| Guatemala | Both | SD | 2019 | 3.3723 | 1.9051 | 5.3322 |
| Democratic Republic of the Congo | Both | SD | 2019 | 5.3651 | 3.0229 | 8.6222 |
| Israel | Both | SD | 2019 | 4.3233 | 2.4679 | 7.0360 |
| Niue | Both | SD | 2019 | 3.3728 | 1.9040 | 5.4260 |
| Uganda | Both | SD | 2019 | 5.3779 | 3.0237 | 8.5120 |
| Mali | Both | SD | 2019 | 5.9230 | 3.4117 | 9.5425 |
| Zimbabwe | Both | SD | 2019 | 5.3731 | 3.1133 | 8.5137 |
| Ireland | Both | SD | 2019 | 4.3195 | 2.4526 | 6.8489 |
| Morocco | Both | SD | 2019 | 3.7950 | 2.1449 | 6.0593 |
| Sudan | Both | SD | 2019 | 3.7933 | 2.1125 | 6.1118 |
| Liberia | Both | SD | 2019 | 5.8791 | 3.3511 | 9.4467 |
| Northern Mariana Islands | Both | SD | 2019 | 3.3935 | 1.8994 | 5.4310 |
| Oman | Both | SD | 2019 | 3.8162 | 2.1397 | 6.1695 |
| Nauru | Both | SD | 2019 | 3.3748 | 1.9243 | 5.3821 |
| Mauritania | Both | SD | 2019 | 5.9421 | 3.3917 | 9.4392 |
| Nigeria | Both | SD | 2019 | 5.8928 | 3.3708 | 9.4092 |
| Palestine | Both | SD | 2019 | 3.7853 | 2.1116 | 6.1124 |
| Zambia | Both | SD | 2019 | 5.3731 | 3.0697 | 8.5609 |
| Benin | Both | SD | 2019 | 5.9368 | 3.3996 | 9.5443 |
| Niger | Both | SD | 2019 | 5.9302 | 3.3880 | 9.3996 |
| South Sudan | Both | SD | 2019 | 5.3344 | 3.0239 | 8.5336 |
| Palau | Both | SD | 2019 | 3.3778 | 1.9007 | 5.3721 |
|  |  |  |  |  |  |  |

| **Table 26 The age-standardized incidence rate of major dermatitis and DALYs combined with different factors (global, different SDI, gender) summarized results in 2019 (or 1990-2019), Page 135-138** | | | | | | | |
| --- | --- | --- | --- | --- | --- | --- | --- |
| **Group** | | **Dermatitis** | | | | | |
|  |  | **Incidence** | | | **DALY** | | |
|  |  | **Age-standardized rates per 100,000 persons** | | **percent change** | **Age-standardized rates per 100,000 persons** | | **percent change** |
| **SDI** | **Sex** | **1990 (95% CI)** | **2019 (95% CI)** | **1990-2019 (95% CI)** | **1990 (95% CI)** | **2019 (95% CI)** | **1990-2019 (95% CI)** |
| **High SDI** | **Male** | 4477.3337(3976.2427-5023.4508) | 4340.5091(3855.7501-4857.0321) | -0.0306(-0.0366- -0.0245) | 163.0948(93.2303-262.3855) | 156.3118(89.0274-251.4453) | -0.0416(-0.0536- -0.0296) |
|  | **Female** | 5628.0682(4972.6651-6370.4362) | 5420.0704(4807.0541-6109.6649) | -0.0370(-0.0433- -0.0303) | 223.1691(127.4644-359.5248) | 214.4789(122.0239-345.8437) | -0.0389(-0.049- -0.0290) |
|  | **Both** | 5046.3757(4466.2034-5673.5182) | 4866.7872(4318.0660-5460.9921) | -0.0356(-0.0412- -0.0300) | 192.7076(109.7712-309.8751) | 184.6675(105.1112-296.7526) | -0.0417(-0.0500- -0.0344) |
| **High-middle SDI** | **Male** | 4500.9508(3899.8675-5093.0252) | 4636.5478(4014.9118-5262.7968) | 0.0301(0.0253-0.0347) | 119.0335(69.1019-188.1784) | 123.9517(72.3622-196.3601) | 0.0413(0.0277-0.0548) |
|  | **Female** | 5435.7614(4692.9467-6236.4584) | 5495.3191(4754.1364-6287.2131) | 0.0110(0.0054-0.0167) | 173.5118(100.9699-274.4364) | 181.0486(103.7650-287.7947) | 0.0434(0.0309-0.0566) |
|  | **Both** | 4971.2820(4305.3316-5674.6034) | 5062.2407(4385.5198-5775.7678) | 0.0183(0.0143-0.0224) | 146.0393(84.8814-231.1693) | 151.8287(87.8332-240.9907) | 0.0396(0.0301-0.0489) |
| **middle SDI** | **Male** | 5287.7358(4543.4658-6051.8570) | 4878.2380(4194.9066-5587.6487) | -0.0774(-0.0820- -0.0724) | 112.0035(66.7097-174.5114) | 111.7931(66.5380-174.4910) | -0.0019(-0.0140-0.0109) |
|  | **Female** | 5840.8361(5021.5293- 6719.0913) | 5794.0060(4976.6459-6670.1422) | -0.0080(-0.0112- -0.0048) | 166.0383(97.6460-260.1469) | 162.2455(94.9729-254.4554) | -0.0228(-0.0345- -0.0116) |
|  | **Both** | 5564.3414(4780.5437- 6379.7120) | 5342.6488(4593.5414-6130.5969) | -0.0398(-0.0426- -0.0370) | 138.6636(82.0927-215.9783) | 136.9152(80.8024-214.2455) | -0.0126(-0.0209--0.0046) |
| **Low-middle SDI** | **Male** | 4976.2799(4291.2940-5685.5591) | 4864.8082(4196.6223-5551.3850) | -0.0224(-0.0242- -0.0204) | 105.4405(62.2467-164.0893) | 104.2855(61.8166-162.1900) | -0.0110(-0.0243-0.0021) |
|  | **Female** | 5537.4938(4759.9388-6358.3086) | 5543.4592(4772.3364-6355.4757) | 0.0011(-0.0013-0.0037) | 130.2445(76.8261-203.7177) | 127.4410(75.4290-198.7598) | -0.0215(-0.0333- -0.0104) |
|  | **Both** | 5253.0633(4527.7777-6017.2760) | 5207.3377(4489.5443-5958.8762) | -0.0087(-0.0105- -0.0069) | 117.6496(69.5722-182.8915) | 115.8810(68.7472-180.1714) | -0.0150(-0.0249- -0.0064) |
| **Low SDI** | **Male** | 5099.0749(4452.0643-5767.1052) | 4596.2117(4009.7198-5193.1840) | -0.0986(-0.1001- -0.0969) | 85.5967(51.4878-133.0171) | 84.3620(50.7086-130.2210) | -0.0144(-0.0294- -0.0011) |
|  | **Female** | 5539.8360(4803.8073-6287.3009) | 5539.6967(4806.2176-6283.3834) | 0.0000(-0.0015-0.0013) | 97.8127(58.3475-152.5018) | 97.2390(58.1133-151.8858) | -0.0059(-0.0188-0.0077) |
|  | **Both** | 5318.7527(4632.8787-6025.8978) | 5072.0282(4418.7512-5746.4829) | -0.0464(-0.0476- -0.0451) | 91.6566(54.7499-142.0591) | 90.8022(54.2303-140.8034) | -0.0093(-0.0197-0.0011) |
| **Global** | **Male** | 4855.0107(4210.2211-5512.0138） | 4924.0097(4270.5610-5599.5590) | 0.0142(0.0108-0.0175) | 114.1767(67.2878-178.8987) | 111.8410(66.2202-174.9826) | -0.0205(-0.0286- -0.0124) |
|  | **Female** | 5556.6251(4814.5261-6358.0443） | 5563.2498(4818.0381-6368.6391) | 0.0012(-0.0029-0.0056) | 157.9989(92.6894-248.9179) | 151.7910(89.0931-239.0627) | -0.0393(-0.0472- -0.0314) |
|  | **Both** | 5204.4291(4517.0914-5949.2963） | 5244.3988(4551.7244-5979.3176) | 0.0077(0.0045-0.0111) | 135.9009(79.9488-214.0275) | 131.6711(77.5876-206.8696) | -0.0311(-0.0378- -0.0249) |
|  |  |  |  |  |  |  |  |
| **Group** | | **Atopic dermatitis** | | | | | |
|  |  | **Incidence** | | | **DALY** | | |
|  |  | **Age-standardized rates per 100,000 persons** | | **percent change** | **Age-standardized rates per 100,000 persons** | | **percent change** |
| **SDI** | **Sex** | **1990 (95% CI)** | **2019 (95% CI)** | **1990-2019 (95% CI)** | **1990 (95% CI)** | **2019 (95% CI)** | **1990-2019 (95% CI)** |
| **High SDI** | **Male** | 321.2784(301.3824-341.3855) | 309.1893(289.6693-329.9791) | -0.0376(-0.0508--0.0237) | 135.2475(72.3893-228.6431) | 129.9786(69.5909-219.3316) | -0.0390(-0.0531- -0.0255) |
|  | **Female** | 499.8202(472.8721-527.5276) | 484.1783(457.7784-512.1446) | -0.0313(-0.0411--0.0207) | 188.9878(101.3340-316.1332) | 182.3910(98.0570-307.3115) | -0.0349(-0.0460- -0.0229) |
|  | **Both** | 409.1284(385.7456-432.8233) | 394.4542(371.5985-417.3112) | -0.0359(-0.0442--0.0277) | 161.7520(86.6560-272.4654) | 155.5406(83.4644-261.9977) | -0.0384(-0.0474- -0.0295) |
| **High-middle SDI** | **Male** | 266.6719(252.6510-281.0475) | 279.0329(264.8316-293.5968) | 0.0464(0.0338-0.0604) | 91.8325(49.1399-154.7714) | 95.4297(50.8194-159.6917) | 0.0392(0.0230-0.0555) |
|  | **Female** | 440.6767(419.3842-461.9207) | 463.4600(441.5803-485.2813) | 0.0517(0.0391-0.0640) | 139.4014(73.7746-235.2552) | 146.7301(77.9871-246.9473) | 0.0526(0.0378-0.0675) |
|  | **Both** | 352.2975(335.5211-369.9588) | 368.4836(351.0207-386.7380) | 0.0459(0.0367-0.0556) | 115.3627(61.2870-194.0445) | 120.4296(63.8046-202.1894) | 0.0439(0.0326-0.0556) |
| **middle SDI** | **Male** | 284.0483(271.2118-297.5573) | 259.9803(248.1490-272.5343) | -0.0847(-0.0974- -0.0713) | 79.1386(42.4901-132.3130) | 79.0778(42.1330-132.3027) | -0.0008(-0.0177-0.0159) |
|  | **Female** | 469.6452(448.7131-490.1584) | 446.0551(425.7193-464.7858) | -0.0502(-0.0637- -0.0371) | 129.3402(68.8542-216.4033) | 125.6938(66.9636-210.9003) | -0.0282( -0.0427--0.0144) |
|  | **Both** | 375.5650(359.5706-390.9805) | 352.1898(336.4264-367.4982) | -0.0622(-0.0717- -0.0517) | 103.8815(55.4667-173.5997) | 102.2516(54.2922-171.6548) | -0.0157( -0.0274--0.0055) |
| **Low-middle SDI** | **Male** | 260.6269(245.4221-276.7242) | 248.2918(233.8096-263.5387) | -0.0473(-0.0517- -0.0430) | 75.0453(40.0443-125.5224) | 73.8530(39.5751-123.5061) | -0.0159(-0.0323-0.0012) |
|  | **Female** | 339.6482(321.5116-358.6253) | 322.8606(305.4998-340.9221) | -0.0494(-0.0546- -0.0441) | 96.1913(51.4049-160.4714) | 93.3111(49.6542-155.4591) | -0.0299(-0.0449--0.0153) |
|  | **Both** | 299.5397(283.7929-316.4791) | 285.4254(270.3178-301.3075) | -0.0471(-0.0506- -0.0434) | 85.4521(45.6966-142.5232) | 83.5774(44.7767-139.8255) | -0.0219(-0.0338--0.0103) |
| **Low SDI** | **Male** | 189.4868(178.5749-201.9551) | 159.9655(150.8988-170.0096) | -0.1558(-0.1614- -0.1499) | 56.2466(30.5120-94.8664) | 54.8877(29.3733-91.9758) | -0.0242(-0.0440--0.0063) |
|  | **Female** | 222.2747(210.1642-235.8632) | 216.8113(205.3695-229.5318) | -0.0246(-0.0302- -0.0185) | 65.2079(35.1038-108.7454) | 64.4972(34.7426-107.7339) | -0.0109(-0.0272-0.0072) |
|  | **Both** | 205.7307(194.4640-218.1661) | 188.1815(178.0950-199.2461) | -0.0853(-0.0900- -0.0804) | 60.6871(32.6904-101.7106) | 59.6780(31.9642-100.1477) | -0.0166(-0.0302--0.0029) |
| **Global** | **Male** | 272.3400(259.0031-286.1006) | 266.6894(253.8420-280.2492) | -0.0207(-0.0283- -0.0127) | 84.5891(45.1163-142.0661) | 82.0109(43.7200-137.7186) | -0.0305(-0.0395- -0.0215) |
|  | **Female** | 413.8120(394.9024-433.1748) | 390.5420(372.4707-408.3218) | -0.0562(-0.0643- -0.0477) | 123.7267(65.7921-207.2789) | 117.6519(62.6067-197.4150) | -0.0491(-0.0578- -0.0404) |
|  | **Both** | 342.3050(327.0355-358.4175) | 327.9136(312.7599-343.6677) | -0.0420(-0.0481- -0.0358) | 103.9911(55.3169-174.5661) | 99.6852(53.0912-167.4329) | -0.0414(-0.0475- -0.0350) |
|  |  |  |  |  |  |  |  |
| **Group** | | **Contact dermatitis** | | | | | |
|  |  | **Incidence** | | | **DALY** | | |
|  |  | **Age-standardized rates per 100,000 persons** | | **percent change** | **Age-standardized rates per 100,000 persons** | | **percent change** |
| **SDI** | **Sex** | **1990 (95% CI)** | **2019 (95% CI)** | **1990-2019 (95% CI)** | **1990 (95% CI)** | **2019 (95% CI)** | **1990-2019 (95% CI)** |
| **High SDI** | **Male** | 2309.5629(1833.6548-2810.7542) | 2213.7913(1754.3458-2702.3922) | -0.0415(-0.0522- -0.0306) | 23.5734(15.1080-34.9922) | 22.1527(14.2803-32.9251) | -0.0603(-0.0800- -0.0397) |
|  | **Female** | 2945.3435(2313.8490-3629.0688) | 2809.8276(2212.4774-3452.3443) | -0.0460(-0.0569- -0.0348) | 29.5340(19.1000-43.8251) | 27.6082(17.7813-41.0198) | -0.0652(-0.0861- -0.0445) |
|  | **Both** | 2625.4592(2068.7619-3216.9210) | 2505.4542(1979.8450-3063.7478) | -0.0457(-0.0549- -0.0361) | 26.5322(17.0857-39.4403) | 24.8216(16.0310-37.0273) | -0.0645(-0.0802- -0.0479) |
| **High-middle SDI** | **Male** | 2611.7843(2047.1502-3191.7962) | 2705.5831(2114.9019-3302.5082) | 0.0359(0.0281-0.0432) | 23.8807(14.9475-35.4827) | 25.0755(15.7233-37.4380) | 0.0500(0.0280-0.0718) |
|  | **Female** | 3435.2111(2712.4927-4192.0039) | 3433.5658(2720.4812-4182.1921) | -0.0005(-0.0082-0.0080) | 31.0776(19.6504-45.9572) | 31.1622(19.7450-46.2027) | 0.0027(-0.0155-0.0199) |
|  | **Both** | 3027.5031(2385.9513-3692.8321) | 3067.7851(2417.5886-3741.9077) | 0.0133(0.0068-0.0198) | 27.5114(17.3189-40.9649) | 28.1006(17.6757-41.8087) | 0.0214(0.0066-0.0358) |
| **middle SDI** | **Male** | 3188.1593(2476.5316-3915.0785) | 2909.5856(2258.5382-3566.4022) | -0.0874(-0.0935- -0.0794) | 29.0921(18.1759-43.7703) | 28.8576(17.8901-43.5244) | -0.0081(-0.0220-0.0063) |
|  | **Female** | 3699.5362(2913.4639-4526.6603) | 3668.5758(2887.2509-4485.3101) | -0.0084(-0.0130- -0.0043) | 33.3361(21.1210-49.0917) | 33.1461(20.7863-49.3044) | -0.0057(-0.0193-0.0085) |
|  | **Both** | 3443.6662(2689.3846-4205.0973) | 3294.5941(2573.1487-4024.3112) | -0.0433(-0.0469- -0.0395) | 31.2113(19.6627-46.4362) | 31.0249(19.5010-46.1529) | -0.0060(-0.0164-0.0040) |
| **Low-middle SDI** | **Male** | 2942.3690(2287.0978-3610.2043) | 2869.1086(2232.8756-3517.4702) | -0.0249(-0.0274- -0.0221) | 26.5946(16.6811-39.5501) | 26.5294(16.6221-39.7729) | -0.0025(-0.0201-0.0159) |
|  | **Female** | 3398.5032(2666.1627-4184.6200) | 3377.6994(2650.1604-4154.3920） | -0.0061(-0.0086- -0.0033 ) | 30.2637(19.0539-44.5378) | 30.2236(19.0038-44.7158) | -0.0013(-0.0175-0.0158) |
|  | **Both** | 3167.3349(2468.3855-3895.8755) | 3127.1088(2436.7674-3839.4989) | -0.0127(-0.0149- -0.0104) | 28.4041(17.8710-42.1621) | 28.4028(17.8174-42.1527) | 0.0000(-0.0124-0.0128) |
| **Low SDI** | **Male** | 2727.3707(2130.9103-3329.1432) | 2457.8661(1921.1145-3003.2924) | -0.0988(-0.1013- -0.0962) | 24.5654(15.3519-36.6304) | 24.6029(15.3313-36.7997) | 0.0015(-0.0182-0.0216) |
|  | **Female** | 3120.0442(2445.4662-3824.6287) | 3115.7809(2442.6691-3814.8103) | -0.0014(-0.0034-0.0007) | 27.7263(17.4057-41.3211) | 27.8282(17.2965-41.4135) | 0.0037(-0.0159-0.0232) |
|  | **Both** | 2922.8883(2291.0991-3579.7625) | 2790.5367(2188.2161-3414.0224) | -0.0453(-0.0471- -0.0432) | 26.1379(16.4102-39.0012) | 26.2326(16.4093-39.1330) | 0.0036(-0.0116-0.0168) |
| **Global** | **Male** | 2774.1544(2169.1887-3395.0065) | 2802.7859(2182.8659-3426.8620) | 0.0103(0.0048-0.0151) | 25.6677(16.0898-38.1026) | 25.8156(16.0938-38.5597) | 0.0058(-0.0049-0.0164) |
|  | **Female** | 3333.5760(2626.4224-4085.6360) | 3326.8553(2618.9106-4070.2312) | -0.0020(-0.0084-0.0047) | 30.5172(19.2735-44.9576) | 30.2843(19.1718-44.8530) | -0.0076(-0.0181-0.0024) |
|  | **Both** | 3053.2513(2401.4428-3741.1057) | 3066.0421(2405.3768-3755.3806) | 0.0042(-0.0007-0.0094) | 28.0846(17.6198-41.6829) | 28.0575(17.6212-41.7765) | -0.0010(-0.0094-0.0076) |
|  |  |  | |  |  | |  |
|  |  |  |  |  |  |  |  |
| **Group** | | **Seborrhoeic dermatitis** | | | | | |
|  |  | **Incidence** | | | **DALY** | | |
|  |  | **Age-standardized rates per 100,000 persons** | | **percent change** | **Age-standardized rates per 100,000 persons** | | **percent change** |
| **SDI** | **Sex** | **1990 (95% CI)** | **2019 (95% CI)** | **1990-2019 (95% CI)** | **1990 (95% CI)** | **2019 (95% CI)** | **1990-2019 (95% CI)** |
| **High SDI** | **Male** | 1846.4924(1705.3308-1987.9326) | 1817.53(1678.63-1954.89) | -0.0157(-0.0206- -0.0112) | 4.2775(2.4412-6.8334) | 4.1805(2.3802-6.6599) | -0.0227(-0.0419- -0.0033) |
|  | **Female** | 2182.9046(2005.4739-2357.2282) | 2126.06(1956.15-2292.81) | -0.0260(-0.0305- -0.0216) | 4.6474(2.6426-7.3986) | 4.4796(2.5574-7.1147) | -0.0361(-0.0544- -0.0172) |
|  | **Both** | 2011.7882(1852.8128-2168.6014) | 1966.88(1814.87-2118.09) | -0.0223(-0.0261- -0.0187) | 4.4234(2.5162-7.0154) | 4.3053(2.4548-6.8467) | -0.0267(-0.0405- -0.0135) |
| **High-middle SDI** | **Male** | 1622.4946(1500.0818-1746.5157) | 1651.93(1526.28-1781.38) | 0.0181(0.0151-0.0209) | 3.3203(1.8733-5.3067) | 3.4466(1.9573-5.5247) | 0.0380(0.0105-0.0652) |
|  | **Female** | 1559.8736(1435.6816-1677.3380) | 1598.29(1472.47-1717.39) | 0.0246(0.0216-0.0277) | 3.0328(1.7169-4.8180) | 3.1563(1.7966-5.0342) | 0.0407(0.0142-0.0673) |
|  | **Both** | 1591.4814(1469.0632-1711.0480) | 1625.97(1500.95-1749.86) | 0.0217(0.0194-0.0240) | 3.1652(1.7980-5.0345) | 3.2985(1.8789-5.2839) | 0.0421(0.0245-0.0621) |
| **middle SDI** | **Male** | 1815.5282(1673.7405-1958.8471) | 1708.67(1577.25-1845.90) | -0.0589(-0.0634- -0.0542) | 3.7728(2.1355-6.0280) | 3.8577(2.1912-6.2309) | 0.0225(0.0025-0.0428) |
|  | **Female** | 1671.6547(1536.2508-1800.7019) | 1679.38(1545.19-1809.15) | 0.0046(0.0009-0.0087) | 3.3620(1.9081-5.3246) | 3.4056(1.9373-5.4438) | 0.0130(-0.0063-0.0317) |
|  | **Both** | 1745.1101(1607.0420-1877.4338) | 1695.86(1563.11-1825.93) | -0.0282(-0.0315- -0.0249) | 3.5709(2.0210-5.6727) | 3.6386(2.0704-5.7855) | 0.0190(0.0048-0.0322) |
| **Low-middle SDI** | **Male** | 1773.2841(1634.9859-1918.5724) | 1747.41(1612.44-1889.17) | -0.0146(-0.0169- -0.0121) | 3.8006(2.1541-6.0114) | 3.9031(2.2224-6.2145) | 0.0270(0.0021-0.0510) |
|  | **Female** | 1799.3423(1656.1549-1942.7831) | 1842.90(1696.55-1991.86) | 0.0242(0.0211-0.0272) | 3.7896(2.1576-5.9967) | 3.9063(2.2281-6.2122) | 0.0308(0.00640-0.0578) |
|  | **Both** | 1786.1887(1649.2612-1929.3157) | 1794.80(1657.86-1938.72) | 0.0048(0.0026-0.0069) | 3.7934(2.1545-6.0064) | 3.9008(2.2224-6.2109) | 0.0283(0.0113-0.0456) |
| **Low SDI** | **Male** | 2182.2174(2010.3481-2365.3859) | 1978.38(1820.99-2146.87) | -0.0934(-0.0953- -0.091) | 4.7847(2.7201-7.5809) | 4.8714(2.7956-7.7075) | 0.0181(-0.0042-0.0390) |
|  | **Female** | 2197.5170(2014.1548-2382.2616) | 2207.10(2023.76-2391.47) | 0.0044(0.0030-0.0057) | 4.8785(2.7957-7.7269) | 4.9136(2.8051-7.8325) | 0.0072(-0.0146-0.0306) |
|  | **Both** | 2190.1337(2005.6770-2374.4215) | 2093.31(1916.51-2270.26) | -0.0442(-0.0456--0.0428) | 4.8315(2.7649-7.6447) | 4.8916(2.7974-7.7673) | 0.0124(-0.0035-0.0280) |
| **Global** | **Male** | 1808.5163(1671.4640-1949.3779) | 1854.53(1714.91-2002.62) | 0.0254(0.0216-0.0290) | 3.9199(2.2286-6.2093) | 4.0146(2.2798-6.4224) | 0.0241(0.0133-0.0347) |
|  | **Female** | 1809.2371(1665.2418-1946.7970) | 1845.85(1697.66-1985.53) | 0.0202(0.0162-0.0245) | 3.7550(2.1350-5.9491) | 3.8547(2.1929-6.1368) | 0.0266(0.0150-0.0380) |
|  | **Both** | 1808.8728(1668.9329-1945.8972. ) | 1850.44(1706.25-1993.74) | 0.0230(0.0197-0.0261) | 3.8252(2.1781-6.0547) | 3.9284(2.2410-6.2459) | 0.0270(0.0185-0.0354) |

Table 27 SDI values for all estimated GBD locations in 2019(marked countries with the highest age-standardized incidence and DALYs , 1990-2019, Page 139-172

| **Socio-Demographic Index values for all estimated GBD 2019 locations, 1990-2019** | | | | | | | | | | | | | | | | | | | | | | | | | | | | | | | **Highest ( in 2019)** | |
| --- | --- | --- | --- | --- | --- | --- | --- | --- | --- | --- | --- | --- | --- | --- | --- | --- | --- | --- | --- | --- | --- | --- | --- | --- | --- | --- | --- | --- | --- | --- | --- | --- |
| **Location** | **1990** | **1991** | **1992** | **1993** | **1994** | **1995** | **1996** | **1997** | **1998** | **1999** | **2000** | **2001** | **2002** | **2003** | **2004** | **2005** | **2006** | **2007** | **2008** | **2009** | **2010** | **2011** | **2012** | **2013** | **2014** | **2015** | **2016** | **2017** | **2018** | **2019** | **Incidence** | **DALYs** |
| Global | 0·511 | 0·516 | 0·521 | 0·525 | 0·529 | 0·534 | 0·538 | 0·542 | 0·547 | 0·551 | 0·556 | 0·561 | 0·566 | 0·571 | 0·576 | 0·581 | 0·586 | 0·591 | 0·596 | 0·601 | 0·607 | 0·612 | 0·616 | 0·621 | 0·626 | 0·631 | 0·635 | 0·641 | 0·647 | 0·651 |  |  |
| Central Europe, eastern Europe, and central Asia | 0·648 | 0·654 | 0·662 | 0·666 | 0·669 | 0·672 | 0·675 | 0·678 | 0·681 | 0·684 | 0·687 | 0·69 | 0·694 | 0·698 | 0·704 | 0·71 | 0·714 | 0·719 | 0·724 | 0·728 | 0·732 | 0·735 | 0·738 | 0·742 | 0·745 | 0·748 | 0·751 | 0·754 | 0·758 | 0·76 |  |  |
| Central Asia | 0·551 | 0·555 | 0·557 | 0·558 | 0·559 | 0·559 | 0·56 | 0·56 | 0·561 | 0·563 | 0·566 | 0·569 | 0·574 | 0·579 | 0·585 | 0·591 | 0·598 | 0·605 | 0·611 | 0·617 | 0·622 | 0·627 | 0·632 | 0·637 | 0·642 | 0·647 | 0·651 | 0·655 | 0·659 | 0·663 |  |  |
| Armenia | 0·536 | 0·541 | 0·541 | 0·542 | 0·544 | 0·546 | 0·55 | 0·554 | 0·559 | 0·564 | 0·57 | 0·577 | 0·586 | 0·596 | 0·606 | 0·616 | 0·626 | 0·637 | 0·647 | 0·652 | 0·658 | 0·662 | 0·666 | 0·67 | 0·673 | 0·676 | 0·679 | 0·682 | 0·686 | 0·689 | A1 | D2 |
| Azerbaijan | 0·576 | 0·578 | 0·579 | 0·578 | 0·576 | 0·573 | 0·569 | 0·565 | 0·561 | 0·559 | 0·559 | 0·561 | 0·564 | 0·569 | 0·575 | 0·583 | 0·594 | 0·607 | 0·619 | 0·628 | 0·637 | 0·645 | 0·652 | 0·658 | 0·664 | 0·669 | 0·673 | 0·677 | 0·68 | 0·683 | A1 | D2 |
| Georgia | 0·654 | 0·658 | 0·657 | 0·654 | 0·65 | 0·644 | 0·638 | 0·633 | 0·628 | 0·625 | 0·624 | 0·626 | 0·63 | 0·636 | 0·641 | 0·646 | 0·651 | 0·656 | 0·66 | 0·663 | 0·665 | 0·668 | 0·672 | 0·676 | 0·68 | 0·684 | 0·688 | 0·693 | 0·697 | 0·702 | A1 | D2 |
| Kazakhstan | 0·602 | 0·606 | 0·611 | 0·615 | 0·619 | 0·622 | 0·625 | 0·628 | 0·63 | 0·632 | 0·635 | 0·639 | 0·644 | 0·649 | 0·655 | 0·661 | 0·667 | 0·674 | 0·679 | 0·683 | 0·688 | 0·692 | 0·696 | 0·7 | 0·704 | 0·708 | 0·712 | 0·716 | 0·72 | 0·723 | A1 | D2 |
| Kyrgyzstan | 0·532 | 0·537 | 0·541 | 0·543 | 0·542 | 0·541 | 0·539 | 0·537 | 0·536 | 0·534 | 0·534 | 0·535 | 0·537 | 0·54 | 0·544 | 0·546 | 0·549 | 0·552 | 0·555 | 0·558 | 0·56 | 0·563 | 0·565 | 0·569 | 0·574 | 0·578 | 0·583 | 0·588 | 0·592 | 0·596 | A1 | D2 |
| Mongolia | 0·465 | 0·47 | 0·475 | 0·48 | 0·484 | 0·49 | 0·495 | 0·501 | 0·506 | 0·512 | 0·517 | 0·523 | 0·528 | 0·534 | 0·539 | 0·545 | 0·55 | 0·555 | 0·56 | 0·563 | 0·566 | 0·57 | 0·575 | 0·579 | 0·584 | 0·588 | 0·592 | 0·597 | 0·601 | 0·606 | A1 | D2 |
| Tajikistan | 0·468 | 0·473 | 0·474 | 0·474 | 0·472 | 0·468 | 0·462 | 0·457 | 0·451 | 0·445 | 0·441 | 0·44 | 0·443 | 0·448 | 0·456 | 0·463 | 0·47 | 0·477 | 0·483 | 0·489 | 0·495 | 0·5 | 0·505 | 0·511 | 0·516 | 0·521 | 0·526 | 0·531 | 0·535 | 0·539 | A1 | D2 |
| Turkmenistan | 0·548 | 0·551 | 0·554 | 0·557 | 0·557 | 0·558 | 0·558 | 0·557 | 0·556 | 0·557 | 0·561 | 0·565 | 0·57 | 0·576 | 0·582 | 0·588 | 0·595 | 0·601 | 0·606 | 0·611 | 0·616 | 0·622 | 0·628 | 0·635 | 0·642 | 0·648 | 0·654 | 0·66 | 0·666 | 0·67 | A1 | D2 |
| Uzbekistan | 0·49 | 0·492 | 0·494 | 0·496 | 0·498 | 0·501 | 0·505 | 0·51 | 0·515 | 0·52 | 0·525 | 0·531 | 0·536 | 0·541 | 0·546 | 0·551 | 0·556 | 0·561 | 0·567 | 0·572 | 0·578 | 0·584 | 0·59 | 0·597 | 0·603 | 0·609 | 0·616 | 0·622 | 0·627 | 0·631 | A1 | D2 |
| Central Europe | 0·641 | 0·647 | 0·652 | 0·658 | 0·665 | 0·672 | 0·678 | 0·683 | 0·689 | 0·695 | 0·702 | 0·709 | 0·715 | 0·72 | 0·726 | 0·731 | 0·736 | 0·74 | 0·745 | 0·75 | 0·756 | 0·76 | 0·764 | 0·768 | 0·771 | 0·775 | 0·778 | 0·781 | 0·785 | 0·788 |  |  |
| Albania | 0·54 | 0·537 | 0·534 | 0·533 | 0·535 | 0·538 | 0·544 | 0·549 | 0·555 | 0·561 | 0·569 | 0·577 | 0·585 | 0·593 | 0·601 | 0·608 | 0·615 | 0·621 | 0·627 | 0·631 | 0·636 | 0·64 | 0·645 | 0·651 | 0·658 | 0·664 | 0·669 | 0·674 | 0·678 | 0·681 |  |  |
| Bosnia and Herzegovina | 0·533 | 0·534 | 0·532 | 0·529 | 0·527 | 0·528 | 0·54 | 0·558 | 0·576 | 0·591 | 0·604 | 0·616 | 0·626 | 0·636 | 0·644 | 0·651 | 0·658 | 0·665 | 0·671 | 0·677 | 0·682 | 0·686 | 0·691 | 0·695 | 0·698 | 0·702 | 0·706 | 0·71 | 0·714 | 0·718 |  |  |
| Bulgaria | 0·631 | 0·641 | 0·648 | 0·656 | 0·666 | 0·671 | 0·676 | 0·681 | 0·677 | 0·675 | 0·68 | 0·688 | 0·693 | 0·697 | 0·701 | 0·706 | 0·71 | 0·715 | 0·718 | 0·724 | 0·733 | 0·737 | 0·74 | 0·743 | 0·746 | 0·75 | 0·752 | 0·755 | 0·76 | 0·764 |  |  |
| Croatia | 0·68 | 0·688 | 0·692 | 0·692 | 0·692 | 0·691 | 0·691 | 0·697 | 0·703 | 0·707 | 0·713 | 0·719 | 0·725 | 0·73 | 0·734 | 0·739 | 0·745 | 0·748 | 0·753 | 0·758 | 0·763 | 0·767 | 0·77 | 0·774 | 0·777 | 0·781 | 0·784 | 0·788 | 0·791 | 0·794 |  |  |
| Czech Republic | 0·688 | 0·696 | 0·705 | 0·718 | 0·736 | 0·748 | 0·755 | 0·76 | 0·765 | 0·771 | 0·776 | 0·782 | 0·786 | 0·79 | 0·794 | 0·798 | 0·801 | 0·804 | 0·807 | 0·81 | 0·813 | 0·816 | 0·818 | 0·819 | 0·82 | 0·82 | 0·82 | 0·822 | 0·825 | 0·828 |  |  |
| Hungary | 0·659 | 0·663 | 0·671 | 0·678 | 0·685 | 0·693 | 0·7 | 0·707 | 0·713 | 0·718 | 0·724 | 0·73 | 0·735 | 0·741 | 0·746 | 0·751 | 0·756 | 0·76 | 0·763 | 0·768 | 0·772 | 0·773 | 0·774 | 0·774 | 0·775 | 0·778 | 0·781 | 0·784 | 0·788 | 0·791 |  |  |
| Montenegro | 0·701 | 0·701 | 0·699 | 0·695 | 0·69 | 0·687 | 0·686 | 0·687 | 0·69 | 0·692 | 0·696 | 0·701 | 0·706 | 0·712 | 0·717 | 0·723 | 0·729 | 0·736 | 0·743 | 0·749 | 0·754 | 0·759 | 0·764 | 0·768 | 0·773 | 0·777 | 0·78 | 0·784 | 0·788 | 0·791 |  |  |
| North Macedonia | 0·618 | 0·62 | 0·623 | 0·625 | 0·627 | 0·631 | 0·635 | 0·64 | 0·646 | 0·651 | 0·656 | 0·662 | 0·668 | 0·674 | 0·679 | 0·684 | 0·689 | 0·694 | 0·7 | 0·704 | 0·709 | 0·713 | 0·717 | 0·722 | 0·726 | 0·73 | 0·734 | 0·738 | 0·741 | 0·744 |  |  |
| Poland | 0·632 | 0·637 | 0·644 | 0·653 | 0·661 | 0·67 | 0·677 | 0·685 | 0·693 | 0·701 | 0·709 | 0·717 | 0·724 | 0·73 | 0·735 | 0·74 | 0·743 | 0·747 | 0·752 | 0·757 | 0·763 | 0·77 | 0·775 | 0·78 | 0·784 | 0·788 | 0·791 | 0·795 | 0·798 | 0·802 |  |  |
| Romania | 0·625 | 0·632 | 0·635 | 0·638 | 0·643 | 0·649 | 0·653 | 0·655 | 0·659 | 0·664 | 0·669 | 0·677 | 0·682 | 0·686 | 0·693 | 0·698 | 0·702 | 0·707 | 0·711 | 0·718 | 0·726 | 0·729 | 0·734 | 0·74 | 0·741 | 0·744 | 0·747 | 0·752 | 0·756 | 0·76 |  |  |
| Serbia | 0·626 | 0·635 | 0·639 | 0·639 | 0·64 | 0·64 | 0·644 | 0·647 | 0·651 | 0·657 | 0·661 | 0·665 | 0·67 | 0·676 | 0·685 | 0·694 | 0·702 | 0·709 | 0·716 | 0·723 | 0·729 | 0·735 | 0·739 | 0·744 | 0·748 | 0·753 | 0·756 | 0·76 | 0·763 | 0·767 |  |  |
| Slovakia | 0·656 | 0·662 | 0·668 | 0·679 | 0·693 | 0·702 | 0·709 | 0·716 | 0·724 | 0·731 | 0·739 | 0·746 | 0·752 | 0·756 | 0·76 | 0·766 | 0·772 | 0·777 | 0·781 | 0·784 | 0·789 | 0·794 | 0·798 | 0·801 | 0·803 | 0·803 | 0·804 | 0·805 | 0·808 | 0·812 |  |  |
| Slovenia | 0·726 | 0·731 | 0·736 | 0·741 | 0·746 | 0·751 | 0·756 | 0·762 | 0·768 | 0·774 | 0·78 | 0·787 | 0·793 | 0·797 | 0·802 | 0·807 | 0·811 | 0·814 | 0·818 | 0·82 | 0·822 | 0·824 | 0·825 | 0·827 | 0·829 | 0·831 | 0·833 | 0·835 | 0·838 | 0·84 |  |  |
| Eastern Europe | 0·68 | 0·687 | 0·697 | 0·702 | 0·702 | 0·705 | 0·707 | 0·708 | 0·709 | 0·711 | 0·711 | 0·713 | 0·716 | 0·72 | 0·727 | 0·734 | 0·74 | 0·745 | 0·751 | 0·757 | 0·762 | 0·765 | 0·768 | 0·772 | 0·777 | 0·781 | 0·785 | 0·788 | 0·791 | 0·793 |  |  |
| Belarus | 0·591 | 0·595 | 0·6 | 0·606 | 0·611 | 0·614 | 0·618 | 0·62 | 0·621 | 0·624 | 0·629 | 0·635 | 0·642 | 0·65 | 0·658 | 0·665 | 0·671 | 0·678 | 0·687 | 0·695 | 0·703 | 0·709 | 0·713 | 0·719 | 0·725 | 0·73 | 0·734 | 0·738 | 0·742 | 0·745 |  |  |
| Estonia | 0·665 | 0·676 | 0·687 | 0·696 | 0·7 | 0·705 | 0·711 | 0·717 | 0·721 | 0·726 | 0·733 | 0·741 | 0·748 | 0·753 | 0·759 | 0·765 | 0·771 | 0·777 | 0·785 | 0·792 | 0·798 | 0·804 | 0·809 | 0·813 | 0·817 | 0·821 | 0·825 | 0·829 | 0·833 | 0·835 |  | D2 |
| Latvia | 0·675 | 0·682 | 0·691 | 0·7 | 0·708 | 0·713 | 0·716 | 0·719 | 0·721 | 0·723 | 0·727 | 0·733 | 0·739 | 0·745 | 0·753 | 0·76 | 0·766 | 0·774 | 0·784 | 0·793 | 0·797 | 0·798 | 0·801 | 0·803 | 0·804 | 0·805 | 0·809 | 0·813 | 0·817 | 0·82 |  |  |
| Lithuania | 0·67 | 0·672 | 0·682 | 0·691 | 0·694 | 0·696 | 0·7 | 0·705 | 0·709 | 0·714 | 0·723 | 0·73 | 0·736 | 0·743 | 0·752 | 0·76 | 0·765 | 0·771 | 0·782 | 0·792 | 0·797 | 0·801 | 0·808 | 0·813 | 0·817 | 0·822 | 0·829 | 0·835 | 0·839 | 0·843 |  |  |
| Moldova | 0·585 | 0·589 | 0·591 | 0·594 | 0·594 | 0·595 | 0·594 | 0·593 | 0·591 | 0·587 | 0·585 | 0·585 | 0·588 | 0·593 | 0·6 | 0·607 | 0·615 | 0·622 | 0·63 | 0·637 | 0·644 | 0·651 | 0·658 | 0·665 | 0·672 | 0·677 | 0·683 | 0·688 | 0·693 | 0·696 |  |  |
| Russia | 0·695 | 0·703 | 0·716 | 0·72 | 0·719 | 0·722 | 0·724 | 0·725 | 0·726 | 0·728 | 0·728 | 0·728 | 0·73 | 0·734 | 0·741 | 0·749 | 0·754 | 0·759 | 0·764 | 0·77 | 0·775 | 0·777 | 0·779 | 0·784 | 0·788 | 0·793 | 0·797 | 0·801 | 0·803 | 0·805 |  |  |
| Ukraine | 0·653 | 0·657 | 0·661 | 0·665 | 0·666 | 0·667 | 0·667 | 0·667 | 0·666 | 0·665 | 0·664 | 0·665 | 0·668 | 0·672 | 0·679 | 0·685 | 0·692 | 0·699 | 0·706 | 0·71 | 0·713 | 0·718 | 0·721 | 0·725 | 0·727 | 0·729 | 0·73 | 0·732 | 0·734 | 0·736 |  |  |
| High income | 0·755 | 0·76 | 0·765 | 0·769 | 0·773 | 0·777 | 0·78 | 0·783 | 0·786 | 0·788 | 0·791 | 0·795 | 0·799 | 0·801 | 0·804 | 0·806 | 0·807 | 0·809 | 0·812 | 0·816 | 0·82 | 0·823 | 0·826 | 0·829 | 0·832 | 0·835 | 0·839 | 0·842 | 0·845 | 0·847 |  |  |
| Australasia | 0·742 | 0·746 | 0·749 | 0·753 | 0·757 | 0·761 | 0·765 | 0·769 | 0·773 | 0·777 | 0·781 | 0·785 | 0·789 | 0·793 | 0·797 | 0·799 | 0·799 | 0·8 | 0·803 | 0·807 | 0·81 | 0·812 | 0·816 | 0·821 | 0·825 | 0·828 | 0·832 | 0·835 | 0·837 | 0·84 |  |  |
| Australia | 0·738 | 0·741 | 0·745 | 0·749 | 0·753 | 0·757 | 0·761 | 0·766 | 0·77 | 0·774 | 0·778 | 0·782 | 0·787 | 0·791 | 0·795 | 0·797 | 0·798 | 0·799 | 0·802 | 0·806 | 0·809 | 0·812 | 0·815 | 0·82 | 0·824 | 0·828 | 0·832 | 0·834 | 0·837 | 0·839 |  |  |
| New Zealand | 0·757 | 0·762 | 0·765 | 0·769 | 0·772 | 0·774 | 0·778 | 0·782 | 0·785 | 0·787 | 0·79 | 0·794 | 0·796 | 0·798 | 0·802 | 0·803 | 0·8 | 0·8 | 0·803 | 0·807 | 0·809 | 0·812 | 0·816 | 0·821 | 0·825 | 0·828 | 0·832 | 0·835 | 0·838 | 0·84 |  |  |
| High-income Asia Pacific | 0·767 | 0·773 | 0·779 | 0·785 | 0·79 | 0·796 | 0·801 | 0·805 | 0·809 | 0·813 | 0·816 | 0·819 | 0·823 | 0·826 | 0·83 | 0·833 | 0·836 | 0·839 | 0·842 | 0·844 | 0·847 | 0·85 | 0·853 | 0·856 | 0·859 | 0·862 | 0·865 | 0·868 | 0·871 | 0·873 |  |  |
| Brunei | 0·676 | 0·682 | 0·688 | 0·694 | 0·7 | 0·706 | 0·712 | 0·717 | 0·723 | 0·729 | 0·735 | 0·741 | 0·747 | 0·753 | 0·758 | 0·764 | 0·769 | 0·774 | 0·779 | 0·784 | 0·789 | 0·793 | 0·797 | 0·801 | 0·806 | 0·809 | 0·813 | 0·817 | 0·82 | 0·823 |  |  |
| Japan | 0·791 | 0·796 | 0·801 | 0·805 | 0·809 | 0·813 | 0·817 | 0·82 | 0·822 | 0·824 | 0·826 | 0·828 | 0·83 | 0·833 | 0·836 | 0·838 | 0·84 | 0·842 | 0·844 | 0·846 | 0·848 | 0·85 | 0·853 | 0·855 | 0·857 | 0·86 | 0·862 | 0·865 | 0·867 | 0·87 |  | D2 A2 |
| Aichi | 0·801 | 0·806 | 0·811 | 0·816 | 0·82 | 0·825 | 0·828 | 0·831 | 0·833 | 0·835 | 0·837 | 0·838 | 0·841 | 0·843 | 0·846 | 0·848 | 0·85 | 0·852 | 0·854 | 0·856 | 0·859 | 0·861 | 0·864 | 0·866 | 0·869 | 0·871 | 0·874 | 0·876 | 0·879 | 0·881 |  |  |
| Akita | 0·746 | 0·75 | 0·754 | 0·759 | 0·763 | 0·768 | 0·772 | 0·776 | 0·779 | 0·781 | 0·784 | 0·786 | 0·789 | 0·791 | 0·794 | 0·796 | 0·798 | 0·8 | 0·802 | 0·803 | 0·806 | 0·808 | 0·811 | 0·813 | 0·816 | 0·819 | 0·822 | 0·824 | 0·827 | 0·83 |  |  |
| Aomori | 0·736 | 0·74 | 0·745 | 0·749 | 0·754 | 0·758 | 0·762 | 0·766 | 0·768 | 0·771 | 0·773 | 0·775 | 0·779 | 0·782 | 0·786 | 0·789 | 0·791 | 0·794 | 0·796 | 0·798 | 0·8 | 0·803 | 0·806 | 0·809 | 0·811 | 0·814 | 0·817 | 0·82 | 0·823 | 0·826 |  |  |
| Chiba | 0·79 | 0·794 | 0·8 | 0·804 | 0·808 | 0·813 | 0·817 | 0·82 | 0·822 | 0·824 | 0·825 | 0·826 | 0·827 | 0·829 | 0·832 | 0·834 | 0·835 | 0·837 | 0·838 | 0·84 | 0·842 | 0·844 | 0·846 | 0·848 | 0·85 | 0·852 | 0·855 | 0·857 | 0·859 | 0·861 |  |  |
| Ehime | 0·757 | 0·761 | 0·766 | 0·771 | 0·775 | 0·78 | 0·784 | 0·788 | 0·791 | 0·795 | 0·797 | 0·8 | 0·802 | 0·805 | 0·807 | 0·809 | 0·811 | 0·812 | 0·814 | 0·816 | 0·818 | 0·82 | 0·822 | 0·824 | 0·827 | 0·83 | 0·833 | 0·836 | 0·838 | 0·841 |  |  |
| Fukui | 0·767 | 0·772 | 0·778 | 0·782 | 0·787 | 0·791 | 0·795 | 0·799 | 0·802 | 0·806 | 0·808 | 0·811 | 0·813 | 0·816 | 0·818 | 0·821 | 0·823 | 0·826 | 0·828 | 0·831 | 0·833 | 0·836 | 0·838 | 0·84 | 0·842 | 0·844 | 0·846 | 0·849 | 0·851 | 0·854 |  |  |
| Fukuoka | 0·783 | 0·786 | 0·791 | 0·795 | 0·799 | 0·803 | 0·807 | 0·81 | 0·813 | 0·815 | 0·818 | 0·82 | 0·822 | 0·825 | 0·828 | 0·829 | 0·831 | 0·832 | 0·833 | 0·835 | 0·837 | 0·84 | 0·842 | 0·844 | 0·846 | 0·848 | 0·85 | 0·853 | 0·855 | 0·858 |  |  |
| Fukushima | 0·751 | 0·755 | 0·76 | 0·764 | 0·768 | 0·773 | 0·776 | 0·779 | 0·781 | 0·784 | 0·785 | 0·787 | 0·79 | 0·794 | 0·797 | 0·8 | 0·803 | 0·805 | 0·807 | 0·809 | 0·812 | 0·814 | 0·816 | 0·818 | 0·821 | 0·823 | 0·826 | 0·83 | 0·833 | 0·836 |  |  |
| Gifu | 0·771 | 0·776 | 0·781 | 0·786 | 0·79 | 0·795 | 0·798 | 0·802 | 0·804 | 0·806 | 0·808 | 0·81 | 0·813 | 0·815 | 0·818 | 0·821 | 0·823 | 0·825 | 0·826 | 0·828 | 0·831 | 0·833 | 0·836 | 0·838 | 0·84 | 0·842 | 0·845 | 0·847 | 0·849 | 0·852 |  |  |
| Gunma | 0·774 | 0·778 | 0·783 | 0·788 | 0·792 | 0·796 | 0·8 | 0·803 | 0·805 | 0·807 | 0·808 | 0·81 | 0·813 | 0·816 | 0·819 | 0·821 | 0·823 | 0·825 | 0·828 | 0·83 | 0·833 | 0·836 | 0·838 | 0·841 | 0·844 | 0·847 | 0·85 | 0·852 | 0·855 | 0·858 |  |  |
| Hiroshima | 0·788 | 0·793 | 0·798 | 0·802 | 0·807 | 0·811 | 0·815 | 0·818 | 0·821 | 0·823 | 0·825 | 0·827 | 0·829 | 0·832 | 0·834 | 0·836 | 0·837 | 0·839 | 0·84 | 0·841 | 0·843 | 0·846 | 0·849 | 0·851 | 0·854 | 0·857 | 0·859 | 0·862 | 0·865 | 0·868 |  |  |
| Hokkaidō | 0·768 | 0·772 | 0·777 | 0·782 | 0·786 | 0·791 | 0·794 | 0·797 | 0·799 | 0·802 | 0·803 | 0·805 | 0·808 | 0·81 | 0·813 | 0·815 | 0·817 | 0·818 | 0·82 | 0·821 | 0·823 | 0·825 | 0·827 | 0·829 | 0·831 | 0·833 | 0·836 | 0·838 | 0·841 | 0·843 |  |  |
| Hyōgo | 0·794 | 0·798 | 0·804 | 0·808 | 0·812 | 0·817 | 0·82 | 0·823 | 0·826 | 0·828 | 0·83 | 0·832 | 0·833 | 0·835 | 0·837 | 0·838 | 0·839 | 0·841 | 0·842 | 0·844 | 0·845 | 0·848 | 0·85 | 0·852 | 0·854 | 0·857 | 0·859 | 0·862 | 0·864 | 0·866 |  |  |
| Ibaraki | 0·773 | 0·777 | 0·782 | 0·787 | 0·791 | 0·796 | 0·8 | 0·803 | 0·805 | 0·807 | 0·808 | 0·81 | 0·812 | 0·815 | 0·818 | 0·821 | 0·823 | 0·826 | 0·828 | 0·831 | 0·834 | 0·837 | 0·839 | 0·842 | 0·844 | 0·847 | 0·85 | 0·852 | 0·855 | 0·858 |  |  |
| Ishikawa | 0·774 | 0·78 | 0·785 | 0·79 | 0·795 | 0·799 | 0·803 | 0·807 | 0·81 | 0·813 | 0·816 | 0·818 | 0·821 | 0·824 | 0·826 | 0·829 | 0·83 | 0·832 | 0·833 | 0·834 | 0·835 | 0·837 | 0·839 | 0·841 | 0·843 | 0·846 | 0·848 | 0·851 | 0·854 | 0·856 |  |  |
| Iwate | 0·738 | 0·742 | 0·747 | 0·752 | 0·757 | 0·762 | 0·766 | 0·769 | 0·772 | 0·775 | 0·777 | 0·779 | 0·782 | 0·785 | 0·789 | 0·791 | 0·793 | 0·796 | 0·798 | 0·8 | 0·803 | 0·806 | 0·808 | 0·811 | 0·814 | 0·818 | 0·821 | 0·824 | 0·828 | 0·831 |  |  |
| Kagawa | 0·772 | 0·777 | 0·783 | 0·788 | 0·792 | 0·796 | 0·8 | 0·803 | 0·805 | 0·807 | 0·809 | 0·812 | 0·814 | 0·817 | 0·82 | 0·823 | 0·824 | 0·826 | 0·827 | 0·829 | 0·831 | 0·834 | 0·837 | 0·839 | 0·842 | 0·844 | 0·847 | 0·85 | 0·853 | 0·855 |  |  |
| Kagoshima | 0·744 | 0·748 | 0·753 | 0·757 | 0·761 | 0·766 | 0·77 | 0·773 | 0·776 | 0·779 | 0·781 | 0·784 | 0·787 | 0·79 | 0·793 | 0·795 | 0·797 | 0·799 | 0·801 | 0·803 | 0·806 | 0·808 | 0·811 | 0·813 | 0·816 | 0·819 | 0·822 | 0·825 | 0·828 | 0·831 |  |  |
| Kanagawa | 0·82 | 0·824 | 0·828 | 0·832 | 0·836 | 0·84 | 0·843 | 0·846 | 0·848 | 0·849 | 0·85 | 0·851 | 0·853 | 0·855 | 0·857 | 0·859 | 0·86 | 0·862 | 0·864 | 0·865 | 0·867 | 0·869 | 0·87 | 0·872 | 0·873 | 0·875 | 0·877 | 0·878 | 0·88 | 0·882 |  |  |
| Kōchi | 0·744 | 0·748 | 0·752 | 0·757 | 0·761 | 0·766 | 0·77 | 0·774 | 0·777 | 0·781 | 0·784 | 0·787 | 0·79 | 0·793 | 0·796 | 0·798 | 0·799 | 0·801 | 0·803 | 0·805 | 0·807 | 0·81 | 0·812 | 0·815 | 0·817 | 0·82 | 0·823 | 0·826 | 0·829 | 0·832 |  |  |
| Kumamoto | 0·754 | 0·758 | 0·763 | 0·767 | 0·771 | 0·775 | 0·778 | 0·78 | 0·782 | 0·784 | 0·786 | 0·788 | 0·791 | 0·794 | 0·797 | 0·8 | 0·801 | 0·803 | 0·804 | 0·806 | 0·808 | 0·811 | 0·813 | 0·816 | 0·819 | 0·822 | 0·825 | 0·828 | 0·831 | 0·834 |  |  |
| Kyōto | 0·801 | 0·805 | 0·81 | 0·814 | 0·818 | 0·822 | 0·825 | 0·828 | 0·831 | 0·833 | 0·836 | 0·838 | 0·84 | 0·842 | 0·845 | 0·847 | 0·849 | 0·85 | 0·852 | 0·854 | 0·855 | 0·857 | 0·859 | 0·861 | 0·863 | 0·865 | 0·868 | 0·87 | 0·872 | 0·875 |  |  |
| Mie | 0·765 | 0·77 | 0·776 | 0·781 | 0·785 | 0·79 | 0·794 | 0·797 | 0·8 | 0·802 | 0·805 | 0·808 | 0·811 | 0·814 | 0·818 | 0·821 | 0·823 | 0·825 | 0·828 | 0·83 | 0·832 | 0·835 | 0·838 | 0·841 | 0·844 | 0·847 | 0·85 | 0·853 | 0·856 | 0·859 |  |  |
| Miyagi | 0·774 | 0·779 | 0·784 | 0·789 | 0·793 | 0·798 | 0·801 | 0·803 | 0·805 | 0·807 | 0·808 | 0·81 | 0·813 | 0·816 | 0·819 | 0·822 | 0·823 | 0·826 | 0·827 | 0·829 | 0·831 | 0·834 | 0·837 | 0·839 | 0·842 | 0·845 | 0·848 | 0·851 | 0·854 | 0·857 |  |  |
| Miyazaki | 0·741 | 0·744 | 0·749 | 0·752 | 0·755 | 0·759 | 0·763 | 0·767 | 0·771 | 0·774 | 0·777 | 0·779 | 0·782 | 0·785 | 0·788 | 0·791 | 0·792 | 0·794 | 0·795 | 0·797 | 0·799 | 0·802 | 0·805 | 0·807 | 0·81 | 0·814 | 0·817 | 0·82 | 0·823 | 0·826 |  |  |
| Nagano | 0·778 | 0·782 | 0·787 | 0·791 | 0·795 | 0·799 | 0·803 | 0·806 | 0·808 | 0·811 | 0·813 | 0·815 | 0·817 | 0·82 | 0·822 | 0·825 | 0·827 | 0·829 | 0·831 | 0·833 | 0·835 | 0·837 | 0·839 | 0·841 | 0·843 | 0·845 | 0·848 | 0·851 | 0·853 | 0·856 |  |  |
| Nagasaki | 0·743 | 0·747 | 0·752 | 0·756 | 0·761 | 0·765 | 0·769 | 0·772 | 0·775 | 0·778 | 0·78 | 0·783 | 0·785 | 0·787 | 0·79 | 0·792 | 0·794 | 0·796 | 0·797 | 0·799 | 0·801 | 0·804 | 0·807 | 0·81 | 0·813 | 0·815 | 0·818 | 0·821 | 0·824 | 0·827 |  |  |
| Nara | 0·779 | 0·784 | 0·789 | 0·794 | 0·799 | 0·803 | 0·807 | 0·81 | 0·812 | 0·815 | 0·817 | 0·819 | 0·822 | 0·824 | 0·826 | 0·827 | 0·829 | 0·83 | 0·831 | 0·833 | 0·834 | 0·836 | 0·837 | 0·839 | 0·841 | 0·843 | 0·845 | 0·847 | 0·849 | 0·851 |  |  |
| Niigata | 0·758 | 0·762 | 0·767 | 0·772 | 0·776 | 0·78 | 0·784 | 0·788 | 0·791 | 0·794 | 0·796 | 0·799 | 0·803 | 0·805 | 0·808 | 0·811 | 0·813 | 0·815 | 0·817 | 0·819 | 0·821 | 0·823 | 0·826 | 0·829 | 0·832 | 0·834 | 0·837 | 0·84 | 0·842 | 0·845 |  |  |
| Ōita | 0·765 | 0·769 | 0·774 | 0·779 | 0·783 | 0·787 | 0·791 | 0·795 | 0·798 | 0·8 | 0·803 | 0·805 | 0·808 | 0·811 | 0·814 | 0·817 | 0·818 | 0·82 | 0·821 | 0·823 | 0·825 | 0·827 | 0·83 | 0·832 | 0·834 | 0·836 | 0·839 | 0·841 | 0·844 | 0·847 |  |  |
| Okayama | 0·778 | 0·783 | 0·788 | 0·792 | 0·796 | 0·8 | 0·804 | 0·807 | 0·809 | 0·812 | 0·814 | 0·816 | 0·819 | 0·822 | 0·825 | 0·827 | 0·829 | 0·831 | 0·833 | 0·836 | 0·838 | 0·841 | 0·843 | 0·845 | 0·848 | 0·85 | 0·853 | 0·855 | 0·858 | 0·861 |  |  |
| Okinawa | 0·736 | 0·74 | 0·745 | 0·749 | 0·753 | 0·757 | 0·76 | 0·763 | 0·764 | 0·766 | 0·768 | 0·77 | 0·774 | 0·778 | 0·782 | 0·785 | 0·787 | 0·789 | 0·791 | 0·793 | 0·795 | 0·798 | 0·8 | 0·802 | 0·804 | 0·807 | 0·81 | 0·813 | 0·816 | 0·819 |  |  |
| Ōsaka | 0·806 | 0·81 | 0·816 | 0·82 | 0·824 | 0·828 | 0·832 | 0·834 | 0·837 | 0·839 | 0·841 | 0·842 | 0·844 | 0·846 | 0·848 | 0·849 | 0·85 | 0·852 | 0·853 | 0·854 | 0·856 | 0·858 | 0·86 | 0·862 | 0·864 | 0·866 | 0·869 | 0·871 | 0·873 | 0·875 |  |  |
| Saga | 0·752 | 0·756 | 0·761 | 0·765 | 0·77 | 0·774 | 0·778 | 0·781 | 0·783 | 0·786 | 0·788 | 0·79 | 0·794 | 0·797 | 0·8 | 0·802 | 0·804 | 0·806 | 0·808 | 0·81 | 0·812 | 0·814 | 0·816 | 0·818 | 0·82 | 0·822 | 0·825 | 0·828 | 0·831 | 0·834 |  |  |
| Saitama | 0·783 | 0·788 | 0·793 | 0·798 | 0·802 | 0·806 | 0·81 | 0·813 | 0·816 | 0·817 | 0·818 | 0·819 | 0·821 | 0·823 | 0·826 | 0·828 | 0·829 | 0·831 | 0·833 | 0·835 | 0·837 | 0·839 | 0·841 | 0·843 | 0·845 | 0·847 | 0·849 | 0·851 | 0·854 | 0·856 |  |  |
| Shiga | 0·794 | 0·799 | 0·805 | 0·81 | 0·814 | 0·819 | 0·823 | 0·826 | 0·829 | 0·831 | 0·833 | 0·835 | 0·837 | 0·84 | 0·843 | 0·845 | 0·847 | 0·849 | 0·85 | 0·852 | 0·853 | 0·855 | 0·858 | 0·86 | 0·862 | 0·864 | 0·867 | 0·869 | 0·872 | 0·874 |  |  |
| Shimane | 0·74 | 0·745 | 0·75 | 0·754 | 0·759 | 0·764 | 0·768 | 0·773 | 0·776 | 0·78 | 0·783 | 0·785 | 0·788 | 0·792 | 0·795 | 0·798 | 0·801 | 0·803 | 0·805 | 0·807 | 0·809 | 0·812 | 0·815 | 0·818 | 0·821 | 0·824 | 0·827 | 0·83 | 0·833 | 0·836 |  |  |
| Shizuoka | 0·781 | 0·785 | 0·79 | 0·794 | 0·799 | 0·803 | 0·807 | 0·81 | 0·812 | 0·814 | 0·816 | 0·817 | 0·82 | 0·823 | 0·827 | 0·83 | 0·831 | 0·834 | 0·836 | 0·838 | 0·84 | 0·843 | 0·845 | 0·848 | 0·851 | 0·853 | 0·856 | 0·858 | 0·861 | 0·864 |  |  |
| Tochigi | 0·773 | 0·778 | 0·783 | 0·788 | 0·793 | 0·797 | 0·8 | 0·803 | 0·805 | 0·807 | 0·809 | 0·811 | 0·813 | 0·816 | 0·819 | 0·822 | 0·824 | 0·827 | 0·829 | 0·831 | 0·834 | 0·836 | 0·839 | 0·841 | 0·844 | 0·847 | 0·851 | 0·854 | 0·857 | 0·859 |  |  |
| Tokushima | 0·755 | 0·76 | 0·766 | 0·771 | 0·775 | 0·779 | 0·783 | 0·787 | 0·79 | 0·794 | 0·797 | 0·8 | 0·804 | 0·808 | 0·812 | 0·816 | 0·818 | 0·821 | 0·823 | 0·825 | 0·828 | 0·831 | 0·833 | 0·836 | 0·839 | 0·842 | 0·845 | 0·848 | 0·851 | 0·854 |  |  |
| Tōkyō | 0·867 | 0·872 | 0·877 | 0·881 | 0·884 | 0·888 | 0·891 | 0·894 | 0·896 | 0·897 | 0·898 | 0·899 | 0·9 | 0·902 | 0·904 | 0·906 | 0·907 | 0·908 | 0·91 | 0·912 | 0·913 | 0·915 | 0·917 | 0·918 | 0·92 | 0·921 | 0·923 | 0·924 | 0·925 | 0·927 |  |  |
| Tottori | 0·758 | 0·762 | 0·767 | 0·772 | 0·776 | 0·781 | 0·785 | 0·788 | 0·791 | 0·794 | 0·796 | 0·798 | 0·8 | 0·802 | 0·805 | 0·807 | 0·808 | 0·81 | 0·812 | 0·813 | 0·815 | 0·816 | 0·818 | 0·82 | 0·822 | 0·825 | 0·827 | 0·83 | 0·833 | 0·835 |  |  |
| Toyama | 0·778 | 0·783 | 0·789 | 0·793 | 0·797 | 0·801 | 0·805 | 0·808 | 0·811 | 0·814 | 0·817 | 0·819 | 0·822 | 0·825 | 0·829 | 0·831 | 0·833 | 0·836 | 0·838 | 0·839 | 0·842 | 0·844 | 0·846 | 0·848 | 0·85 | 0·853 | 0·855 | 0·858 | 0·861 | 0·863 |  |  |
| Wakayama | 0·755 | 0·759 | 0·764 | 0·769 | 0·772 | 0·777 | 0·78 | 0·783 | 0·786 | 0·789 | 0·792 | 0·794 | 0·798 | 0·801 | 0·804 | 0·807 | 0·81 | 0·812 | 0·814 | 0·816 | 0·819 | 0·822 | 0·825 | 0·828 | 0·832 | 0·835 | 0·838 | 0·842 | 0·845 | 0·848 |  |  |
| Yamagata | 0·748 | 0·752 | 0·757 | 0·761 | 0·765 | 0·769 | 0·772 | 0·775 | 0·778 | 0·78 | 0·782 | 0·784 | 0·787 | 0·79 | 0·794 | 0·797 | 0·799 | 0·802 | 0·805 | 0·807 | 0·81 | 0·813 | 0·816 | 0·818 | 0·821 | 0·824 | 0·827 | 0·83 | 0·833 | 0·835 |  |  |
| Yamaguchi | 0·771 | 0·775 | 0·78 | 0·783 | 0·787 | 0·791 | 0·795 | 0·798 | 0·801 | 0·804 | 0·806 | 0·808 | 0·811 | 0·814 | 0·817 | 0·82 | 0·821 | 0·824 | 0·825 | 0·828 | 0·83 | 0·833 | 0·836 | 0·839 | 0·841 | 0·844 | 0·847 | 0·849 | 0·852 | 0·855 |  |  |
| Yamanashi | 0·777 | 0·782 | 0·787 | 0·791 | 0·795 | 0·799 | 0·803 | 0·806 | 0·808 | 0·81 | 0·812 | 0·814 | 0·816 | 0·818 | 0·821 | 0·824 | 0·827 | 0·83 | 0·832 | 0·834 | 0·836 | 0·839 | 0·841 | 0·843 | 0·846 | 0·848 | 0·851 | 0·853 | 0·856 | 0·858 |  |  |
| South Korea | 0·686 | 0·697 | 0·708 | 0·719 | 0·73 | 0·74 | 0·75 | 0·76 | 0·767 | 0·776 | 0·784 | 0·791 | 0·799 | 0·805 | 0·811 | 0·817 | 0·823 | 0·828 | 0·833 | 0·837 | 0·842 | 0·846 | 0·851 | 0·855 | 0·859 | 0·863 | 0·867 | 0·871 | 0·875 | 0·878 |  |  |
| Singapore | 0·688 | 0·697 | 0·705 | 0·714 | 0·723 | 0·731 | 0·74 | 0·749 | 0·756 | 0·762 | 0·769 | 0·776 | 0·783 | 0·789 | 0·794 | 0·801 | 0·808 | 0·814 | 0·822 | 0·828 | 0·835 | 0·839 | 0·843 | 0·847 | 0·85 | 0·852 | 0·855 | 0·858 | 0·86 | 0·861 |  |  |
| High-income North America | 0·771 | 0·773 | 0·777 | 0·78 | 0·784 | 0·787 | 0·79 | 0·792 | 0·794 | 0·797 | 0·8 | 0·805 | 0·809 | 0·811 | 0·814 | 0·815 | 0·814 | 0·817 | 0·822 | 0·828 | 0·834 | 0·837 | 0·841 | 0·844 | 0·847 | 0·85 | 0·854 | 0·857 | 0·859 | 0·86 |  |  |
| Canada | 0·79 | 0·792 | 0·795 | 0·797 | 0·8 | 0·804 | 0·809 | 0·812 | 0·815 | 0·819 | 0·824 | 0·828 | 0·832 | 0·835 | 0·838 | 0·84 | 0·842 | 0·843 | 0·845 | 0·848 | 0·851 | 0·853 | 0·856 | 0·859 | 0·861 | 0·864 | 0·867 | 0·869 | 0·871 | 0·873 |  |  |
| Greenland | 0·655 | 0·652 | 0·651 | 0·651 | 0·653 | 0·653 | 0·653 | 0·655 | 0·661 | 0·664 | 0·667 | 0·671 | 0·678 | 0·684 | 0·689 | 0·696 | 0·704 | 0·71 | 0·715 | 0·721 | 0·728 | 0·734 | 0·737 | 0·74 | 0·743 | 0·747 | 0·751 | 0·756 | 0·759 | 0·761 |  |  |
| USA | 0·768 | 0·771 | 0·775 | 0·778 | 0·782 | 0·785 | 0·788 | 0·789 | 0·791 | 0·794 | 0·797 | 0·802 | 0·806 | 0·809 | 0·811 | 0·812 | 0·811 | 0·814 | 0·819 | 0·826 | 0·832 | 0·835 | 0·839 | 0·842 | 0·845 | 0·849 | 0·853 | 0·856 | 0·858 | 0·859 | D1 C1 | C2 |
| Alabama | 0·732 | 0·736 | 0·741 | 0·745 | 0·749 | 0·751 | 0·753 | 0·753 | 0·754 | 0·757 | 0·761 | 0·767 | 0·772 | 0·776 | 0·779 | 0·779 | 0·777 | 0·78 | 0·786 | 0·794 | 0·801 | 0·805 | 0·809 | 0·811 | 0·812 | 0·814 | 0·816 | 0·818 | 0·819 | 0·819 |  |  |
| Alaska | 0·748 | 0·75 | 0·754 | 0·76 | 0·766 | 0·771 | 0·776 | 0·779 | 0·782 | 0·784 | 0·787 | 0·793 | 0·796 | 0·799 | 0·801 | 0·801 | 0·798 | 0·8 | 0·804 | 0·811 | 0·817 | 0·821 | 0·826 | 0·83 | 0·834 | 0·839 | 0·844 | 0·848 | 0·85 | 0·851 |  |  |
| Arizona | 0·739 | 0·742 | 0·745 | 0·747 | 0·75 | 0·752 | 0·754 | 0·755 | 0·757 | 0·758 | 0·761 | 0·766 | 0·769 | 0·771 | 0·774 | 0·775 | 0·775 | 0·782 | 0·791 | 0·801 | 0·81 | 0·814 | 0·818 | 0·822 | 0·825 | 0·829 | 0·834 | 0·838 | 0·84 | 0·842 |  |  |
| Arkansas | 0·711 | 0·716 | 0·723 | 0·727 | 0·731 | 0·734 | 0·735 | 0·736 | 0·737 | 0·739 | 0·742 | 0·748 | 0·752 | 0·755 | 0·758 | 0·757 | 0·756 | 0·76 | 0·766 | 0·774 | 0·783 | 0·787 | 0·79 | 0·794 | 0·796 | 0·799 | 0·803 | 0·807 | 0·809 | 0·811 |  |  |
| California | 0·757 | 0·757 | 0·76 | 0·762 | 0·767 | 0·772 | 0·778 | 0·783 | 0·788 | 0·793 | 0·798 | 0·804 | 0·809 | 0·811 | 0·814 | 0·815 | 0·816 | 0·82 | 0·825 | 0·831 | 0·838 | 0·841 | 0·845 | 0·848 | 0·852 | 0·856 | 0·861 | 0·865 | 0·868 | 0·87 |  |  |
| Colorado | 0·788 | 0·791 | 0·794 | 0·798 | 0·801 | 0·803 | 0·805 | 0·806 | 0·807 | 0·808 | 0·81 | 0·815 | 0·818 | 0·821 | 0·824 | 0·825 | 0·825 | 0·829 | 0·834 | 0·84 | 0·847 | 0·851 | 0·855 | 0·859 | 0·863 | 0·867 | 0·871 | 0·874 | 0·876 | 0·877 |  |  |
| Connecticut | 0·829 | 0·832 | 0·835 | 0·838 | 0·841 | 0·843 | 0·845 | 0·847 | 0·849 | 0·852 | 0·855 | 0·86 | 0·864 | 0·865 | 0·867 | 0·868 | 0·868 | 0·87 | 0·873 | 0·877 | 0·881 | 0·884 | 0·887 | 0·89 | 0·893 | 0·896 | 0·899 | 0·901 | 0·902 | 0·902 |  |  |
| Delaware | 0·787 | 0·791 | 0·796 | 0·798 | 0·801 | 0·803 | 0·805 | 0·805 | 0·806 | 0·808 | 0·81 | 0·815 | 0·818 | 0·82 | 0·822 | 0·822 | 0·822 | 0·826 | 0·831 | 0·838 | 0·843 | 0·847 | 0·85 | 0·852 | 0·854 | 0·857 | 0·859 | 0·861 | 0·862 | 0·863 |  |  |
| Washington, DC | 0·784 | 0·788 | 0·793 | 0·798 | 0·805 | 0·812 | 0·82 | 0·826 | 0·833 | 0·839 | 0·846 | 0·853 | 0·859 | 0·862 | 0·865 | 0·865 | 0·865 | 0·867 | 0·869 | 0·872 | 0·876 | 0·877 | 0·879 | 0·88 | 0·881 | 0·883 | 0·884 | 0·886 | 0·886 | 0·886 |  |  |
| Florida | 0·759 | 0·763 | 0·768 | 0·773 | 0·777 | 0·78 | 0·783 | 0·784 | 0·786 | 0·789 | 0·792 | 0·798 | 0·802 | 0·804 | 0·807 | 0·807 | 0·807 | 0·812 | 0·818 | 0·826 | 0·832 | 0·836 | 0·839 | 0·842 | 0·844 | 0·846 | 0·85 | 0·852 | 0·854 | 0·856 |  |  |
| Georgia | 0·742 | 0·747 | 0·752 | 0·757 | 0·761 | 0·764 | 0·767 | 0·768 | 0·768 | 0·769 | 0·772 | 0·777 | 0·78 | 0·783 | 0·785 | 0·785 | 0·784 | 0·789 | 0·796 | 0·804 | 0·812 | 0·817 | 0·821 | 0·825 | 0·828 | 0·832 | 0·835 | 0·838 | 0·84 | 0·841 |  |  |
| Hawaii | 0·769 | 0·771 | 0·775 | 0·78 | 0·785 | 0·791 | 0·796 | 0·799 | 0·802 | 0·804 | 0·806 | 0·811 | 0·814 | 0·817 | 0·82 | 0·819 | 0·818 | 0·821 | 0·825 | 0·83 | 0·836 | 0·84 | 0·843 | 0·847 | 0·85 | 0·853 | 0·856 | 0·858 | 0·859 | 0·86 |  |  |
| Idaho | 0·744 | 0·748 | 0·753 | 0·758 | 0·762 | 0·765 | 0·767 | 0·768 | 0·769 | 0·77 | 0·772 | 0·777 | 0·78 | 0·781 | 0·784 | 0·783 | 0·782 | 0·786 | 0·792 | 0·8 | 0·807 | 0·81 | 0·813 | 0·815 | 0·817 | 0·82 | 0·823 | 0·825 | 0·827 | 0·827 |  |  |
| Illinois | 0·775 | 0·777 | 0·781 | 0·784 | 0·788 | 0·792 | 0·796 | 0·799 | 0·802 | 0·805 | 0·809 | 0·815 | 0·819 | 0·823 | 0·826 | 0·828 | 0·828 | 0·832 | 0·837 | 0·842 | 0·848 | 0·851 | 0·854 | 0·858 | 0·86 | 0·863 | 0·867 | 0·869 | 0·871 | 0·872 |  |  |
| Indiana | 0·758 | 0·761 | 0·766 | 0·769 | 0·772 | 0·774 | 0·775 | 0·776 | 0·777 | 0·779 | 0·782 | 0·787 | 0·791 | 0·793 | 0·795 | 0·794 | 0·792 | 0·795 | 0·8 | 0·806 | 0·812 | 0·816 | 0·819 | 0·823 | 0·826 | 0·829 | 0·833 | 0·835 | 0·837 | 0·838 |  |  |
| Iowa | 0·781 | 0·784 | 0·788 | 0·791 | 0·794 | 0·796 | 0·798 | 0·8 | 0·802 | 0·803 | 0·806 | 0·81 | 0·813 | 0·815 | 0·817 | 0·816 | 0·816 | 0·818 | 0·822 | 0·828 | 0·833 | 0·836 | 0·84 | 0·843 | 0·847 | 0·851 | 0·856 | 0·859 | 0·862 | 0·864 |  |  |
[truncated: 144,203 more chars]
